# Cell type–selective targeting by heterobifunctional protein binders via in-cell enrichment

**DOI:** 10.1038/s41467-026-77460-w

**Published:** 2026-09-17

**Authors:** Ahmed Bulldan, Min Zheng, Christian Meyners, Patrick L. Purder, Johannes Krieger, Johannes K. Dreizler, Thomas M. Geiger, Max L. Repity, Marte Høen Lein, Ingrid Quist-Løkken, Noel Tewes, Ellie R. Smith, Katjana Schwab, Martin Fischer, Martin P. Schwalm, Raghunath Dey, Saran Aswathaman Sivashanmugam, Sarah Schlesiger, Sebastien Moniot, Stefan Knapp, Ingo V. Hartung, Toril Holien, Alexander Loewer, Felix Hausch

**Affiliations:** 1 Department of Biology, Technical University of Darmstadt, Darmstadt, Germany; 2 Department of Chemistry, Technical University of Darmstadt, Darmstadt, Germany; 3 Medicinal Chemistry & Drug Design, Global Research & Development, Merck Healthcare KGaA, Darmstadt, Germany; 4 Department of Immunology and Transfusion Medicine, St. Olav’s University Hospital, Trondheim, Norway; 5 Department of Clinical and Molecular Medicine, Norwegian University of Science and Technology - NTNU, Trondheim, Norway; 6 Computational Biology Group, Leibniz Institute on Aging–Fritz Lipmann Institute (FLI), Jena, Germany; 7 Institute of Medical Biochemistry, Medical Faculty, Otto von Guericke University, Magdeburg, Germany; 8 Institute of Pharmaceutical Chemistry, Goethe University, Frankfurt am Main, Germany; 9 Structural Genomics Consortium (SGC), Buchmann Institute for Life Sciences, Frankfurt am Main, Germany; 10 German Consortium for Translational Cancer research (DKTK) partner site Frankfurt/Mainz, Frankfurt am Main, Germany; 11 Biomolecule Analytics & Proteomics, Site Management Lab Services, Merck KGaA, Darmstadt, Germany; 12 Department of Biomedical Laboratory Science, Norwegian University of Science and Technology - NTNU, Trondheim, Norway; 13 Centre for Synthetic Biology, Technical University of Darmstadt, Darmstadt, Germany; 14 Institute of Pharmaceutical and Biomedical Science (IPBS), Johannes Gutenberg University, Mainz, Germany; 15 Present Address: Zhongshan Institute for Drug Discovery, Shanghai Institute of Materia Medica, Chinese Academy of Sciences, Zhongshan, China; 16 Present Address: Vetter Pharma, Ravensburg, Germany; 17 Present Address: Boehringer Ingelheim Vetmedica GmbH, Ingelheim a.R., Germany; 18 Present Address: Institute of Structural Biology, University of Bonn, Germany

**Keywords:** Drug delivery, Drug discovery and development, Drug delivery, Biochemistry

## Abstract

Non-catalytic heterobifunctional protein binders promise to expand the range of therapeutic options by establishing complexes between key target proteins and accessory presenter proteins equipped with additional properties. Here, we systematically investigate the rational design of such molecules, explore the biochemical basis of complex formation and determine how they achieve cellular efficacy using the endogenously expressed immunophilin FKBP12 as presenter protein and the transcriptional regulator BRD4 as target protein. We present classes of bifunctional molecules that enable selective, FKBP12-dependent killing of specific cell types at subnanomolar concentrations and allow to differentiate between closely related bromodomains of the BET family. We propose that the strongly potentiated efficacy of these bifunctional compounds is based on cellular enrichment through binding to the highly abundant presenter protein FKBP12, a mechanism we term “CellTrap”. Our findings substantiate the concept that highly expressed, non-essential proteins can be repurposed as selective recruiters to expand therapeutic windows of existing small-molecule inhibitors, opening new avenues for designing targeted drugs with improved cell-type specificity.

## Abstract (web summary)

Non-catalytic heterobifunctional protein binders are an exciting new modality offering a range of new therapeutic options. Here the authors show the intracellular enrichment of these molecules caused by high abundance of presenter proteins (CellTrap effect) that leads to a dramatic boost in potency.

## Introduction

**Fig. 1:**
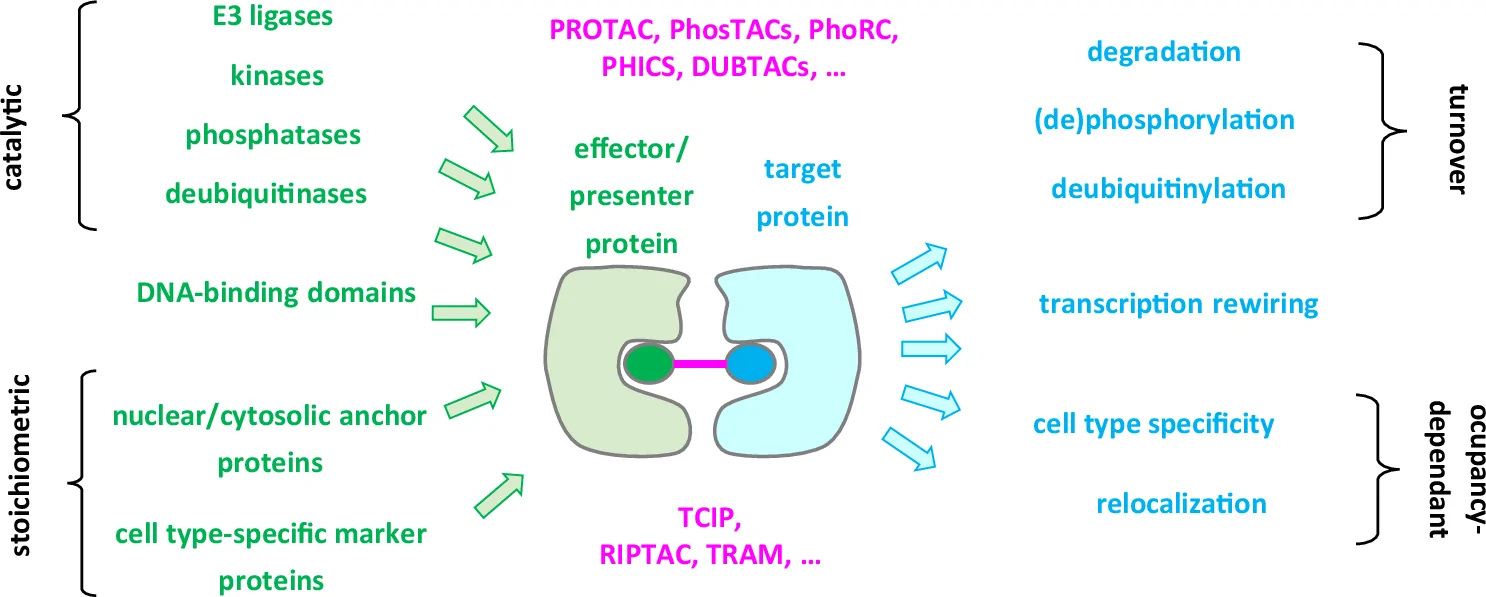
Examples of heterobifunctional drug modalities. Bifunctional molecules comprised of a ligand for the target protein (blue) and a ligand for an effector protein (green) are tethered by a linker (magenta) and allow recruitment of the latter to induce de novo functional effects. Catalytic effectors can operate at sub-stoichiometric drug levels, whereas non-catalytic bifunctionals require persistent target occupancy.

Proximity-inducing drugs are an emerging therapeutic modality with the potential to unlock gain-of-function pharmacology, cell-type specificity, and the ability to target difficult-to-drug proteins. Precedented in nature by molecular glues like FK506 and Rapamycin^1^, the field recently gained substantially preclinical and clinical traction with the use of designed heterobifunctional molecules as chemical inducers of protein-protein interactions (Fig. 1). This is most prominently exemplified by proteolysis targeting chimeras (PROTACs), which co-engage E3 ubiquitin ligases and target proteins to catalytically induce the degradation of the latter^2^. Inspired by PROTACs, several additional concepts of event-driven heterobifunctionals have emerged using phosphatases (PhosTACs, PhoRC), kinases (PHICS), deubiquitinating enzymes (DUBTACs), ribozymes (RIBOTACs), autophagic vesicles (AUTACs), lysosomal compartments (LYTACs), or transcriptional/epigenetic chemical inducers of proximity (TCIPs)^3^ as hijacked effector proteins^4,5^.

More recently, heterobifunctional molecules have been proposed as therapeutics that do not engage enzymatic machineries of effector proteins but rely on sustained target protein occupancy (Fig. 1). These offer intriguing new modes of action. For example, exploiting presenter proteins (i.e., proteins that bind to one part of the heterobifunctional molecules) that are specifically expressed in diseased cells increasing the therapeutic window of existing inhibitors of essential proteins. This can be realized by heterobifunctional RIPTACs (Regulated Induced Proximity-Targeting Chimeras)^6^, the first of which has recently entered clinical trials^7^. A related concept proposes to alter the subcellular localization of target proteins by anchoring them to cell compartment-specific proteins (TRAMs)^8^. Both approaches have been validated by proof-of-concept studies using exogenous overexpressed proteins, but examples for effectively targeting endogenous proteins remain limited^9^. First clinical experience with nondegrading complex-inducing therapeutics has been gained with the immunosuppressants FK506, Rapamycin and Cyclosporin A and related tricomplex-inducing molecular glues^10–12^ that target difficult-to-drug proteins. However, the development of novel tricomplex inhibitors required a decade-long discovery effort with limited rational guidance. Bifunctional molecules that can build on pre-existing ligands for presenter and target proteins can be assembled and explored much faster.

Due to their larger size and higher complexity, bifunctional modalities are in general burdened by unfavorable pharmacological properties (e.g., reduced cell permeability, high cellular efflux, low solubility and resulting poor oral bioavailability). These challenges could be overcome with catalytically acting PROTACs (which require only low intracellular drug concentrations) by property-focused design, which enabled reaching therapeutically active drug exposures in patients after oral dosing^13^. However, these pharmacokinetic hurdles remain relevant for bifunctionals that lack catalytic enhancement and therefore will require higher intracellular drug concentrations. Today, there is a lack of systematic studies on the behavior and performance of this class of molecules, and in turn, their pharmacological prospects remain unclear.

To explore the potential of non-catalytic bifunctionals, we selected FKBP12 and BRD4 as paradigm presenter and target proteins. FKBP12 is the key presenter protein for the natural products FK506 and rapamycin^14^ and enables their molecular glue mechanism as immunosuppressants. Moreover, FKBP12 and engineered versions thereof are abundantly used as adapter proteins for chemically induced protein-protein interactions in cells^1,15,16^. BRD4 is a member of the BET family of epigenetic reader proteins. It regulates the basal transcriptional machinery, mediates protein interactions in super enhancers and plays a crucial role in lineage specification^17^. Among the genes regulated by BRD4 are disease-associated genes such as the oncogene MYC and KEAP1. Inhibition of BRD4 has therefore been suggested as mono- or combination therapy against several tumor entities. However, most clinical studies have so far been hampered by dose-limiting side effects.

In this work, we use these model proteins to investigate the various dimensions that determine the potency and efficacy of non-catalytic bifunctionals. Most significantly, we find that the cell killing potency of standard BRD4 inhibitors can be substantially potentiated in a cell-type specific manner by designing bifunctional molecules comprising highly tunable drug-like FKBP12 binders. We propose a first mathematical model rationalizing our findings to provide a basis for systematically unlocking the potential of this exciting new drug modality.

## Results

### Synthesis of dual BRD4- and FKBP12-binding conjugates

**Fig. 2:**
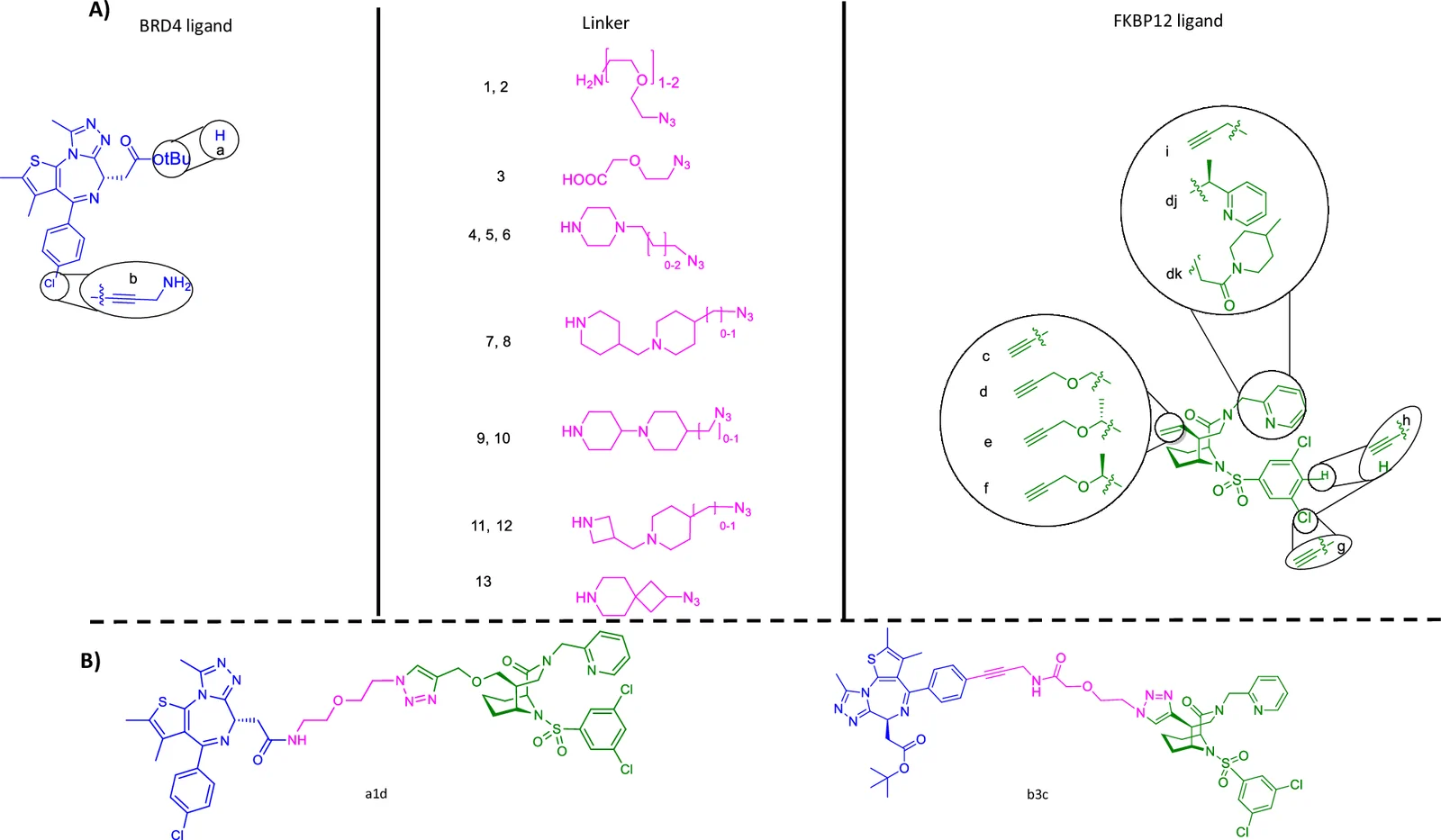
Chemical assembly of the bifunctional BRD4-FKBP12 ligand conjugates. **A** Structures of BRD4 ligands, amine/azide or acid/azide-bifunctionalized linkers and alkyne-functionalized FKBP12 ligands. The attachment points and attachment functionalities are highlighted in the circles with an alphabetical identifier, the linkers are identified by numbers. Note that FKBP12 ligands dj and dk combine the exit vector d with a replacement of the pyridine moiety. **B** Structures of exemplary BRD4-FKBP12 ligand conjugates a1d and b3c.

To generate BRD4-FKBP12 bifunctional molecules, we used the prototypic BET inhibitor (+)-JQ1^18^ and [4.3.1]bicyclic high-affinity ligands for FKBP12^19–22^. For both ligands, the structure-activity relationships are well understood^15,23^, their binding modes are supported by co-crystal structures, and several exit vectors for conjugation have been validated^24^. We first generated a small library of BRD4/FKBP12-targeting heterobifunctional compounds by exploring 2 exit vectors for (+)-JQ1, 13 different amine/azide- or carboxy/azide-bifunctional linkers of varying length, and 9 different FKBP12 ligand variants, each containing an alkyne (Fig. 2A). Our linker selection comprised flexible, partially, and fully rigidified designs including those which have been used in PROTACs with drug-like overall properties.

The (+)-JQ1 derivatives were first coupled to the bifunctional linkers. The resulting intermediates were then conjugated via their azide moieties to the alkyne-functionalized FKBP12 ligands by copper-catalyzed click reaction (Fig. 2B). Overall, 31 BRD4/FKBP12 targeting heterobifunctionals were obtained in this way.

### Biochemical characterization of BRD4-FKBP12 heterobifunctional ligands

**Fig. 3:**
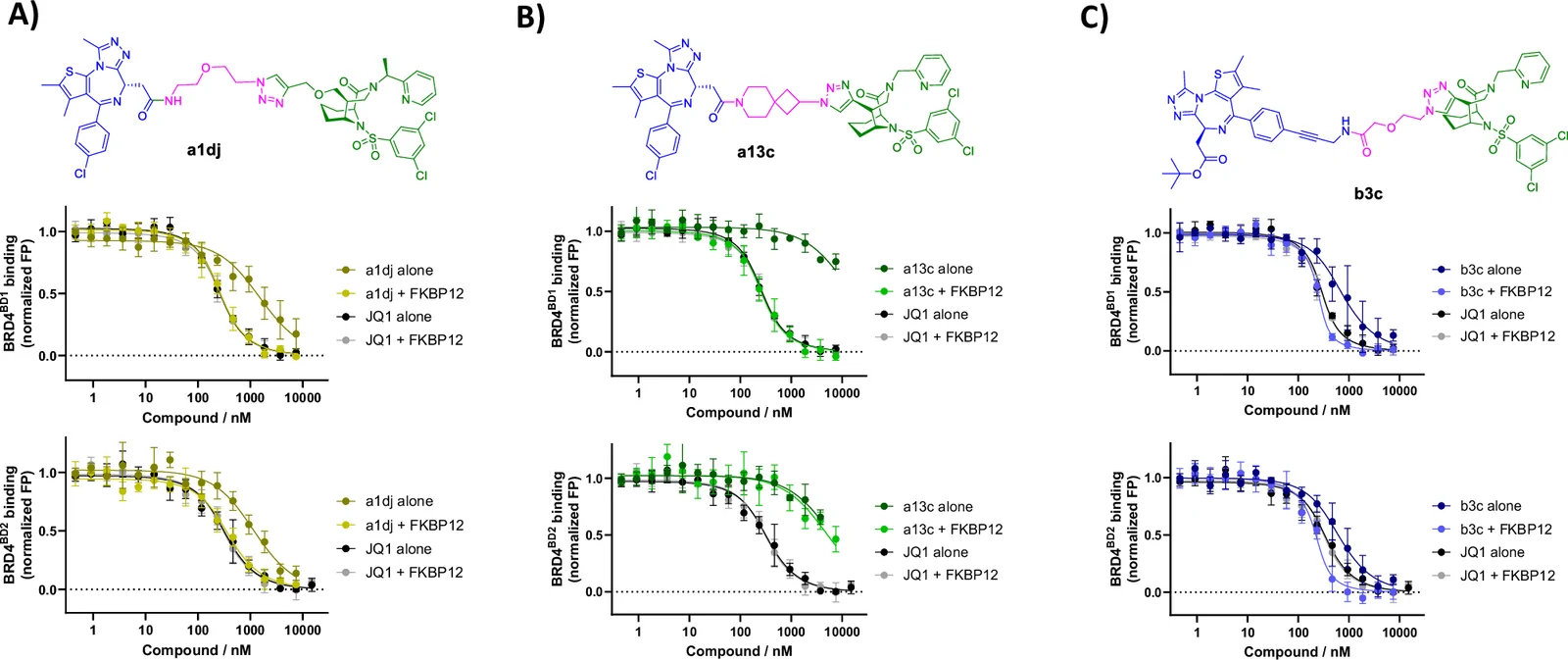
Positive cooperativity and selectivity of BRD4-FKBP12 bifunctional ligands. The binding to BRD4^BD1^ and BRD4^BD2^ of a1dj (**A**), a13c (**B**), b3c (**C**) and JQ1 (**A–C**) as reference was determined alone or in the presence of 15 µM FKBP12 by a competitive FP assay using a fluorescent JQ1 analog as tracer. Data points are indicated as mean from four replicates, and standard deviations are indicated as error bars. The solid lines represent data fitted to a competitive binding model.

With these bifunctionals in hand, we first characterized the binding to FKBP12^25^ and to the isolated BD1 and BD2 domains of BRD4 (Supplementary Fig. 1) by competitive fluorescence polarization (FP) assays. All compounds displayed high to very high affinity for FKBP12 ( < 5 nM for exit vectors c-f, <10 nM for exit vector i, < 100 nM for exit vectors g and h, Table 1 and Supplementary Table 1), in line with previous observations^24^. The binding to BRD4^BD1^ and BRD4^BD2^ was substantially weaker for most conjugates (mean K_i_ = 909 nM), with no differences between BRD4^BD1^ and BRD4^BD2^. To assess simultaneous binding of FKBP12 and BRD4, we repeated the BRD4 binding assays in the presence of 15 µM FKBP12. This further compromised BRD4 binding in several cases (Supplementary Table 1). However, for compounds a1d, a1dj, a13c, and b3c, we observed a > 10-fold positive cooperativity (Fig. 3A–C, Supplementary Fig. 2, and Table 1), i.e., > 10-fold tighter binding to BRD4 in the presence of high concentrations of FKBP12. When pre-bound to FKBP12, these compounds bound to BRD4 as effectively as (+)-JQ1 or even better (for b3c). Of note, compound a13c exhibited high selectivity for BRD4^BD1^ *vs* BRD4^BD2^ in an FKBP12-assisted manner (Table 1 and Fig. 3B). We also tested the advanced compounds a1dj, a13c, and b3c for binding to a protein construct harboring both bromodomains and the connecting endogenous protein sequence (Supplementary Table 2). The selected compounds retained the positive cooperativity and BRD4^BD1^ preference of a13c was retained, indicating that these properties are not influenced by crosstalk between both bromodomains or interactions with the linking sequence.

**Table 1:** Characterization of BRD4-FKBP12 bifunctional ligands

Comp ound	Binding to FKBP12	Binding to BRD4^BD1^ −/+ FKBP12		Binding to BRD4^BD2^ −/+ FKBP12		HTRF BRD4^BD1^ + GFP-FKBP12	HTRF BRD4^BD2^ + GFP -FKBP12	nano BRET FKBP12	nano BRET BRD4	Cell toxicity (MV4-11) +/− FKBP12 PROTAC	
	K_d_ [nM]	K_d_ [nM]	α	K_d_ [nM]	α	K_d_ [nM]	K_d_ [nM]	IC_50_ [nM]	IC_50_ [nM]	IC_50_ [nM]	ratio
JQ1		37 ± 5 39 ± 5	1.0	72 ± 13 64 ± 10	1.1					27 34	0.8
a1d	1.0	450 ± 70 25 ± 4	18	320 ± 50 70 ± 13	4.5	9 ± 3	26 ± 6	14	820	235 1.7	138
a13c	1.7	4600 ± 800 35 ± 5	131	2300 ± 400 1900 ± 300	1.2	13 ± 2	300 ± 30	76	9057	236 7.7	30.6
a1dj	0.4	470 ± 60 39 ± 5	12	400 ± 90 90 ± 18	4.4	15 ± 3	30 ± 7	2.6	653	103 0.09	1144
b3c	3.7	130 ± 20 8 ± 2	16	150 ± 20 13 ± 3	12	1.0 ± 0.6	8 ± 3	29	194	16 0.06	267

Binding of FKBP12-BRD4 ligand conjugates to purified BRD4 BD1 or BD2 bromo domains (BRD4^BD1^ and BRD4^BD2^) was determined in a competitive fluorescence polarization assay in the absence or presence of 15 µM FKBP12 using 2 nM of a fluorescently labeled JQ1-analog as a tracer. Binding to FKBP12 was determined in a competitive fluorescence polarization assay. Direct formation of the ternary complex was determined by a homogeneous time-resolved FRET (HTRF) assay using constant concentrations of eGFP-FKBP12 (5 µM) and His-BRD4^BD1^ or BRD4^BD2^ (100 nM) and serial dilution of bifunctionals. NanoBRET data was determined in intact HEK293 cells by expression of NanoLuc-fusion proteins and using fluorescently labeled FKBP12 or BRD4 ligands. Cell toxicity was measured in MV4-11 cells after five days of treatment. α signifies the binding cooperativity factor calculated as the ratio of binding to BRD4 in the absence or presence of FKBP12.

We also investigated ternary complex formation of the best compounds by a homogeneous time-resolved FRET assay (HTRF, Supplementary Fig. 3). This confirmed the enhanced affinities of the FKBP12-compound complexes towards BRD4 as well as the BD1/BD2 selectivity of a13c (Table 1). Notably, we observed different upper plateaus for the FKBP12-BRD4^BD1^ complexes induced by b3c vs. those induced by a1d, a1dj, or a13c (Supplementary Fig. 3). This indicated the induction of ternary complexes with different architectures, i.e., with different relative orientation of the GFP tag on FKBP12 vs the His tag (complexed in the HTRF assay with a Terbium-labeled antibody) on BRD-BRD4^BD1^.

### Structure of the ternary complexes

**Fig. 4:**
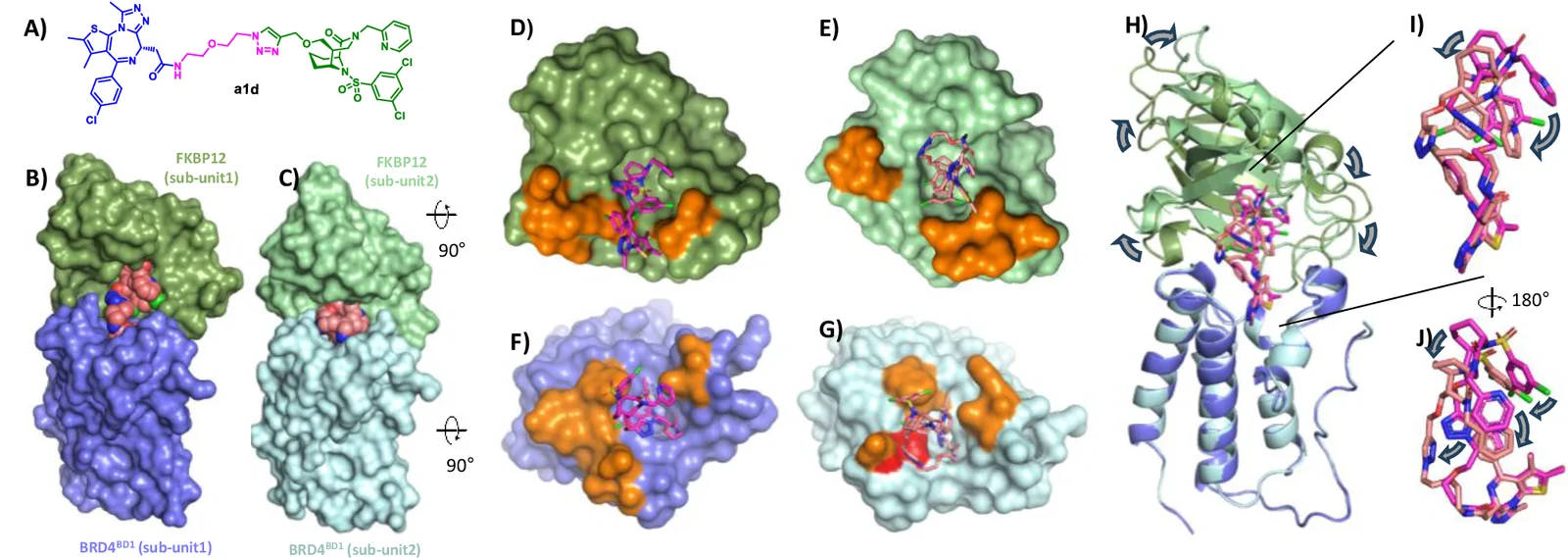
Structure of the FKBP12:a1d:BRD4^BD1^ ternary complex (PDB-ID 9QW8). **A** Chemical structure of a1d. **B**,** C** Two different ternary FKBP12:a1d:BRD4^BD1^ complexes are present in the crystallographic unit cell (sub-complexes a and b). **D**,** E** a1d bound to FKBP12 in the two crystallographic asymmetric units. BRD4^BD1^ is not shown for clarity. **F**,** G** a1d bound to BRD4^BD1^ in the two crystallographic units. FKBP12 is not shown for clarity. **H**–**J** Superposition of the two ternary complexes, aligned on the BRD4^BD1^ subunits. Repositioning of FKBP12 and between the two subunits is indicated by arrows. **I**, **J** Magnification of a1d in the two FKBP12BRD4^BD1^-bound conformations, extracted from (**H**) and viewed from two different angles. **B**–**J** FKBP12 is colored light/dark green, BRD4^BD1^ is colored cyan/dark blue, and compound a1d is colored pale pink/magenta. **D**–**G** Amino residues forming ternary protein-protein contacts are highlighted in orange, and amino acids contacting the linker are colored in red.

To gain more insights into the underlying binding modes, we co-crystallized the ternary complexes of FKBP12:a1d:BRD4^BD1^ (Fig. 4) and FKBP12:b3c:BRD4^BD1^ (Supplementary Fig. 4A). For a1d, two slightly different binding modes were observed comparing the two complexes present in the crystallographic asymmetric unit (Fig. 4A–C). The binding of the [4.3.1] bicyclic sulfonamide to FKBP12 and the JQ1-moiety to BRD4^BD1^ were almost identical to the respective binary complexes (Supplementary Fig. 5), as expected. The two ternary binding modes differed by an approximately 30° rotation of FKBP12 and a tilting relative to BRD4^BD1^ and formed a partially distinct FKBP12-BRD4^BD1^ interaction pattern (Fig. 4D–G and Supplementary Fig. 6A, B, K).

In both ternary complexes, contacts to BRD4^BD1^ are formed by the tip of the so called 80 s loop of FKBP12 (H^87^,P^88^,G^89^,I^90^) and parts of the β2-strand of FKBP12 (D^41^, R^42^, K^44^, P^45^,F^46^,K^47^), (Supplementary Fig. 6A, B). These residues are among the most divergent within the FKBP superfamily. For example, the larger homolog FKBP51 has S^118^L^119^P^120^K^121^ in the 80s-equivalent loop and D^72^, R^73^, E^75^P^76^F^77^V^78^ in the β2-strand. Strikingly, none of the key bifunctional compounds showed a cooperative binding to BD1 or BD2 in the context of FKBP51 (Supplementary Table 3). This strongly supports the crucial role of direct protein-protein contacts for the observed positive cooperativity of the compounds in the context of FKBP12.

In addition to the protein-protein interactions, several ‘crossover’ ligand-protein contacts were observed, from the [4.3.1] bicyclic sulfonamide (i.e., from the FKBP12 ligand) to BRD4^BD1^ and from the JQ1-like structure to FKBP12 (Supplementary Fig. 6C–F). In one complex, the triazole moiety of the linker is involved in interactions with BRD4^BD1^ (Supplementary Fig. 6F, J). Notably, the triazole-contacting residues are different in BRD4^BD1^ (Asp^101^, Asp^102^, and Ile^103^, Supplementary Fig. 6G and J) vs BRD4^BD2^ (His^437^, Glu^438^, and Val^439^). To investigate this, we solved the structure of the a1d-induced ternary complex between FKBP12 and BRD4^BD2^ (Supplementary Fig. 7). We observed a rearrangement of the linker, which in the BRD^BD2^-ternary complexes is stabilized by a hydrogen bond from the backbone of Val^438^ to His^437^, which further forms a hydrogen bond to the oxygen of the linker (Supplementary Fig. 7E, F). The linker conformation in the BRD^BD2^-ternary complexes is similar to the linker conformation of the FKBP12-a1d-BRD4^BD1a^ complex (Supplementary Fig. 7A, B), but FKBP12 can nonetheless adopt a similar orientation as in the FKBP12-a1d-BRD4^BD1b^ complex (Supplementary Fig. 7C). Note that the different interaction patterns of BRD4^BD1^ and BRD4^BD2^ both resulted in energetically similar ternary complexes (Table 1).

In both complexes, substantial intramolecular contacts between the [4.3.1] bicyclic sulfonamide and JQ1-part were observed, and both ligands interacted with the linker (Supplementary Fig. 6G, J). In both complexes, a methyl group in the α-position of the pyridyl group, which distinguishes a1d from a1dj, could be spatially accommodated. Notably, in the FKBP12:b3c:BRD4^BD1^ complex_,_ the relative FKBP12-BRD4^BD1^ orientation was drastically different compared to the FKBP12:a1d:BRD4^BD1^ complexes (Supplementary Fig. 4B, C), with substantially larger distances between the structurally resolved N-termini of FKBP12 and BRD4^BD1^ (48.4 Å and 53.3 Å for the two b3c-induced complexes vs 25.2 Å and 29.4 Å for the two a1d-induced complexes). This is consistent with the reduced HTRF signals for the FKBP12: b3c:BRD4^BD1^ complex compared to those for a1d or a1dj (Supplementary Fig. 3), since this assay relies on N-terminal FRET pairs. b3c was the most potent ternary complex-inducing compound, which in turn could translate into a more rigid ternary complex. This, as well as other effects such as altered antibody accessibility, could have contributed to the reduced HTRF efficacies of the b3c-induced complexes.

Taken together, multiple solutions appear to exist to generate additional binding energy in the ternary complexes, which is crucial for positive cooperativity. This relies on direct protein-protein contacts, protein-non-cognate ligand ‘crossover’ contacts, and linker-protein contacts. Moreover, intramolecular contacts within the bifunctional molecule seem to be important to stabilize the bioactive conformation.

The generated FKBP12-BRD4 ligand conjugates are rather large molecules (> 1000 g/mol), which often have reduced cellular permeability. To assess the potential for cellular application, we investigated intracellular FKBP12 and BRD4 occupancy by nanoBRET assays^26^. Gratifyingly, several FKBP12-BRD4 bifunctionals bound to FKBP12 or BRD4 in cells to a similar extent as the unconjugated precursor molecules (compound FK[4.3.1]-16h^22^ or compound 19-(S^Me^)^19^ for FKBP12 and JQ1 for BRD4), including a1d, a1dj, a13c, and b3c (Tab. 1 and SI Tab. 1). We also generated a stable cell line constitutively expressing BRD4–NanoLuc as a fusion protein and performed NanoBRET assays in the presence or absence of FKBP12 (Supplementary Fig. 8). All bifunctional compounds tested displayed strong BRD4 engagement in the presence of FKBP12, whereas treatment with an FKBP12-targeting PROTAC markedly attenuated the apparent BRD4 occupancy of the bifunctional ligands. In contrast, the monovalent BRD4 ligand JQ1 showed only minimal target engagement and was unaffected by PROTAC treatment, consistent with its FKBP12-independent binding mode. In turn, we set out to explore the performance of these compounds in functional cellular assays.

### BRD4-FKBP12 bifunctional ligands display potent cytotoxicity depending on cellular FKBP12 levels

**Fig. 5:**
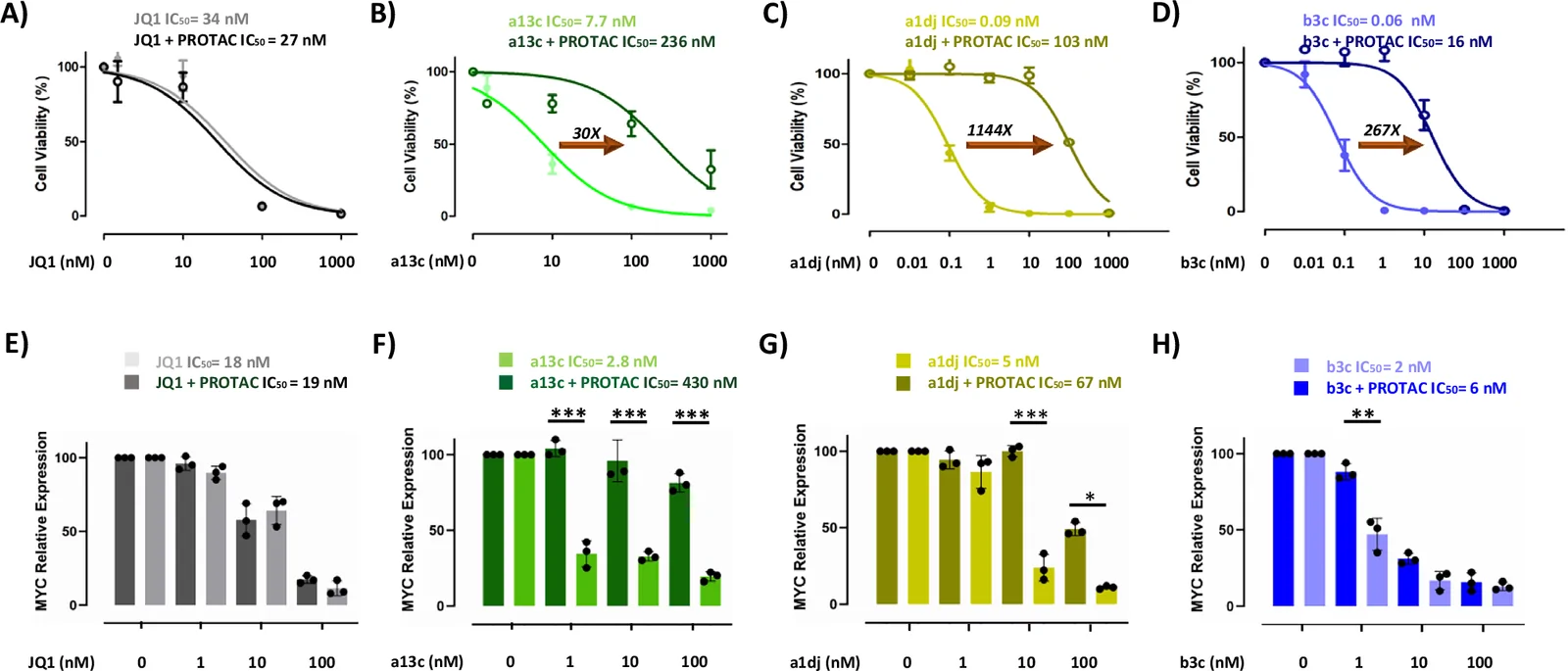
FKBP12-assisted toxicity by BRD4-targeting bifunctionals in MV4-11 cells. **A–D** Cell toxicity for MV4-11 cells treated for five days with the indicated concentrations of (**A**) JQ1, (**B**) a13c, (**C**) a1dj, and (**D**) b3c. All experiments were performed in triplicate, either in the presence or absence of FKBP12 PROTAC. Means and standard errors are shown for each measurement. Lines indicate dose-dependent reduction of cell viability. IC_50_ values in the presence or absence of FKBP12 PROTAC are indicated above each panel. The arrows indicate the fold change. **E–H** Quantitative analysis of MYC mRNA levels in MV4-11 cells following treatment with (**E**) JQ1, **(F**) a13c, (**G**) a1dj, and (**H**) b3c for six hours. These experiments were conducted in the presence or absence of FKBP12 PROTAC. Error bars represent the mean ± standard deviation (SD) from three independent experiments. Statistical analysis was performed using two-way ANOVA (two-sided) followed by Bonferroni’s multiple-comparisons test. For the a13c vs a13c + PROTAC treatment (F(1,4) = 222.5, *P* = 0.0001), concentration (F(3,12) = 52.09, *P* < 0.0001), and the interaction between the two factors (F(3,12) = 31.69, *P* < 0.0001). Bonferroni-adjusted pairwise comparisons at 1 nM (*P* < 0.0001), 10 nM (*P* < 0.0001), and 100 nM (*P* < 0.0001). a1dj vs a1dj + PROTAC (F(1,4) = 434.5, *P* < 0.0001), concentration (F(3,12) = 155.3, *P* < 0.0001), and the treatment × concentration interaction (F(3,12) = 46.84, *P* < 0.0001). Bonferroni-adjusted pairwise comparisons at 10 nM (*P* < 0.0001) and 100 nM (*P* < 0.0001). For the b3c vs b3c + PROTAC treatment (F(1,4) = 75.08, *P* < 0.0001), concentration (F(3,12) = 691.4, *P* < 0.0001), and the treatment × concentration interaction (F(3,12) = 32.19, *P* < 0.0001). Bonferroni-adjusted pairwise comparisons at 1 nM (*P* < 0.0001) and 10 nM (*P* = 0.0012).

First, we evaluated the impact of selected BRD4-FKBP12 bifunctionals on cell viability in MV4-11 cells. This cell line is derived from a hematopoietic malignancy and was chosen based on their known sensitivity to BRD4 inhibition^27^ and high FKBP12 protein level (Supplementary Fig. 8A). In MV4-11 cells, we observed a dose-dependent decrease in cell viability for all compounds tested. Notably, BRD4-FKBP12 bifunctionals showed higher efficacies than JQ1 despite lower binding affinities compared to JQ1 alone (Table 1), with a1dj and b3c reaching sub-nanomolar IC_50_ values (Fig. 5A–D and Supplementary Fig. 9A).

To validate the requirement of ternary complex formation for BRD4 inhibition, we depleted FKBP12 in MV4-11 cells using the FKBP12-targeting PROTAC 5a1 before performing viability assays^24^ (Supplementary Fig. 8B). As expected, JQ1-induced decreases in cell viability were independent of FKBP12 status (Fig. 5A). BRD4-FKBP12 bifunctionals, however, lost their potency in the absence of FKBP12, as indicated by a strong increase of measured IC_50_ values for cell survival in the absence of FKBP12 (Fig. 5B–D and Supplementary Fig. 9A). This effect was most striking for a1dj, where the ratio of IC_50_ values in the presence and absence of FKBP12 exceeded three orders of magnitude (Table 1). Notably, the influence of FKBP12 was concentration-dependent as demonstrated by titration of the FKBP12-targeting PROTAC (Supplementary Fig. 8C, D).

To corroborate that the observed effect of BRD4-FKBP12 bifunctionals on cell viability is based on BRD4 inhibition, we quantified the expression of the known BRD4 target gene MYC at concentrations where we previously observed cytotoxic effects. As expected, JQ1 induced moderate downregulation of MYC at 10 nM and stronger effects at 100 nM, independent of FKBP12 status (Fig. 5E). Again, BRD4-FKBP12 bifunctionals exhibited stronger repression of MYC than JQ1 (Fig. 5F–H and Supplementary Fig. 9B), with b3c inducing notable effects at concentrations as low as 1 nM (Fig. 5H). Importantly, the reduction in MYC expression was attenuated by FKBP12 depletion for the bifunctionals. Due to its high efficacy, rescue of MYC expression by b3c was restricted to 1 nM. At 10 and 100 nM, this compound induced MYC downregulation even after depletion of FKBP12, which was consistent with the observed FKBP12-independent decrease of cell viability at these concentrations (Fig. 5D). Importantly, none of the compounds tested induced degradation of BRD4 (Supplementary Fig. 8E–H and Supplementary Table 4).

Next, we tested cell viability and MYC expression in THP1 cells, which displayed noticeably lower FKBP12 levels than MV4-11 cells (Supplementary Fig. 9A). In THP1 cells, JQ1 showed comparable cytotoxic activity as in MV4-11 (Supplementary Fig. 10A: IC_50_^THP1^ = 70 nM; Fig. 5A: IC_50_^MV4-11^ = 34 nM). The efficacies of FKBP12-BRD4 bifunctionals, however, were strikingly lower in THP1 compared to MV4-11 (10-60 fold), with a13c being even less effective than JQ1 (Supplementary Fig. 10B–E). Moreover, depleting FKBP12 led to a more modest decrease in IC_50_ values for all bifunctionals tested. Interestingly, downregulation of MYC upon BRD4 inhibition was more pronounced in these cells compared to MV4-11 (Supplementary Fig. 10F–J), with similar FKBP12-dependency as observed before.

**Fig. 6:**
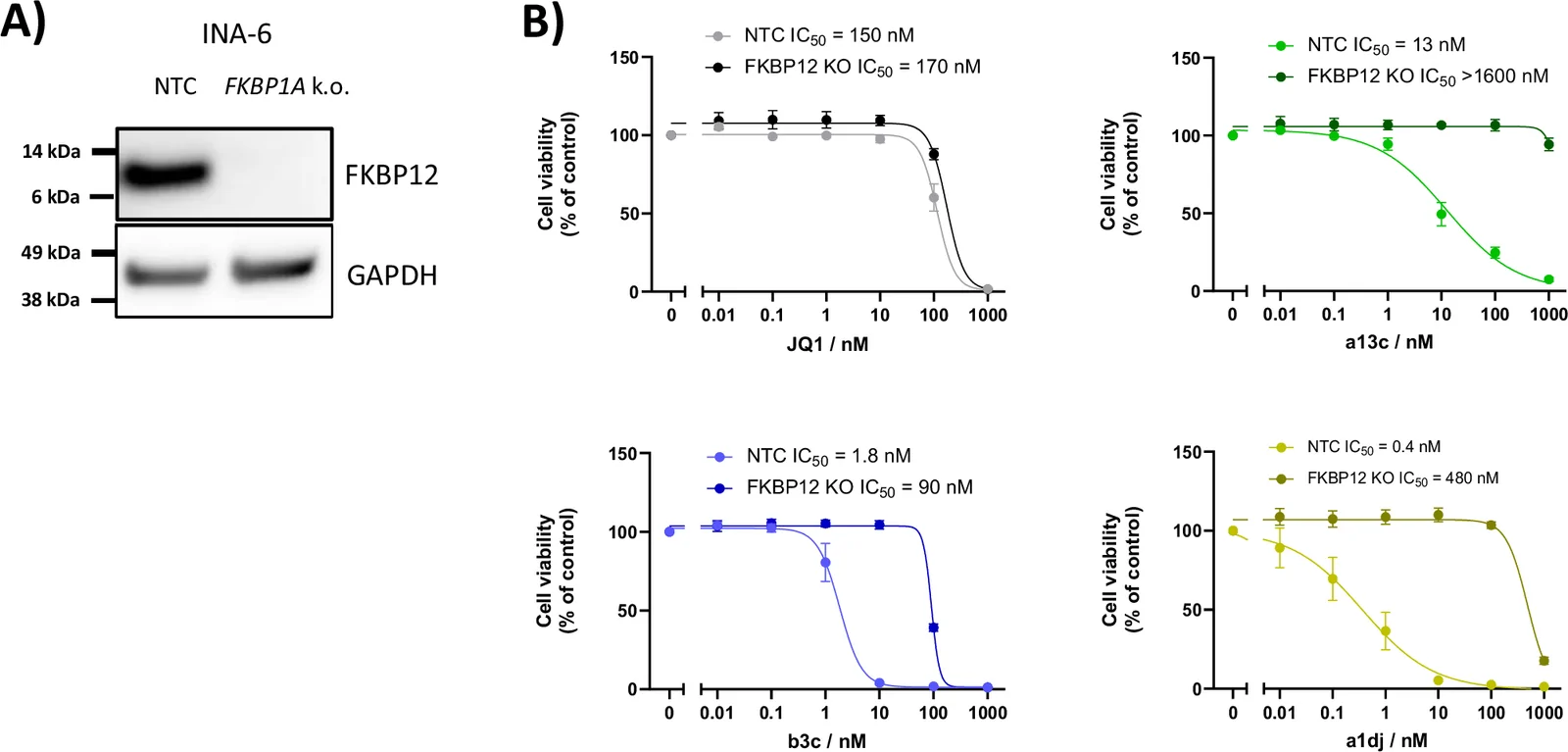
FKBP12-enhanced cell toxicity in myeloma cell lines. **A** Western Blot analysis of INA-6 cells after FKBP12 knockout (FKBP1A gene) or of non-transfected control cells (NTC). **B** Cell toxicity of JQ1 (gray), a13c (green), a1dj (yellow), and b3c (blue) in INA-6 cells after FKBP12 knockout or of nontargeted control (NTC) cells. All experiments were performed in triplicate, means and standard errors are shown for each measurement. Lines indicate dose-dependent reduction of cell viability. IC_50_ values are indicated above each panel.

The observed residual activity of our bifunctional compounds in FKBP12-depleted cells was stronger than predicted from biochemically measured binding to BRD4 alone (see Table 1). To explore if this was due to remaining low levels of FKBP12, we turned to a previously established FKBP12 knock-out in the human myeloma cell line INA-6 (Fig. 6A, see Methods for details). INA-6 cells showed moderate JQ1 sensitivity independent of the FKBP12 status (Fig. 6B). As previously observed for MV4-11 and THP1, FKBP12-BRD4 bifunctionals showed increased efficacies in wild-type cells, reaching sub-nanomolar IC_50_ values for a1dj. In knockout cells, however, cytotoxic effects were hardly observable for a13c and a1dj. Only the strong BRD4 binder b3c induced reduced cell viability at concentrations of 100 nM and higher in FKBP12-negative cells. To further confirm the FKBP12-independent residual activity of b3c, we synthesized a variant of the compound in which FKBP12-binding was dramatically reduced by a ring contraction (Supplementary Fig. 10K, L) and determined its effect on cell survival. Strikingly, the IC_50_ of this compound was three orders of magnitude higher in MV4-11 and THP1 cells compared to b3c, and the remaining activity was unaffected by FKBP12 depletion (Supplementary Figs. 10M, N).

**Fig. 7:**
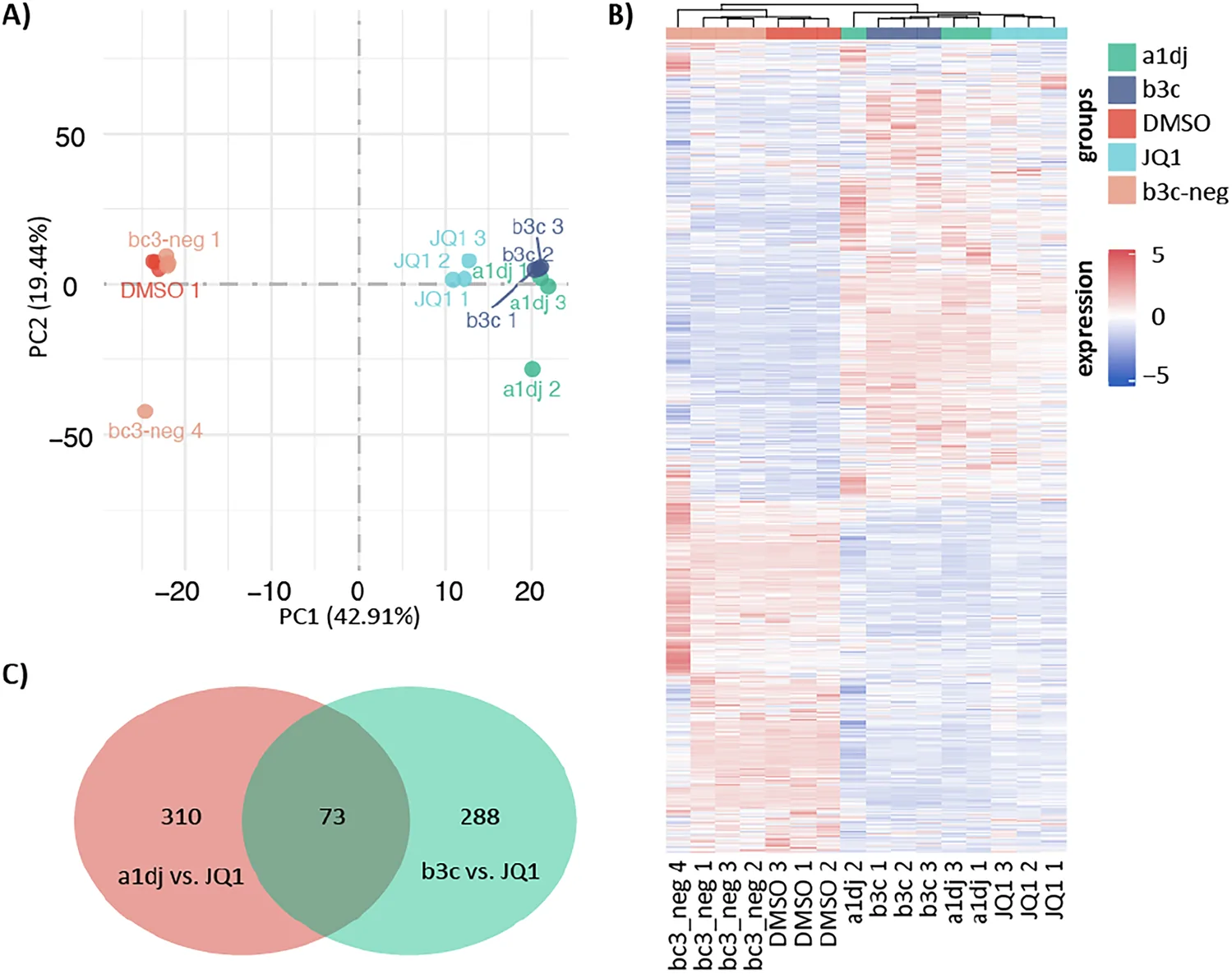
BRD4-targeting compounds elicit comparable changes in gene expression. MV4-11 cells were treated with the indicated compounds for 6 h and subjected to RNA sequencing in triplicate or quadruplicate. **A** Principal component analysis of normalized read counts. Cells treated with BRD4-FKBP12 targeting bifunctionals a1dj and b3c show similar gene expression patterns as JQ1 treated cells and are clearly distinguished from DMSO and the none-FKBP12 targeting analog b3c-neg. **B** Cluster analysis of differentially expressed genes. Each column represents a treatment, and each row a transcript. The heatmap indicates log fold-changes of expression levels scaled across rows, with red indicating higher and blue lower expression. **C** Venn diagram illustrating the number of differentially expressed genes for a1dj and b3c compared with JQ1.

To investigate whether inhibition of BRD4 by FKBP12-BRD4 bifunctionals leads to different remodeling of the transcriptome compared to JQ1, we performed RNA sequencing in MV4-11 cells. Principal component analysis of the resulting data revealed similar effects on gene expression of JQ1, a1dj and b3c, which were clearly separated from cells treated with the non-FKBP12 binding variant b3c-neg or solvent only (Fig. 7A). As expected, BRD4 inhibition induced the differential expression of about 3000 genes, which were largely overlapping between the BRD4-targeting compounds (Supplementary Fig. 11A, B). Clustering differentially expressed genes corroborated the closely related effects of JQ1, a1dj and b3c (Fig. 7B). When gene expression profiles of a1dj and b3c treated cells were compared to JQ1 – treated cells, only about 300 genes were identified as differentially expressed (Fig. 7C). Of note, these genes could not be clearly assigned to distinct biological processes (Supplementary Fig. 11C, D).

**Fig. 8:**
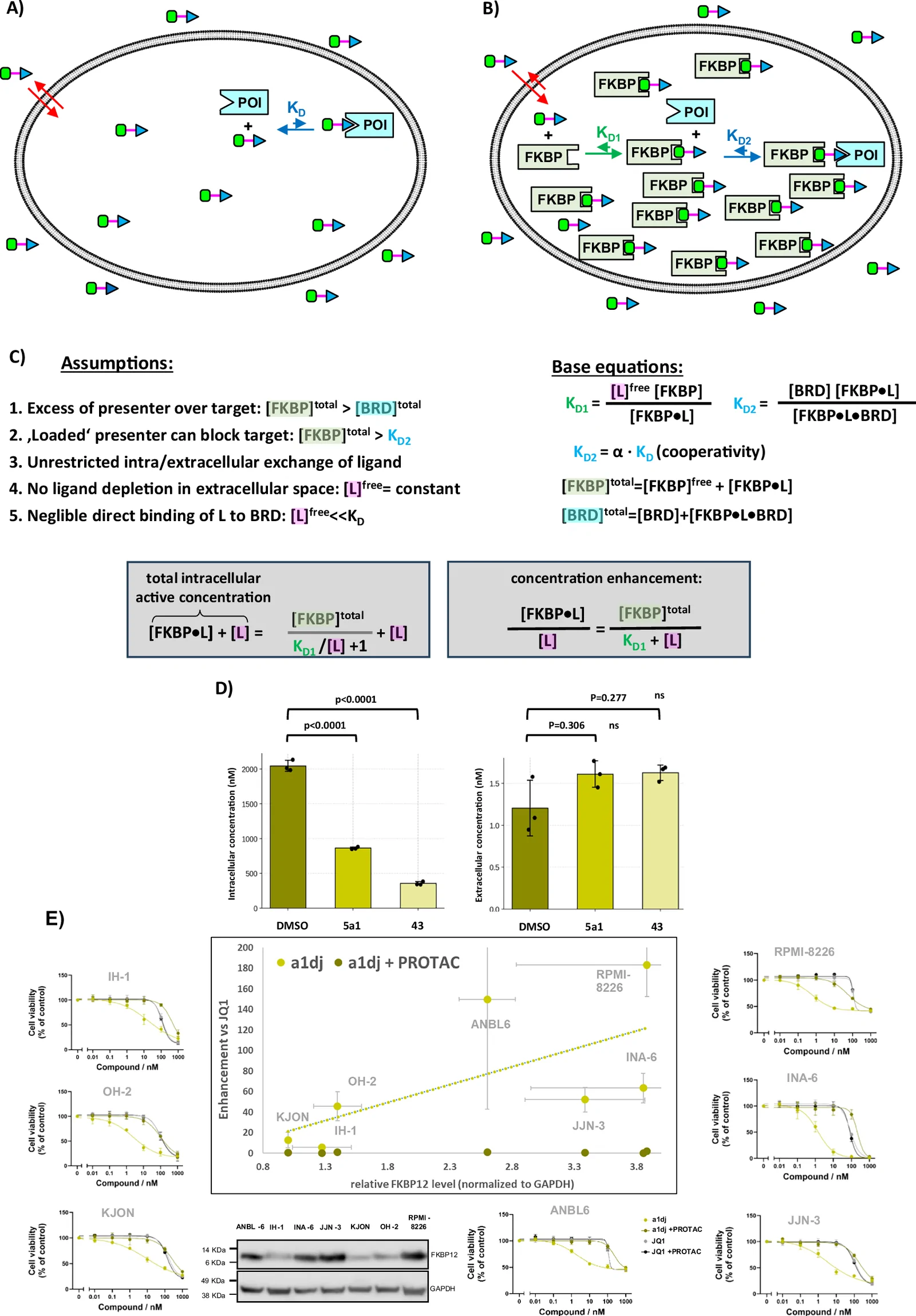
FKBP12-assisted intracellular trapping (CellTrap effect), assuming unrestricted equilibration of unbound drug between extra- and intracellular compartments and neglecting sub-cellular partitioning of the presenter protein. **A** Classical ‘binary’ pharmacology, driven by the affinity to the protein of interest. **B** Presenter protein-assisted pharmacology driven by K_D1_, K_D2_, and the concentration of the presenter protein. Red arrows reflect intracellular drug accumulation due to trapping by intracellular FKBP12. **C** Key numbers and concentration relationships of presenter protein-assisted pharmacology exemplified for FKBP12 and BRD4. L represents the FKBP12-BRD4 bifunctional ligand. **D** Intracellular and extracellular concentrations of a1dj in MV4-11 cells were compared between control cultures and cells pretreated with the FKBP12-degrading PROTAC 5a1 (100 nM, 5 h), followed by addition of 18-(S)Me (10 µM, 10 min) and subsequent co-treatment with a1dj (10 nM, 2 h; total 5a1 exposure: 7 h). Final DMSO concentration was 3% (v/v). FKBP12 degradation by 5a1 reduced intracellular accumulation of a1dj. Competition with the pan-FKBP ligand 18-(S)Me was used to estimate FKBP12-independent (unspecific) cellular accumulation. A consistent increase in extracellular a1dj was observed upon 5a1 treatment across four independent experiments (see Supplementary Fig. 11; pooled Tukey HSD *p* = 0.031 vs DMSO, mean fold change = 1.58). Data represent mean ± SD of *n* = 3 independent biological replicates with individual data points shown. a1dj concentrations were quantified by LC–MS/MS. Statistical analysis was performed using one-way ANOVA with Tukey HSD post hoc correction. One-way ANOVA with Tukey’s HSD post hoc test revealed a significant effect of treatment on intracellular (F(3, 8) = 598.79, *p* < 0.0001) and extracellular concentrations (F(3, 8) = 35.32, *p* < 0.0001). Adjusted *p*-values are shown in the figure. **E** Cell toxicity of a1dj (yellow) in a panel of myeloma cell lines in the presence or absence of FKBP12 PROTAC. Toxicity is substantially enhanced compared to JQ1 (black) in a FKBP12-dependent manner, and the magnitude of enhancement correlates with FKBP12 expression levels (middle graph). FKBP12 and GAPDH levels were quantified from Western Blots (representative experiment shown in the bottom, see also Supplementary Fig. 12A–E). Error bars represent the mean ± standard deviation (SD) from three independent experiments.

Our results so far demonstrate that FKBP12-BRD4 bifunctionals inhibit BRD4 function, leading to potent cell killing in cells depending on the FKBP12 status. Notably, the efficacies of our cooperative protein binders were orders of magnitude higher compared to JQ1. This observation cannot be explained by enhanced biochemical binding, as the FKBP12-assisted biochemical affinities were only 2 - 5-fold higher (Table 1). We hypothesized that the enhanced efficacy in cells is based on an ‘upstream’ binding of bifunctional compounds to the highly abundant protein FKBP12 upon cell entry (Fig. 8A, B). This shifts the equilibrium between extra- and intracellular compound concentrations and thereby increases the effective intracellular concentration. FKBP12 binding, therefore, acts as a cellular trap, which enriches bifunctional compounds in the cells and leads to higher intracellular concentrations compared to monofunctional compounds such as JQ1.

To functionally test this hypothesis, we first developed a mathematical model reflecting intracellular FKBP12 binding and BRD4 inhibition. This model accounted for enhanced cooperative binding of ligands when prebound to FKBP12 (K_D2_ vs K_D_, Fig. 8C) and predicted an increased intracellular concentration of ligand species available for BRD4 inhibition (free ligand + FKBP12-bound ligand), which was positively driven by high FKBP12 levels. In consequence, effective concentrations were predicted to be reachable at very low concentrations, which were dominated by the FKBP12-ligand complex as the active species (Supplementary Fig. 12C). To experimentally test the CellTrap hypothesis, we directly determined cellular concentrations of a1dj in MV4-11 cells. This compound accumulated in the cellular fraction at a mean concentration of 1.68 ± 0.35 µM across four independent experiments (Fig. 8D and Supplementary Fig. 12A, C for approximation parameters^28^), consistent with the estimated intracellular FKBP12 levels in these cells (Supplementary Fig. 12F–H). FKBP12 degradation by the PROTAC 5a1 resulted in a highly consistent reduction of intracellular a1dj accumulation across four independent experiments (mean 51% reduction, range 41–58%; pooled *n* = 11 per group, one-way ANOVA with Tukey HSD *p* < 0.0001), demonstrating that approximately half of the cellular enrichment is FKBP12-mediated. An additional 22% of cellular enrichment was mediated by other FKBPs, which was abolished by competition with a pan-FKBP ligand (18-(S)Me))^19^. The cellular enrichment was not mediated by BRD4 binding, since prior BRD4 depletion did not affect cellular compound levels (Supplementary Fig. 12A). Extracellular a1dj levels showed a modest but significant increase upon treatment by the PROTAC 5a1 (pooled *n* = 12 per group, Tukey HSD *p* = 0.031, mean FC = 1.58 vs DMSO across four experiments; Supplementary Fig. 12B), as expected from reduced intracellular trapping upon FKBP12 degradation. The magnitude of this increase varied between experiments (FC range 0.98–3.75), probably reflecting technical factors including non-specific adsorption of the lipophilic compound to plastic culture surfaces and differences in DMSO concentrations across experiments (2% vs 3% final), rather than biological variability. This interpretation is supported by the high consistency of the intracellular data across all four rounds (Fig. 8D and Supplementary Fig. 12B). To further support the CellTrap concept, we performed a wash-off experiment in MV4-11 cells (Supplementary Fig. 12D, E). The results demonstrate intracellular retention of the bifunctional compound a1dj via FKBP12 binding, as evidenced by persistent MYC repression up to 24 h even after the compound was washed out. To support the CellTrap hypothesis functionally, we determined FKBP12 levels in a panel of human myeloma cells and measured cell survival upon treatment with JQ1 and a1dj (Fig. 8E). Indeed, we observed higher increases of a1dj efficacies compared to JQ1 in cells with higher FKBP12 levels. Importantly, the cytotoxicity of a1dj was almost identical to JQ1 upon depletion of FKBP12 in all cell lines tested (Fig. 8E). Such a correlation between enhanced efficacies and FKBP12 levels were also observed for a13c and, to a lesser extent, for b3c (Supplementary Fig. 13).

### Selective cytotoxicity of a13c in co-cultured prostate-derived cell lines

**Fig. 9:**
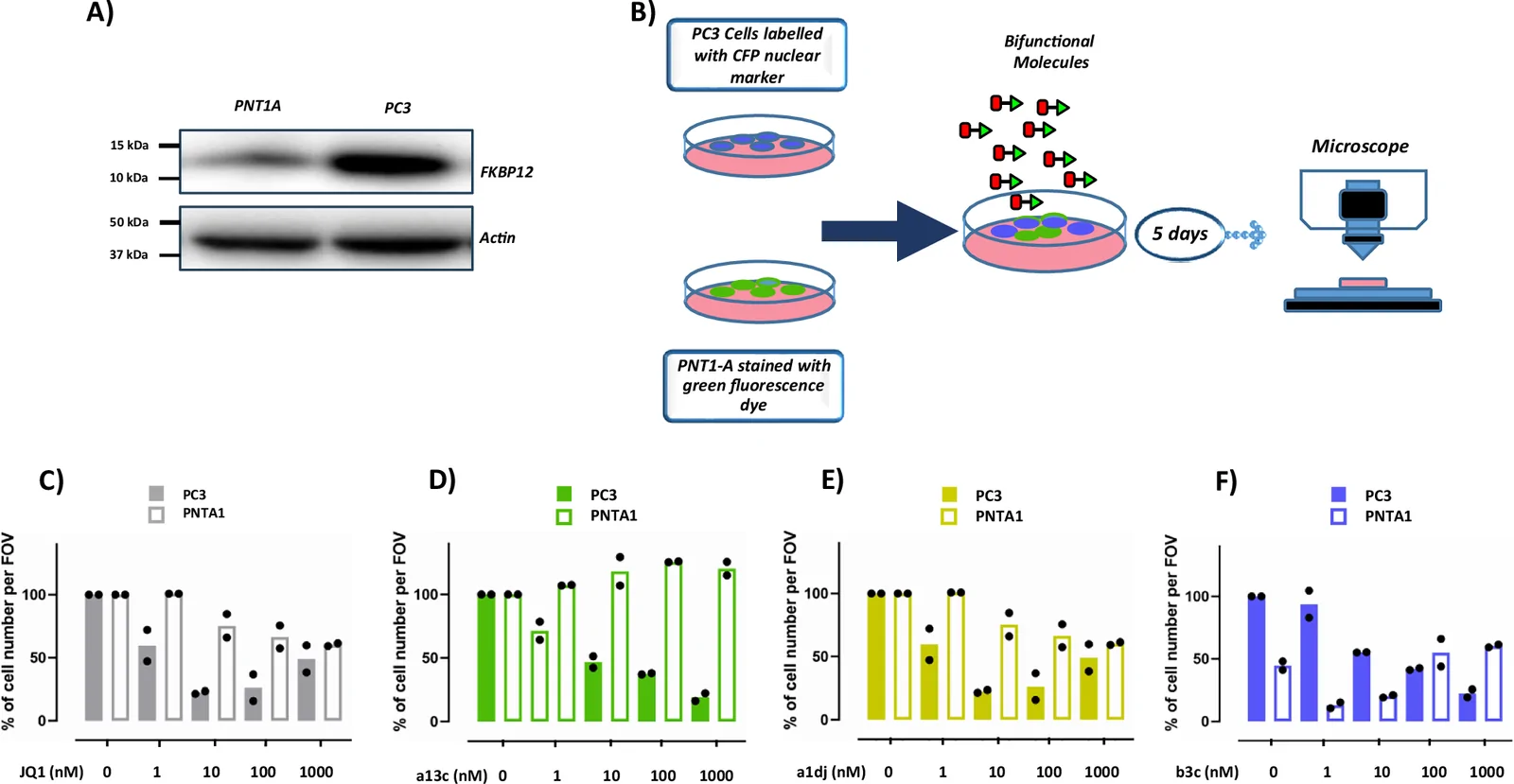
Selective cytotoxicity towards PC3 carcinoma cells over non-transformed PNT1A cells. **A** Western blot analysis showing FKBP12 protein levels in PNT1A and PC3 cell lines. **B** Schematic representation of the co-culture live cell microscopy experiment workflow. **C**–**F** Cell count analysis in PNT1A and PC3 cells following treatment for five days with various concentrations of (**C**) JQ1, (**D**) a13c, (**E**) a1dj, and (**F**) b3c. The data are presented as a result from two independent live cell microscopy experiments.

The cellular trapping of bifunctional compounds and their dependency on FKBP12 levels suggest that they can be employed to preferentially deplete cells with high FKBP12 levels from co-cultures, offering the potential for an unforeseen therapeutic window. To test this hypothesis, we turned to prostate cells where strong differences in FKBP12 levels were observed for nontransformed PNT1A cells compared to PC3 carcinoma cells (Fig. 9A). We first measured cell survival upon treatment with JQ1 and FKBP12-BRD4 bifunctionals. PNT1A cells showed, in general, only modest sensitivity to BRD4 inhibition (Supplementary Fig. 14A–D). As FKBP12 expression was already low in these cells, we observed almost no alteration of cell survival upon FKBP12 depletion. Noticeably, a13c did not affect PNT1A viability even at concentrations of up to 1 µM. In PC3 cells, however, BRD4 inhibition induced strong cytotoxic effects, which were FKBP12 dependent for all tested bifunctionals (Supplementary Fig. 14E–H). As it was recently suggested that the formation of ternary complexes by heterobifunctional compounds could alter the subcellular localization of target proteins^8^, we determined the subcellular location of BRD4 in PC3 cells by immunofluorescence (Supplementary Fig. 14I, J). However, we did not observe any substantial alteration of the ratio between nuclear and cytoplasmic BRD4 levels. To investigate selective cell killing according to FKBP12 levels, we performed co-culture experiments of PNT1A and PC3 cells. We applied differential fluorescent labels to quantify both cell types after treatment with JQ1, a13c, a1dj and b3c (Fig. 9B). For JQ1, we observed preferential killing of carcinoma cells at high concentrations as expected from viability measurements of individual cell types (Fig. 9C, and Supplementary Fig. 14). The concentration range inducing selective cell killing was enlarged for the bifunctionals a1dj and b3c, showing noticeable effects on PC3 cells at 1 to 10 nM (Fig. 9E, F). As PNT1A cells were unaffected by a13c treatment at all concentrations, the most striking effect was observed for this bifunctional; the population of PC3 cells could be efficiently depleted from the co-culture while PNT1A cells continued to proliferate (Fig. 9D). Notably, the selective cytotoxic effect of bifunctionals was reversed upon FKBP12 depletion (Supplementary Fig. 15). This demonstrates that even moderate differences in endogenous presenter protein levels can be used advantageously to induce cell specific toxicity, offering unprecedented opportunities for enhanced therapeutic windows to treating human malignancies.

## Discussion

Drugs that induce proximity between a target protein and an endogenous effector or presenter protein offer numerous unprecedented opportunities for refined pharmacology. Synthetically, proximity-based drugs can be readily implemented by “Lego-like” assembly of heterobifunctional from existing binders of both proteins.

We show for BRD4 and FKBP12 as a target-presenter pair that it was possible to readily obtain heterobifunctional molecules with positive cooperativity. For this, multiple structural solutions exist, three of which we could resolve at an atomic detail. Whether the propensity for positive cooperativity is specific to FKBP12, which has a legacy as a one of nature’s preferred presenter, remains to be seen. The key bifunctionals had reduced BRD4 binding compared to JQ1, although all contained the same BRD4-binding moiety. We previously observed a similar reduction of binding affinity for bifunctionals for FKBP-targeting PROTACs, and uncovered a hydrophobic collapse as a basis for this^24^. The positive cooperativity for our compounds can be retrospectively rationalized based on our ternary complex structures. However, for prospective prediction of cooperativity, many more ternary structures will be necessary, as suggested by recent work on CRBN-based molecular glues^29^.

Our results also show that heterobifunctionals, even in their most basic setup, can readily provide selective ligands that can distinguish between highly similar protein binding pockets, here BRD4^BD1^ vs BRD4^BD2^. This likely is due to the large FKBP12-a13c binary complex, which is predicted to engage an extended surface on BRD4^BD1^, including residues which differ from BRD4^BD2^. Similar effects have repeatedly been observed for PROTACs^30,31^ and are likely a general feature of proximity-based pharmacology. Like for PROTACs, the occurrence of selectivity is hard to predict and must be explored experimentally, underscoring the importance of rapidly exploring linker diversity and linker attachment chemistry. While we discuss the observed cellular effects in the context of BRD4 throughout this manuscript, we cannot exclude that some of the cellular effects might be partially mediated via other target proteins such as BRD2 or BRD3 (Supplementary Table 5).

A striking and, in this magnitude, unexpected finding was the dramatically enhanced potencies of the bifunctionals compared to JQ1. Specifically, the cellular potencies were orders of magnitude higher for the bifunctionals than expected by their biochemical BRD4 affinities. This was strictly dependent on FKBP12 and correlated with the expression level of the presenter protein. We attribute this effect mainly to the intracellular trapping of heterobifunctionals by the highly expressed FKBP12, which we termed ‘CellTrap’ effect (Fig. 8). The potency is further enhanced by the positive cooperativity upon ternary complex formation and possibly could contain other components, such as disruption of PPIs by the tethered FKBP12. Assuming unrestricted equilibration of free ligand, the latter will unidirectionally diffuse into cells, and there bind to FKBP12 until the unbound FKBP12 levels get depleted and approach K_D1_ (Fig. 8B). This follows saturation binding and at [L] = K_D1_, half of the intracellular FKBP12 pool is ligand-bound. The intracellular concentration enhancement scales linearly with presenter protein levels and for [L] « K_D1_ approximately anti-proportionally with the affinity of the presenter protein (Fig. 8C). At these sub-saturating concentrations of the ligand with respect to the presenter protein, the presenter protein is only partially occupied. This is the preferred concentration range for presenter protein-mediated pharmacology, as side effects due to presenter protein blockade are minimized. We assumed an undepletable extracellular reservoir in our mathematical model (Fig. 8C). This was valid in the direct compound uptake assay, which used higher concentrations to allow compound quantification (Fig. 8D). The ‘non-depletion’ assumption might not be valid for highly potent compounds such as a1dj and b3c that are active at picomolar concentrations. However, mathematical modeling of a1dj and b3c at 100pM nominal concentration revealed that sufficient intracellular concentrations of FKBP12-bound compounds could be reached without substantial depletion of the extracellular reservoir.

The key corollary in this model is that the CellTrap effect is most pronounced for highly expressed proteins combined with high-affinity ligands (K_D_ < 5 nM). This can explain the extraordinary cellular potencies observed in this work. Intracellular buffering has been proposed to contribute to the efficacies of the FKBP12-based molecular glues FK506 and Rapamycin^1,32^. Intracellular enrichment was also observed for CypA-based molecular glues^33^ and in drug sequestering strategies^32,34^, but never at this magnitude.

The striking potency enhancement enabled by the CellTrap effect resulted in a large concentration window where the efficacy of the heterobifunctionals were strictly dependent on FKBP12 as a presenter protein. Moreover, FKBP12-high expressing cell lines were clearly more susceptible to FKBP12-BRD4 heterobifunctionals compared to FKBP12-low expression cell lines. This offers an unprecedented layer of cell type specificity, driven by the presenter protein. CellTrap molecules can conceptionally be compared to antibody-drug-conjugates. ADCs achieve cell-type specific cell killing by binding to extracellular antigens, leading to endocytosis and thereby intracellular cytotoxic payload concentrations. CellTrap molecules bind to intracellular presenter proteins, which allows to achieve cytosolic concentrations sufficient to induce cell killing. We demonstrated the preferential killing of PC3 cells over non-transformed PNT1A cells in co-culture experiments. This is conceptually similar to the RIPTAC concept^6^, which, however, has so far only been shown with overexpressed presenter proteins. Our results demonstrate that even moderate differences in presenter protein expression can be used to achieve cell-type specificity.

Taken together, our findings show that remarkable cellular potencies and presenter protein dependencies can be achieved with heterobifunctionals containing high-affinity ligands for high-expressing proteins. We postulate that this may compensate for unfavorable physicochemical properties imparted by the high molecular weight. Indeed, their limited baseline activity in the absence of the CellTrap effect contributes to the observed cell-type specificity, potentially creating favorable therapeutic windows. Furthermore, like other bifunctional modalities, the co-engagement of two proteins enables otherwise difficult-to achieve selectivity and allows to differentiate cells based on the co-engaged presenter protein. Compared to PROTACs, non-degrading heterobifunctionals might be the preferred choice when degradation of the entire target proteins is difficult, e.g., for poorly degradable target proteins, or not desirable, e.g., due to excessive toxicity. Although not observed in this work, we note that engaging the target with a presenter-compound pre-complex might be functionally different than blocking the target with a small molecule alone. It is also important to keep in mind that in cells, two FKBP12-heterobifunctional complexes may be able to bind simultaneously to the BD1 and BD2 domains of full-length BRD4. We expect our findings to be similarly relevant to molecular glues in addition to heterobifunctional molecules. Our findings support CellTrap-assisted heterobifunctionals as therapeutically attractive options, underscore the potential of highly expressed proteins as presenters, and call for developing high-affinity ligands for them.

## Methods

### Fluorescence polarization assays

Binding of compounds to FKBP12 and to the isolated BD1 and BD2 domains of BRD2, BRD3 and BRD4 was determined in a competitive fluorescence polarization assay. For FKBP12, the assay was performed as described by Kozany et al. using the tracer developed by Pomplun et al.^22,25^. In brief, a serial dilution of the compounds in DMSO was prepared using a Biomek FX^P^ pipetting robot (Beckman Colter). The compounds were transferred to black 384-well plates (Greiner 781076) filled with assay buffer (20 mM HEPES-NaOH, pH 8.0, 150 mM NaCl, 0.002% TX-100) and the tracer (final concentration 0.5 nM) as well as FKBP12 (final concentration 1 nM) were added. After equilibration of 30 min at room temperature, the assay plates were read using a Tecan Spark plate reader using excitation/emission wavelengths of 535/590 nm. A similar assay routine was developed for BRD2, BRD3 and BRD4. In brief, a serial dilution of the compounds in DMSO was prepared using a Biomek FX^P^ pipetting robot (Beckman Colter). The compounds were transferred to black 384-well plates (Greiner 781076) filled with assay buffer (20 mM HEPES-NaOH, pH 8.0, 150 mM NaCl, 0.002% TX-100 and 0.05% BSA) or assay buffer supplemented with FKBP12 or FKBP51FK1 (final concentration 15 µM) and the tracer JQ1-FL (final concentration 2 nM) as well as the respective BD1 or BD2 domain of BRD2, BRD3 or BRD4 (final concentration 200 nM) were added. After equilibration of 30 min at room temperature, the assay plates were read using a Tecan Spark plate reader using excitation/emission wavelengths of 485/535 nm. Data analysis was performed with GraphPad Prism 8 software using the fitting equation developed by Wang^35^.

### HTRF assays

Compound induced ternary complex formation was followed by an HTRF assay. This assay relies on the establishment of an HTRF signal between a terbium-labeled antibody binding to the His_6_-tag of the respective BD1 or BD2 domain of BRD4 and EGFP-labeled FKBP12 upon close proximity. Therefore, a serial dilution of the respective compound in EGFP-FKBP12 (final concentration 5 µM) containing assay buffer (20 mM HEPES-NaOH, pH 8.0, 150 mM NaCl, 0.002% TX-100 and 0.05% BSA) was placed in a white 384-well assays plate (ProxiPlate 384 shallow well Plus, Revvity) and a mixture of BRD4^BD1^ or BRD4^BD2^ (final concentration 100 nM) and 2-fold terbium-labeled anti-6xHis antibody (Revvity) was added. After equilibration of 30 min at room temperature, the fluorescence intensity at 535 and 620 nm upon excitation at 340 nm was determined on a Tecan Spark plate reader using a lag time of 150 µs and an integration time of 500 µs. The HTRF signal was determined by forming the ratio 535 nm/620 nM, and the data was fitted to a binary binding model using GraphPad Prism 8 software.

### Protein crystallographic structure determination

In order to generate the FKBP12:**a1d**:BRD4^BD1^, FKBP12:**a1d**:BRD4^BD2^ and the FKBP12:**b3c**:BRD4^BD1^ complexes for protein crystallization trials, 2 ml of a solution containing 140 µM BRD4^BD1^ or 140 µM BRD4^BD2^, 140 µM **a1d** or **b3c** and 150 µM FKBP12 were prepared in 20 mM HEPES, pH 7.5, 150 mM NaCl, 5% DMSO and concentrated to about 400 µl. The samples were separated on a size exclusion column (Superdex 75 10/100 GL increase, Cytiva) and fractions containing the ternary complex were pooled, concentrated to about 10 mg/ml, flash frozen in liquid nitrogen and stored at − 80 °C.

The crystallization of the FKBP12:**a1d**:BRD4^BD1^ complex was performed at room temperature using the hanging drop vapor-diffusion method, equilibrating mixtures of 1 µl protein complex and 1 µl reservoir against 500 µl reservoir solution consisting of 22% PEG3350, 0.2 M NaCl and 0.1 M Tris-HCl, pH 8.5. Crystals were fished, cryo-protected with reservoir solution supplemented with 10% glycol and flash frozen in liquid nitrogen.

The crystallization of the FKBP12:**a1d**:BRD4^BD2^ complex was performed at room temperature using the hanging drop vapor-diffusion method, equilibrating mixtures of 1 µl protein complex and 1 µl reservoir against 500 µl reservoir solution consisting of 18% PEG3350, 0.2 M NH_4_SCN, 0.1 M MES pH 6.5 and 5 mM Anderson−Evans polyoxotungstate. Crystals were fished, cryo-protected with reservoir solution supplemented with 10% glycol and flash frozen in liquid nitrogen.

The crystallization of the FKBP12:**b3c**:BRD4^BD1^ complex was performed at room temperature using the sitting drop vapor-diffusion method, equilibrating mixtures of 0.5 µl protein complex and 0.5 µl reservoir against 30 µl reservoir solution consisting of 25% PEG3350, 0.2 M NH_4_SCN and 0.1 M Tris-HCl, pH 8.8. Crystals were fished, cryo-protected with reservoir solution supplemented with 10% glycol and flash frozen in liquid nitrogen.

The crystallographic experiments were performed at the BL14.1 at the Helmholtz-Zentrum BESSYII synchrotron, Berlin, Germany^36^ and the beamline ID23-1 of the European Synchrotron Radiation Facility (ESRF) in Grenoble, France, and the raw data can be accessed at https://doi.org/10.15151/ESRF-DC-2127908021^37^. Detailed information on the structure solution and the crystallographic data are provided in the Supplementary Information.

### Cell lines

PC3 cells were kindly provided by Dr. G. Scheiner-Bobis (Justus Liebig University Giessen, Germany), THP1 and MV4-11 acute myeloid leukemia cells by Dr. S. Knapp (Goethe University Frankfurt, Germany). PNT1A prostate-derived normal epithelial cells, and RPMI8226 HMCL were purchased from ATCC. HEK293T and PC3 cells were cultured in Dulbecco’s Modified Eagle Medium (DMEM; Gibco, Darmstadt, Germany) supplemented with 10% fetal bovine serum (FBS) and 1% penicillin-streptomycin (P/S). PNT1A, THP1 and MV4-11 cells were maintained in RPMI−1640 medium (Gibco). THP1 cells were supplemented with 15% FBS, while MV4-11 and PNT1A cells were grown in 10% FBS. Media included 1% P/S (Merck, Darmstadt, Germany) and 1% GlutaMAX (Gibco).

The human myeloma cell lines (HMCL) ANBL-6 (kind gift from Dr. D. Jelinek, Mayo Clinic, Rochester, MN, USA), INA-6 (kind gift from Dr. M. Gramatzki, University of Erlangen-Nurnberg, Germany), and JJN-3 (kind gift from Dr. J. Ball, University of Birmingham, UK) were grown in 10% FBS with 1 ng/mL interleukin (IL)-6 (Gibco, Thermo Fisher Scientific, Waltham, MA, USA) added for ANBL-6 and INA-6. RPMI-8226 was grown in 15% FBS in RPMI. IH-1, KJON, and OH-2 were established in our laboratory^38^ and were grown in 10% human serum (HS, Department of Immunology and Transfusion Medicine, St. Olav’s University hospital, Trondheim, Norway) in RPMI, supplemented with IL6 (2 ng/mL). For experiments, 2% HS in RPMI was used, with IL-6 (1 ng/mL) added for all cell lines, except JJN-3 and RPMI-8226.

### NanoBRET

The NanoBRET assay was performed as described previously^26,39^. To study BRD4 engagement, we employed two models: cells stably expressing BRD4-NanoLuc and cells with transient BRD4-NanoLuc expression. For stable cell lines, the coding sequence of human BRD4 (Isoform B) was fused in-frame to NanoLuc luciferase under the control of the human ubiquitin C (UbC) promoter, with left and right homology arms targeting the AAVS1 locus in intron 1 of the PPP1R12C gene to facilitate genomic integration. PC3 prostate cancer cells were transfected with this construct and vectors expressing Cas9 and an sgRNA targeting the AAVS1 locus using Lipofectamine 3000 (Invitrogen). Successful integrands were selected with blasticidin. Expression of the fusion protein was confirmed by western blot. For transient experiments, full-length BRD4 fused to NanoLuc at N terminus, was cloned into an expression plasmid and transfected into HEK293T cells using FuGENE HD (Promega, E2312), with protein expression allowed for 20 h. Serially diluted inhibitors and BRD Tracer-02 (Promega, TracerDB ID: T000022) at the Tracer KD concentration (from TracerDB)^40^ were dispensed into white 384-well plates (Greiner 781207) using an ECHO 550 acoustic dispenser (Labcyte). The cells were trypsinized, resuspended in Opti-MEM without phenol red (Life Technologies), and seeded at 2 × 10^5 cells/mL. After equilibration for 2 h at 37 °C/5% CO₂ following compound treatment, the NanoBRET® NanoGlo Substrate + Extracellular NanoLuc® Inhibitor (Promega, N2162) was added according to the manufacturer’s protocol, and filtered luminescence was measured on a PHERAstar plate reader (BMG Labtech) using a donor (450 nm BP) and acceptor (610 nm LP) filter pair. Competitive displacement data were analyzed using GraphPad Prism 9 with a normalized 3-parameter curve fit: Y = 100/(1 + 10^(X − log IC₅₀)). For BRET measurements after FKBP12 knockdown in stably expressing BRD4 Nano-Luc reporter cell line, 10 nL/per well of the FKBP12 PROTAC was pipetted to each well using Labcyte to achieve the final concentration of 200 nM and the plated cells were incubated over-night at 37 °C and 5% CO_2_. BRET measurements were performed as outlined above.

### Western blot and quantitative Western Blot

To assess FKBP12 levels, 5 × 10⁵ PC3, PNT1A, and HMCL cells were seeded into 6-cm plates and cultured for two days. Suspension cell lines MV4-11 and THP1 were cultured in T25 flasks under similar conditions. For FKBP12 degradation, all cell lines were treated with an FKBP12-specific PROTAC (5a1) 200 nM for either 6 hours or 4 h in the case of HMCL. Following treatment, cells were washed with PBS, harvested, and lysed for 30 min on ice. The lysis buffer used contained 1% IGEPAL CA-630 (Sigma Aldrich, St Louis, MO, USA), 150 mM NaCl, 50 mM Tris–HCl (pH 7.5), protease inhibitor cocktail Complete Mini (Roche, Basel, Switzerland), 50 mM NaF, and 1 mM Na_3_VO_4._ Protein concentrations were determined using the Bradford assay. 20 µg protein was loaded on 10% SDS-PAGE gels alongside a pre-stained molecular weight marker (Bio-Rad, Feldkirchen, Germany). Proteins were transferred to either nitrocellulose or PVDF membranes (Merck, Darmstadt, Germany) using a wet transfer system at 100 V for 1.5 h. Membranes were blocked with 5% skim milk in TBS with 0.1% Tween-20 for 1 h at room temperature and subsequently incubated overnight at 4 °C with primary antibodies. Primary antibodies used were FKBP12 (RRID: AB_2102847, #sc133067, Santa Cruz, TX, USA), FKBP12 (HUABIO: HBO-ET1703-55, BIOZOL, Eching, Germany), Actin (SIGMA-ALDRICH, A5316, Germany), and GAPDH (RRID: AB_2107448, Cat# Ab8245, Abcam, Cambridge, UK). Membranes were washed and incubated with HRP-conjugated secondary antibodies for 1 h at room temperature. Protein bands were detected using an ECL kit and visualized with either Fusion FX gel imaging system or ChemiDoc Imager (Bio-Rad) and quantified using Image Studio software (LI-COR Biosciences, Cambridge, UK). For quantitative western blotting, cell lysates were prepared from a defined number of cells. Recombinant purified FKBP12 protein provided by the Hausch group was used as a reference. Lysates were run on the gel together with the purified FKBP12 protein according to the procedure described above. FKBP12 was detected using a fluorescently-labeled secondary antibody, and band intensities were quantified on an Odyssey Fc Dual-Mode Imaging System (LI-COR Biosciences, Cambridge, UK). These intensities were used to generate a standard curve from the purified protein, which allowed calculation of the amount of FKBP12 loaded for each cell line. To estimate the FKBP12 concentration per cell, cell volumes were measured by staining with the cytoplasmic dye Cytopainter green (Abcam) and imaging trypsinized cells by microscopy. The diameter of at least 100 cells per cell line was measured using ImageJ and used to calculate the average cell volume. Finally, the FKBP12 concentration per cell was estimated mathematically.

### Quantitative RT-PCR

Total RNA was extracted using the Monarch Total RNA Isolation Kit. RNA concentration and purity were assessed using a NanoDrop spectrophotometer. cDNA synthesis was performed using reverse transcription. Quantitative PCR (qPCR) was conducted on an Applied Biosystems StepOne Real-Time PCR system using the following primers: MYC forward: TTGGTGAAGCTAACGTTGAGG; MYC reverse: TATTCTGCCCATTTGGGGACA, Actin forward: TAGAAGCATTTGCGGTGGACGATG; Actin reverse:

GGCACCCAGCACAATGAAGATCAA-

### Cell viability assay

Cell viability and cytotoxicity were assessed using the CellTiter-Glo Luminescent Viability Assay. HMCL, PC3, and PNT1A cells were seeded into 96-well plates at densities of 1 × 10^4^_,_ 1 × 10^3^_,_ and 4 × 10^3^ cells per well, respectively. For FKBP12 degradation experiments, the cells were pre-treated with an FKBP12-specific PROTAC (5a1) 200 nM for either 6 hours or 4 h in the case of HMCL, before adding the compounds or corresponding amount of DMSO, each in a total volume of 100 µl full growth medium. The plates were incubated for either 5 consecutive days or two days for HMCL cells. Suspension cell lines MV4-11 and THP1 were cultured in T25 flasks for 2 days prior to the experiment. FKBP12 degradation was induced following the same procedure described for adherent cells. After incubation, the cells were counted, and 4 × 10³ cells were seeded per well in 96-well plates. Experimental treatments, including the appropriate concentrations of various compounds and necessary controls, were added to each well in 100 µl full growth medium. After the incubation period, the full growth medium containing the compounds was aspirated from all cell lines. The CellTiter-Glo 2.0 reagent (Promega, Madison, WI, USA) was diluted 1:1 with PBS and added to each well. For MV4-11 and THP1 suspension cells, the reagent was instead diluted 1:1 with the existing full growth medium in each well. Luminescence was measured using a TECAN microplate reader (TECAN, Switzerland).

### CRISPR/Cas9 knock-out of *FKBP1A*

INA-6 FKBP1A KO cells were made previously^41^. In brief, CRISPR sgRNAs were designed using Benchling (https://www.benchling.com/). Oligo pairs encoding sgRNA targeting *FKBP1A* (GGGCGCACCTTCCCCAAGCG) or non-targeting control (TGAATCGTAACCTCGCCATT) were annealed and ligated into lenti-CRISPR v2 (gift from Feng Zhang, Addgene plasmid #52961; http://n2t.net/addgene:52961;RRID: Addgene_52961). The plasmids were cotransfected with third-generation lentiviral packaging plasmids using Genejuice (Novagen, Merck Life Science AS, Oslo, Norway) in 293 T packaging cells (Open Biosystems, Thermo Fisher Scientific). The filtered supernatant was used to generate the knockout cells by treating INA-6 with lentiviral particles in the presence of polybrene (8 µg/mL). Fresh medium was added after 4 h. Puromycin (0.5 µg/mL) was added after 48 h to select for cells with successful integration. Loss of *FKBP1A* was confirmed with immunoblotting, and the cells were expanded and used for experiments.

### Live cell microscopy and immunofluorescence staining

For live cell microscopy, PC3 prostate cancer cells and PNT1A normal prostate epithelial cells were co-cultured and analyzed. PC3 cells were labeled with a CFP fluorescent nuclear marker, while PNT1A cells were stained with Cytopainter Green dye. Equal numbers of both cell types were seeded into 24-well plates under the specified experimental conditions. After 5 days of incubation, the culture medium was replaced with microscopy medium to optimize imaging conditions. Images were acquired using live-cell microscopy at 20x magnification. Cell segmentation and quantification were performed using a combination of CELLPOSE and custom MATLAB scripts. For FKBP12 staining, PC3 cells (3 × 10⁵ cells) were seeded onto the poly-L-lysine–coated coverslips and allowed to adhere for two days. The PC3 cells were then treated with the bifunctional compound a1d for 6 h. Following treatment, the cells were washed three times with PBS and fixed using 5% formalin. After fixation, the cells were permeabilized with 0.1% Triton X-100 in PBS and blocked with 1% bovine serum Albumin (BSA) in 0.1% Triton X-100. After the blocking, the cells were incubated overnight with rabbit monoclonal FKBP12 primary antibody (HBO-ET1703-55, BIOZOL) in a humidified chamber. The following day, the cells were washed 3 times with PBS and incubated with Alexa Fluor 488 goat anti-rabbit secondary antibody (A-11034, Invitrogen) diluted in blocking solution for 1 h at room temperature. The cells were then washed three times with PBS, and nuclei were stained with Hoechst (H3570, Invitrogen) for 10 min at room temperature. The prepared slides were imaged using a NIKON ECLIPSE TI immunofluorescence microscope at 60× magnification

### Cell uptake assay

MV4-11 cells were resuspended and cultured in medium without FBS at a density of 2.5 × 10⁶ cells/mL, and 400 µL of the cell suspension was seeded into each well of a 48-well plate. 100 nM PROTAC (5a1, 43^42^, 5a1 + 43 or DMSO; 1% final) was added, and the plate incubated for 5 h at 37 °C. 10 µM 18^(S)Me^ (or DMSO; 2% total) was added to the culture 10 min before addition of 10 nM **a1dj** (3% DMSO final in total) and incubated for 2 h. Cells were harvested by centrifugation at 300 g for 5 min and the culture medium reserved and frozen at − 80 °C. Cells pellets were washed with 200 µL PBS and harvested by centrifugation. Wash supernatant was discarded, and cell pellets frozen at − 80 °C. All treatment conditions were performed in triplicate with an additional control well per condition containing no cells.

For the quantification of **a1dj**, cell culture medium samples were thawed at 37 °C and 850 rpm for 30 min, and centrifuged at 15,000 × *g* for 5 min. 45 µL sample was mixed with 45 µL acetonitrile (ACN), 5 µL of an internal standard (IS) solution (**a1d** at 0.12 µM in methanol), 80 µL methanol and 5 µL formic acid (FA) 10%. A calibration series of **a1dj** ranging from 0.32 to 160 nM was prepared in ACN and treated like samples (addition of 45 µL cell culture medium for matrix match). The mixtures were thoroughly vortexed and centrifuged at 4000 × *g* for 5 min. 80 µL of the supernatant were mixed with 30 µL water, and 20 µL of the mixture injected on the LC/MS system. Cell pellets were first resuspended in 100 µL 50 mM HEPES pH7.4 then lysed by repeated freeze/thaw cycles (*n* = 4) in liquid N_2_ and at 37 °C. Cell lysates were centrifuged at 4000 × *g* for 90 min and 30 µL supernatant mixed with 30 µL ACN, 35 µL ACN + 0.5% FA, and 5 µL IS solution. A calibration series of **a1dj** ranging from 0.1 to 200 nM in ACN was prepared and treated as a sample (addition of HEPES buffer for matrix match). The mixtures were thoroughly vortexed and centrifuged at 4000 × *g* for 5 min. 8 µL of the supernatant were injected on the LC/MS system. Both types of samples were injected on a 1290 Infinity II UHPLC system (Agilent) equipped with Aquity UPLC BEH C18 1.7 µm 2.1 x 50 mm (Waters) column operated at 1 mL/min. The liquid chromatography method consists in an initial phase at 5% B for 0.2 min, followed by a gradient to 90% B over 0.7 min and a plateau at 90% B for an additional 0.2 min. The column is then re-equilibrated at 5% B over 0.2 min. Eluent A consists of water with 0.1% FA and eluent B of ACN with 0.1% FA. For detection, the UHPLC was coupled to a mass spectrometer API 6500 + Triple quadrupole (AB Sciex) via an ESI source. Detection of **a1dj** and of the internal standard, **a1d**, was achieved using MRM in the positive mode using 2 precursor/product pairs per molecule. **a1dj** was monitored via the m/z pairs 524.77/383.1 and 524.77/341.0, and **a1d** with 517.76/383.0 and 517.76/341.0. Data analysis was performed with Skyline 25.1. Intracellular concentrations were calculated using the measured average number of viable cells (0.822 × 10^6^) and cell diameter (13.7 µm), and assuming cells are empty sphere (nucleus and organelles ignored).

### Global proteomics results

Cell pellets equivalent to those used in the cell uptake assay were resuspended in 220 µL of 50 mM Tris pH 8.0, 2% SDS for 30 min at 850 rpm at RT, and centrifuged at 15000 g for 15 min. A total of 32 samples, comprising four biological replicates for the 8 groups, were processed (pretreatment with DMSO, PROTACs alone or in combination, followed by addition of DMSO or 10 nM a1dj: see above for details). The supernatant was reduced with 5 mM DTT at 37 °C for 30 min then alkylated with 15 mM iodoacetamide at room temperature for 30 min in the dark. 150 µl of the solutions were subjected to a Protein Aggregation Capture digestion protocol using amine magnetic beads (MagReSyn) and a King Fischer instrument (Thermo Scientific). In short, soluble proteins are captured on the magnetic beads and washed prior to the addition of 1/50 (w/w) trypsin (Promega) for an overnight digestion at 37 °C and 850 rpm. On the consecutive day, digested peptides are released, and the peptide concentration of the supernatants determined using a fluorimetric assay (Pierce). 200 ng peptide was loaded on Evotips (Evosep Biosystems). Peptides were separated on an Evosep One LC system coupled to a timsTOF Pro 2 instrument (Bruker) using a 30-sample-per-day method (44 min gradient). Peptides were resolved on an 8 cm, 1.5 µm x 150 µm Performance column (Evosep Biosystems). DIA data were acquired and analyzed using DIA-NN 1.8.1 with the following settings: maximum mass accuracy tolerances set to 20 ppm (MS1 and MS2 spectra), enzyme: trypsin, fixed modification: carbamidomethylation at cysteine; number of allowed missed cleavage sites: 1; min and max peptide length: 7 - 30. The relaxed-prot-inf option was used for library-free processing of the data. Data were searched against UniProt Homo sapiens (entries: 20,365, release date: 05/2020) in combination with the common repository of adventitious proteins (cRAP) database containing common contaminants. Match between runs (MBR) was enabled, and the normalization option switched on. The software output was filtered at precursor *q*-value < 1%, global protein *q*-value < 1%.

### RNAseq

MV4-11 cells were treated for 6 hours with JQ1 (500 nM), a1dj (10 nM), b3c (1 nM), b3c-neg (1 nM), or DMSO prior to RNA isolation. RNA was isolated using the Monarch Total RNA Isolation Kit. RNA quality was assessed using an Agilent TapeStation. All samples had calculated RINe values > 6 with a flat baseline. Library preparation and RNA sequencing experiments were performed in biological triplicates or quadruplicates (b3c-neg) by Novogene using poly-A-enrichment and paired-end-sequencing of 150 bp. Samples were demultiplexed, and FastQ files were generated. Raw FASTQ reads were quality-filtered using fastp v1.3.2 to remove adapters, low-quality bases (Phred ≤ 5 in > 50% of read) and poly-N regions (> 10% N bases), retaining reads with Q20 ≥ 95% and Q30 ≥ 90%. GC content was calculated for clean reads. Filtered sequence reads were aligned to the human reference genome GRCh38 using HISAT2 v2.2.1, guided by GTF annotation. Mapped data were summarized on the gene level as Fragments Per Kilobase of transcript sequence per Millions base pairs sequenced (FPKM). Gene counts were quantified using featureCounts v2.0.6. Further data evaluation, normalization and pairwise differential expression analyses was done by DESeq2. For clustering, all differentially expressed genes were pooled as the differential gene set. FPKM values of genes were hierarchically clustered, rows were homogenized as Z-scores. For enrichment analysis of the differential expressed genes, cluster Profiler (Yu G, 2012) software was used on Gene Ontology (http://www.geneontology.org/) terms reflecting biological processes.

### Reporting summary

Further information on research design is available in the Nature Portfolio Reporting Summary linked to this article.

## Supplementary information

Supplementary Information

Reporting Summary

Transparent Peer Review file

## Source data

Source Data

## Data Availability

The sequencing data are publicly available at the Gene Expression Omnibus (GEO) under the accession number GSE317727. The mass spectrometry proteomics data have been deposited in the ProteomeXchange Consortium via the PRIDE^43^ partner repository with the dataset identifier PXD081130. Coordinates for the obtained crystal structures were submitted to the PDB under 9QW8 https://doi.org/10.2210/pdb9QW8/pdb, 9R5N https://doi.org/10.2210/pdb9R5N/pdb and 29QL https://doi.org/10.2210/pdb29QL/pdb. The raw data for PDB-ID 9QW8 can be accessed under https://doi.org/10.15151/ESRF-DC-2127908021. The source data underlying the figures, graphs, and uncropped blots/gels presented in the main figures and Supplementary Information are provided as a Source Data file with this paper. Source data are provided in this paper.
