# Supplementary material for: Cell type–selective targeting by heterobifunctional protein binders via in-cell enrichment
Source: Nat Commun. 2026 Sep 17;17:9929. doi: 10.1038/s41467-026-77460-w (PMC13586308; doi:10.1038/s41467-026-77460-w)
Supplement: Supplementary file 1 [file 41467_2026_77460_MOESM1_ESM.pdf]

1. Supplementary Figures

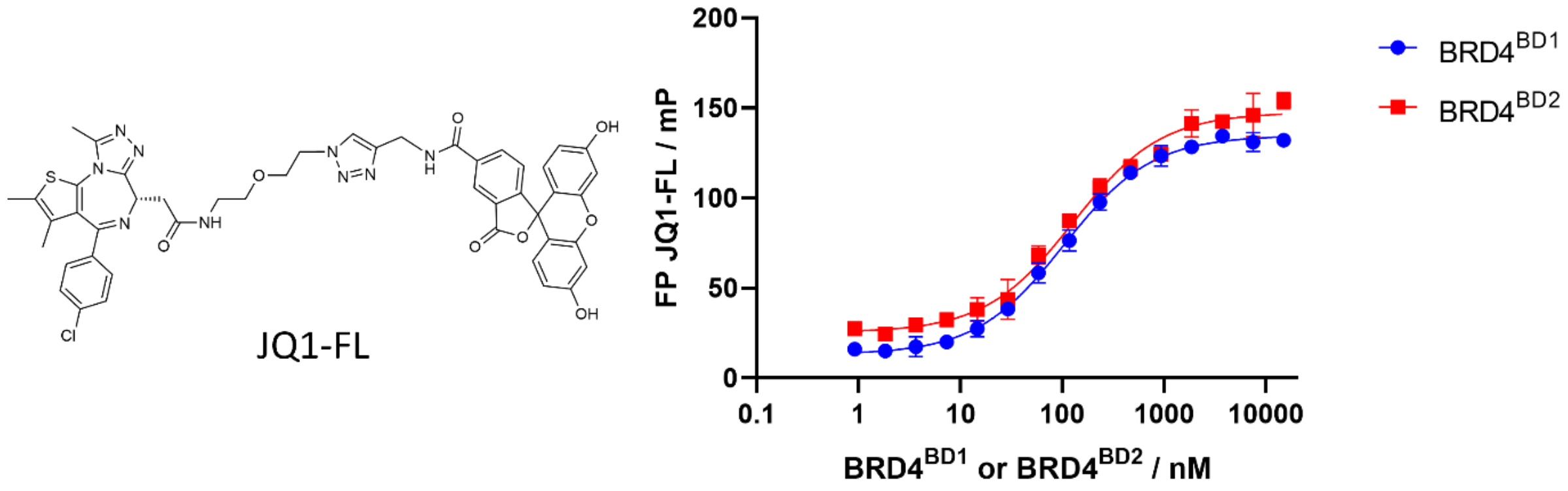

Supplementary Fig. 1 Characteristics of the fluorescence polarization assay for BRD4.
The binding of BRD4^{BD1} and BRD4^{BD2} to JQ1-FL was determined with a fluorescence polarization (FP) assay. Therefore, a serial dilution of BRD4^{BD1} and BRD4^{BD2} was added to 2 nM of JQ1-FL and the fluorescence polarization was determined. Data ponits are indicated as mean from three replicates and standard deviations are indicated as error bars. The solid lines represent data fitted to a binary binding model. The K_d-values were determined to be 100±6 nM for BRD4^{BD1} and 130±10 nM for BRD4^{BD2}.

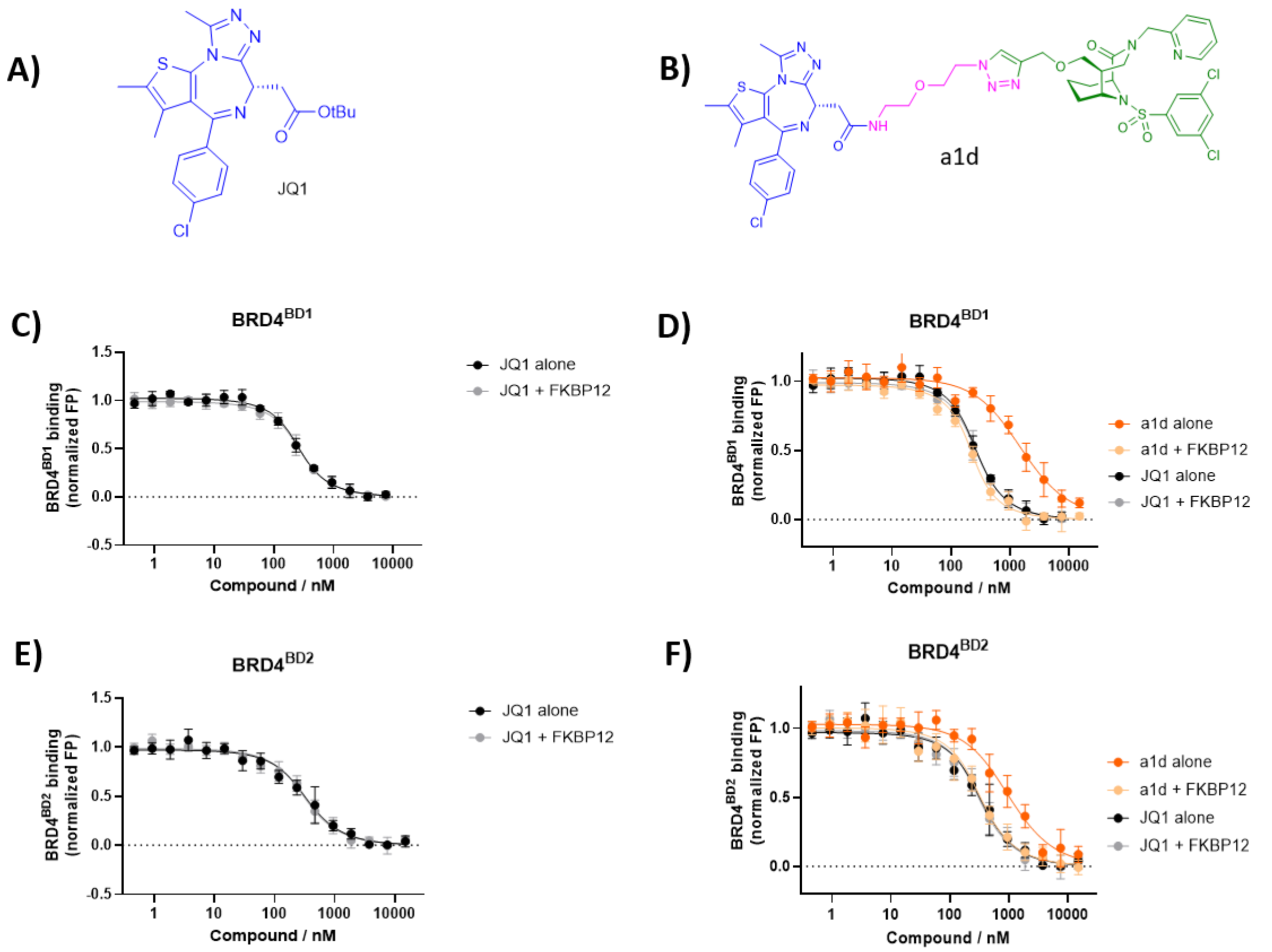

Supplementary Fig. 2 BRD4 binding analysis of a1d.

(A–B) Chemical structures of JQ1 and the representative bifunctional compound a1d. **(C–F)** Binding of a1d and JQ1 to $BRD4^{BD1}$ and $BRD4^{BD2}$, either alone or in the presence of 15 µM FKBP12, was assessed using a competitive fluorescence polarization (FP) assay with a fluorescent JQ1 analog as tracer. Data points represent the mean of four replicates, with standard deviations shown as error bars. Data were fitted using a competitive binding model, and the resulting fits are shown as solid lines.

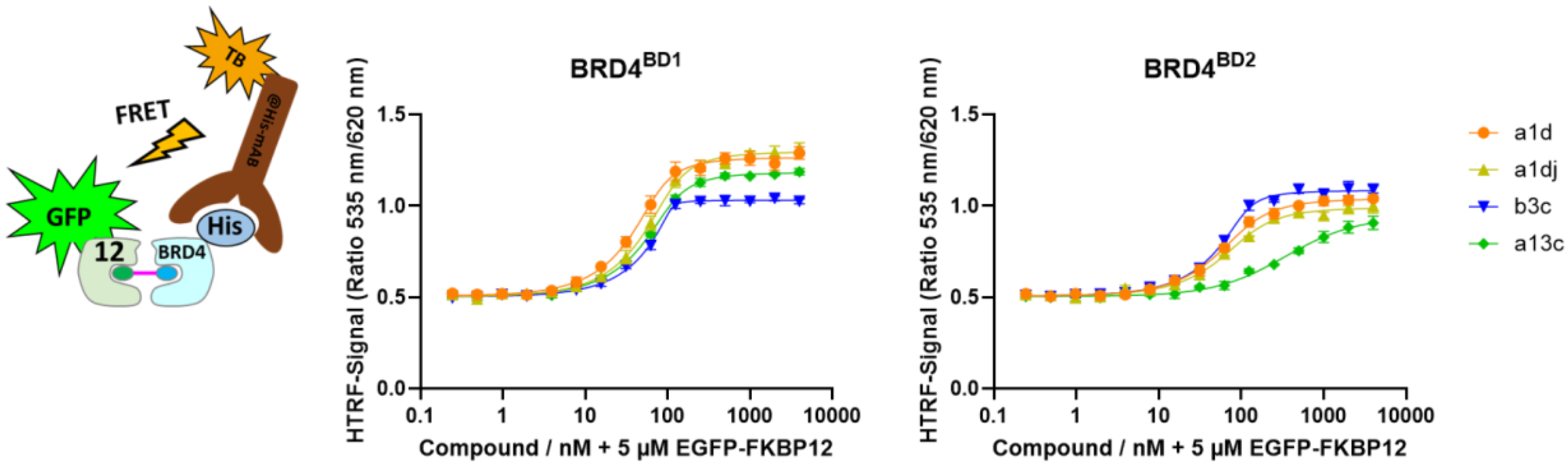

Supplementary Fig. 3 Ternary complex formation assessed by HTRF.

The ternary complex formation between EGFP-FKBP12 and His-BRD4^{BD1} or His-BRD4^{BD2} upon addition of a1d, a1dj, a13c and b3c was determined using a HTRF assay. Therefore a serial dilution of the respective compound was incubated with a mixture of 5 µM EGFP-FKBP12, 100 nM His-BRD4^{BD1} or His-BRD4^{BD2} (complexed with a Terbium-labelled antibody). Data points are indicated as mean from three replicates and standard deviations are indicated as error bars. The solid lines represent data fitted to a binary binding model.

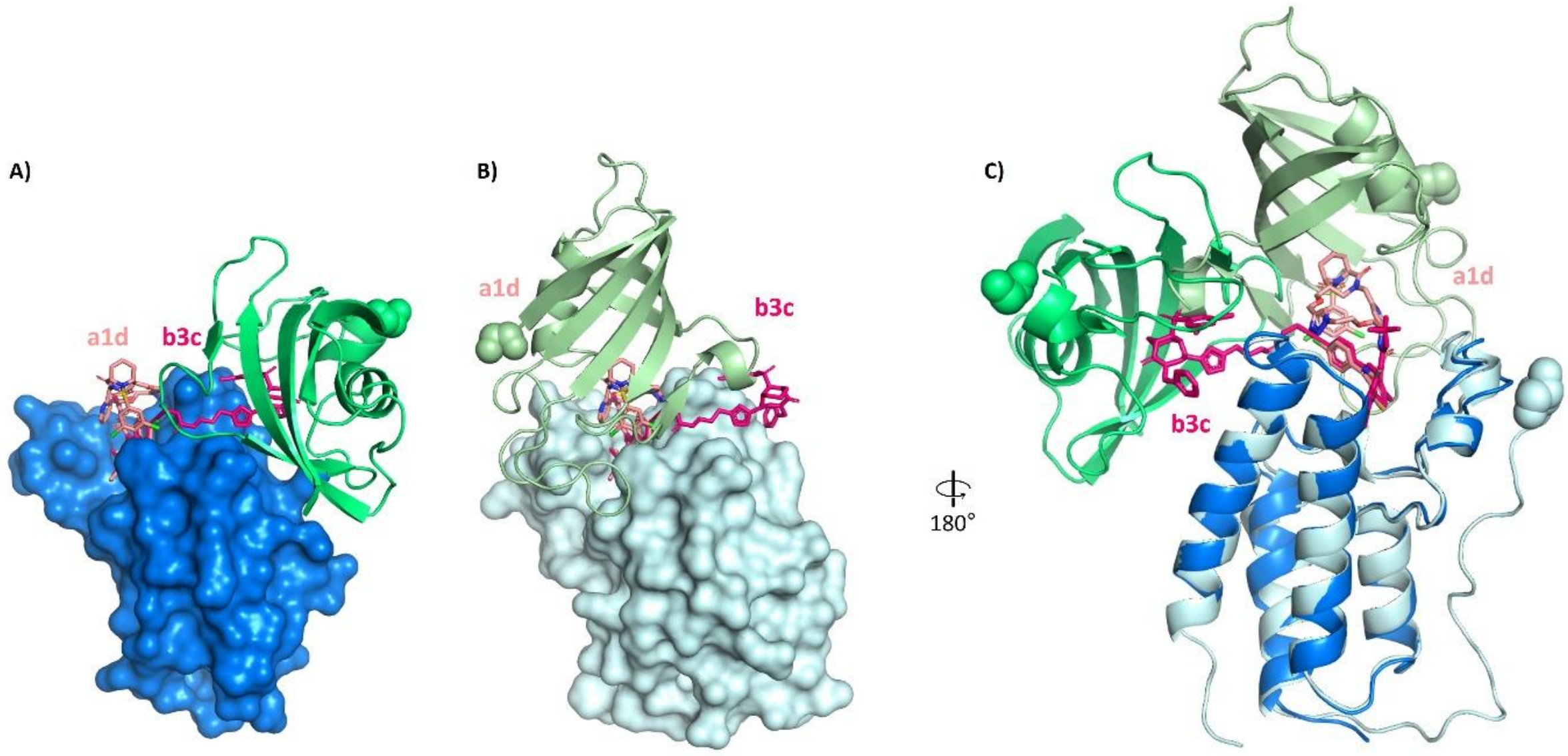

Supplementary Fig. 4 Ternary binding mode of b3c (9R5N) and comparison with a1d (9QW8). **(A)** Ternary complex of BRD4^{BD1} (marine blue surface), b3c (red pink sticks) and FKBP12 (green cartoon). The binding pose of a1d (pale magenta sticks) is shown superimposed from the FKBP12-a1d-BRD4^{BD1} complex. **(B)** Ternary complex of BRD4^{BD1} (pale surface), a1d (pale magenta sticks) and FKBP12 (pale green cartoon), view from the same perspective as in (A). The binding pose of b3c (red pink sticks) is shown superimposed from the FKBP12-b3c-BRD4^{BD1} complex. **(C)** Superposition of the ternary complexes of b3c and a1d, with colors as in (A) and (B) and viewed as in main Fig. 4H (rotated by 180° compared to (A) and (B)). The N-terminal residues of each protein are highlighted as spheres.

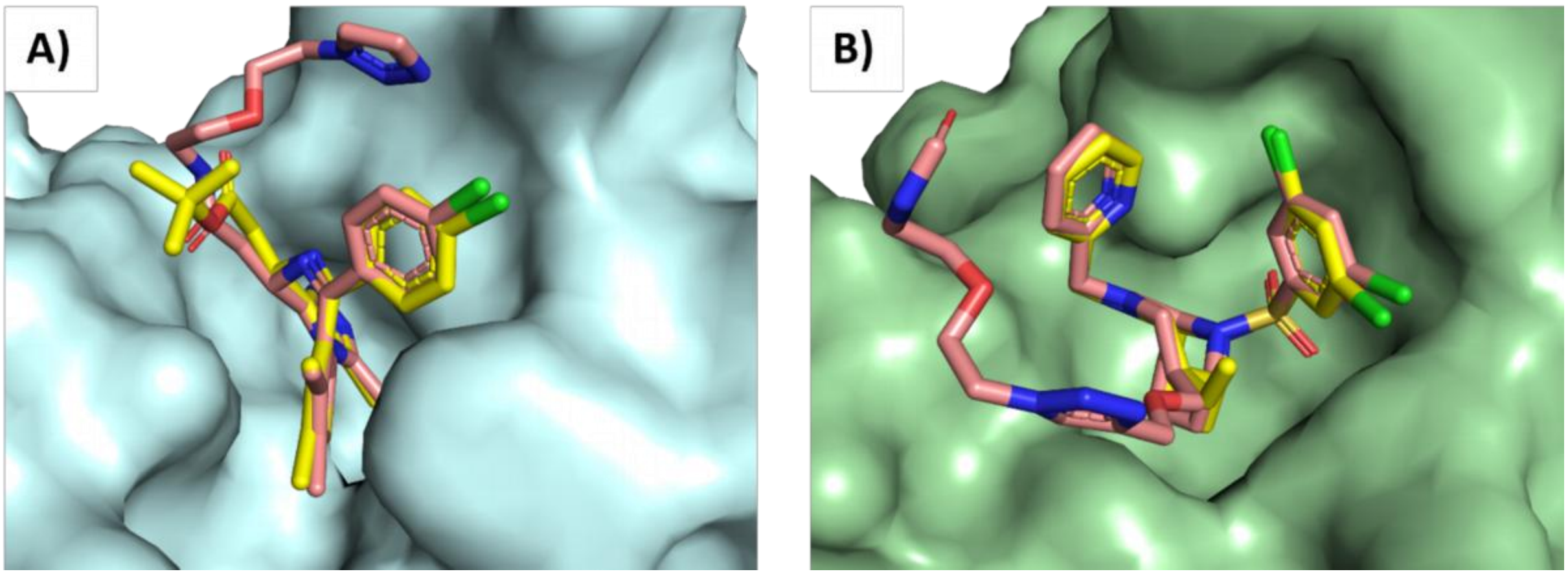

Supplementary Fig. 5 Binding modes of BRD4 & FKBP12 ligands are conserved:

A) JQ1 moiety of **a1d** in the FKBP12-BRD4^{BD1} complex overlayed with JQ1 bound to BRD4^{BD1} (PDB-ID 3MXF). The surface of BRD4^{BD1} is colored cyan, compound **a1d** is colored pale pink and JQ1 is colored in yellow (FKBP12 and FKBP12 binding part of **a1d** omitted for clarity).

B) FKBP12-binding [4.3.1] bicyclic sulfonamide moiety of **a1d** in the FKBP12-BRD4^{BD1} complex overlayed with a [4.3.1] bicyclic sulfonamide FKBP12 ligand bound to FKBP12 (PDB-ID 8CHL). The surface of FKBP12 is colored green, compound **a1d** is colored pale pink and the FKBP12 ligand is colored in yellow (BRD4^{BD1} and JQ1 moiety of **a1d** omitted for clarity).

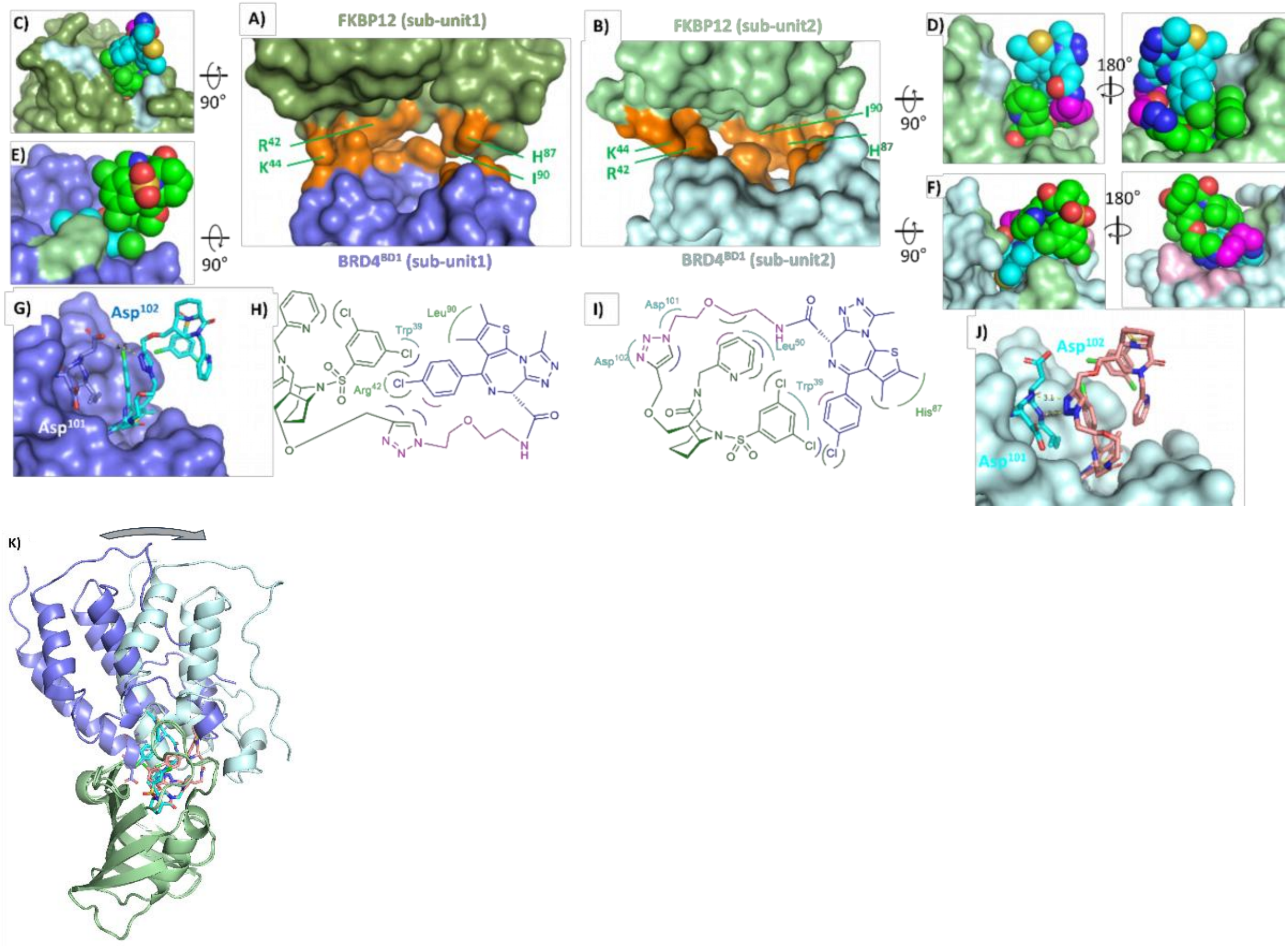

Supplementary Fig. 6. Analyis of ternary complex interactions.

FKBP12 and BRD4^{BD1} are shown as light/dark green and cyan/dark blue surfaces, respectively. Compound **a1d** is shown as sphere model, with carbons of the FKBP12 ligands colored green, carbons of the BRD4 ligands are colored cyan, and carbons of the linker are colored magenta. The two different ternary complexes in the crystallographic unit are shown in light and dark colors, respectively. **(A,B)** Amino residues engaged in direct protein-protein contacts are highlighted in orange. The ligand **a1d** has been omitted for clarity. **(C,D) a1d** bound to FKBP12, BRD4^{BD1} is not shown for clarity. FKBP12 residues contacting the BRD4 ligand part are colored pale cyan. **(E,F) a1d** bound to BRD4^{BD1}, FKBP12 is not shown for clarity. BRD4^{BD1} residues contacting the FKBP12 ligand part are colored pale green and BRD4^{BD1} residues contacting the linker are colored pale pink. **(G,J)** Interaction of linker triazole moiety of **a1d** with BRD4^{BD1}. **(H,I)** Lig Plot analysis of intra-ligand and 'cross-over' contacts of **a1d** bound to FKBP12 and BRD4^{BD1} in the two subunits. **(K)** Structures of the two FKBP12-a1d-BRD4^{BD1} complexes observed in the crystal, super-positioned by their FKBP12 unit. **a1d** is shown as cyan or pale pink sticks.

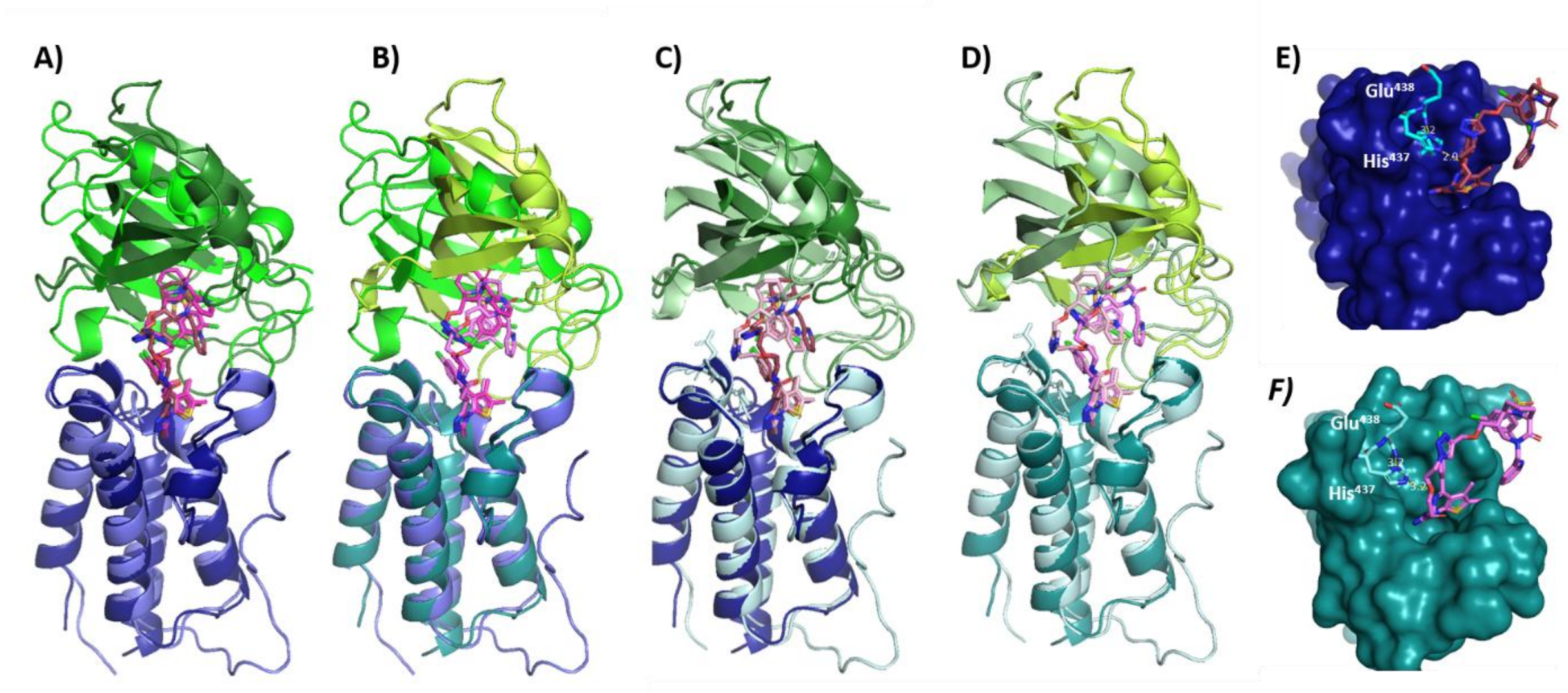

Supplementary Fig. 7 Structure of the FKBP12:a1d:BRD4^{BD2} ternary complex (PDB-ID 29QL). Two different ternary FKBP12:a1d:BRD4^{BD2} complexes are present in the crystallographic unit cell (sub-complexes BD2a and BD2b). In the FKBP12:a1d:BRD4^{BD2a} complex, FKBP12, a1d and BRD4^{BD2} are colored dark green, raspberry and dark blue, and in the FKBP12:a1d:BRD4^{BD2b} complex, FKBP12, a1d and BRD4^{BD2} are colored limon, violet and teal, respectively. **A-D)** Superposition of the two BRD4^{BD2}-containing ternary complexes on the each of the FKBP12:a1d:BRD4^{BD1} ternary complex (from PDB-ID 9QW8), aligned on the BRD4BD subunits. Proteins are shown as cartoons, a1d is shown as sticks. **E,F)** Binding of a1d to BRD4^{BD2} in the ternary complexes (FKBP12 omitted for clarity). H^{437}EV^{430} of BRD4^{BD2}-is highlighted as sticks. Hydrogen bonds from H^{437} to V^{430} to and the linker oxygen are sowh as yellow dotted lines, with distances in Å.

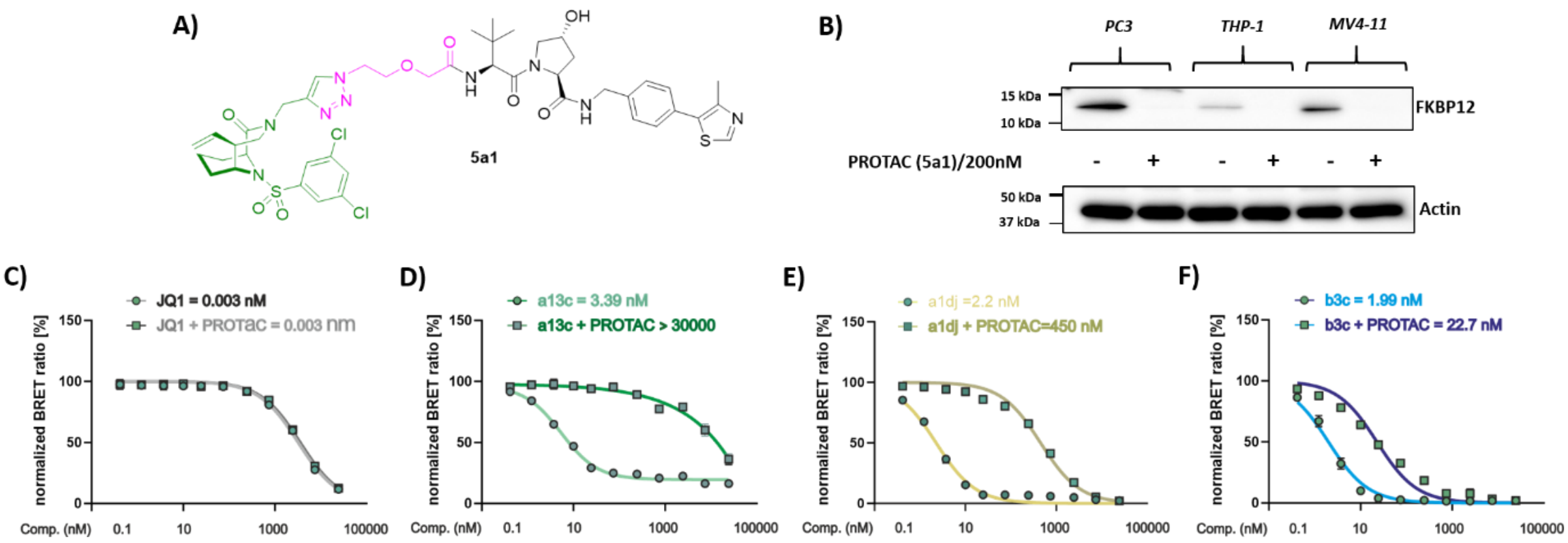

Supplementary Fig 8 Western Blot analysis of FKBP12 levels and NanoBRET assessment of BRD4 in PC3 cells.

(A) Chemical structure of the high-potency FKBP12 PROTAC 5a1. **(B)** PC3 cells, serving as a positive control, were seeded in a 6-cm plate, while THP1 and MV4-11 cell lines were seeded in T25 flasks for two days. Following this incubation, all cell lines were treated with 200 nM FKBP12-specific PROTAC for six hours to deplete FKBP12 protein levels. Western blot analysis was then performed to evaluate FKBP12 levels **(C-F)** NanoBRET assays performed in PC3 cells constitutively expressing BRD4–NanoLuc as a fusion protein. Cells were treated with JQ1 (C), a13c (D), a1dj (E), or b3c (F) in the presence or absence of FKBP12. Data points represent the mean of biological replicates.

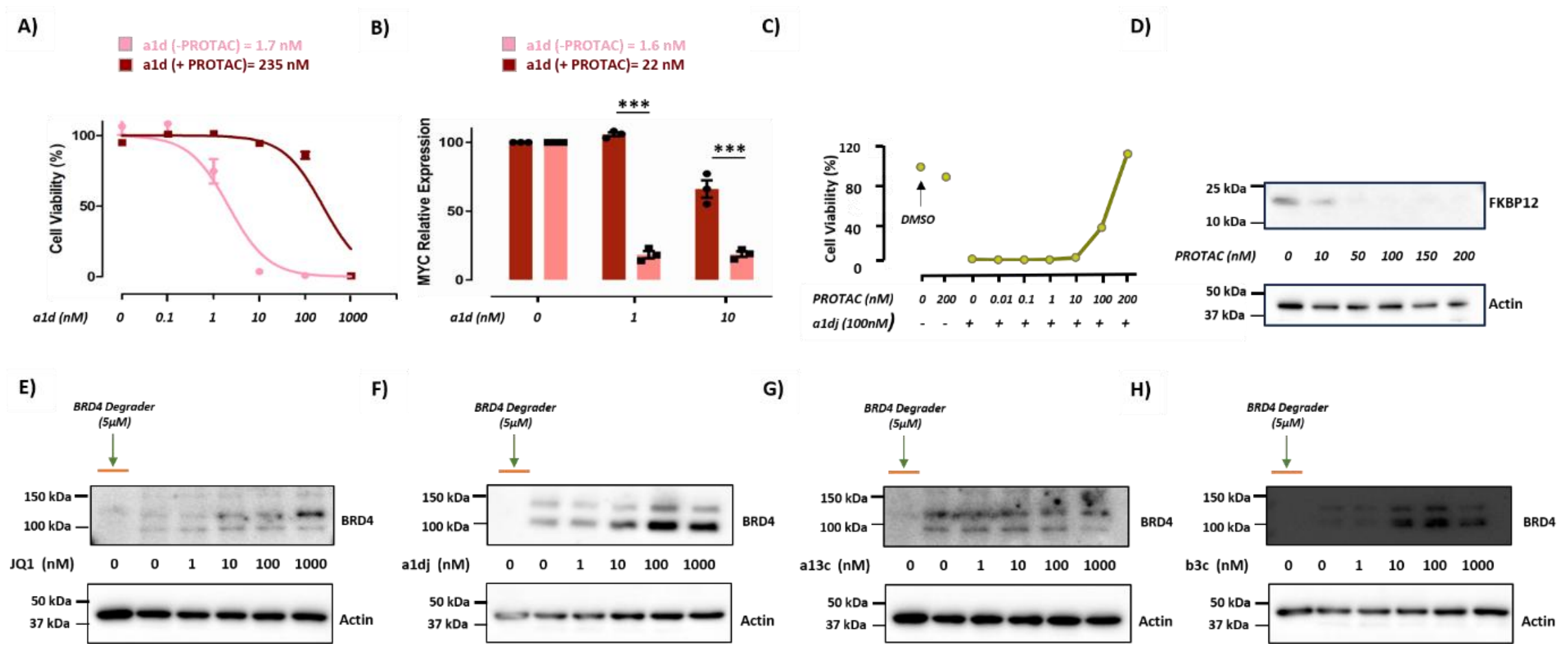

Supplementary Fig. 9 FKBP12-dependent cytotoxic effect of BRD4-targeting bifunctionals independent of BRD4 degradation.

Cell toxicity for MV4-11 **(A)** treated for five days with the indicated concentrations of a1d in the presence or absence of PROTAC. Pink lines indicate absence of PROTAC, brown lines represent the PROTAC treatment. Data are presented as mean ± standard deviation (SD). Estimated IC_{50} values in the presence or absence of FKBP12 PROTAC are indicated above the graph. **(B)** Quantitative analysis of MYC mRNA levels in MV4-11 following treatment with a1d. Data are presented as mean ± standard deviation. IC_{50} indicated above the graph. Two-way ANOVA followed by Bonferroni's multiple-comparisons test revealed significant effects of treatment ($F(1,4) = 399.1$, $P < 0.0001$), concentration ($F(2,8) = 180.0$, $P < 0.0001$), and treatment × concentration interaction ($F(2,8) = 100.1$, $P < 0.0001$). Bonferroni-adjusted pairwise comparisons showed significant differences at 1 nM and 10 nM (all $P < 0.0001$). **(C)** Cell Toxicity for MV4-11 treated for five days with 100 nM a1dj, after depleting the FKBP12 using different concentrations of FKBP12-specific PROTAC. **(D)** Western Blot analysis of FKBP12 depletion in MV4-11 cells. **(E-H)** BRD4 levels analyzed by Western blot in MV4-11 cells following 6 hours treatment with increasing concentrations of (E) JQ1, (F) a1dj, (G) a13c, and (H) b3c. A BRD4 degrader was included as a control and loaded in the first lane of each blot. Actin was used as a loading control.

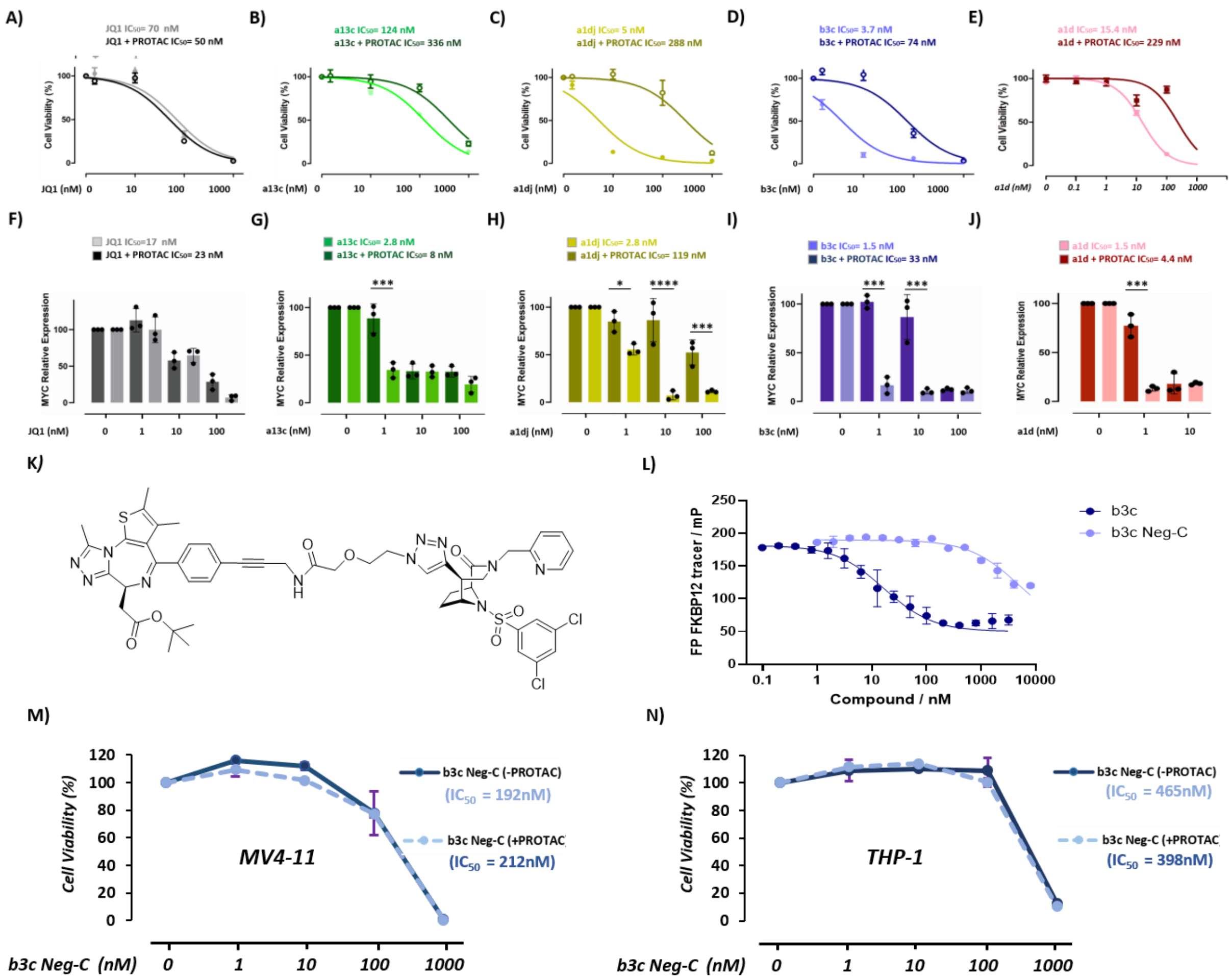

Supplementary Fig. 10 FKBP12-assisted toxicity by BRD4-targeting bifunctionals in THP1 and MV4-11 Cells.

(A-E) Cell toxicity for THP1 cells treated for five days with the indicated concentrations of (A) JQ1, (B) a13c, (C) a1dj, (D) b3c, and (E) a1d. The treatment was done either in the presence or absence of FKBP12 PROTAC. Estimated IC_{50} values in the presence or absence of FKBP12 PROTAC are indicated above each panel. **(F-J)** Quantitative analysis of MYC mRNA levels in THP1 cells following treatment with (F) JQ1, (G) a13c, (H) a1dj, (I) b3c, and (J) a1d. for six hours, either in the presence or absence of PROTAC. Two-way ANOVA (two-sided) followed by Bonferroni's multiple-comparisons test was performed for panels F–J. For a13c (a13c vs. a13c + PROTAC), significant effects of treatment ($F(1,4) = 21.95$, $P = 0.0094$), concentration ($F(3,12) = 122.0$, $P < 0.0001$), and treatment × concentration interaction ($F(3,12) = 17.45$, $P < 0.0001$) were observed. Bonferroni-adjusted pairwise comparison was significant at 1 nM ($P < 0.0001$). For a1dj (a1dj vs. a1dj + PROTAC), the effects of treatment ($F(1,4) = 224.8$, $P < 0.0001$), concentration ($F(3,12) = 40.24$, $P < 0.0001$), and treatment × concentration interaction ($F(3,12) = 12.84$, $P = 0.0006$) were observed. Bonferroni-adjusted pairwise comparisons were significant at 1 nM ($P = 0.0130$), 10 nM ($P < 0.0001$), and 100 nM ($P = 0.0007$). For b3c (b3c vs. b3c + PROTAC), significant effects of treatment ($F(1,4) = 44.71$, $P = 0.0026$), concentration ($F(3,12) = 72.90$, $P < 0.0001$), and treatment × concentration interaction ($F(3,12) = 33.70$, $P < 0.0001$) were

observed. Bonferroni-adjusted pairwise comparisons were significant at 1 nM ($P < 0.0001$) and 100 nM ($P = 0.0012$). **(K)** Chemical structure of bc3 Neg-C, which does not bind FKBP12 and serves as a negative control for b3c. **(L)** The binding to FKBP12 of compounds b3c and b3c Neg-C was determined by a competitive FP assay using a fluorescent FKBP12 ligand as tracer. The solid lines represent data fitted to a competitive binding model. **(M,N)** Cell viability assays for MV4-11 (M) and THP-1 (N) treated for five days with the indicated concentrations of the none-FKBP12 targeting analog b3c-neg. Dark blue continuous lines indicate absence of PROTAC, light blue intermittent lines represent the PROTAC treatment. All experiments were performed in triplicate, except for (L), which was conducted in replicates, either in the presence or absence of FKBP12 PROTAC. Error bars represent the mean ± standard deviation (SD) from three independent experiments, or from replicates (L).

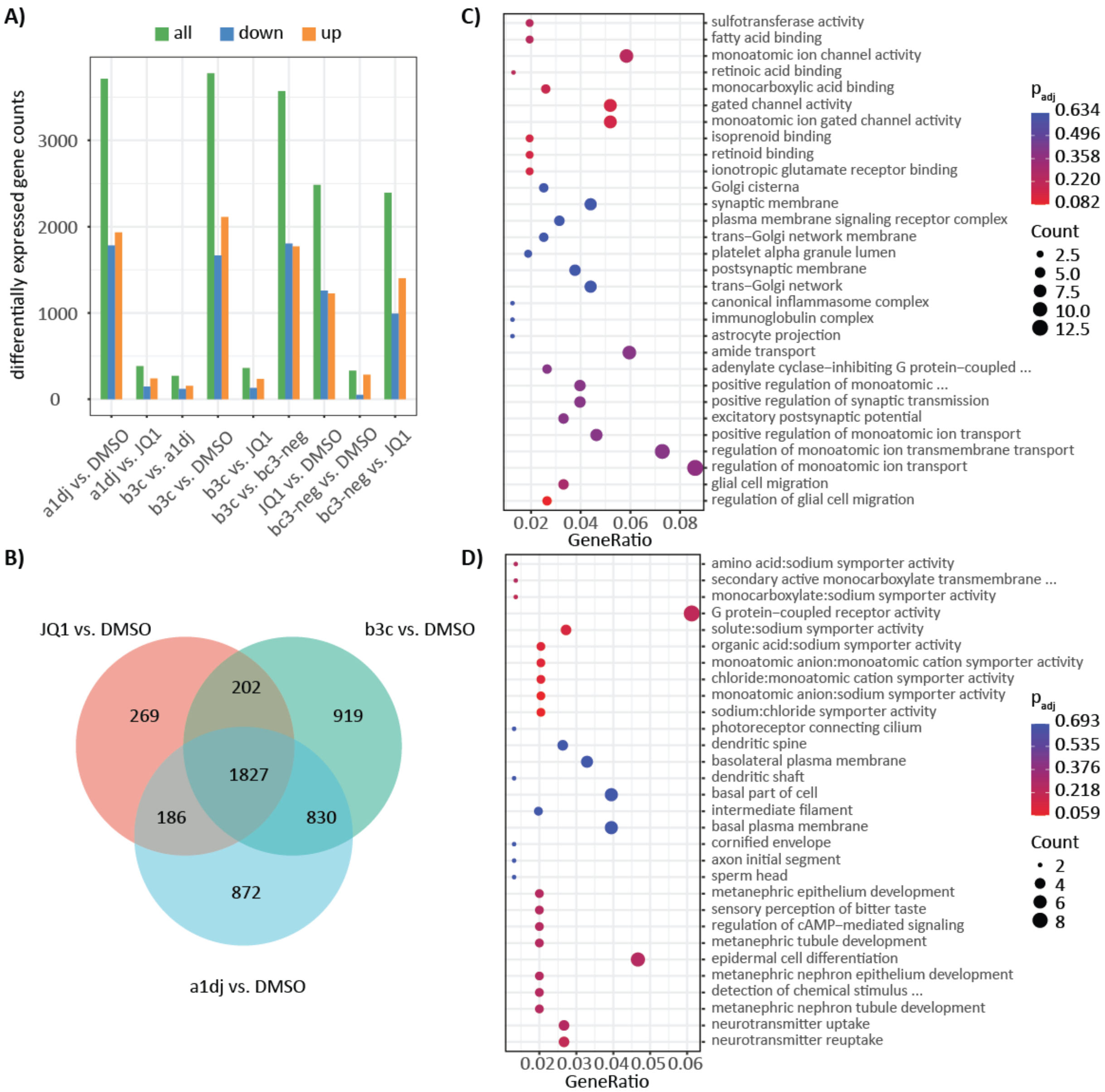

Supplementary Fig. 11 Transcriptome-wide analysis of changes in gene expression.

(A) Summary of differentially expressed genes across treatment conditions (**B)** Venn diagram showing the number of differentially expressed genes for JQ1, b3c, and a1dj compared with DMSO. Most regulated genes are shared among all three treatments, reflecting common BRD4-dependent gene expression. (**C-D)** Pathway enrichment using differential gene expression upon a1dj (C) or b3c (D) treatment compared to JQ1 treated cells. Gene ratio is the proportion of differentially expressed genes found within the indicated biological process (derived from the GO database) compared to the total genes annotated to that pathway.

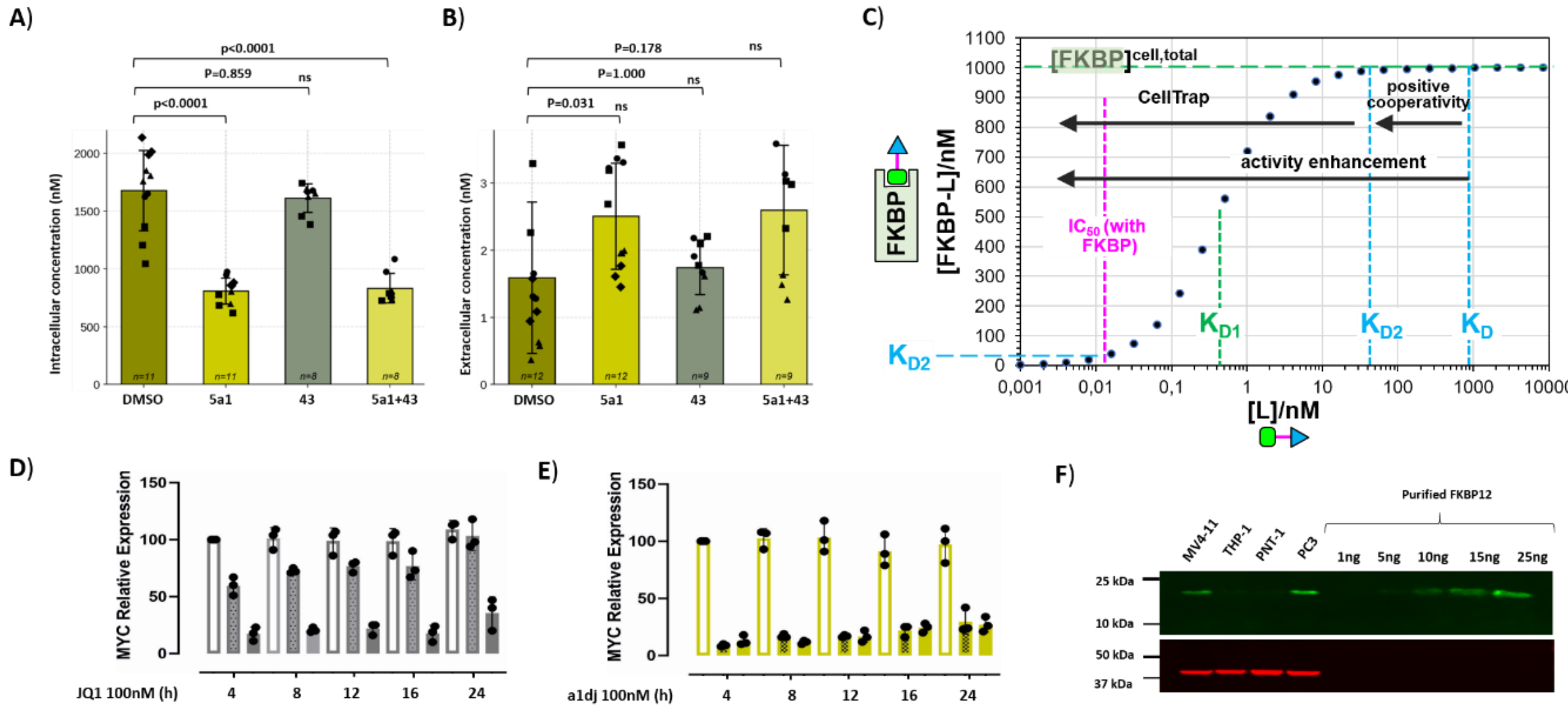

G)

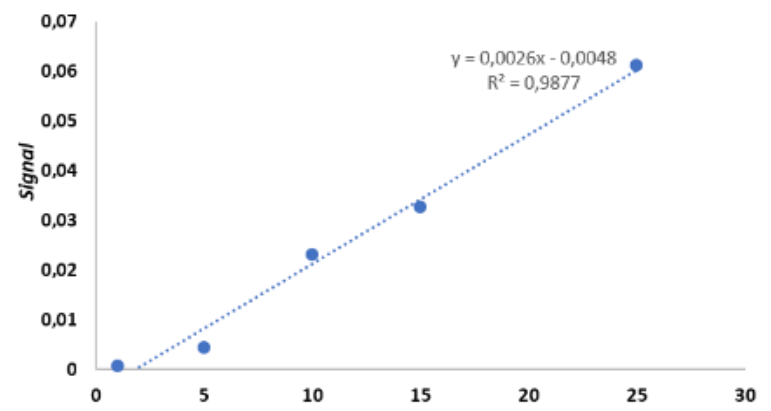

H)

Cell line	Total Protein (ng)	Cell number	Cell Diameter (µm)	Cell Volume (pL)	c(FKBP12 per cell) (µM)
PC3	15,9	$4x10^5$	17.6 ± 1.2	2.8 ± 0.5	1,158 ± 1.2
PNT1A	8,8	$4.4x10^5$	11.9 ± 0.7	1.1 ± 0.2	0,573 ± 1.5
MV4-11	2,6	$5.2x10^5$	13.8 ± 1.4	1.1 ± 0.3	1,252 ± 3.4
THP-1	3	$4.4x10^5$	12.6 ± 1.2	0.9 ± 0.3	0,499 ± 1.6

Supplementary Fig. 12 FKBP12-driven cellular accumulation and assisted activity enhancement exemplified by a1dj.

(A,B) Intracellular (A) and cell culture medium (B) concentrations of a1dj in MV4-11 cells, pooled across four independent experiments (n=2–3 biological replicates per group per experiment), were compared between control cultures and cultures pretreated with the FKBP12-degrading PROTAC 5a1 or the BRD4-degrading PROTAC 43 (100 nM each in 1% DMSO, 5 h), either alone or in combination, followed by treatment with a1dj (10 nM in 2% DMSO total, 2 h; total exposure time to 5a1/43: 7 h); final DMSO concentration 2% (v/v). Pretreatment with 5a1, but not 43, resulted in decreased intracellular accumulation of a1dj across all experiments, consistent with FKBP12-dependent cellular enrichment. A directionally consistent increase in extracellular a1dj was observed in 5a1-treated cultures, reaching statistical significance in the pooled analysis (Tukey-adjusted p=0.031 vs DMSO), though with inter-experiment variability attributable to technical factors. Data represent mean ± SD of pooled replicates, with individual data points shown per experiment round using distinct marker shapes (● Round 1, ■ Round 2, ▲ Round 3, ◆ Round 4). a1dj concentrations were quantified by LC-MS/MS. One-way ANOVA with Tukey's HSD post hoc test identified a main effect of treatment on intracellular ($F(1, 20) = 62.84$, $p < 0.0001$) and extracellular concentrations ($F(1, 22) = 5.33$, $p < 0.031$). Adjusted p-values are shown in the figure. **(C)** The formation of the intracellular FKBP12-ligand complex in dependence of ligand concentration is modelled assuming a total intracellular FKBP12 concentration of 1µM[1] and no ligand depletion (unlimited replenishment of free intracellular ligand by the extracellular ligand reservoir). Due to the high intracellular FKBP12 concentration and the high K_{D2} for ternary complex formation, only 1,4% of FKBP12 needs to the occupied to bind 50% of BRD4. Due to the high affinity to FKBP12 this is achieved at very low concentrations (12pM), representing an activity

enhancement of more than 5 orders of magnitude. We note that under these extreme conditions the assumptions of ligand non-depletion in the extracellular medium might not be valid anymore. **(D,E)** Quantitative analysis of MYC mRNA levels in MV4-11 cells treated with (D) JQ1 or (E) a1dj. After treatment, JQ1 and a1dj were washed off with PBS and MYC mRNA level were determined using qPCR at indicated time points. **(F)** Immunoblot analysis showing FKBP12 levels in different cell lines alongside increasing amounts of purified FKBP12 protein used as standards. β-Actin was included as a loading control. **(G)** Standard curve generated from the quantified FKBP12 signals of the purified protein. **(H)** Table summarizing cell diameter, estimated cell volume, and calculated intracellular FKBP12 concentrations for each cell line. Error bars represent the mean ± standard deviation from three independent experiments

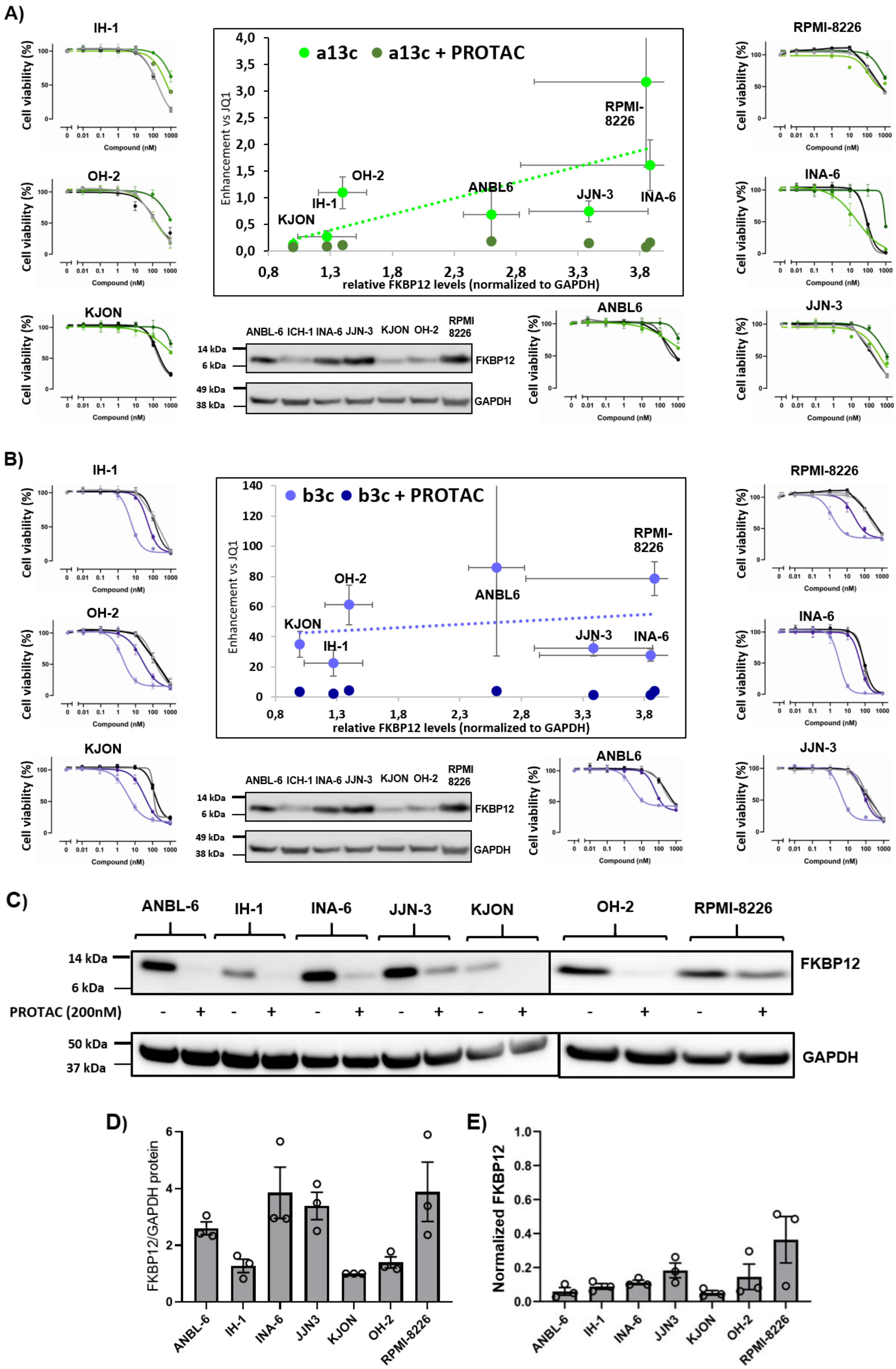
A)
IH-1
OH-2
KJON
RPMI-8226
INA-6
JJN-3
ANBL6
Cell viability (%)
Compound (nM)
a13c
a13c + PROTAC
Enhancement vs JQ1
relative FKBP12 levels (normalized to GAPDH)
RPMI-8226
ANBL6
JJN-3
INA-6
ANBL-6 ICH-1 INA-6 JJN-3 KJON OH-2 RPMI 8226
14 kDa
6 kDa
49 kDa
38 kDa
FKBP12
GAPDH
B)
b3c
b3c + PROTAC
C)
ANBL-6
IH-1
INA-6
JJN-3
KJON
OH-2
RPMI-8226
14 kDa
6 kDa
PROTAC (200nM)
50 kDa
37 kDa
FKBP12
GAPDH
D)
FKBP12/GAPDH protein
ANBL-6
IH-1
INA-6
JJN3
KJON
OH-2
RPMI-8226
E)
Normalized FKBP12

F)

IC50 Values

Cell Line	JQ1-	JQ1 +PROTAC	a1dj -PROTAC	a1dj +PROTAC	a13c -PROTAC	a13c +PROTAC	b3c -PROTAC	b3c +PROTAC
ANBL6	240	230	1.6	200	350	1200	2.8	60
IH1	110	110	19	430	400	1200	4.9	50
INA6	89	77	1.4	210	28	960	3.2	54
JJN3	120	105	2.3	140	160	680	3.7	80
KJON	140	120	11	280	1100	1500	4.0	36
OH2	110	110	2.4	100	100	950	1.8	24
RPMI	110	110	0.6	52	68	670		

Mean FKBP12 Levels (Normalized to GAPDH)

Cell Line	Mean FKBP12
ANBL6	2.60
IH1	1.27
INA6	3.85
JJN3	3.39
KJON	1.00
OH2	1.40
RPMI	3.88

Enhancement vs JQ1

Cell Line	JQ1/a1dj - PROTAC	JQ1/a1dj + PROTAC	JQ1/a13c - PROTAC	JQ1/a13c + PROTAC	JQ1/b3c- PROTAC	JQ1/b3c+ PROTAC
ANBL6	150.00	1.15	0.69	0.19	85.71	3.83
IH1	5.79	0.26	0.28	0.09	22.45	2.20
INA6	63.57	0.37	3.18	0.08	27.81	1.43
JJN3	52.17	0.75	0.75	0.15	32.43	1.31
KJON	12.73	0.43	0.13	0.08	35.00	3.33
OH2	45.83	1.10	1.10	0.12	61.11	4.58
RPMI	183.33	2.12	1.62	0.16	78.57	3.79

Supplementary Fig. 13 FKBP12-enhanced cell toxicity in myeloma cell lines. (A) Cell toxicity of a13c (green) correlates with FKBP12 expression levels and is substantially reduced after FKBP12 knockdown using a FKBP12 PROTAC. **(B)** b3c (blue) is substantially enhanced compared to JQ1 in a panel of myeloma cell lines and this is abolished after FKBP12 knockdown using a FKBP12 PROTAC. **(C)** Western Blot analysis of FKBP12 levels in the indicated myeloma cell lines in the presence or absence of FKB12 PROTAC. **(D)** Quantification of FKBP12 levels normalized to GAPDH in the indicated myeloma cell lines. Circles represent measurements from three independent repeats, bars represent mean levels, error bar standard deviations. **(E)** Quantification of FKBP12 depletion upon PROTAC treatment in the indicated myeloma cell lines. Data are reported as FKBP12 levels (normalized to GAPDH) after PROTAC treatment relative to FKBP12 levels (normalized to GAPDH) without PROTAC treatment. Circles represent measurements from three independent repeats, bars represent mean levels, error bar standard deviations. **(F)** Quantification of IC_{50} values and FKBP12 expression levels from Fig. 8E and SI Fig. 13A and B.

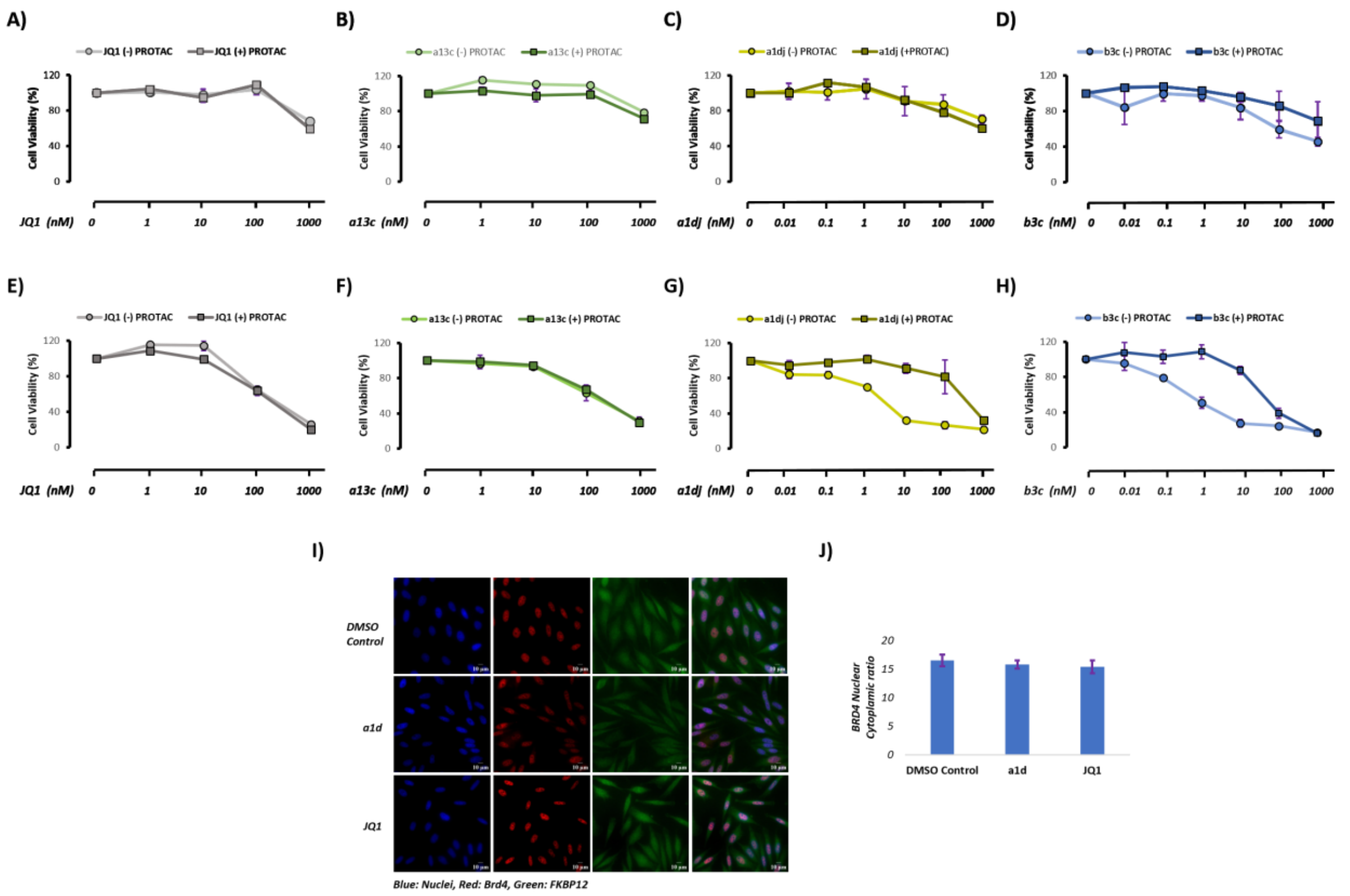

Supplementary Fig. 14 FKBP12-assissted toxicity and FKBP12 localization in PC3 PNT1-A cells following bifuctionals treatment.

(A-D) Cell toxicity for PNT1-A cells treated for five days with the indicated concentrations of (A) JQ1, (B) a13c, (C) a1dj, and (D) b3c. All experiments were performed in triplicate, either in the presence (lighter colors) or absence (darker color) of FKBP12 PROTAC. **(E-H)** Cell toxicity for PC3 cells treated for five days with the indicated concentrations of (E) JQ1, (F) a13c, (G) a1dj, and (H) b3c. All experiments were performed in triplicate, either in the presence (lighter colors) or absence (darker color) of FKBP12 PROTAC. Error bars represent the mean ± standard deviation (SD) from three independent experiments. **(I)** Immunofluorescence staining of PC3 cells treated with a1d, JQ1, or DMSO (control) for 6 hours. Scale bar in (I): 10 µm **(J)** Quantification of the BRD4/FKBP12 nuclear ratio from the images shown in (I). The mean ratio of 100 cells is shown with error bars indicate the mean ± standard deviation from three independent experiments

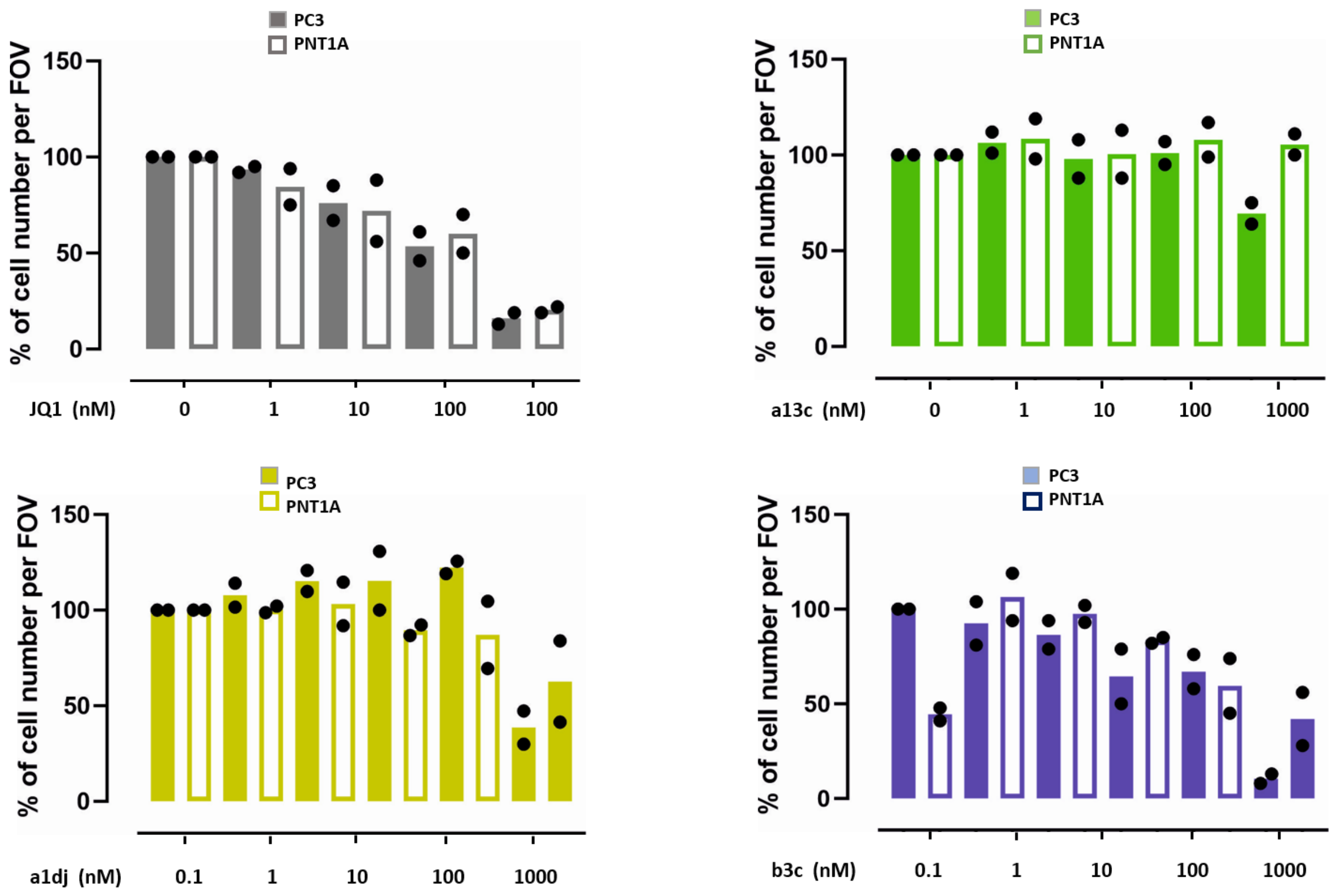

Supplementary Fig. 15 Cytotoxicity of co-cultured PC3 carcinoma and non-transformed PNT1A cells in the absence of FKBP12.

(A-D) Microscopic cell count analysis in PNT1-A and PC3 cells following treatment for five days with various concentrations of (A) JQ1, (B) a13c, (C) a1dj, and (D) b3c. All cells were treated with the indicated compounds after FKBP12-degradation using PROTAC. The graphs represent the data obtained from two independent live cell microscopy experiments.

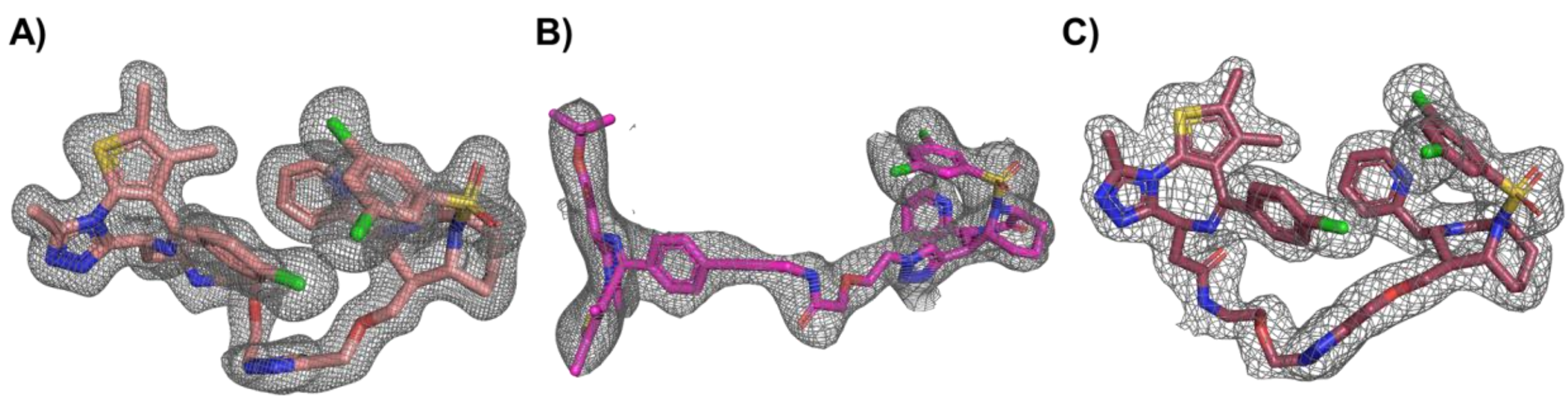

Supplementary Fig. 16 Representive electron density for a1d in PDB-ID 9QW8 (A), b3c in PDB-ID 9R5N (B) and a1d in PDB-ID 29QL (C).

The electron density (2Fo-Fc) at a contour level of 1σ is indicated as grey mesh.

^{1}H and ^{13}C spectra of the precursor compounds

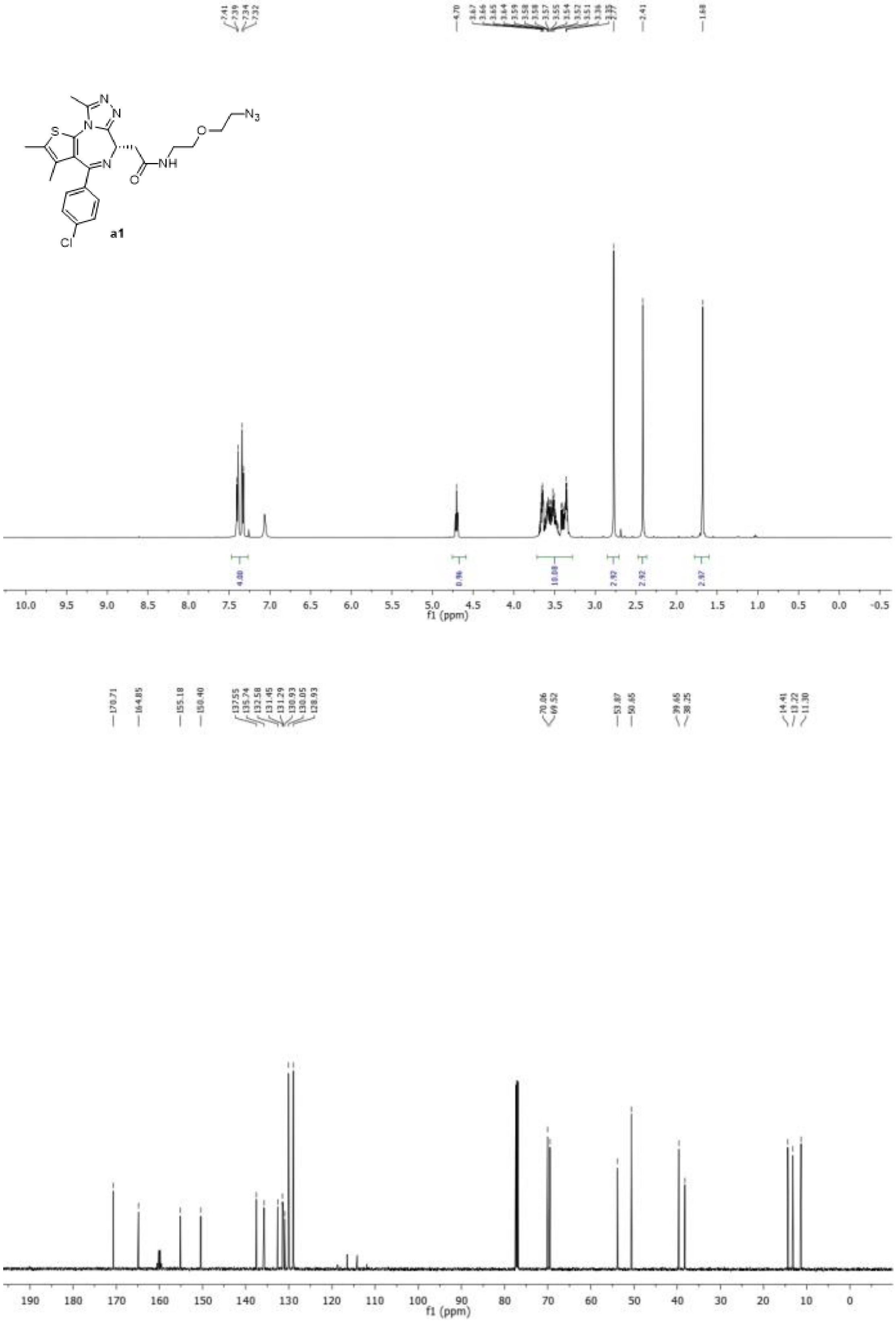

Supplementary Fig. 17

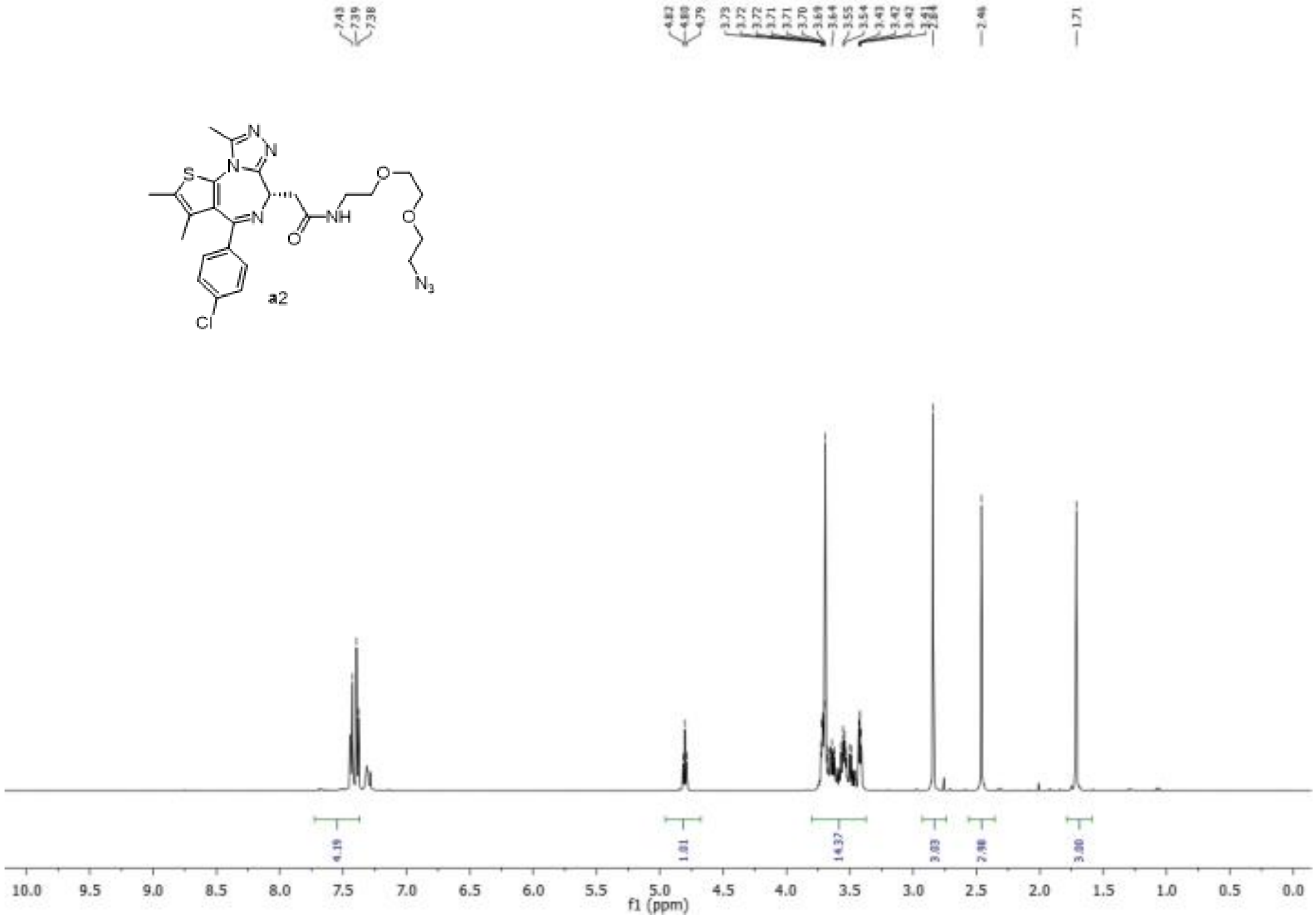

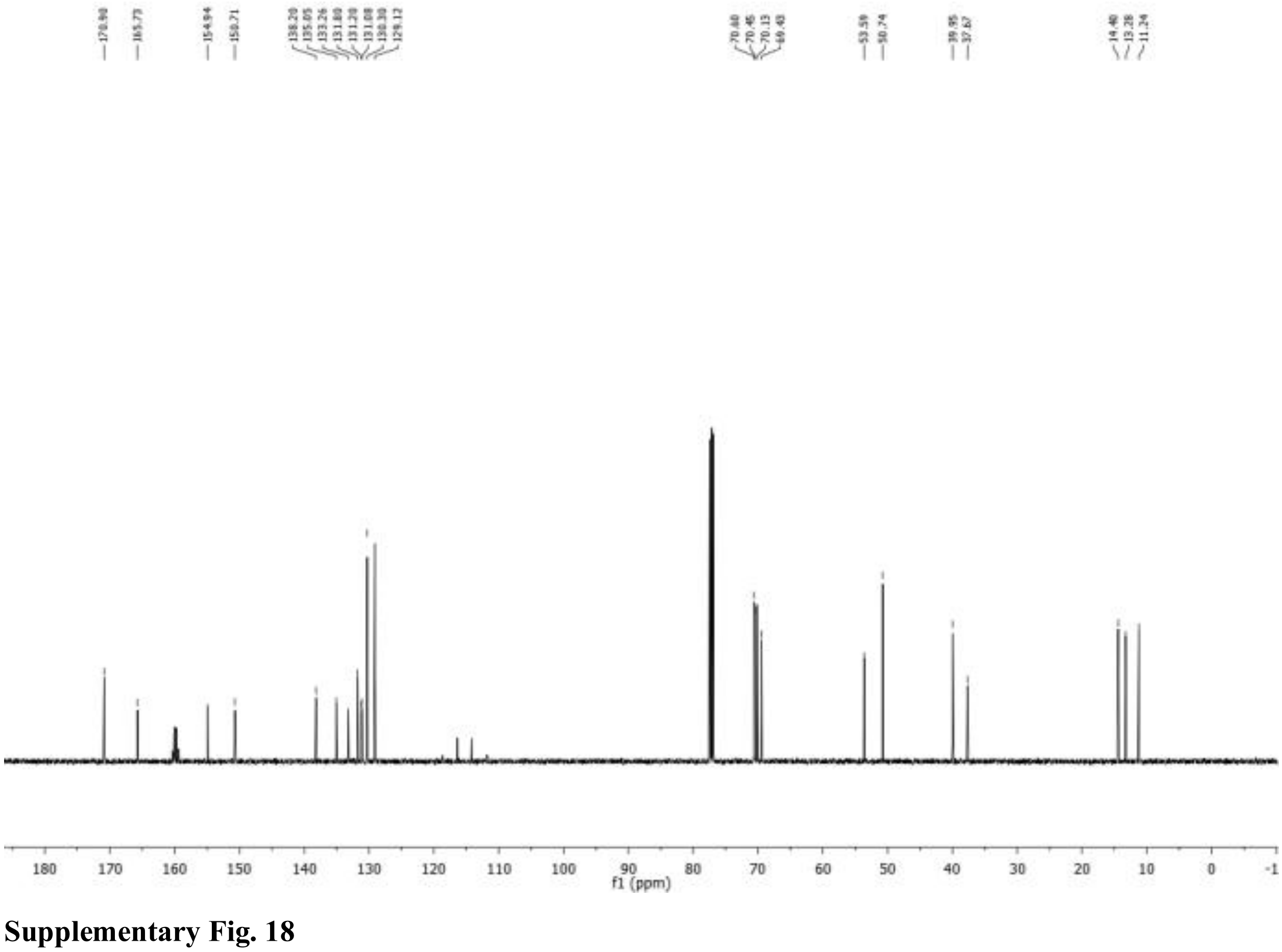

Supplementary Fig. 18

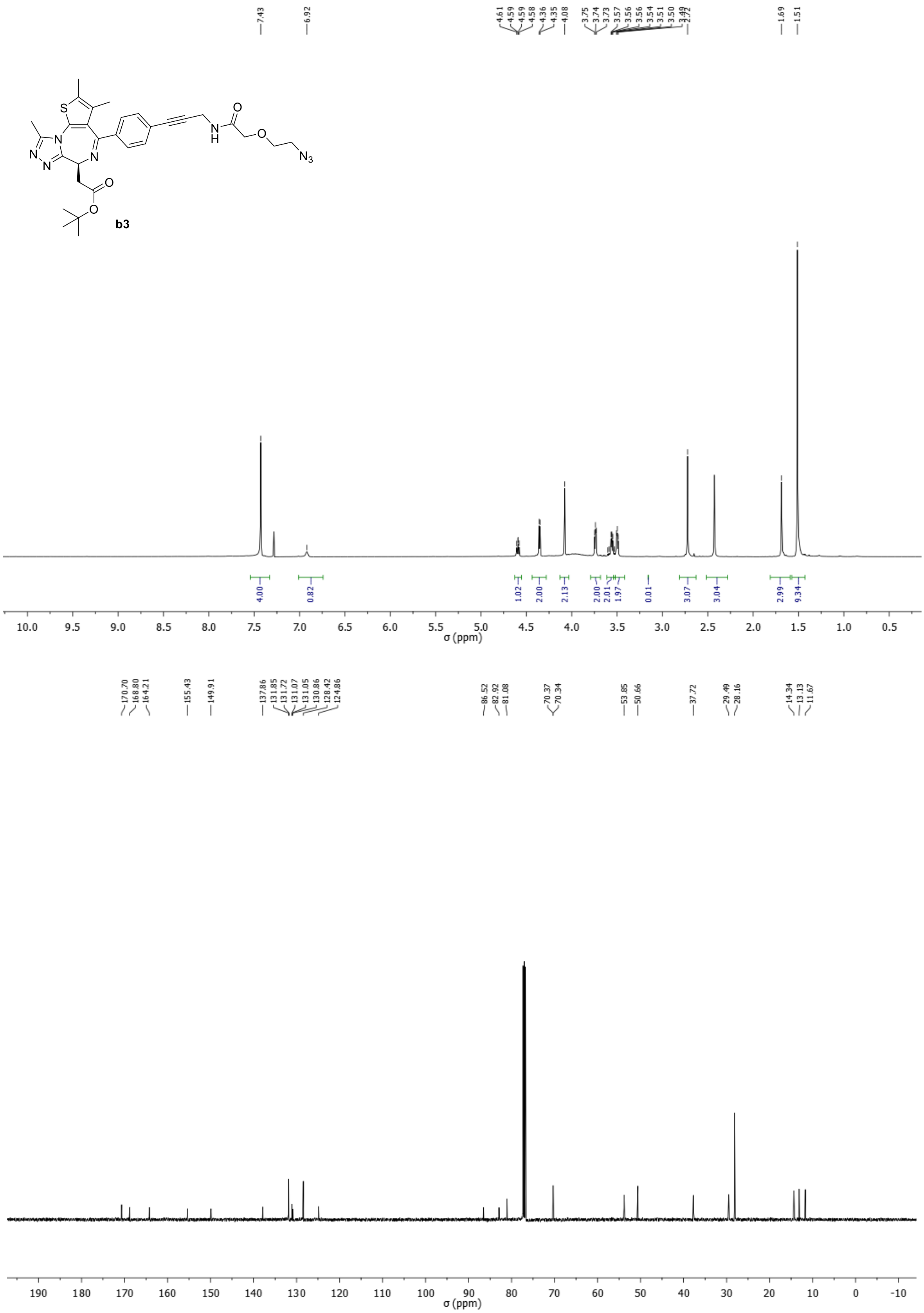

Supplementary Fig. 19

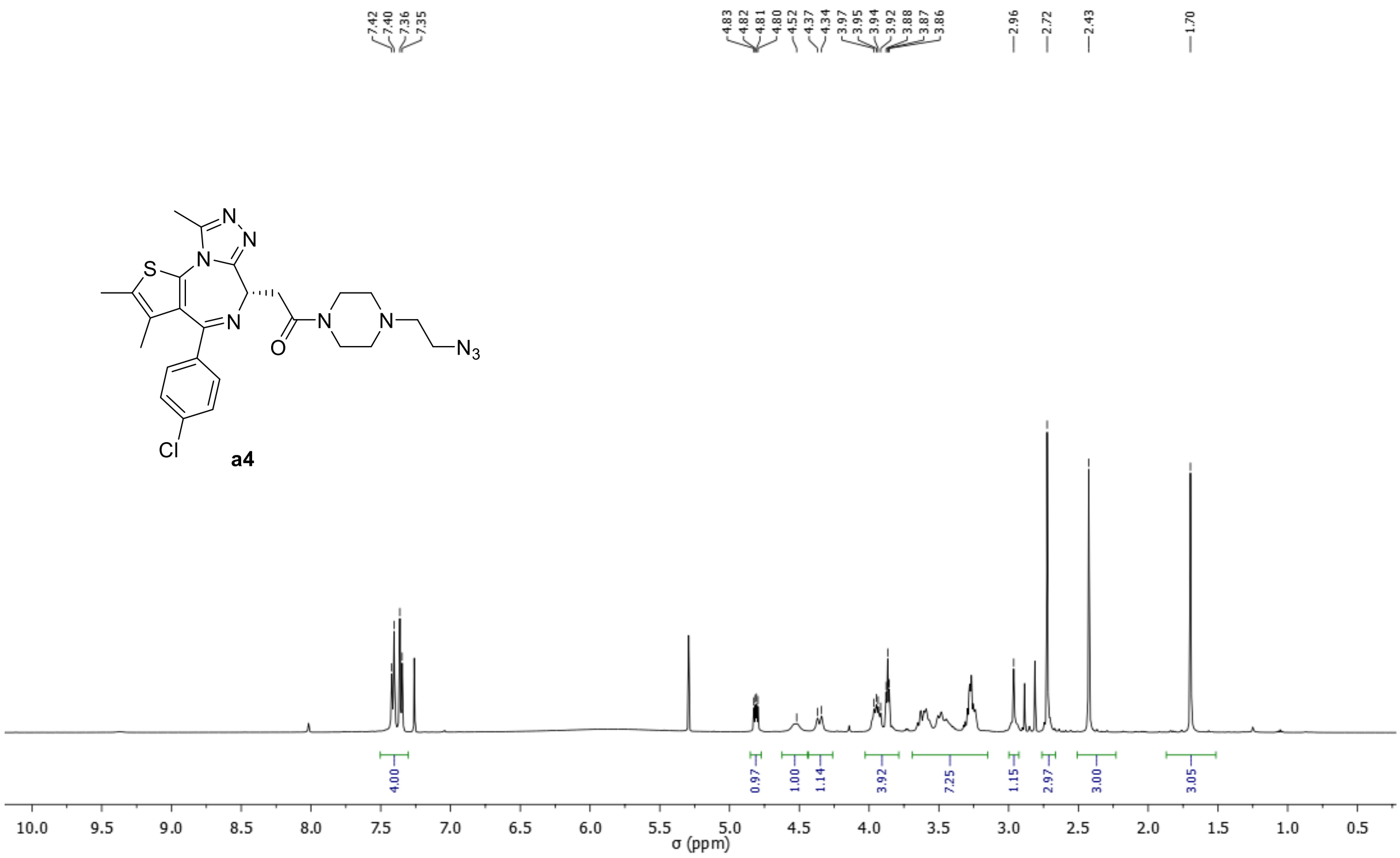

a4

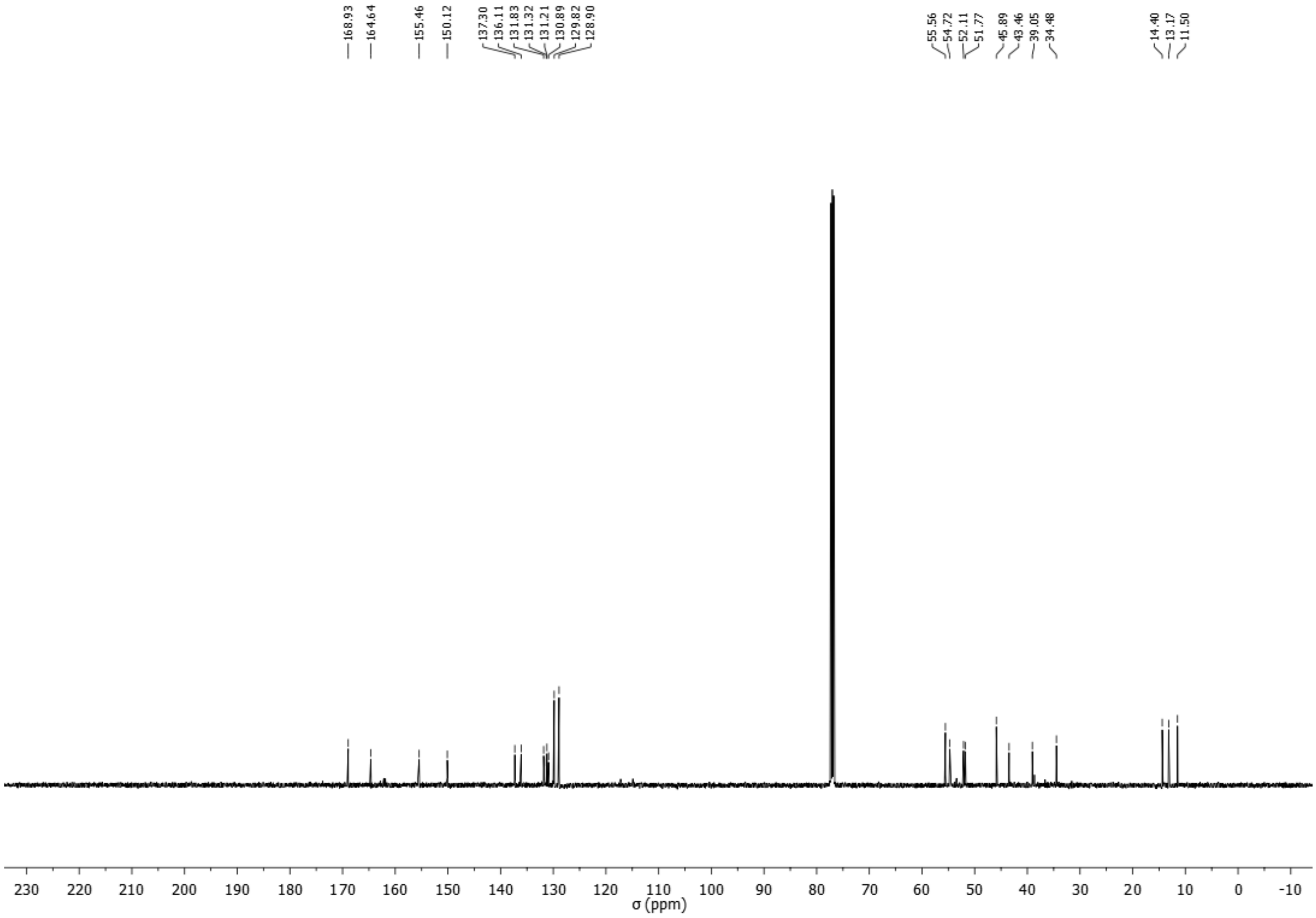

Supplementary Fig. 20

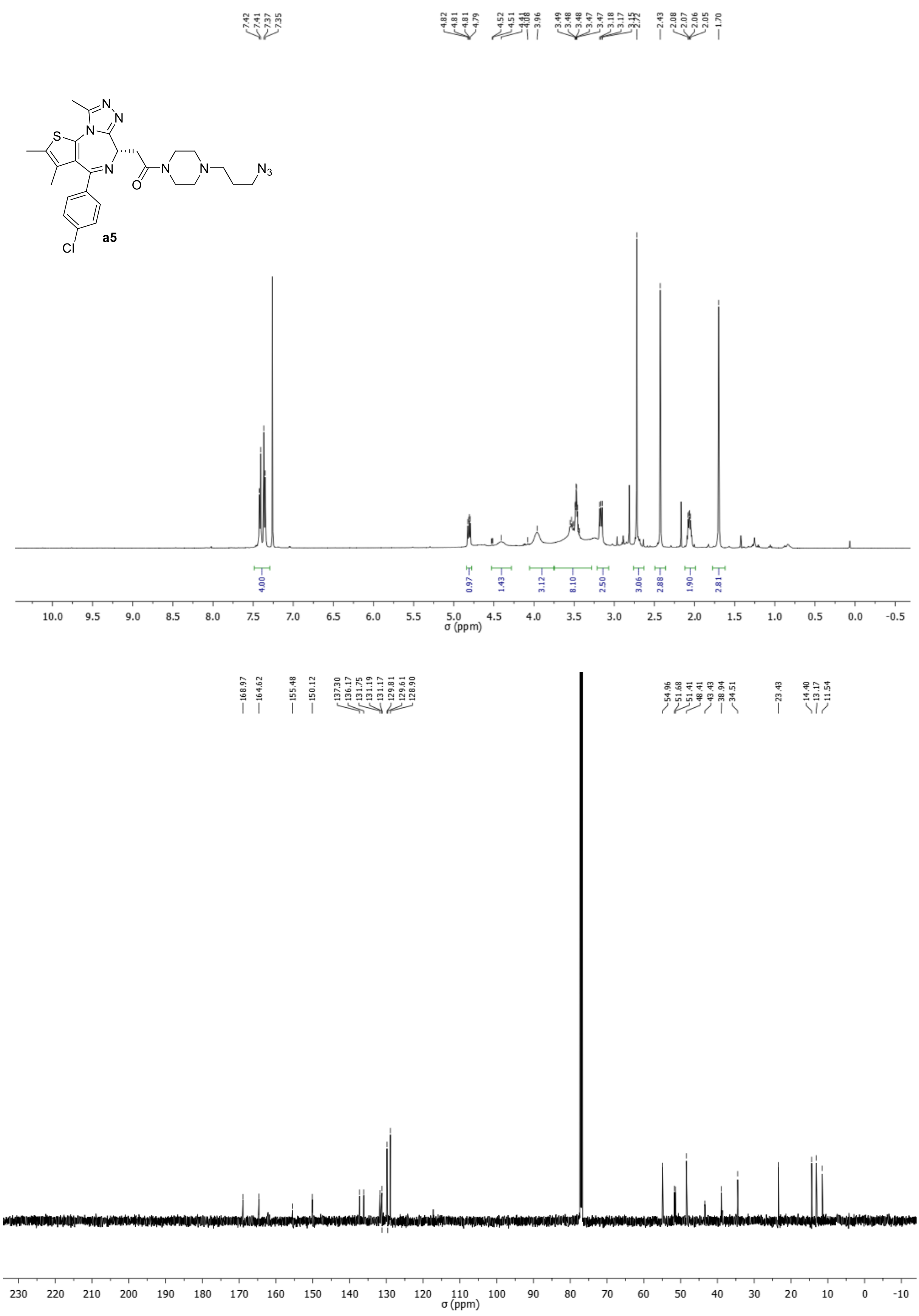

Supplementary Fig. 21

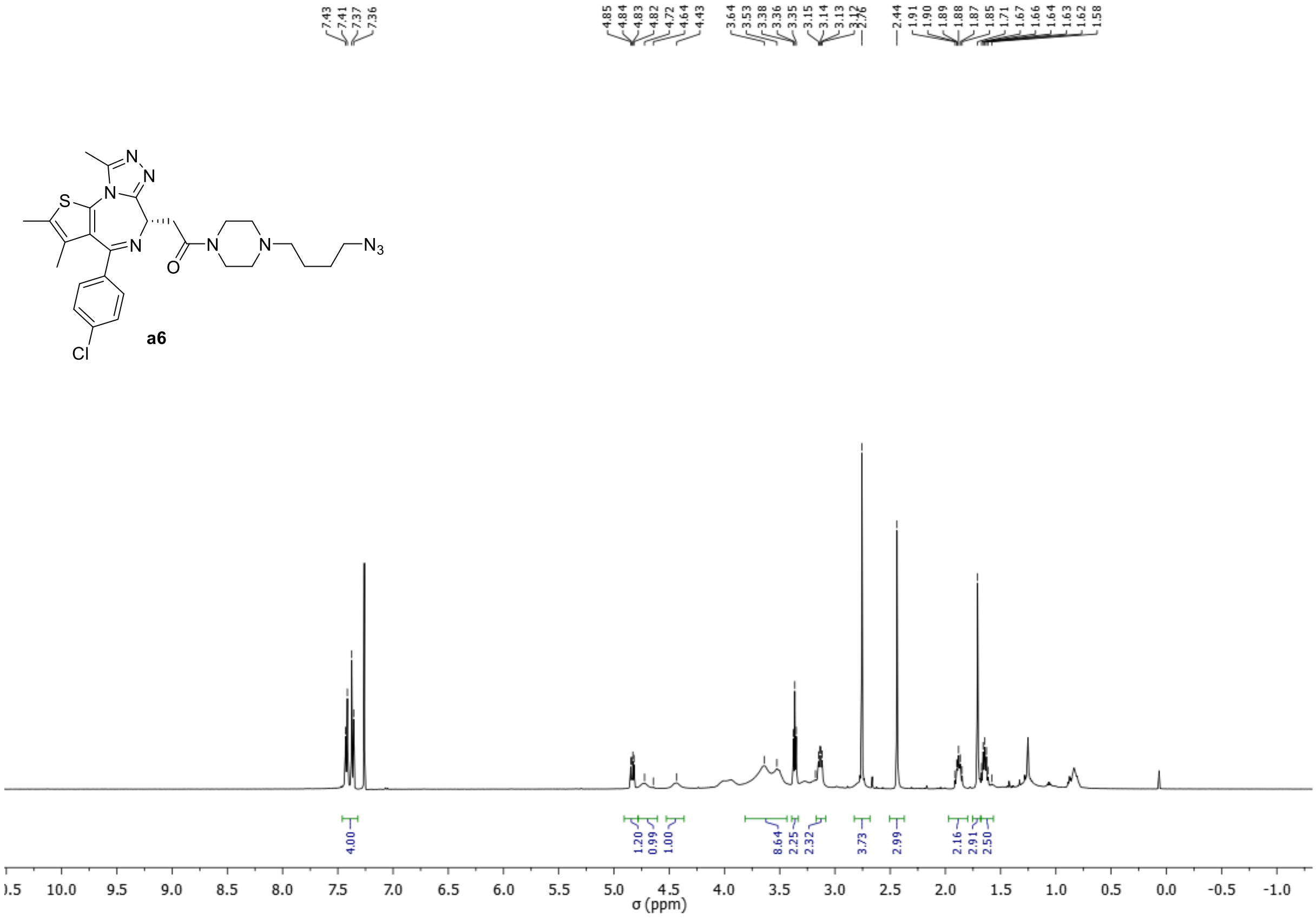

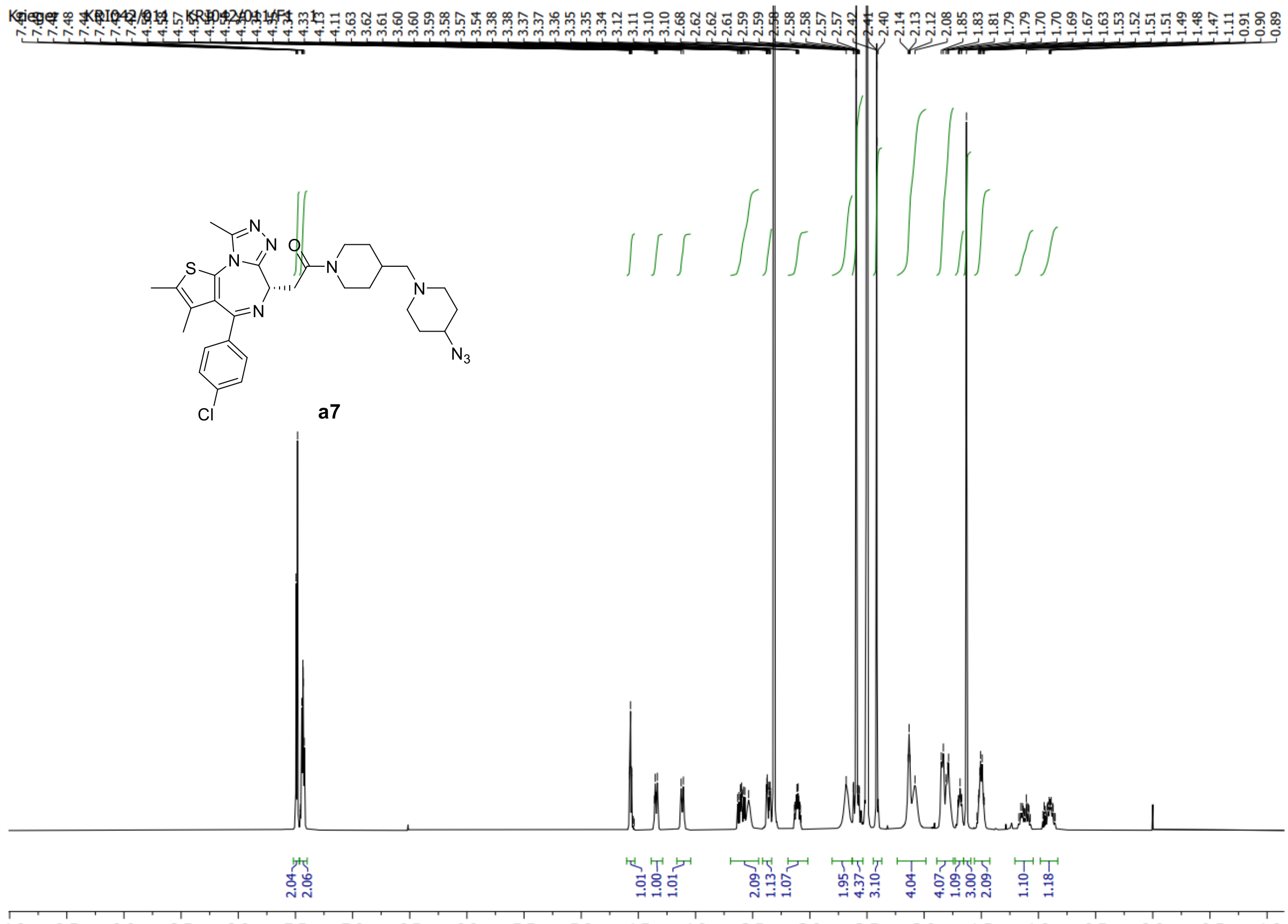

Supplementary Fig. 22

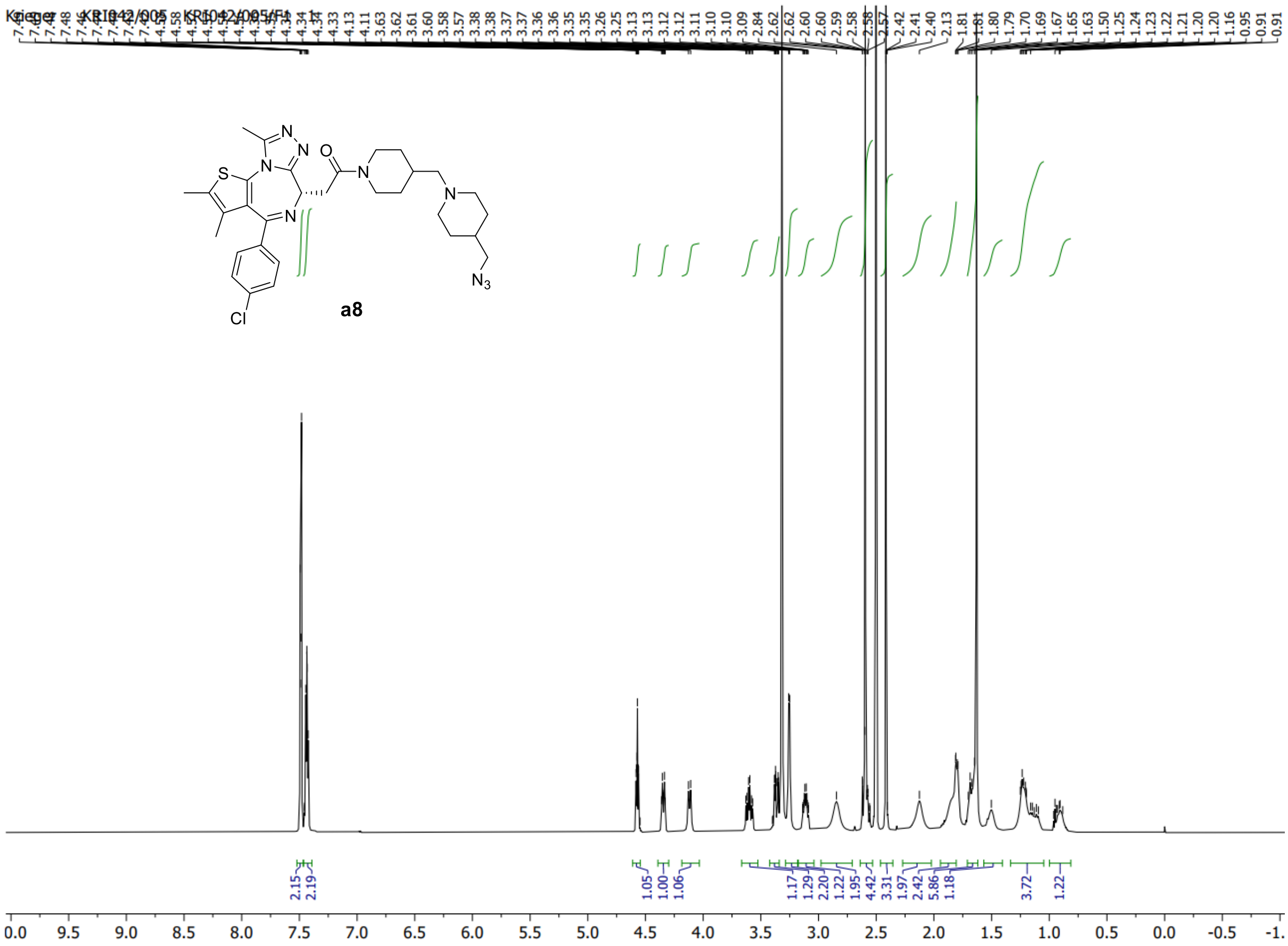

Supplementary Fig. 23

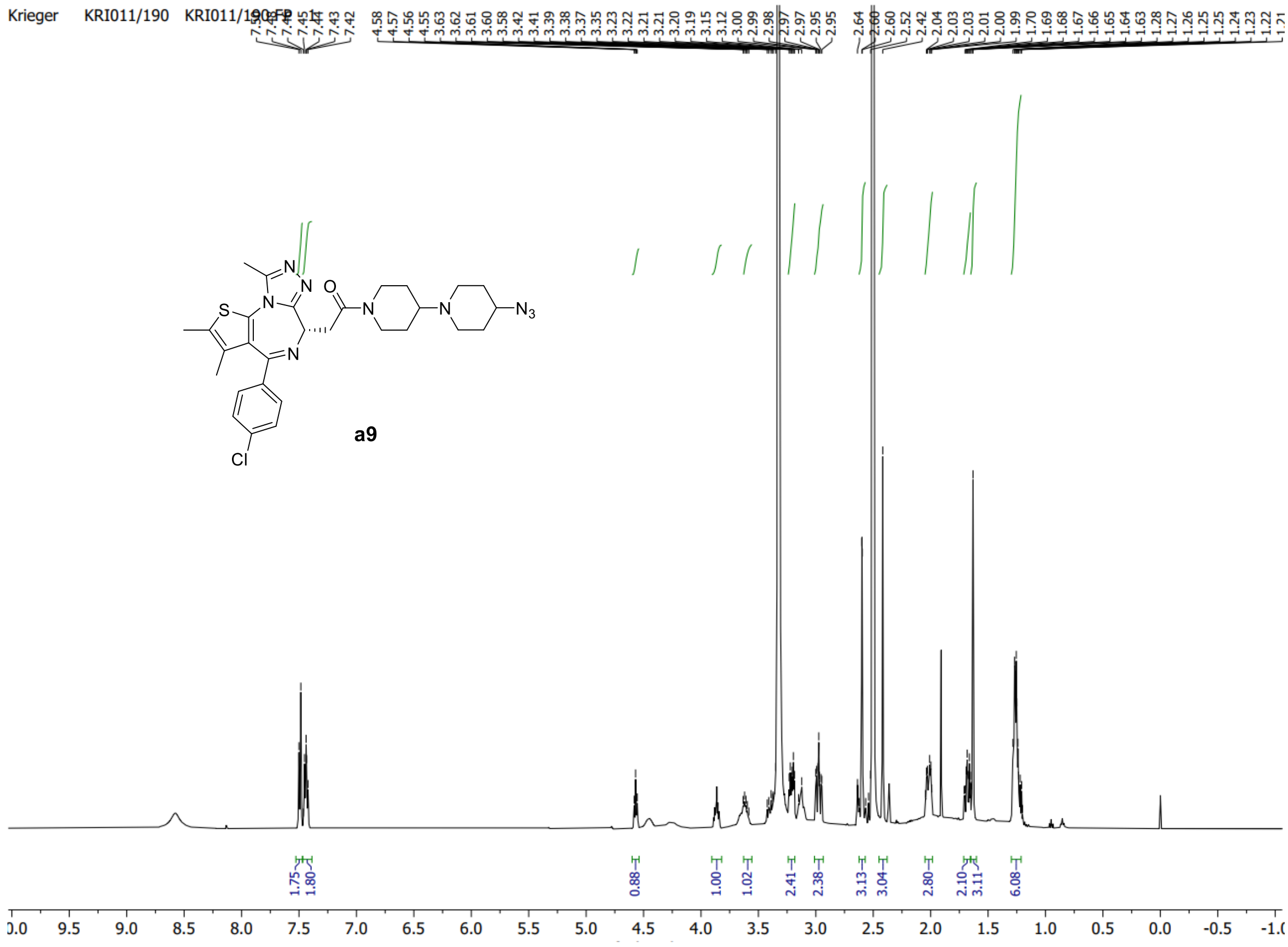

Supplementary Fig. 24

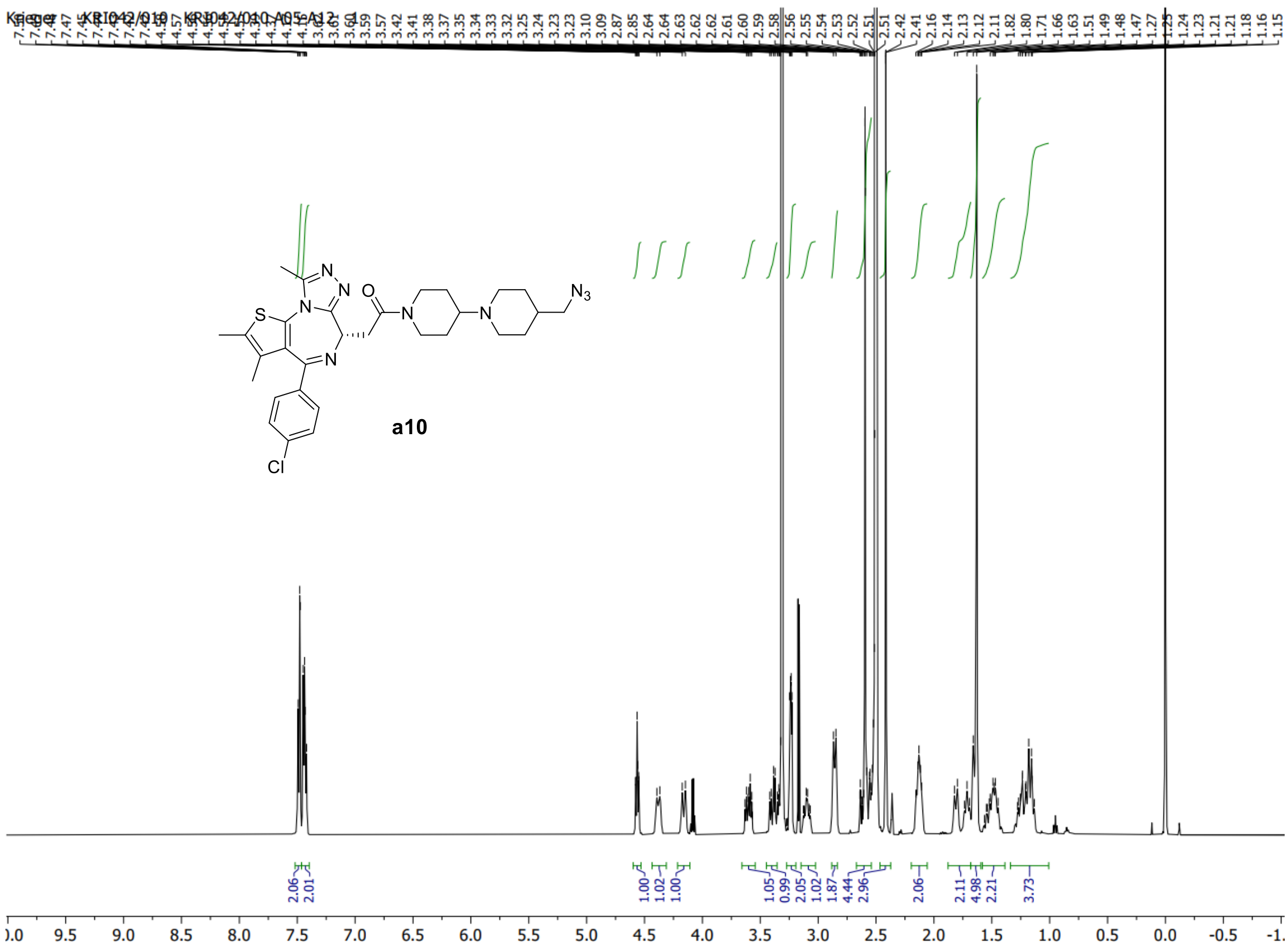

Supplementary Fig. 25

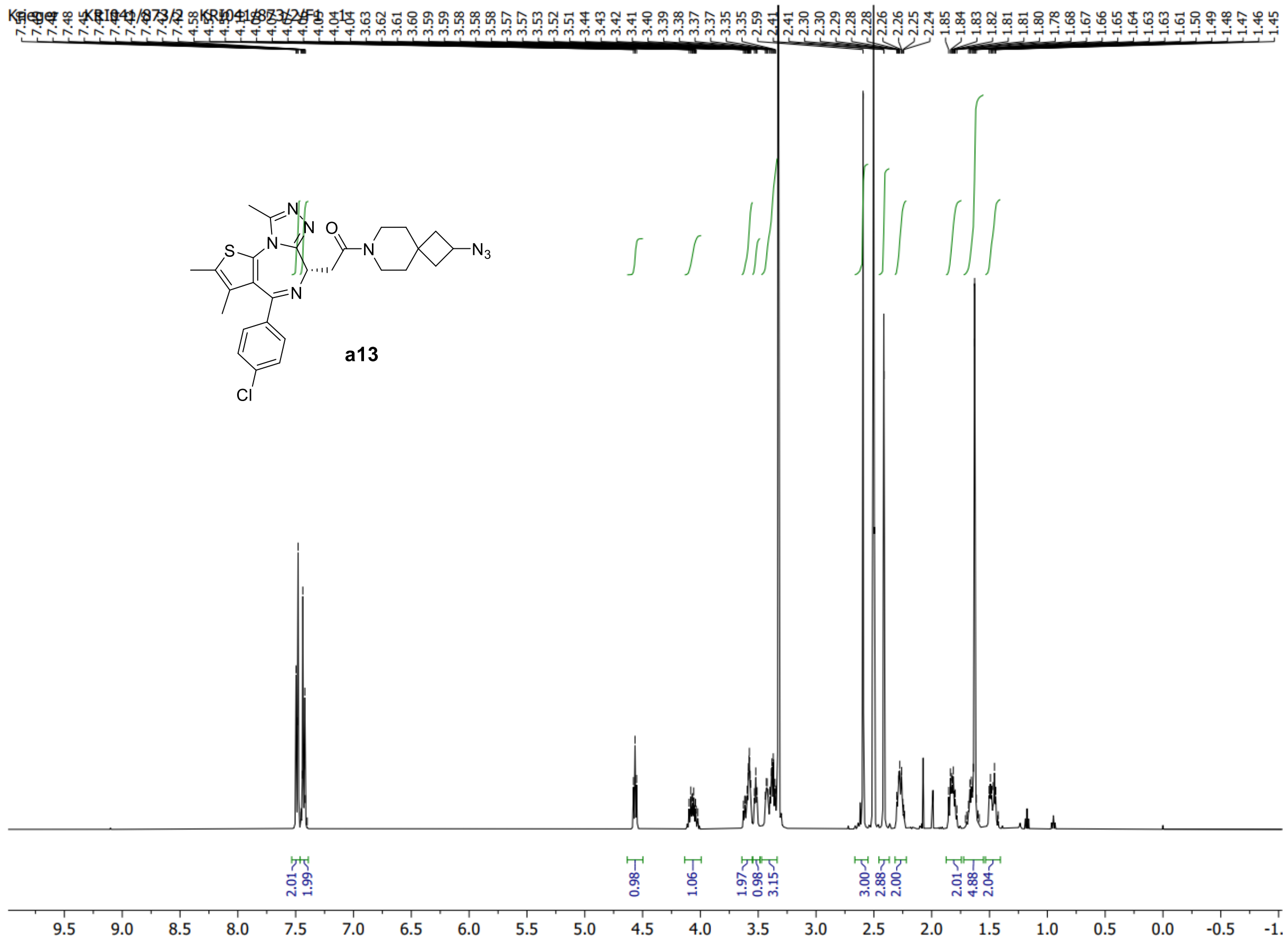

Supplementary Fig. 26

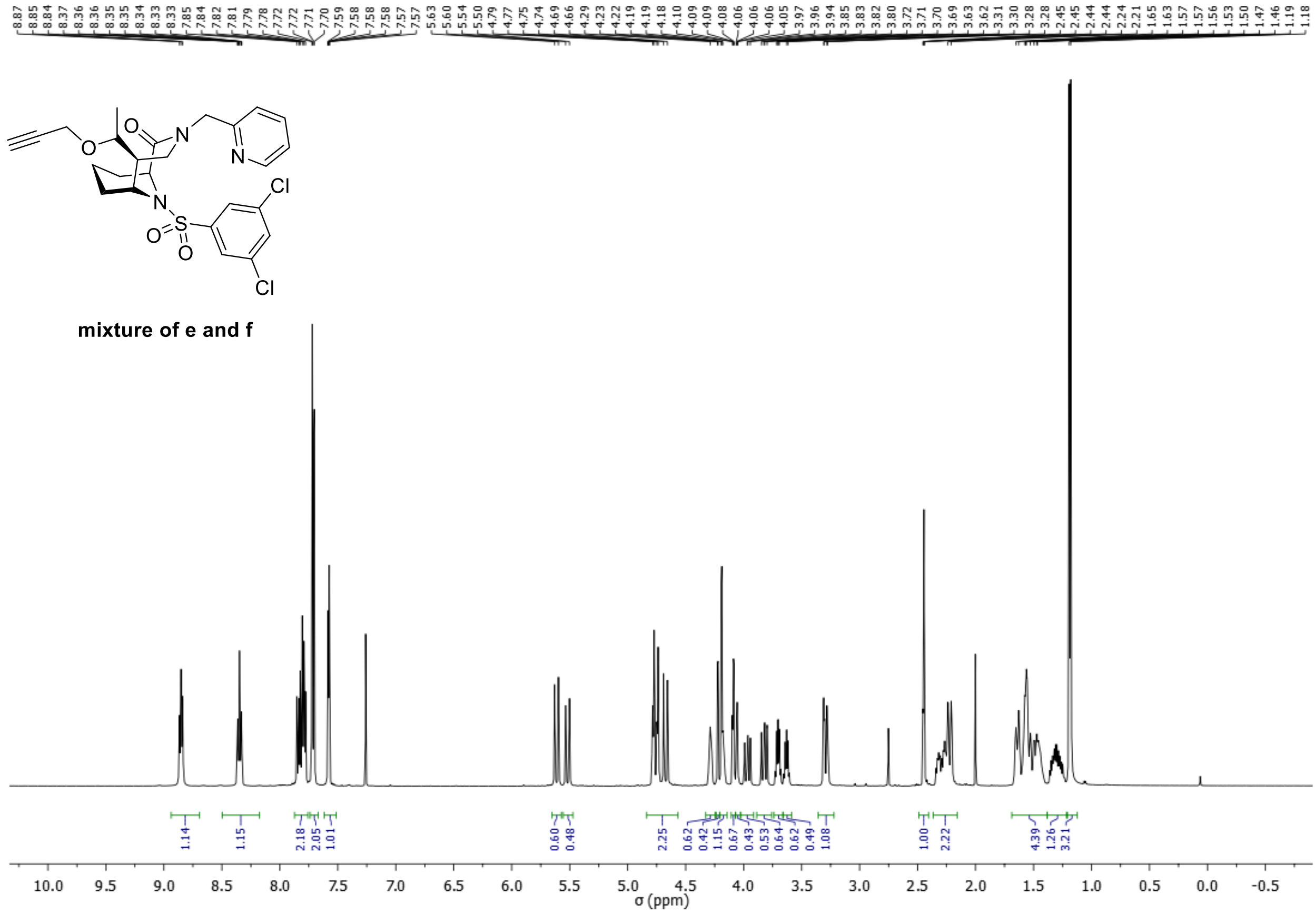

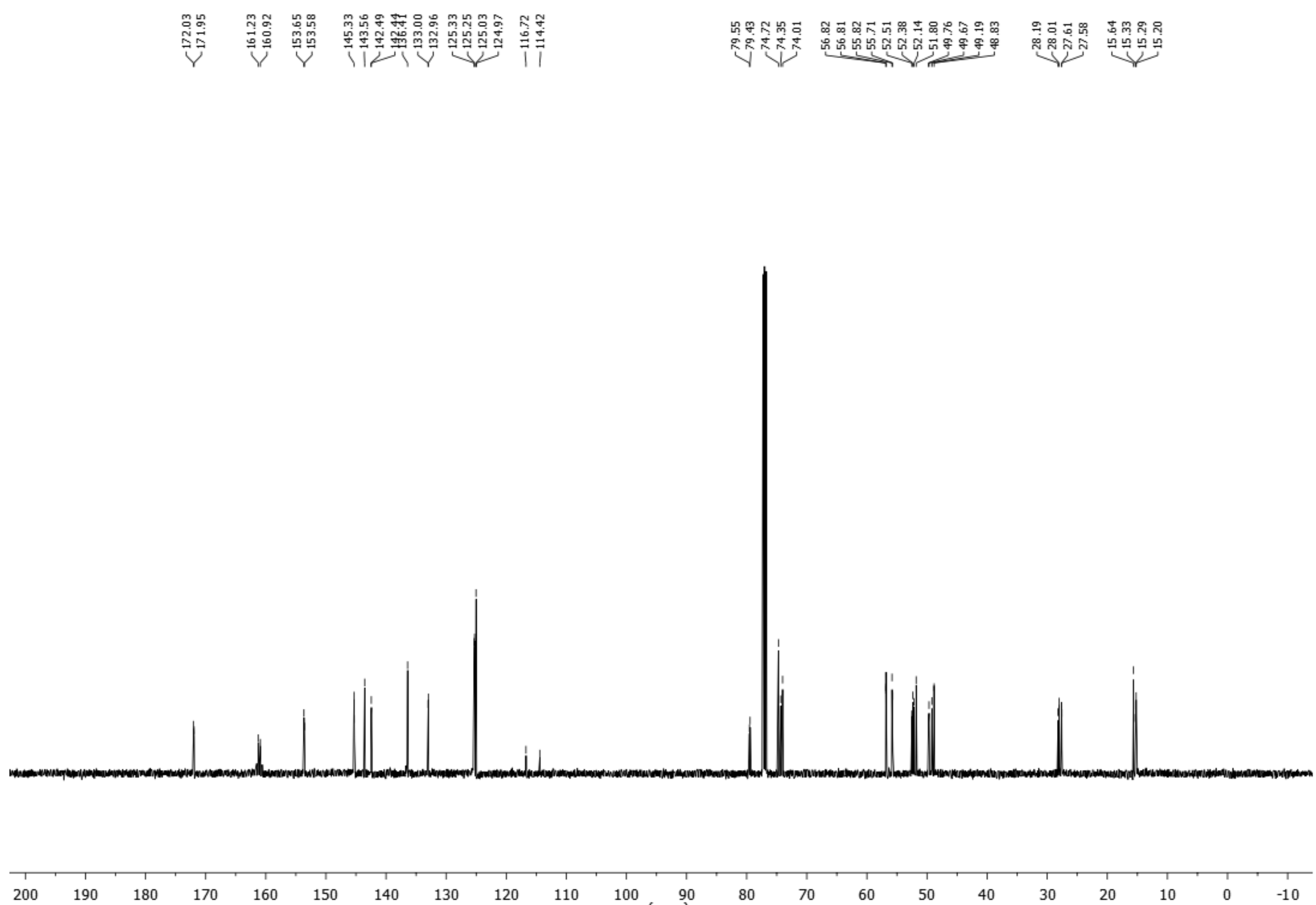

Supplementary Fig. 27

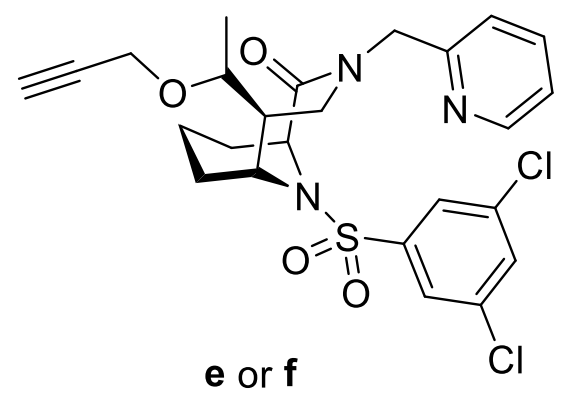

e or **f**

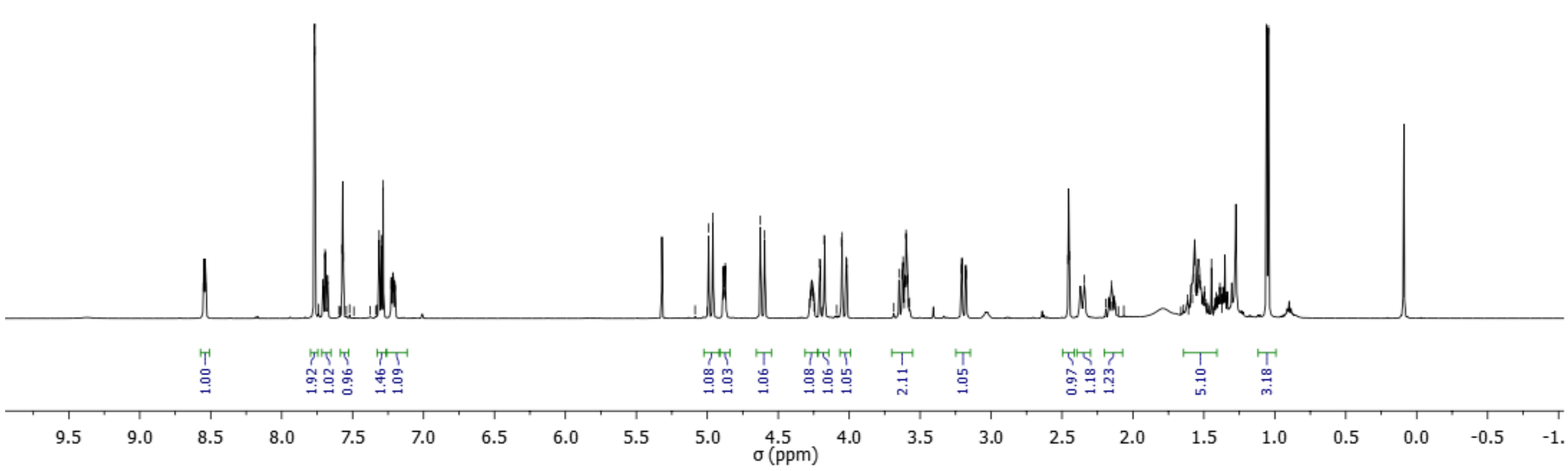

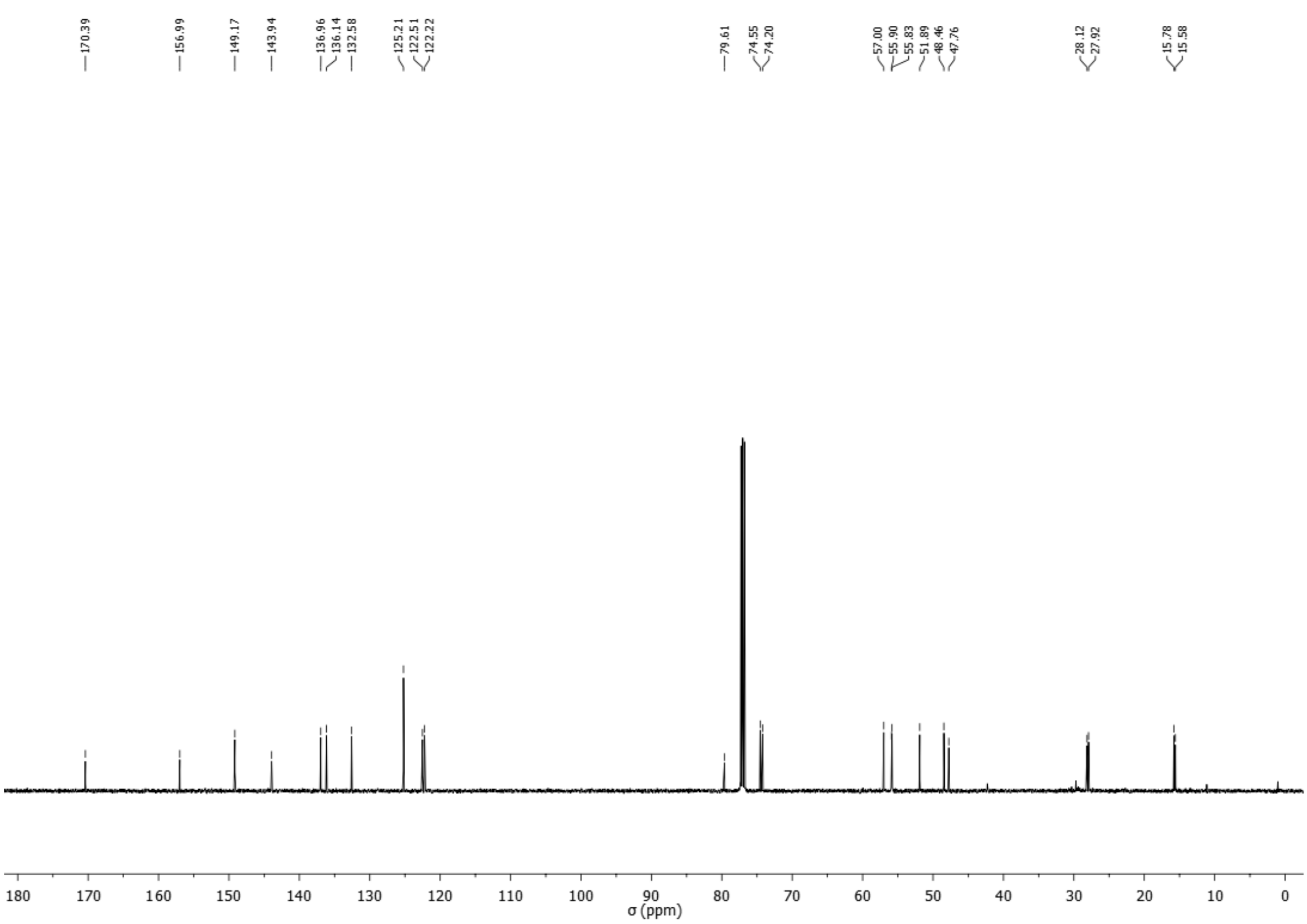

Supplementary Fig. 28

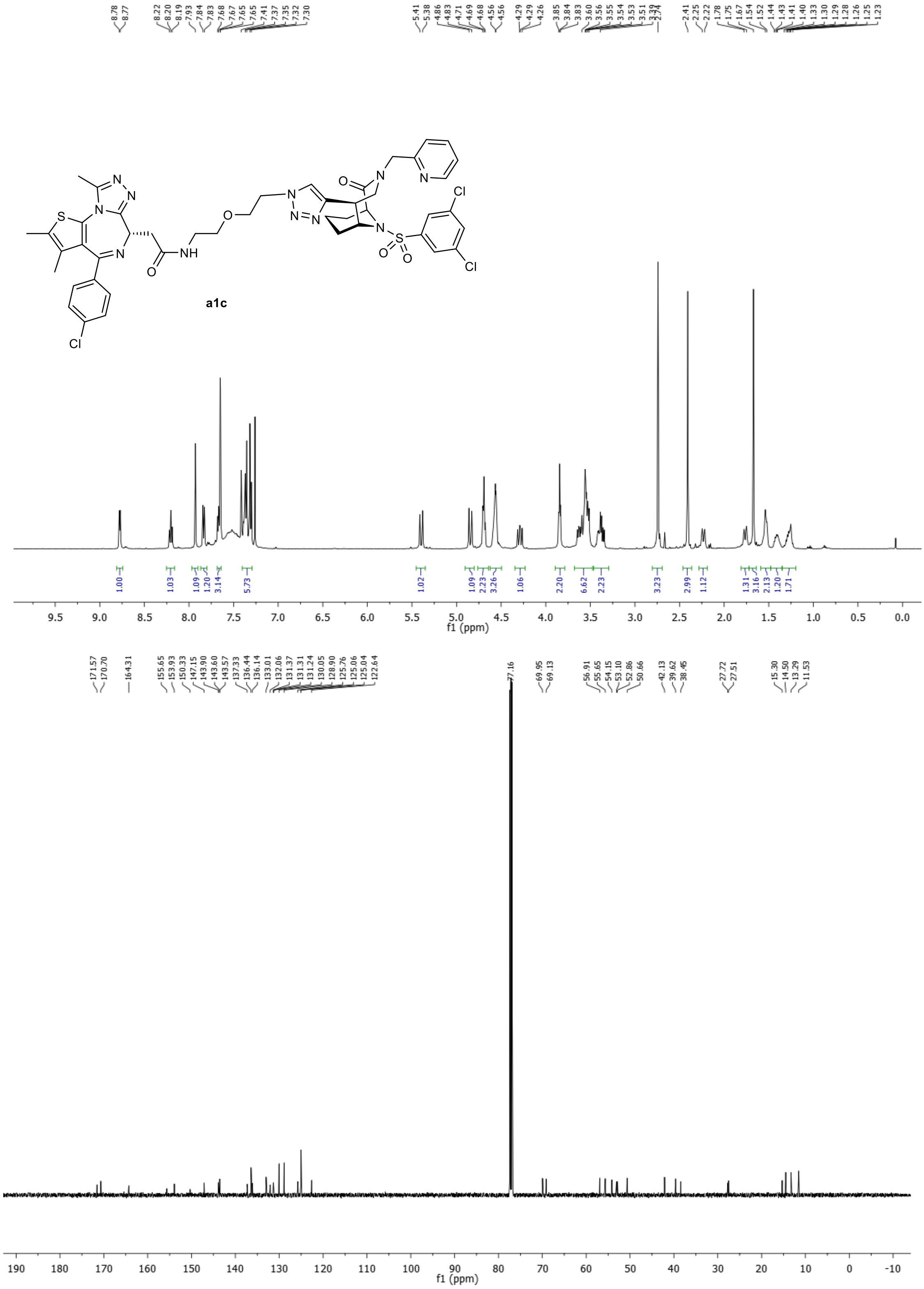

Supplementary Fig. 29

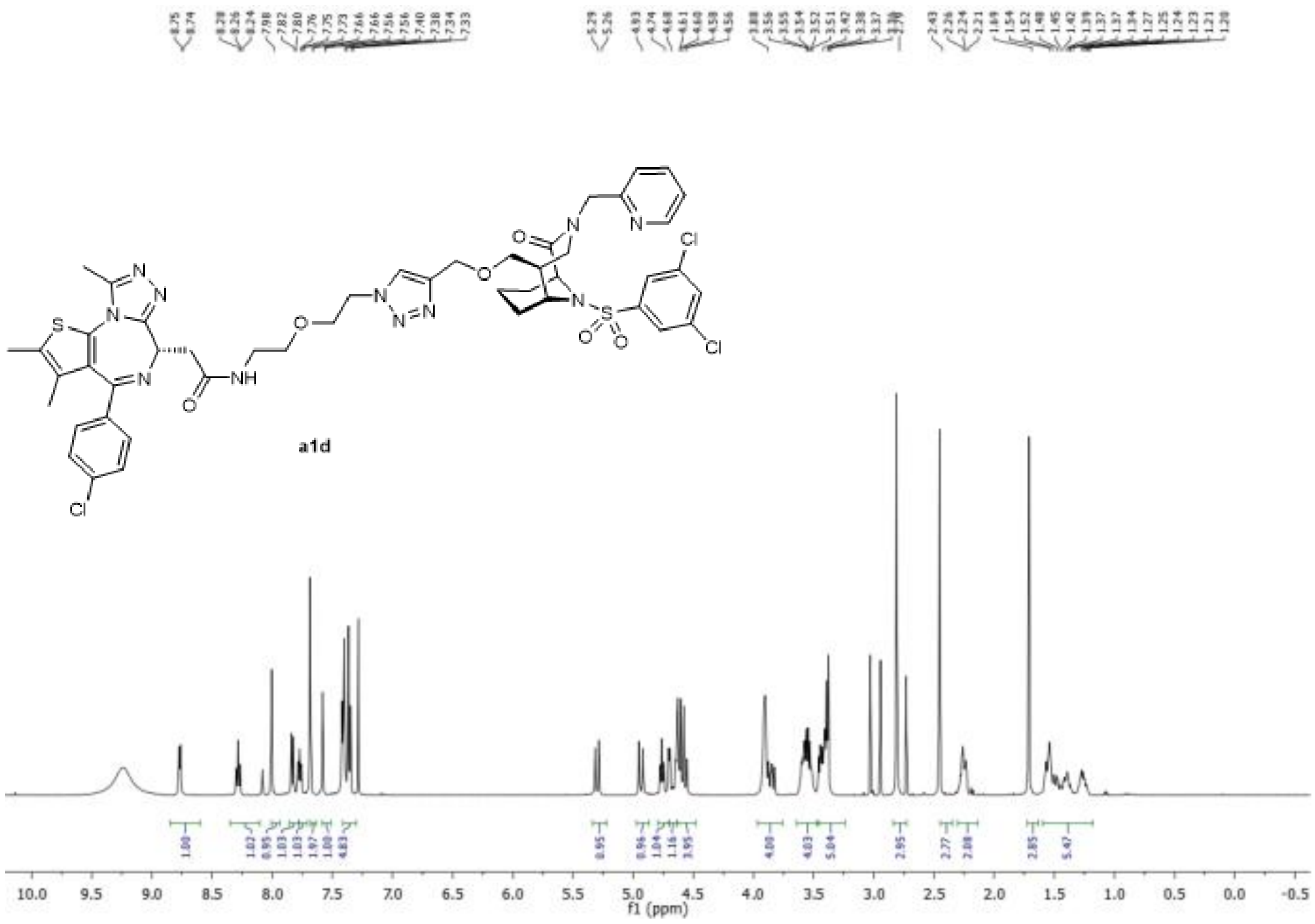

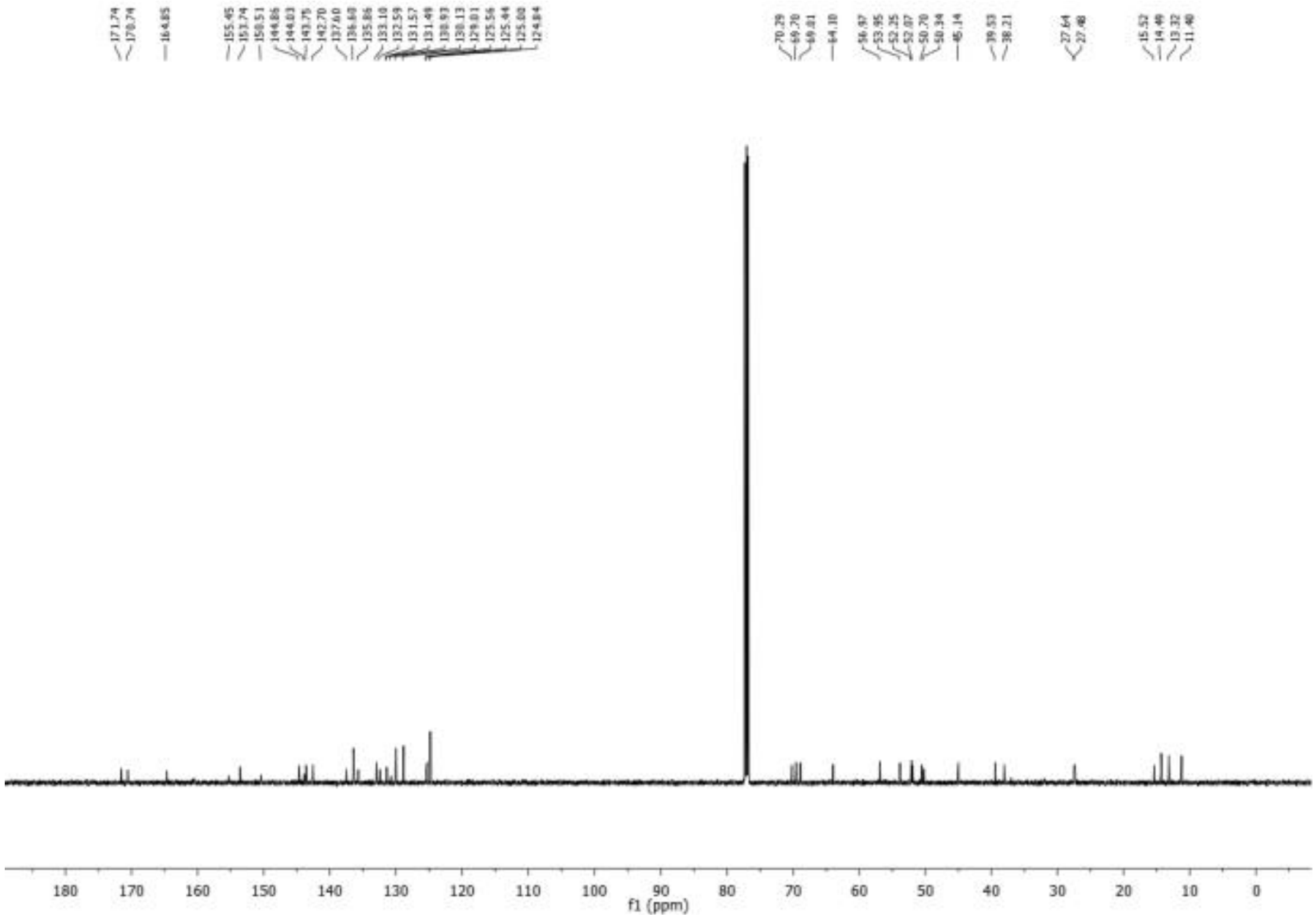

Supplementary Fig. 30

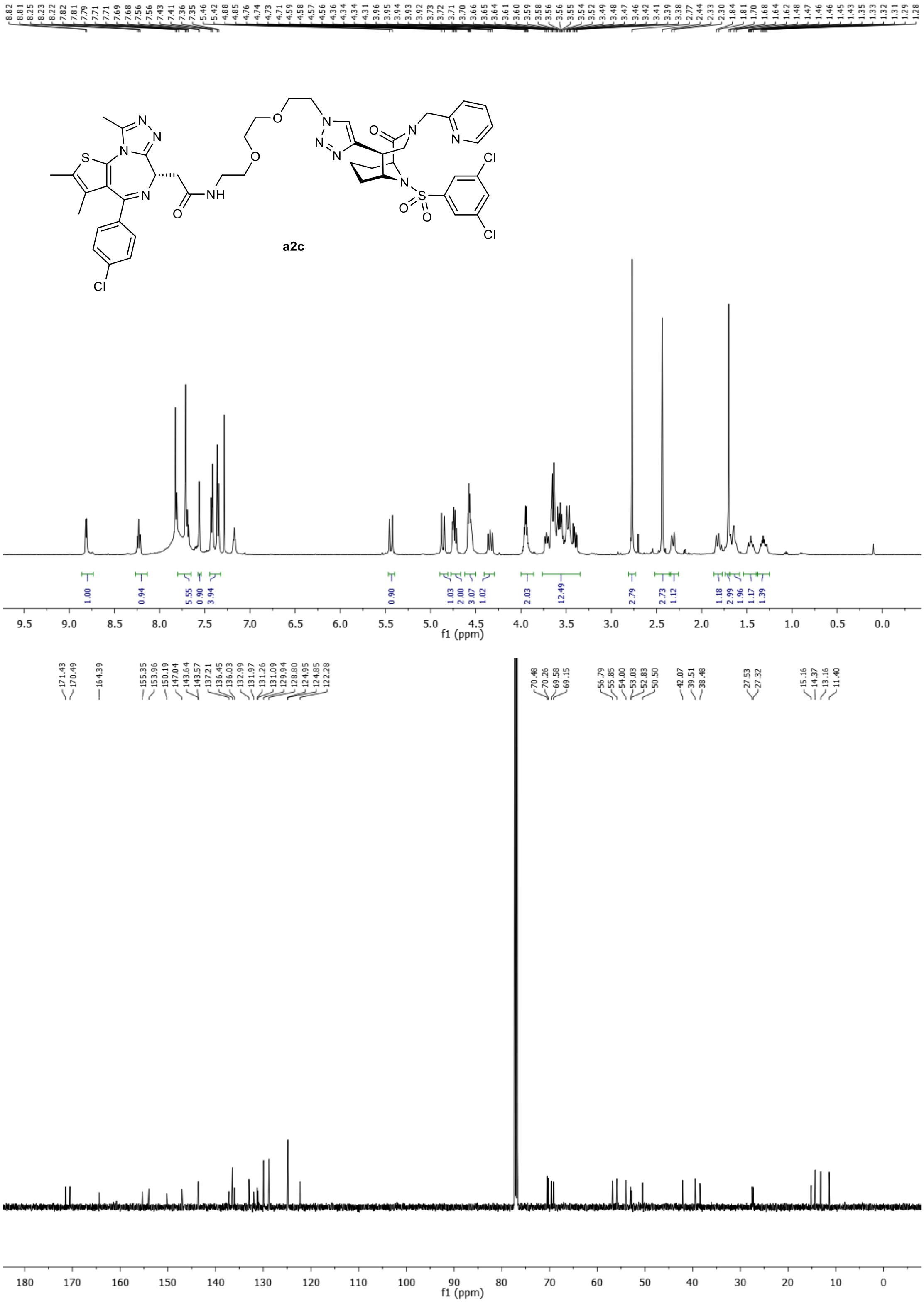

Supplementary Fig. 31

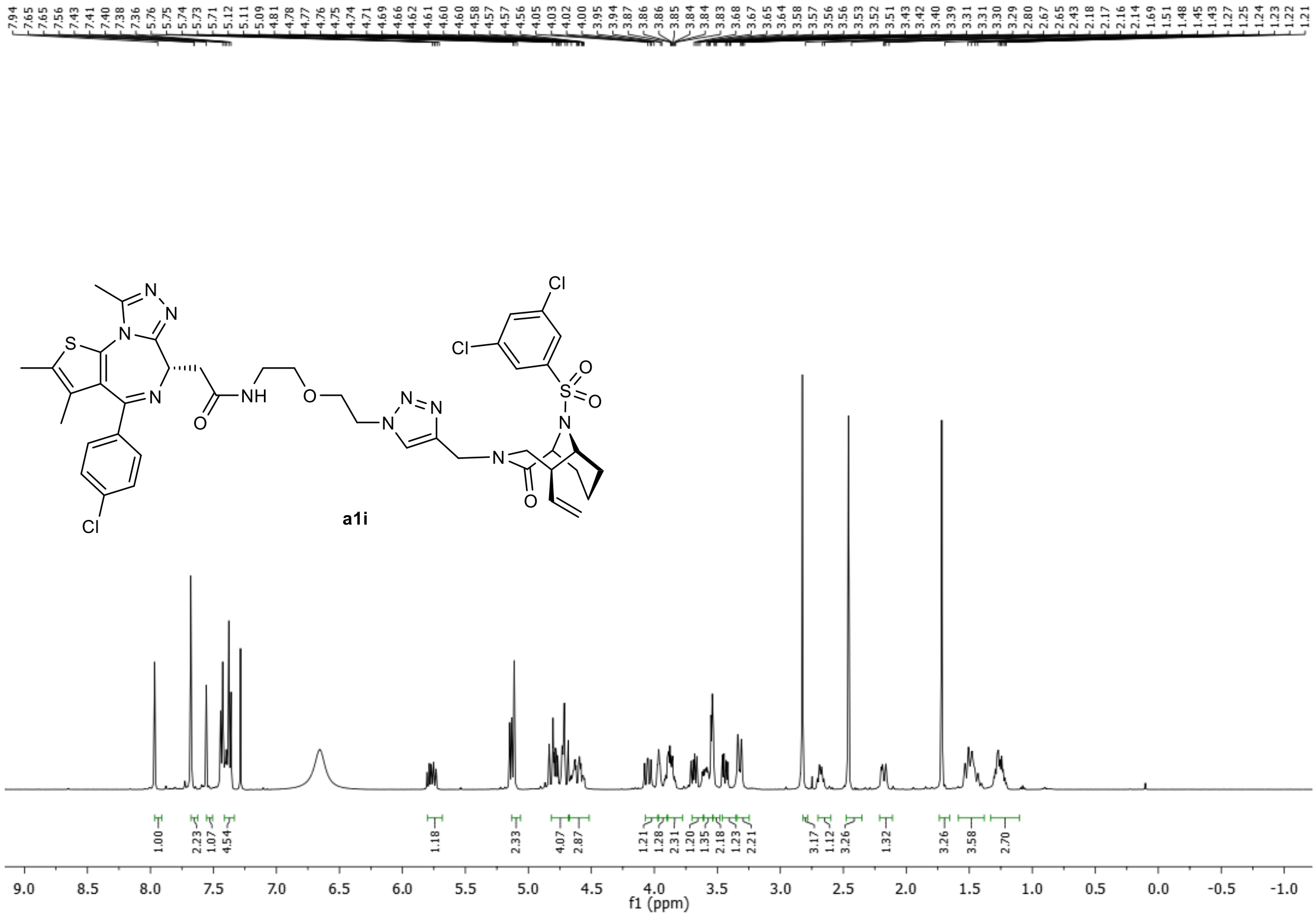

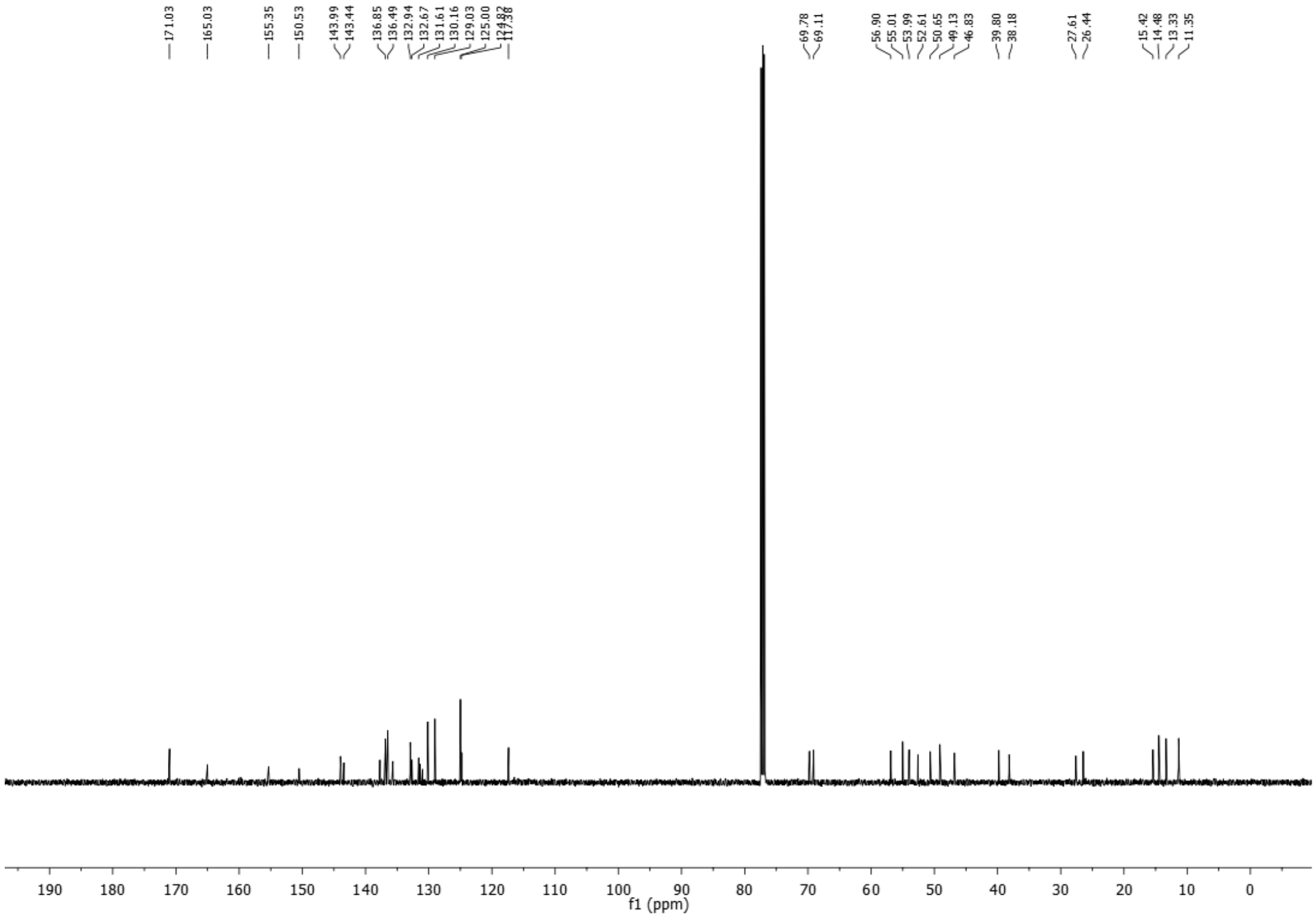

Supplementary Fig. 32

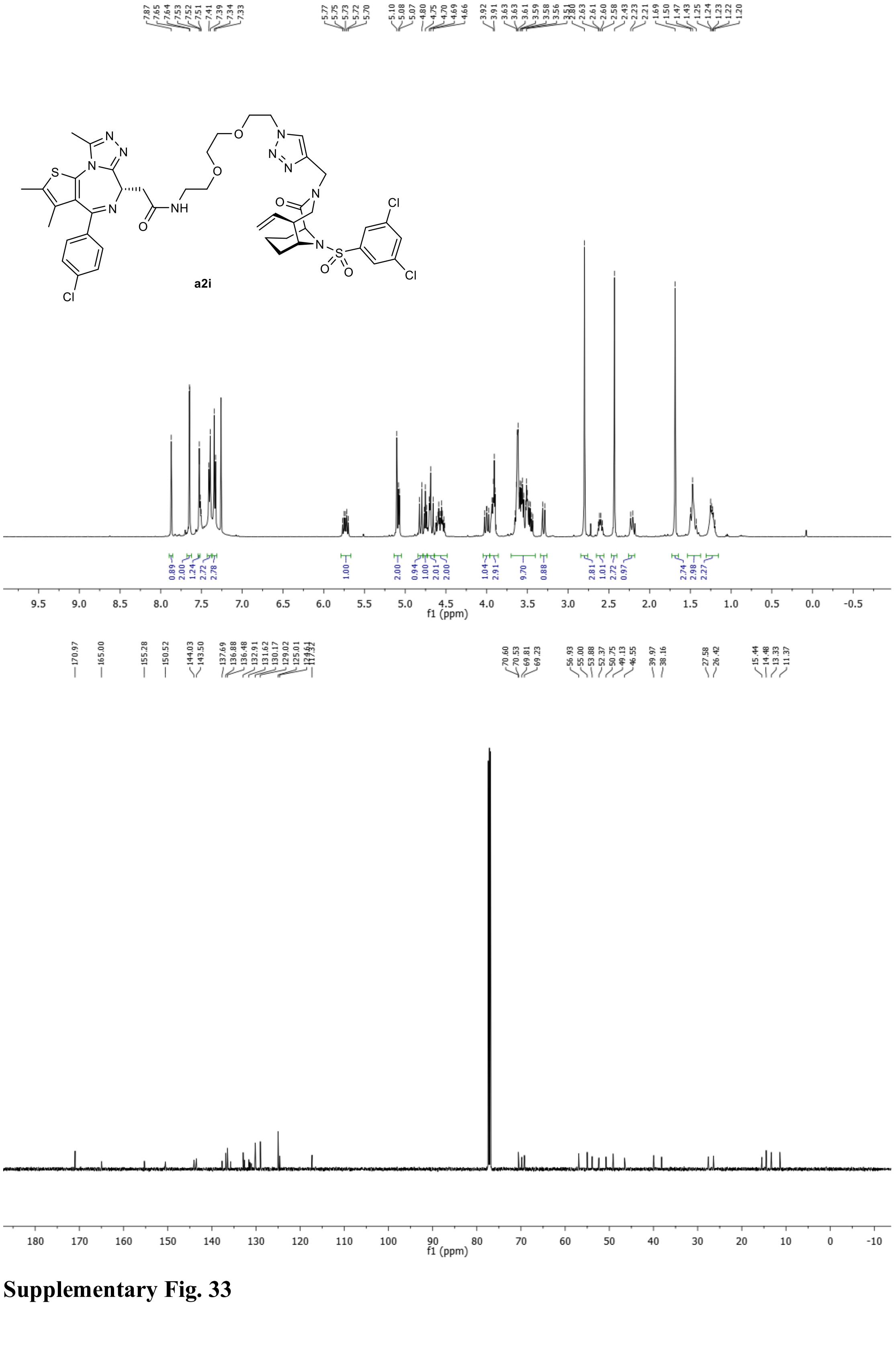

Supplementary Fig. 33

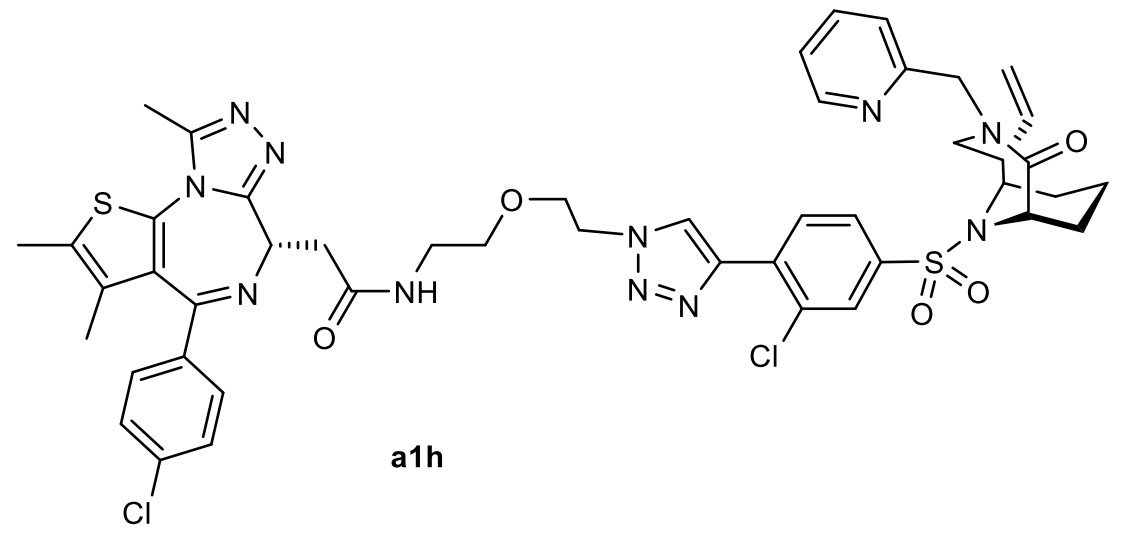

a1h

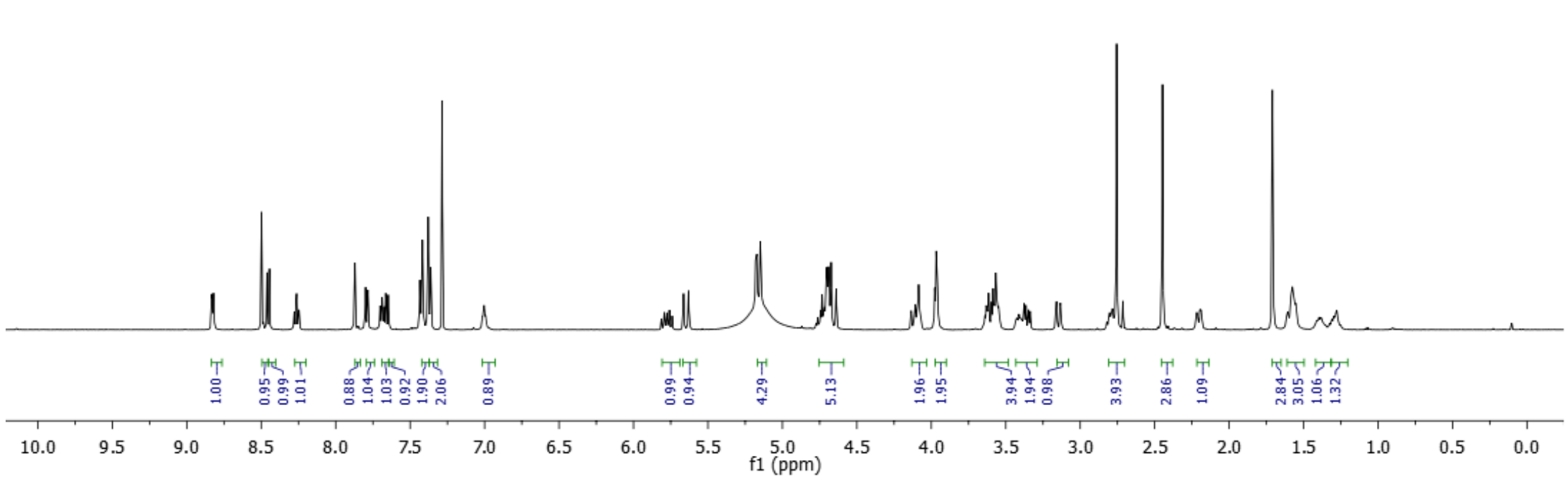

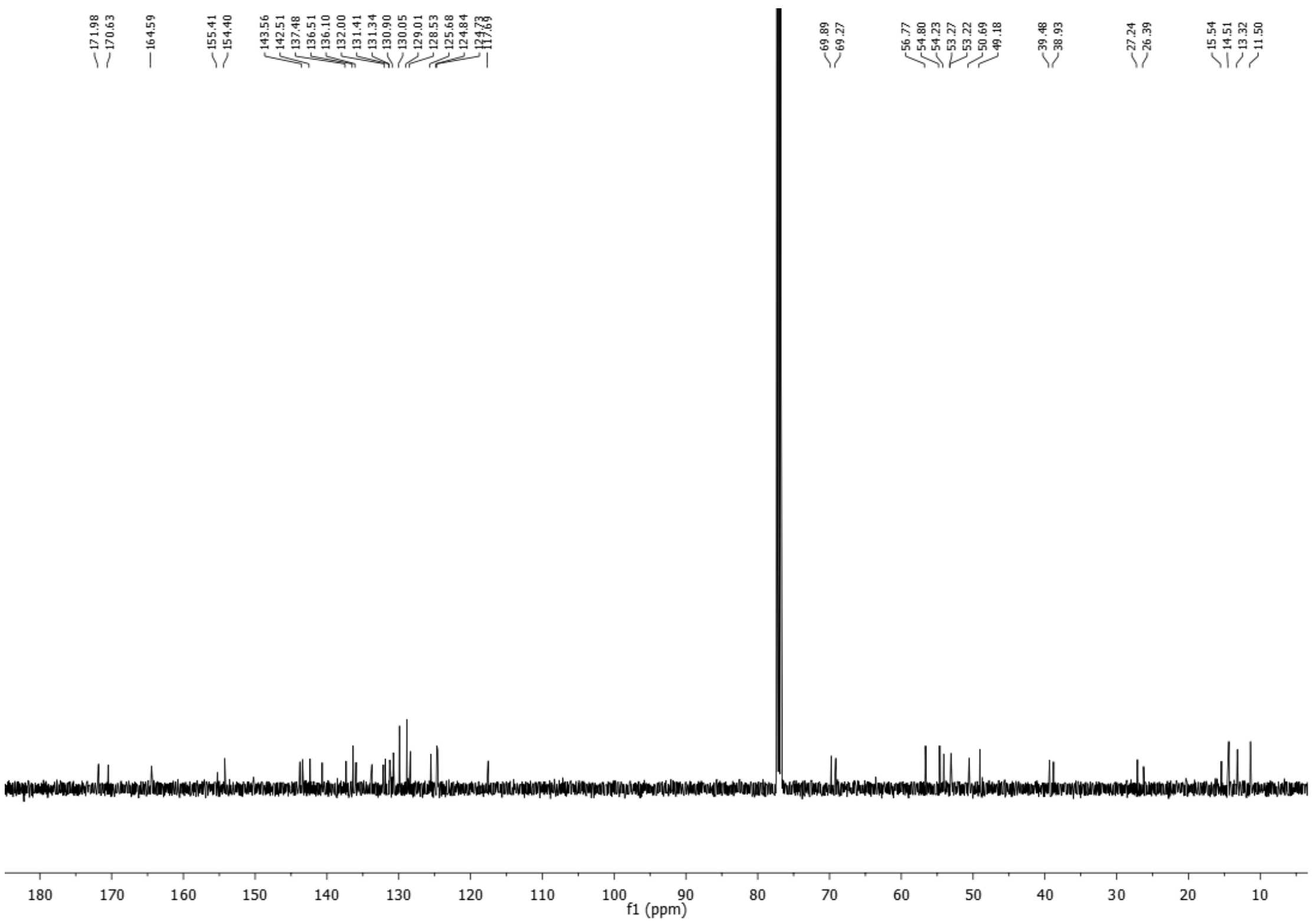

Supplementary Fig. 34

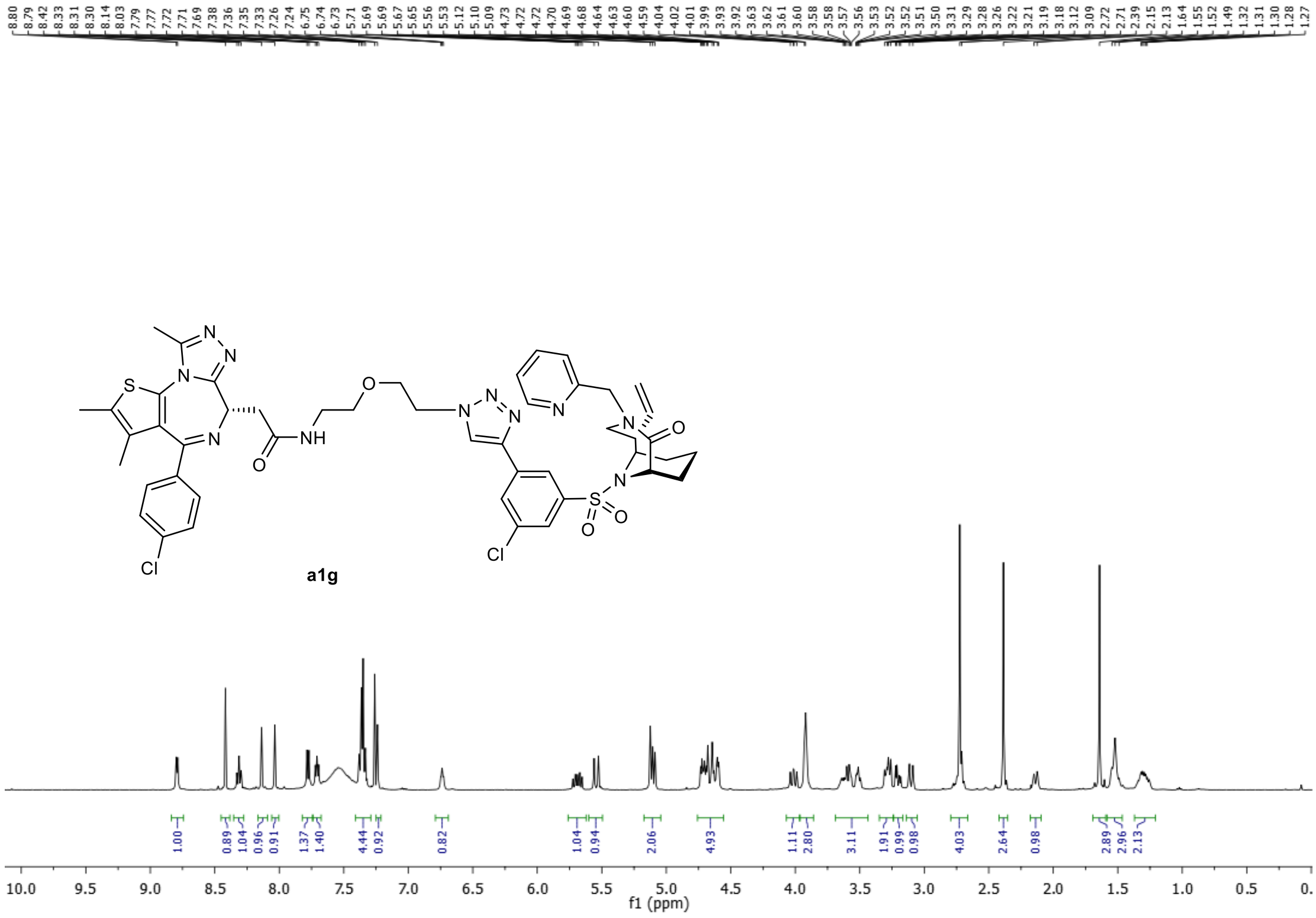

a1g

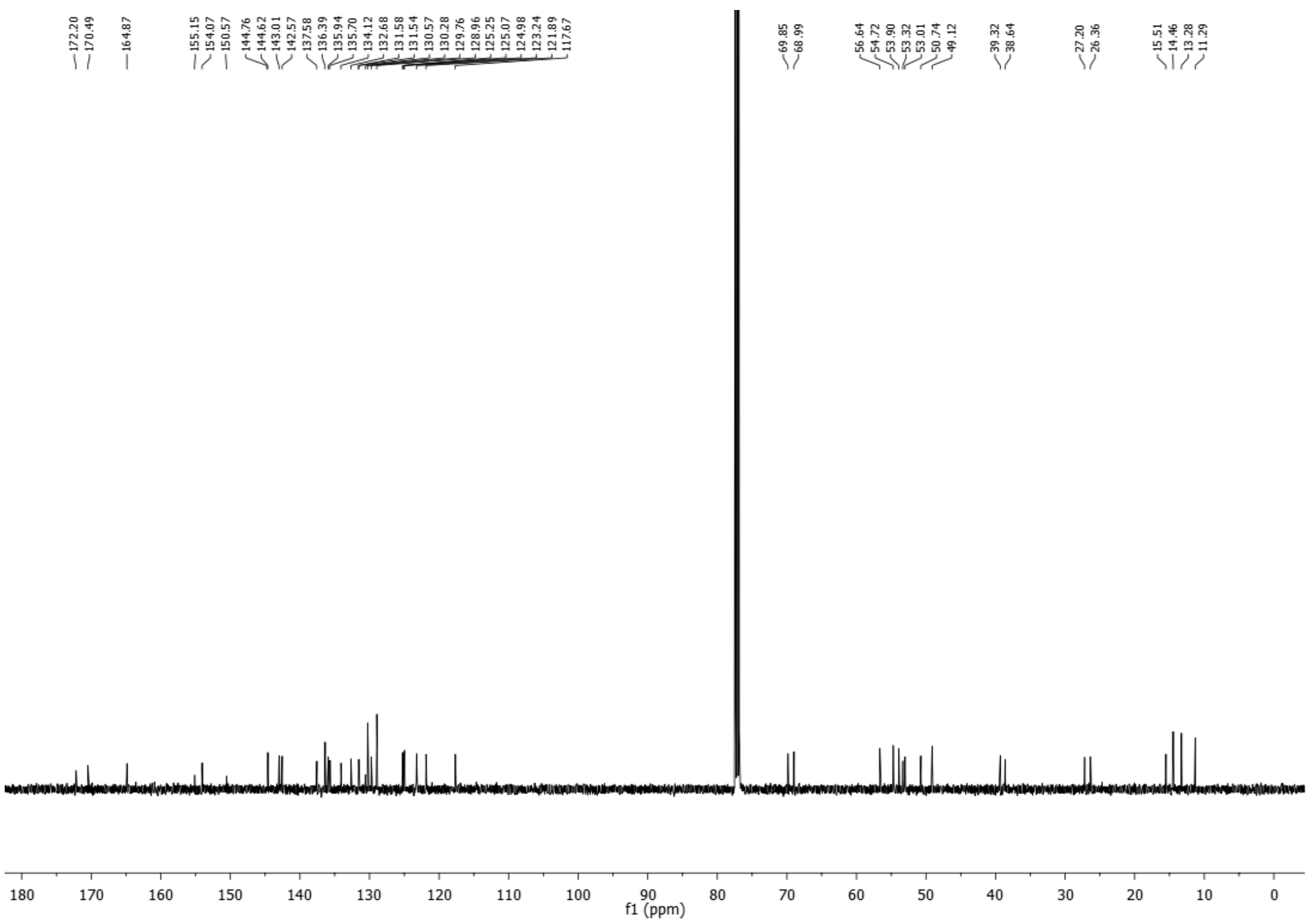

Supplementary Fig. 35

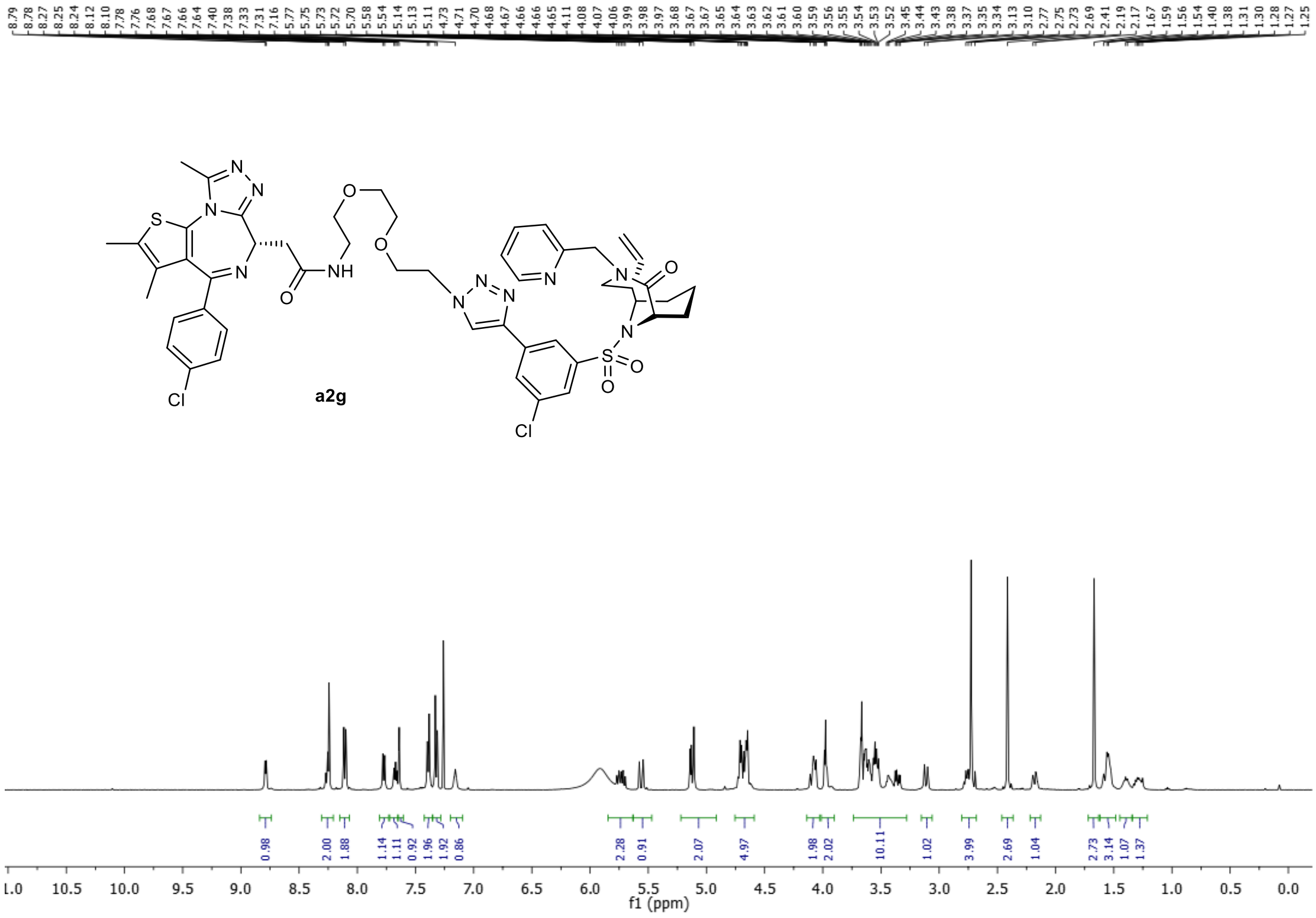

Supplementary Fig. 36

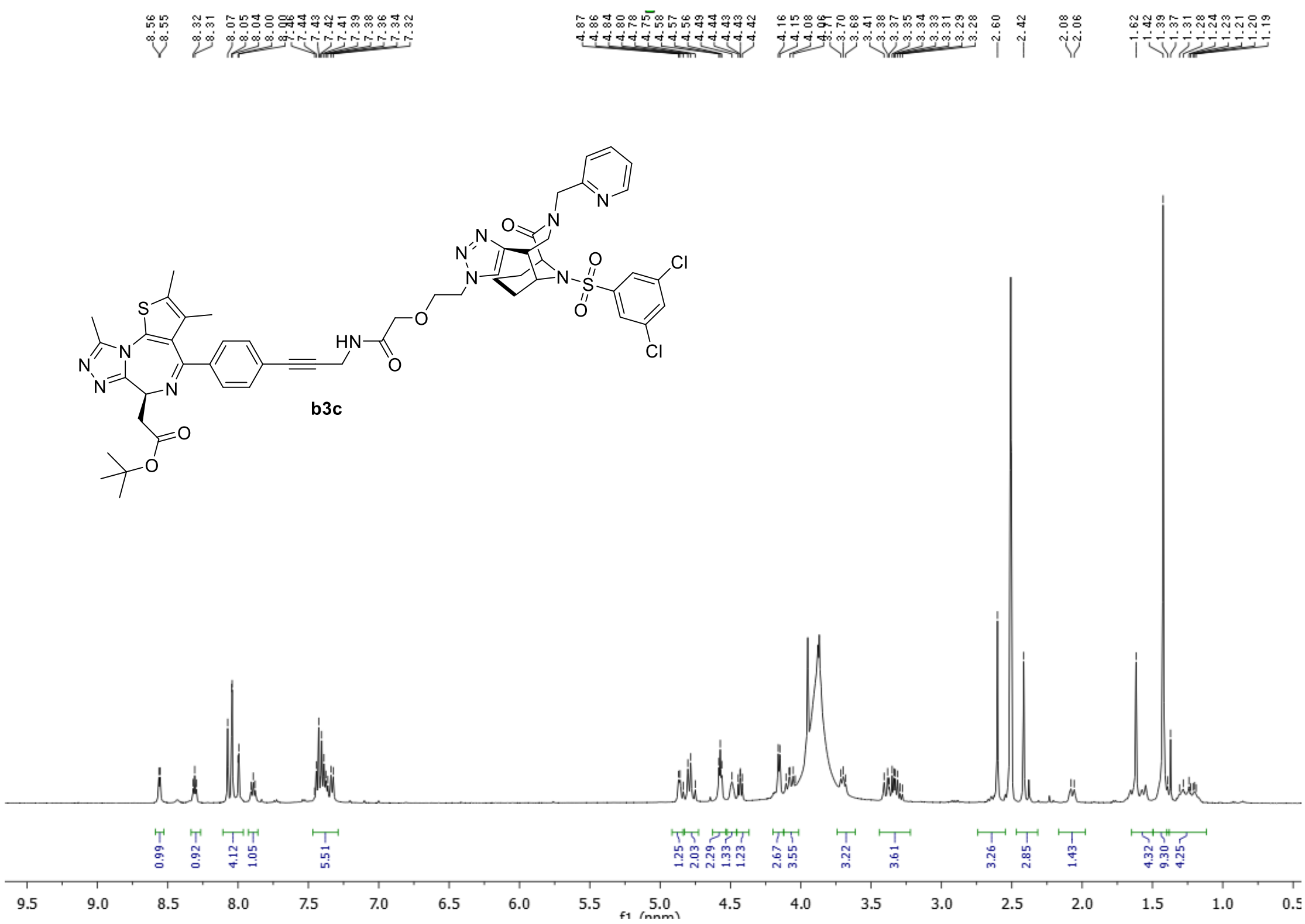

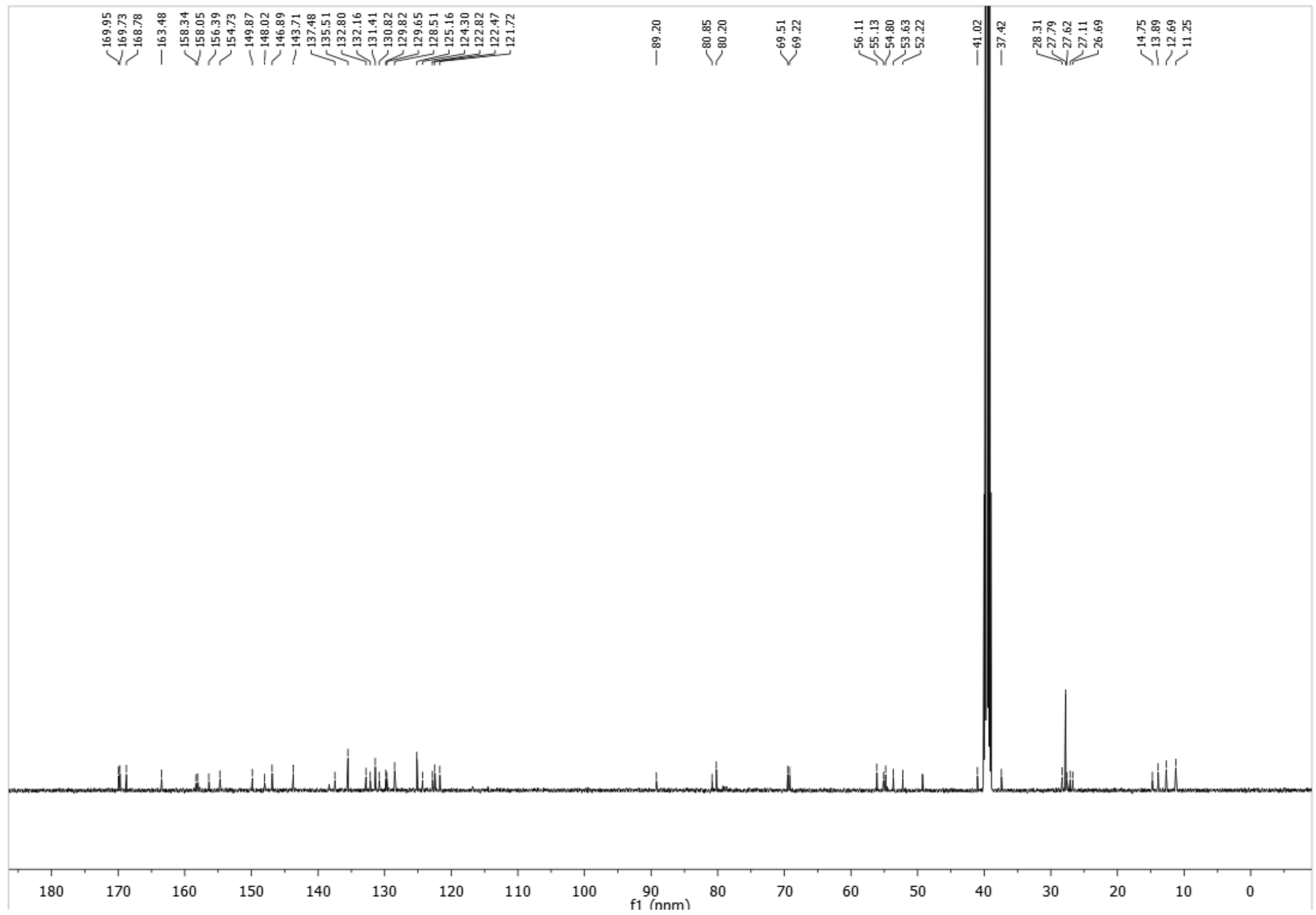

Supplementary Fig. 37

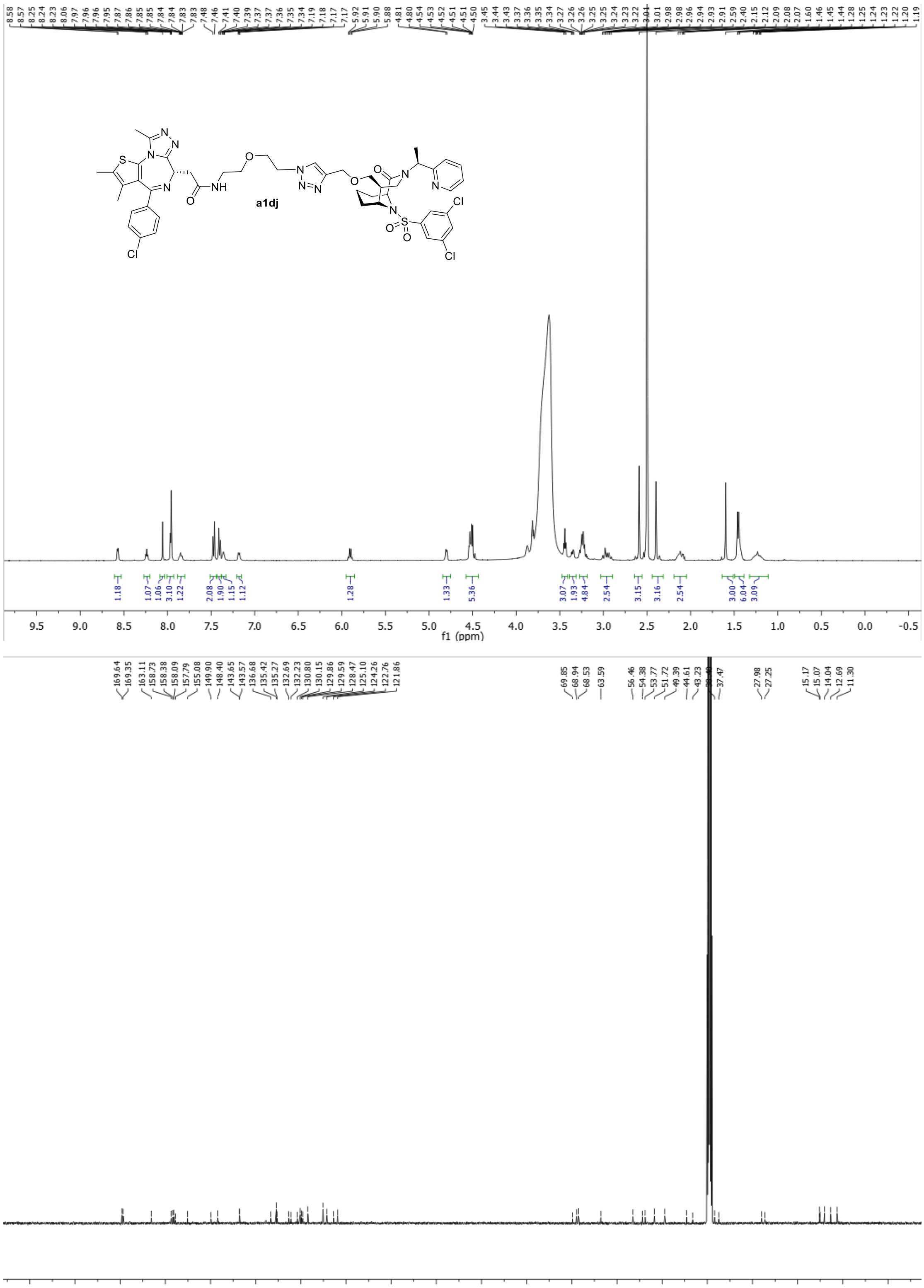

Supplementary Fig. 38

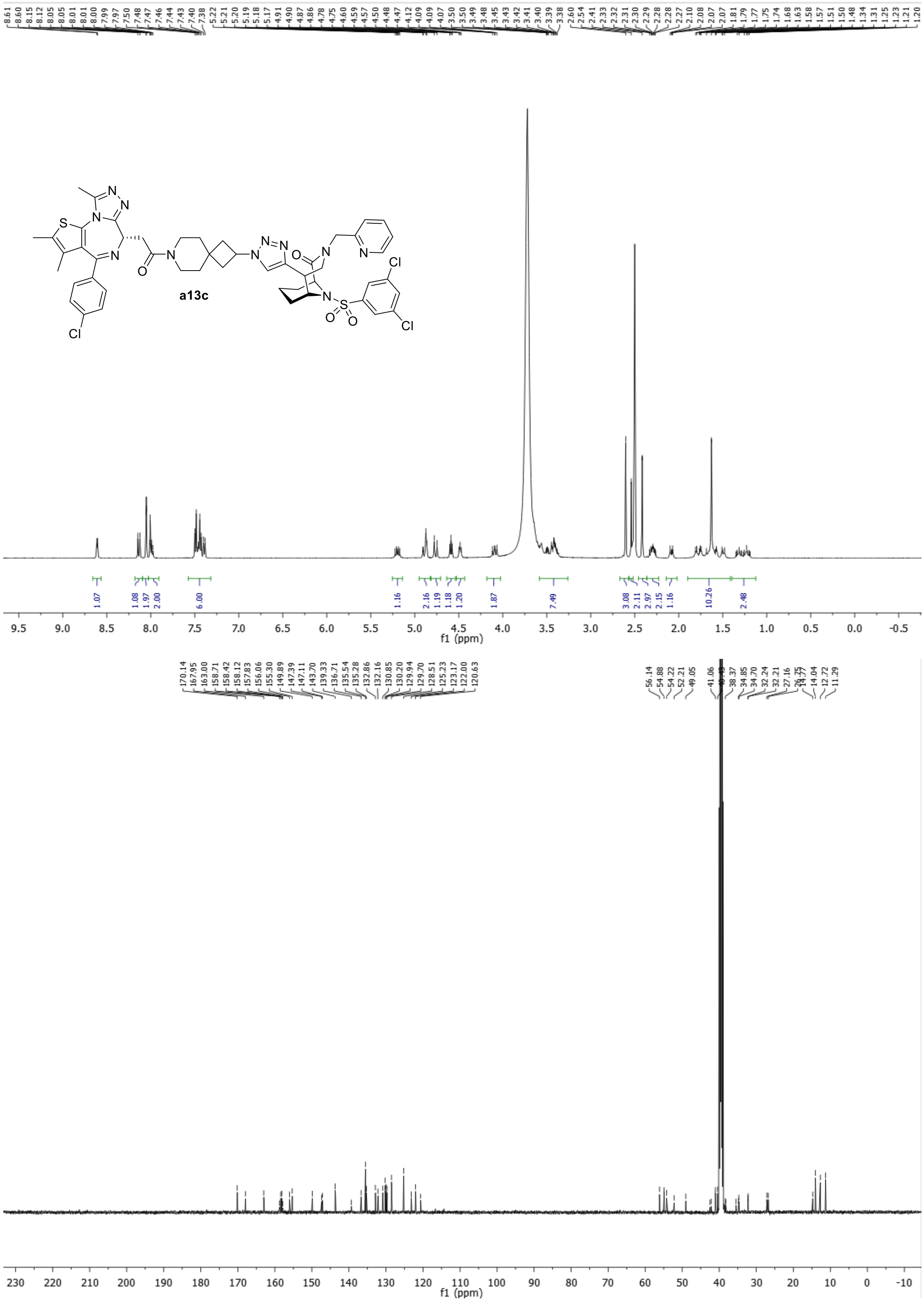

Supplementary Fig. 39

LC-MS analytics of the final heterobifunctionals

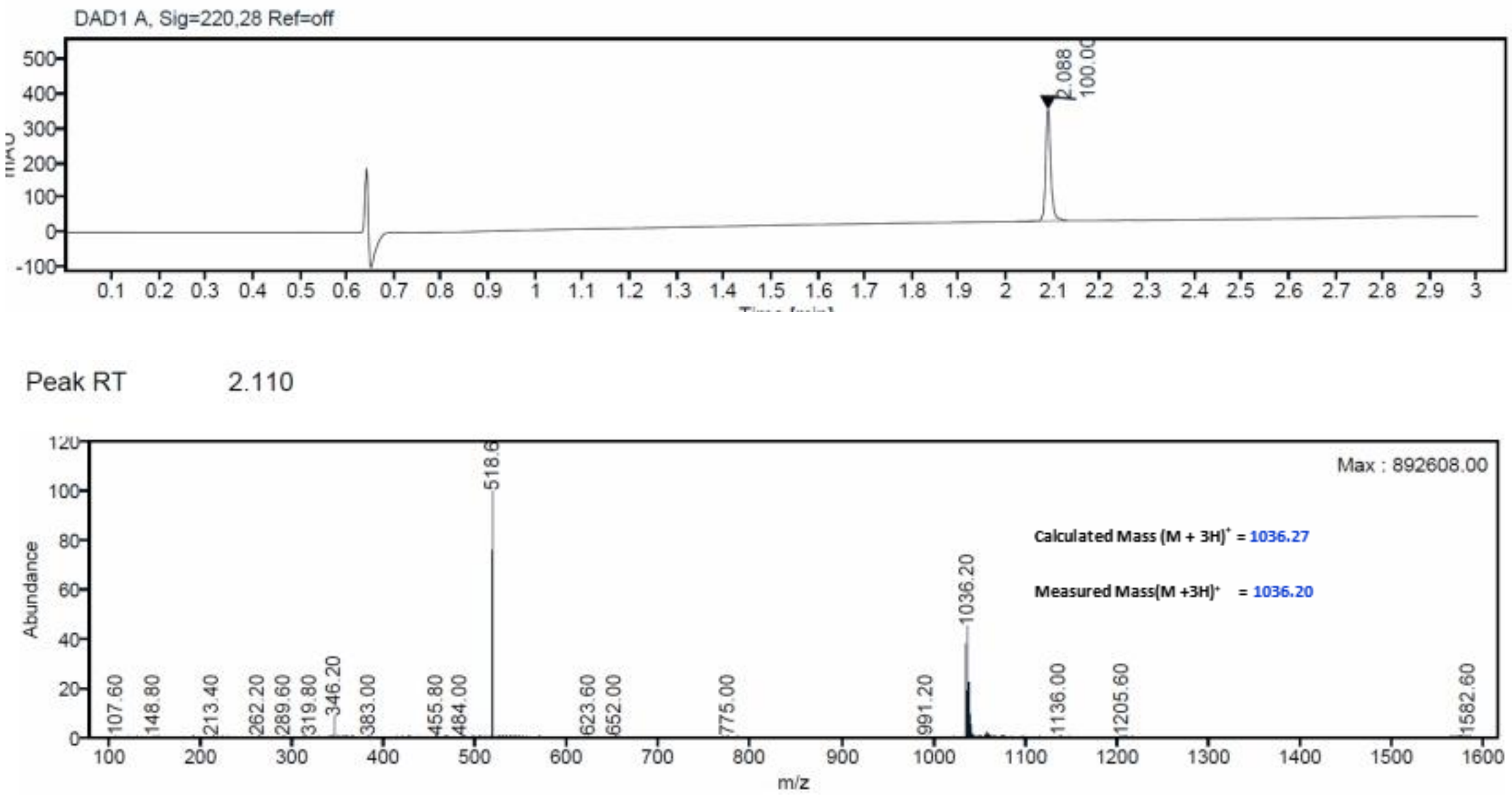

Supplementary Fig. 40 LCMS trace of **a1d**

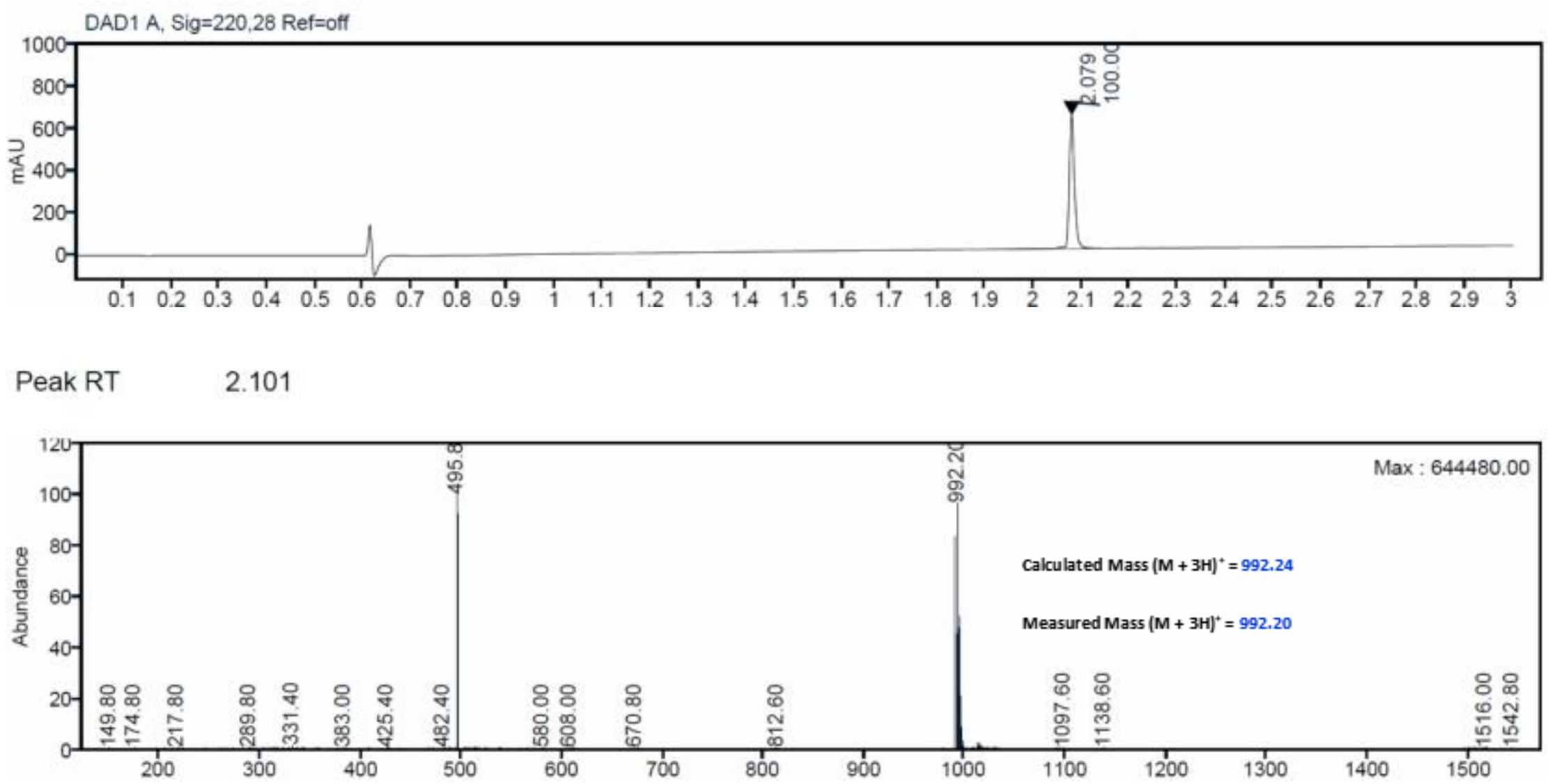

Supplementary Fig. 41 LCMS trace of **a1c**

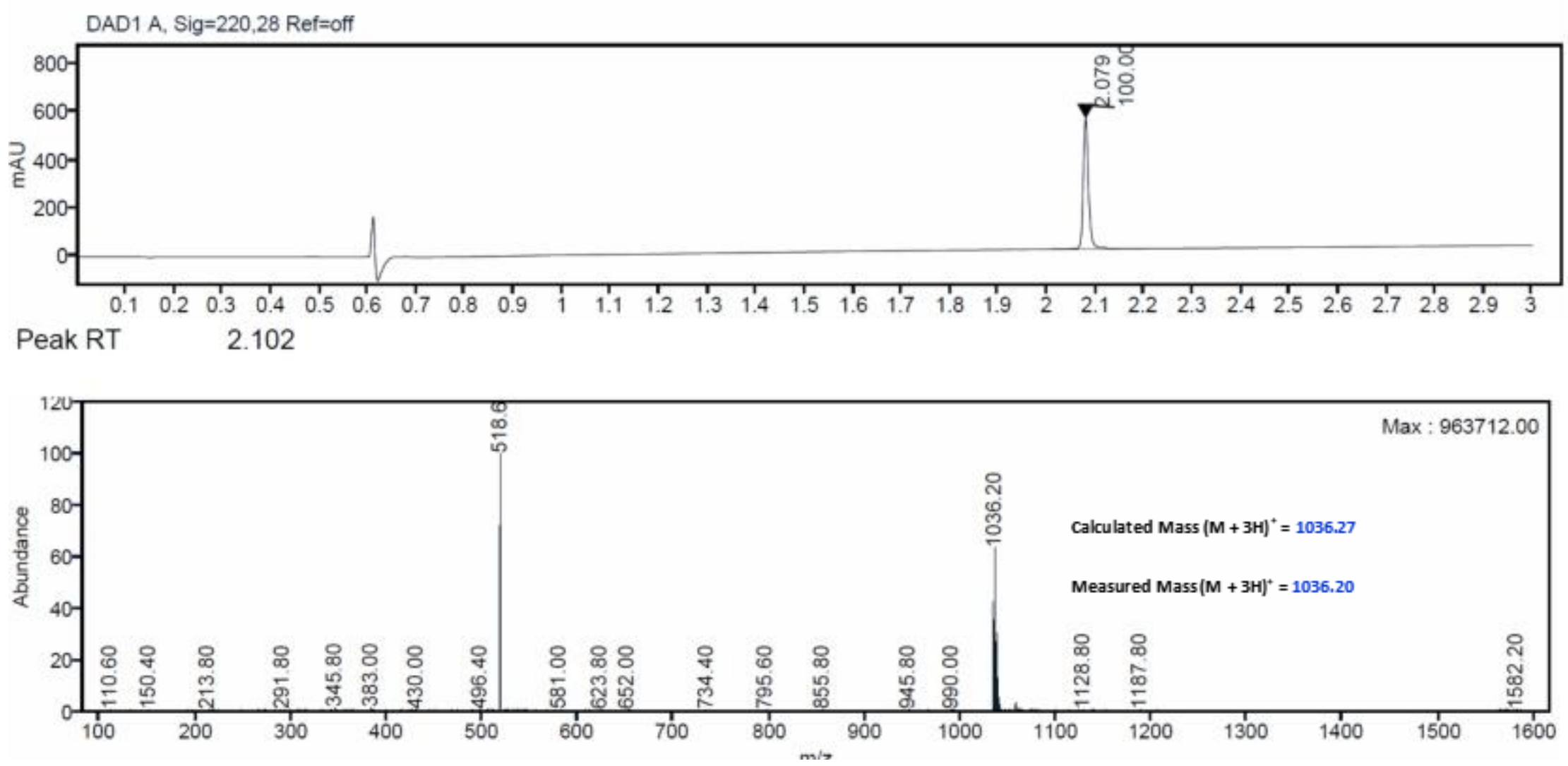

Supplementary Fig. 42 LCMS trace of **a2c**

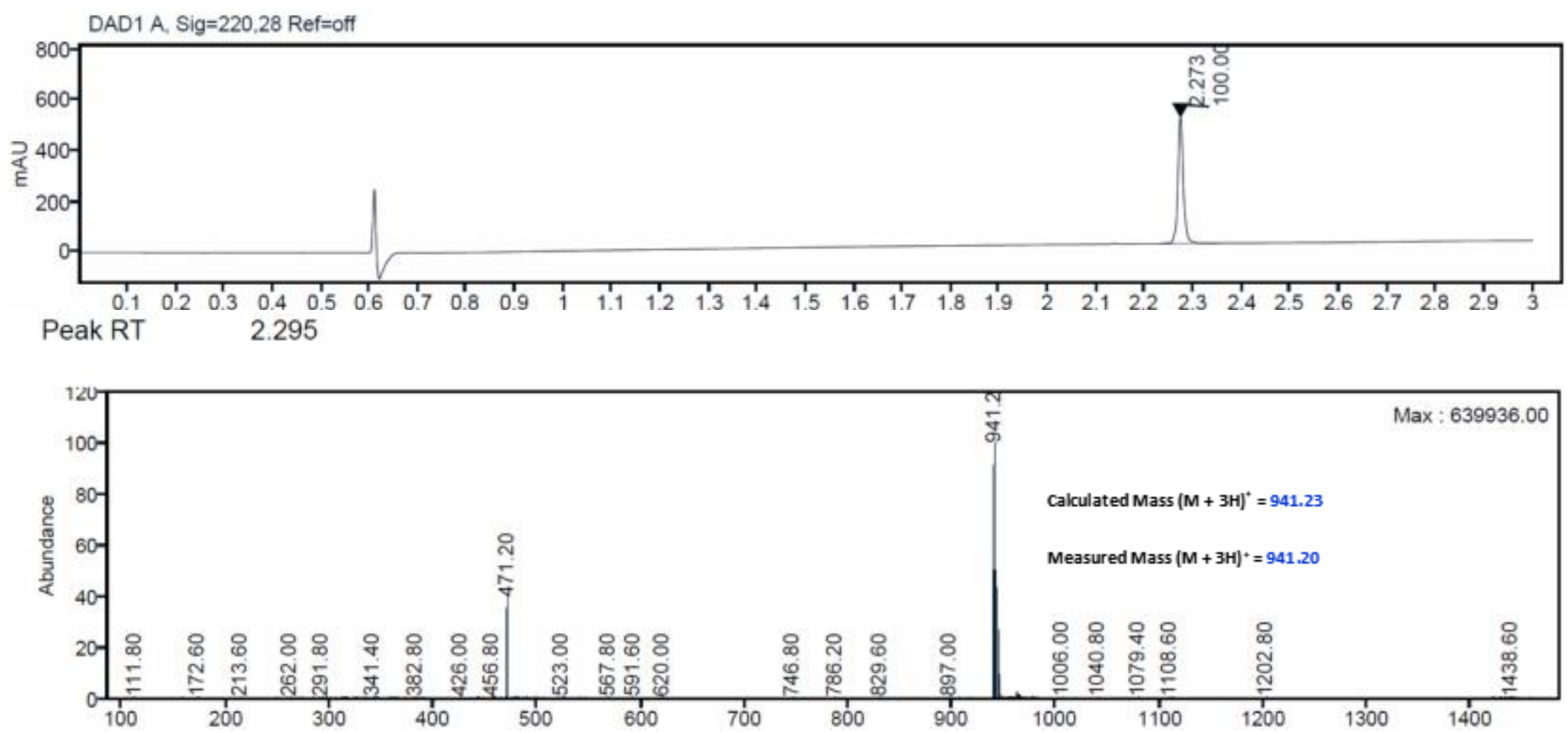

Supplementary Fig. 43 LCMS trace of **a1i**

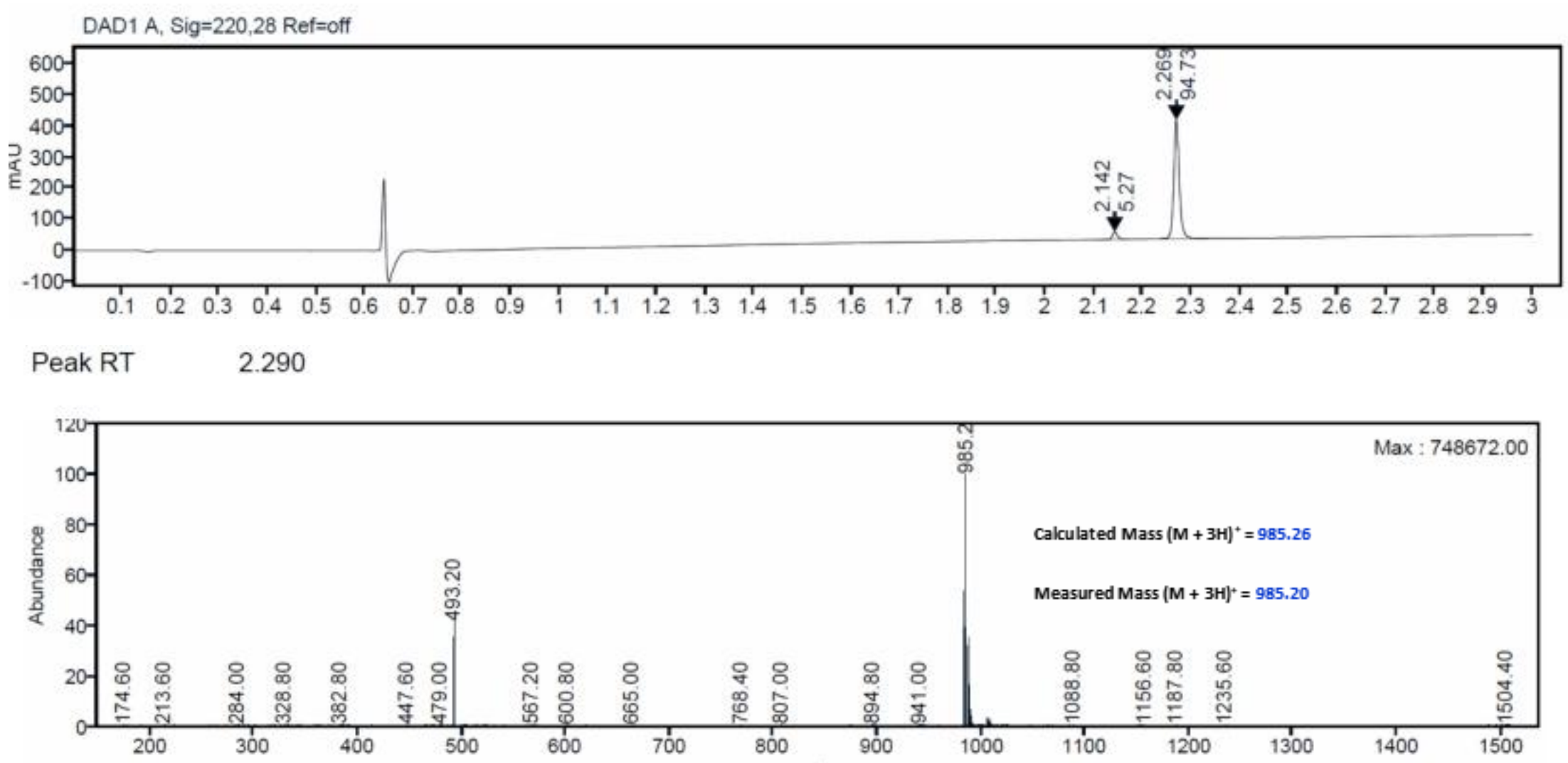

Supplementary Fig. 44 LCMS trace of **a2i**

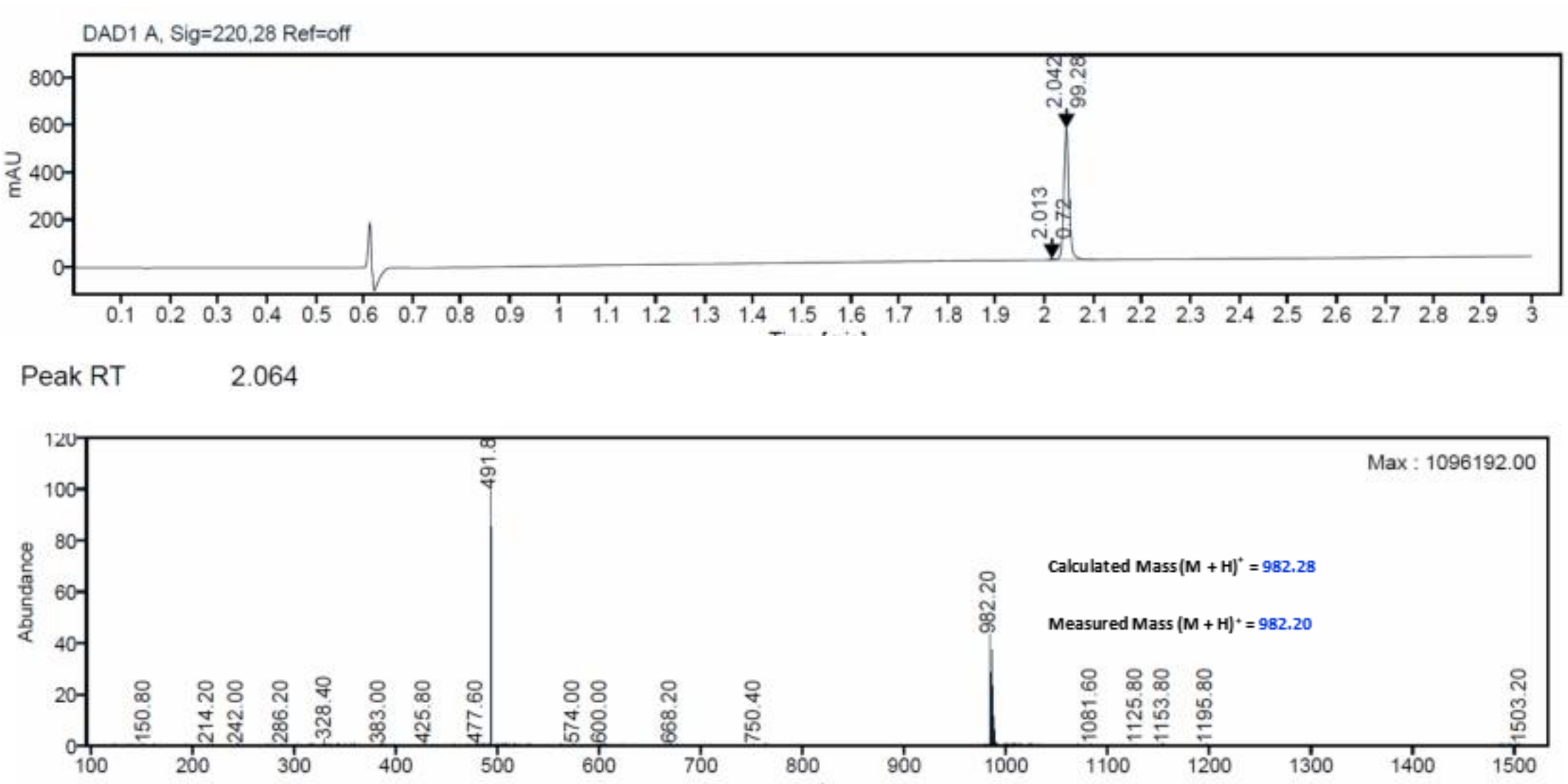

Supplementary Fig. 45 LCMS trace of **a1h**

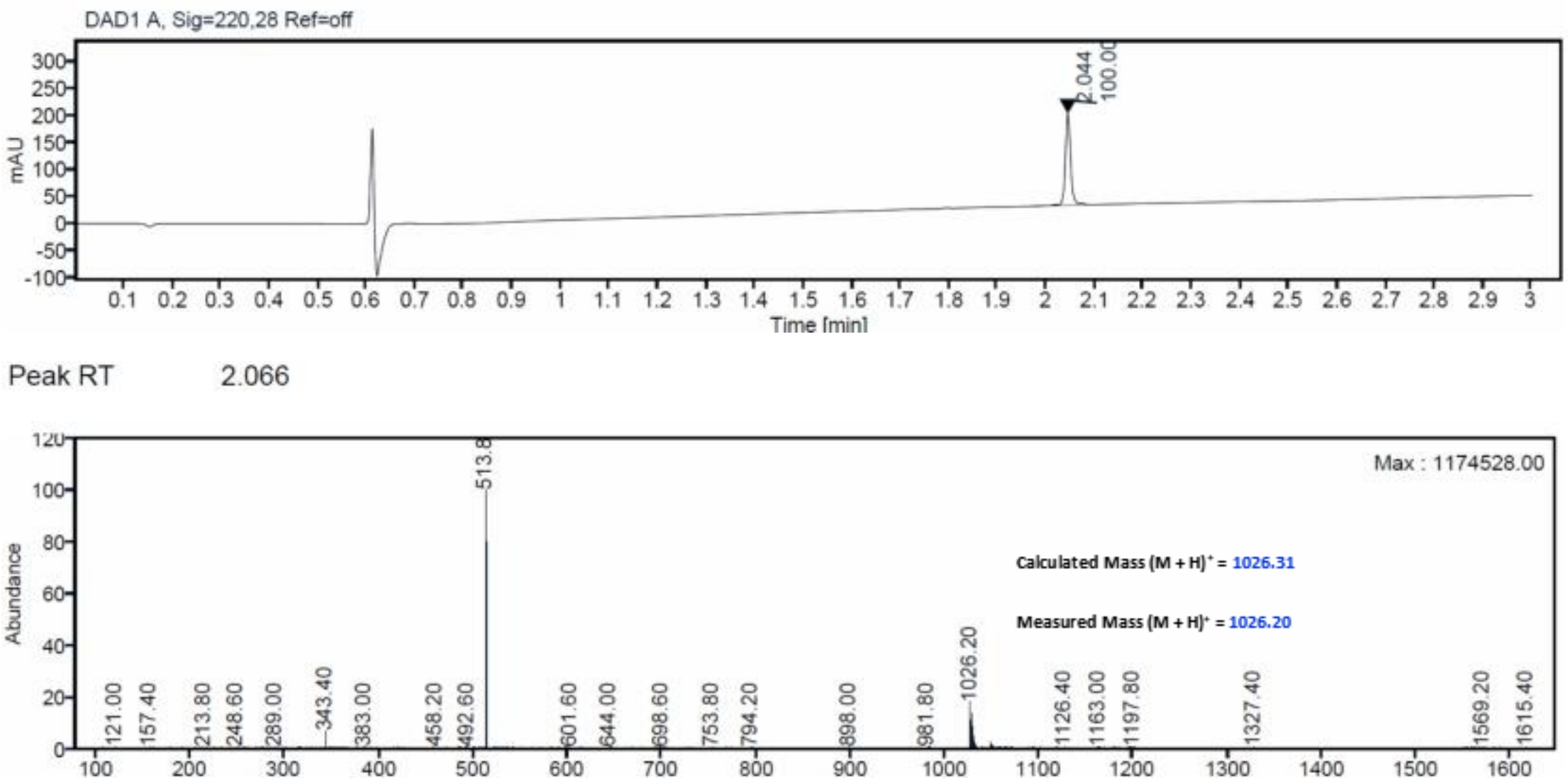

Supplementary Fig. 46 LCMS trace of **a2h**

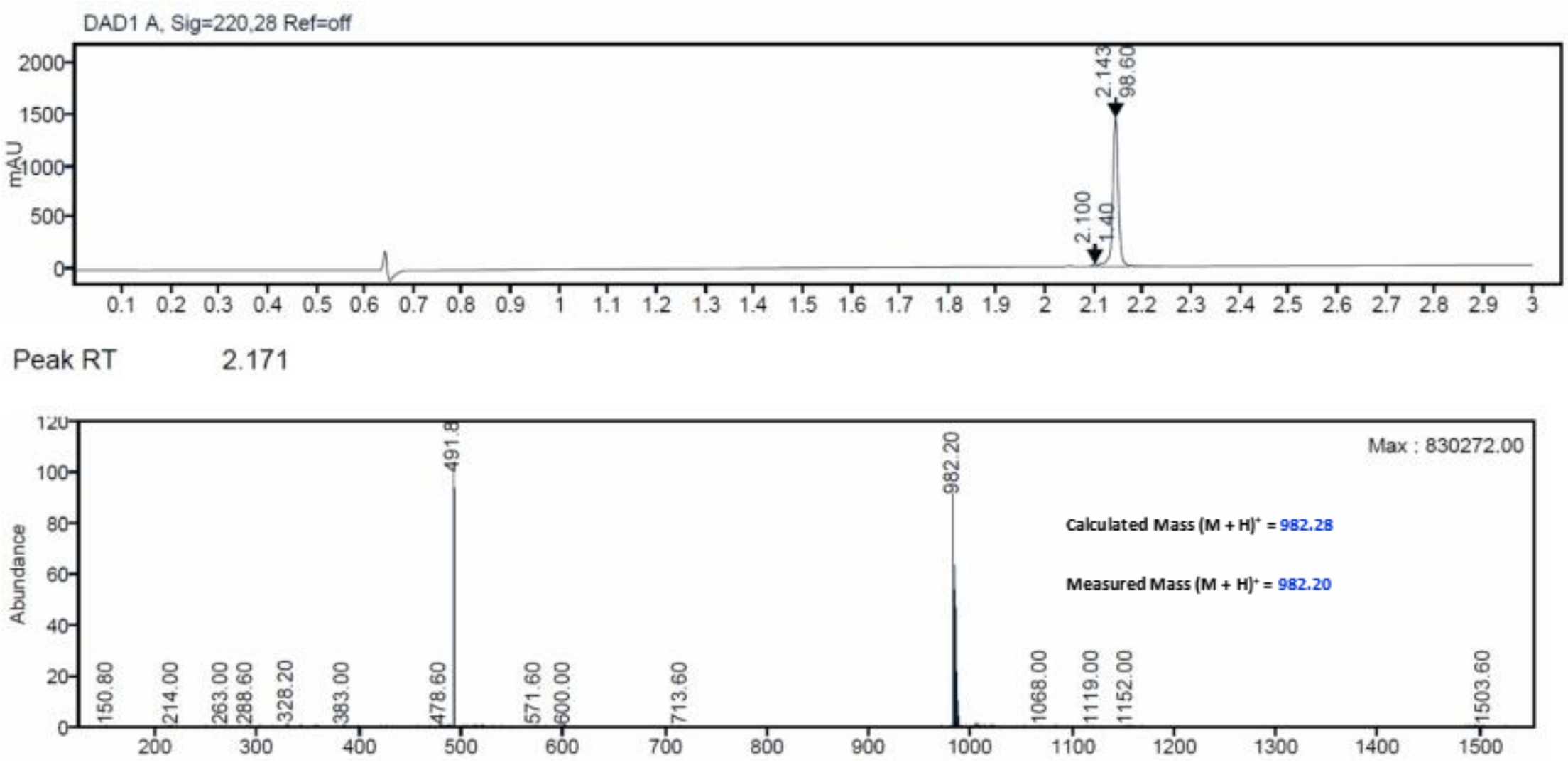

Supplementary Fig. 47 LCMS trace of **a1g**

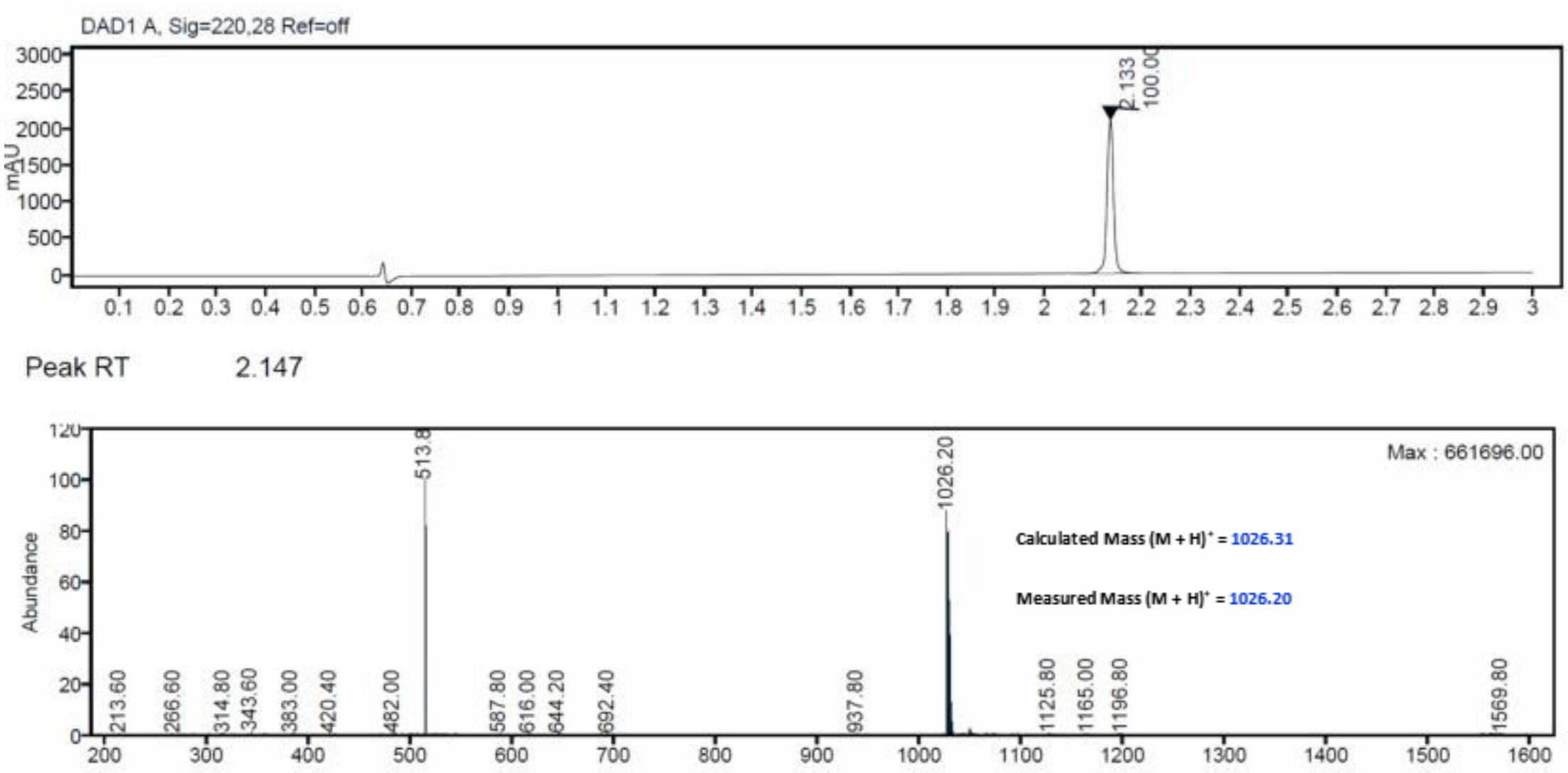

Supplementary Fig. 48 LCMS trace of **a2g**

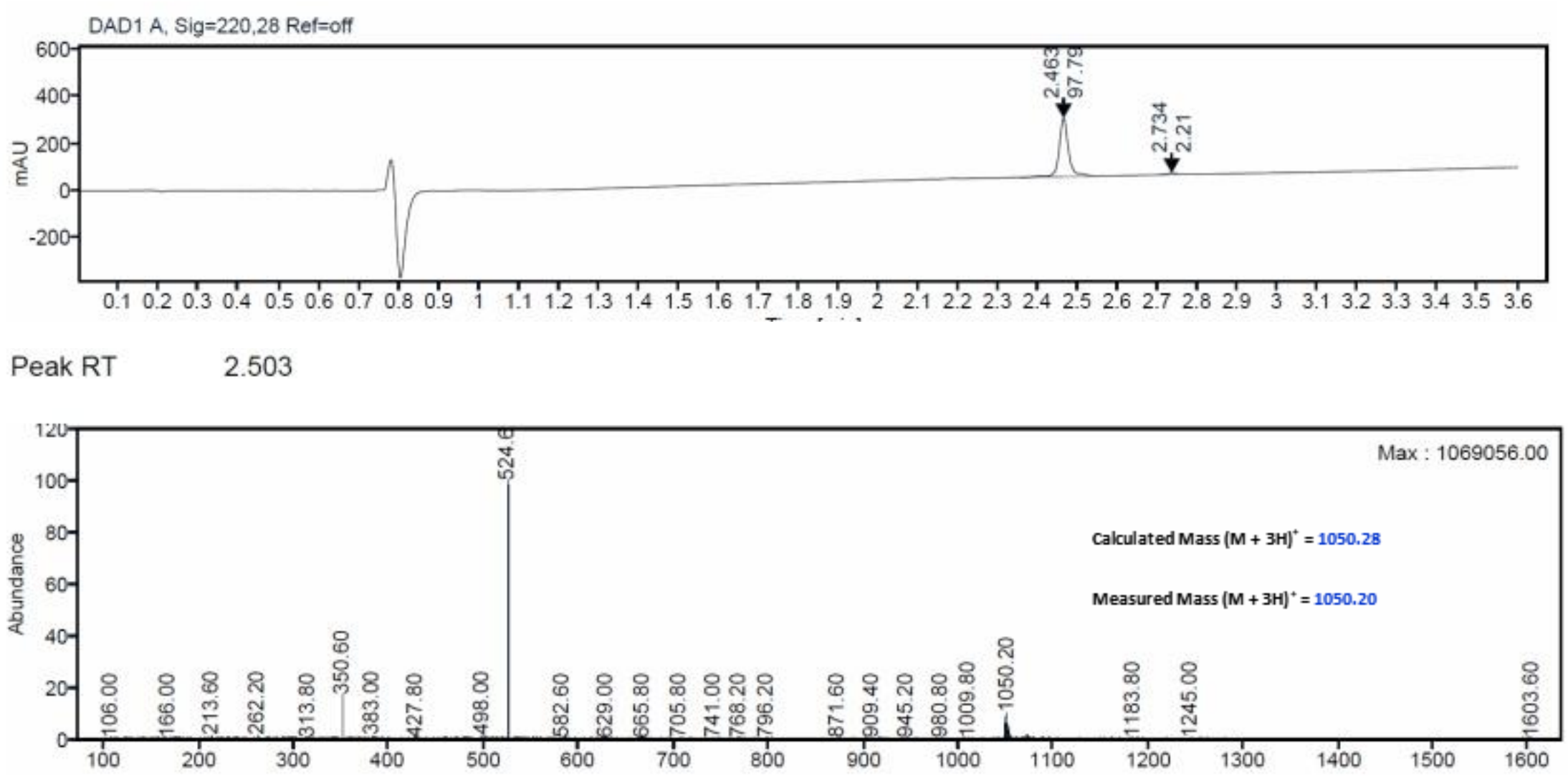

Supplementary Fig. 49 LCMS trace of **a1dj**

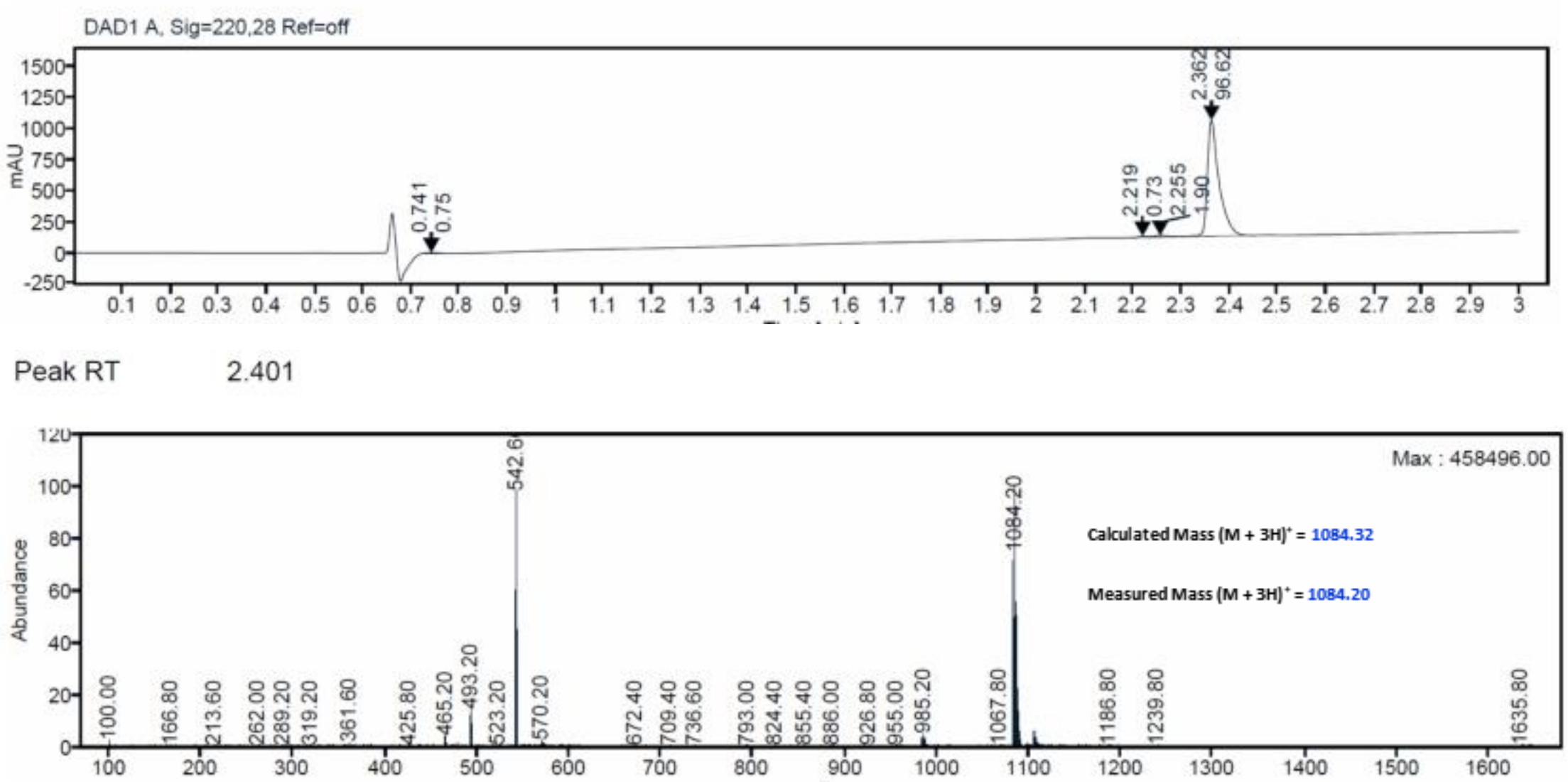

Supplementary Fig. 50 LCMS trace of **a1dk**

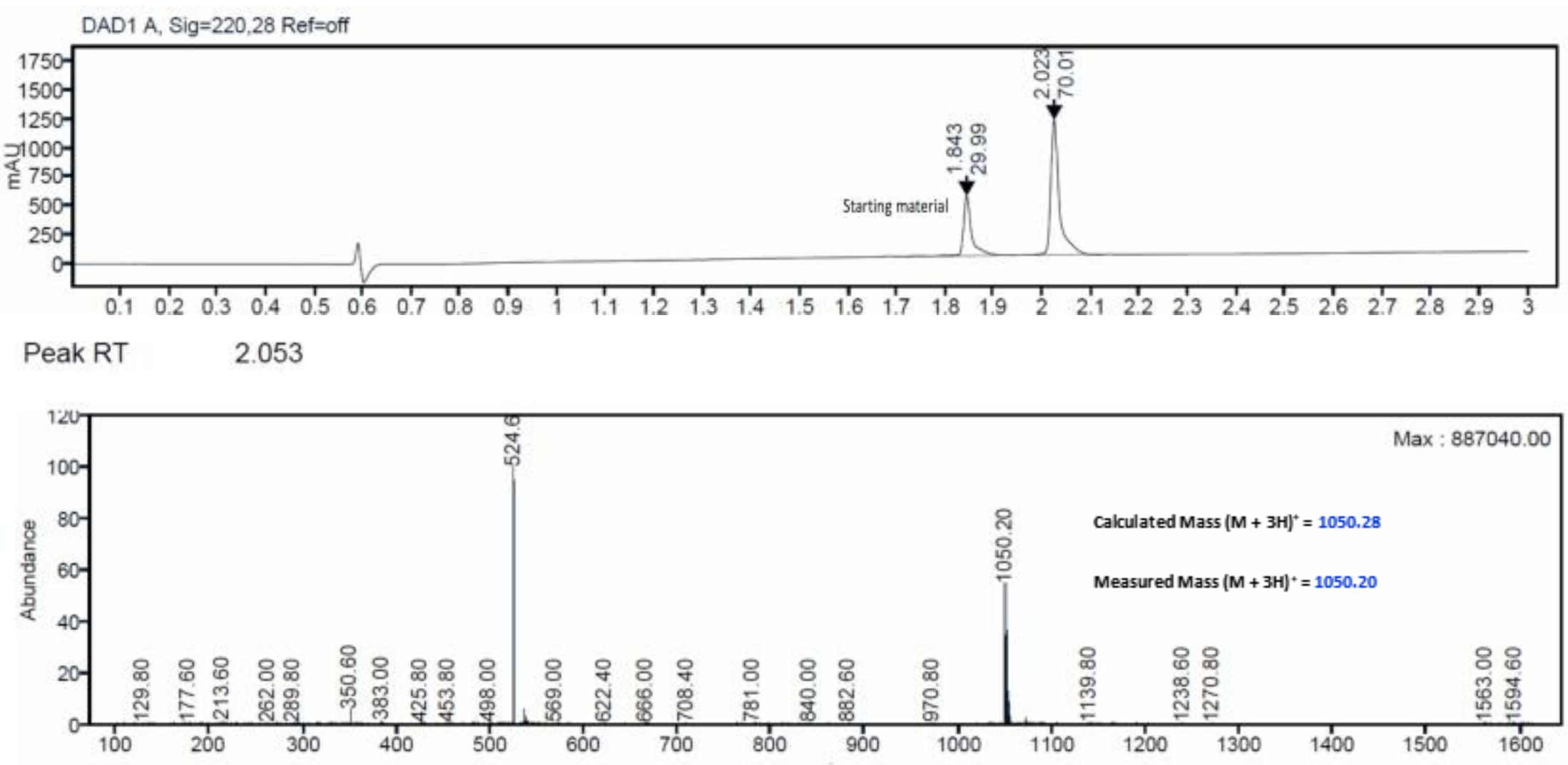

Supplementary Fig. 51 LCMS trace of **a1e**

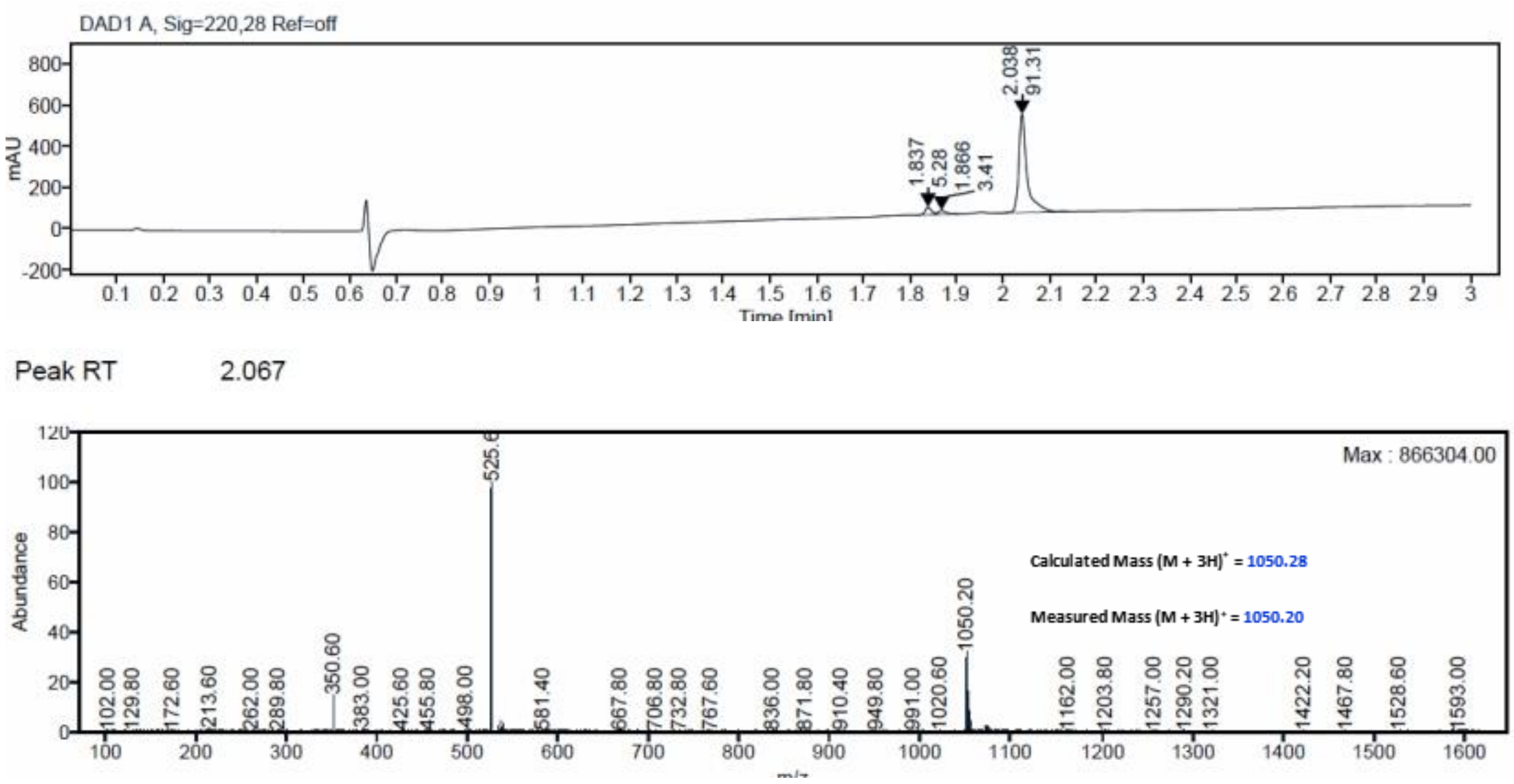

Supplementary Fig. 52 LCMS trace of **a1f**

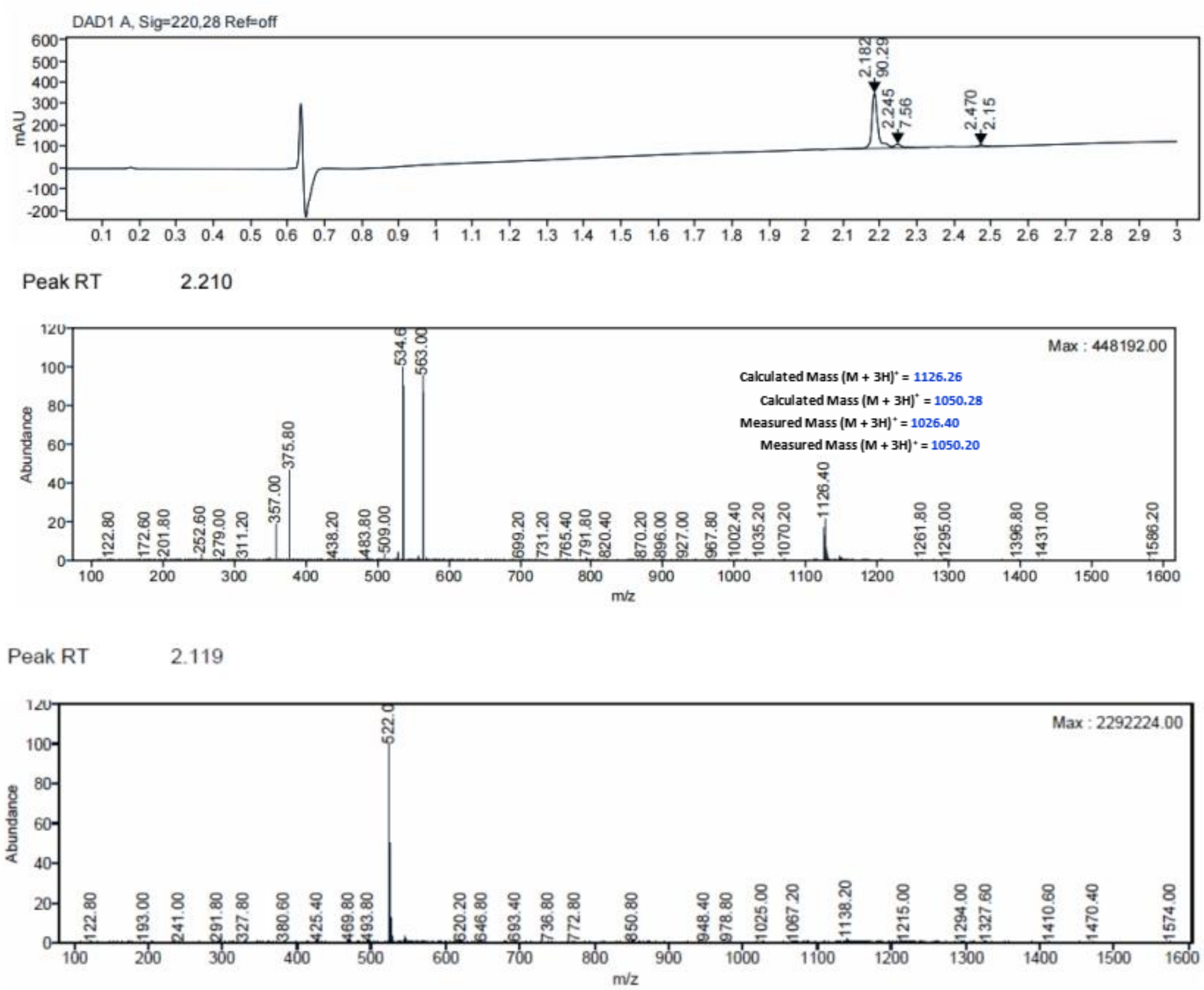

Supplementary Fig. 53 LCMS trace of **b3d**

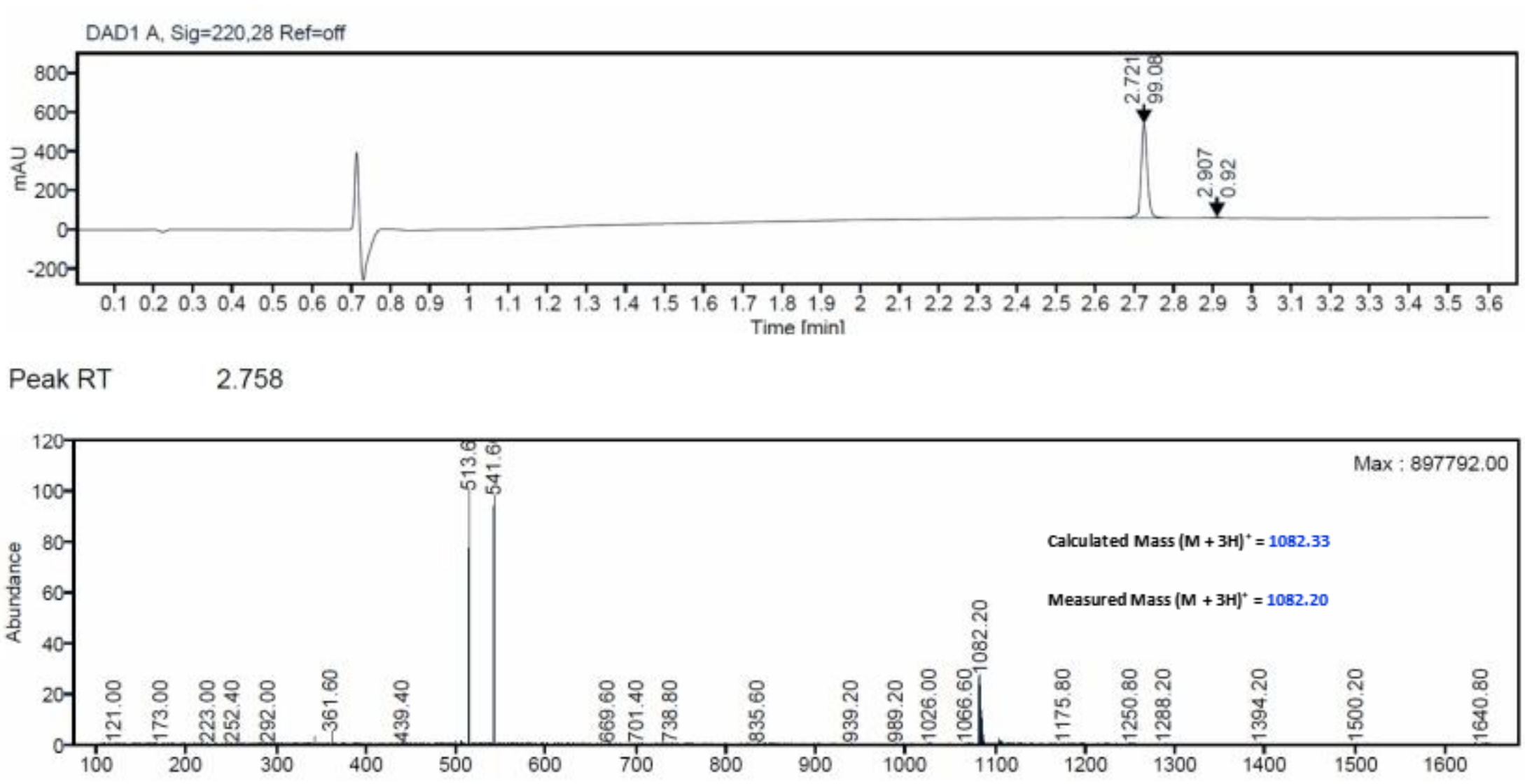

Supplementary Fig. 54 LCMS trace of **b3c**

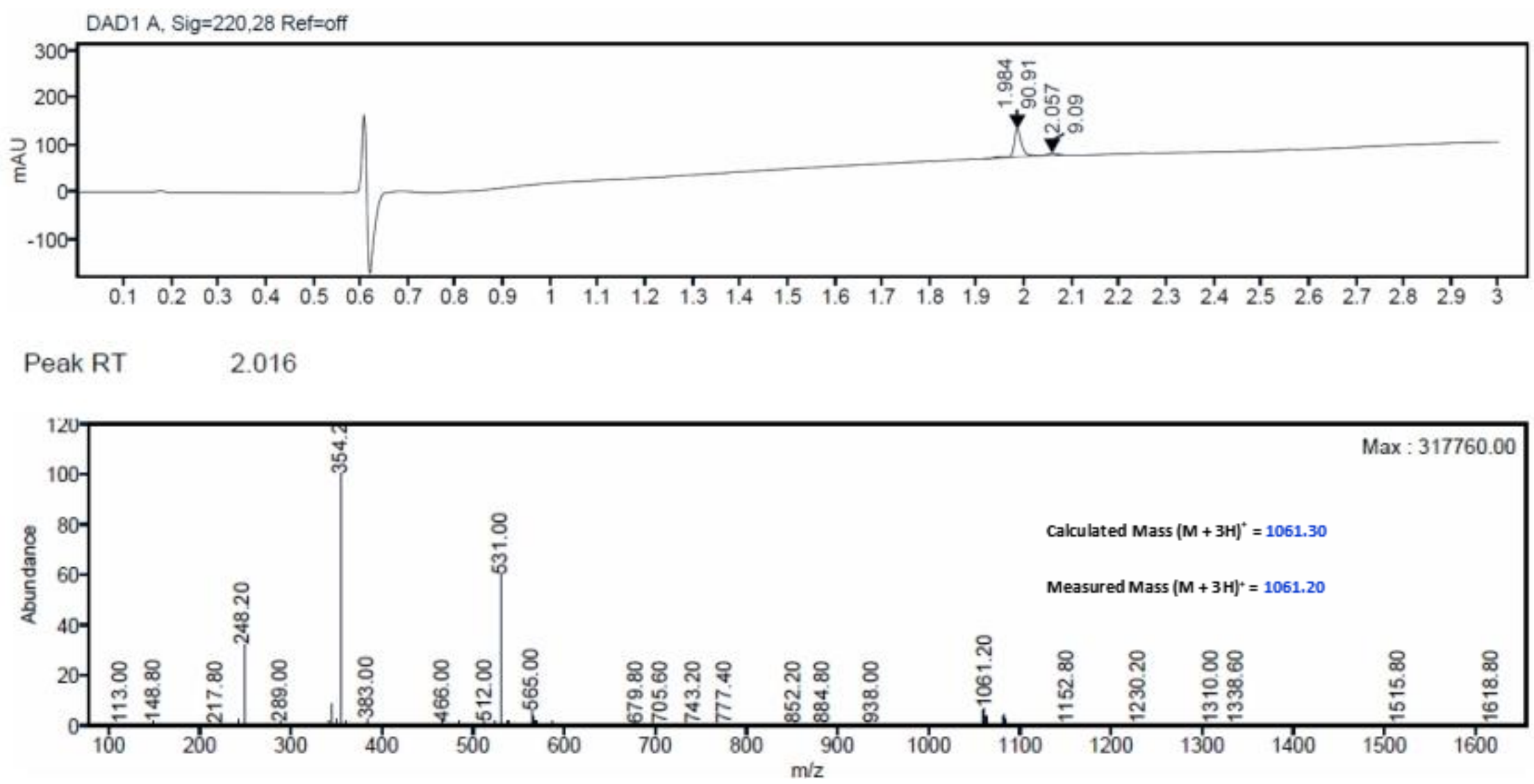

Supplementary Fig. 55 LCMS trace of **a4d**

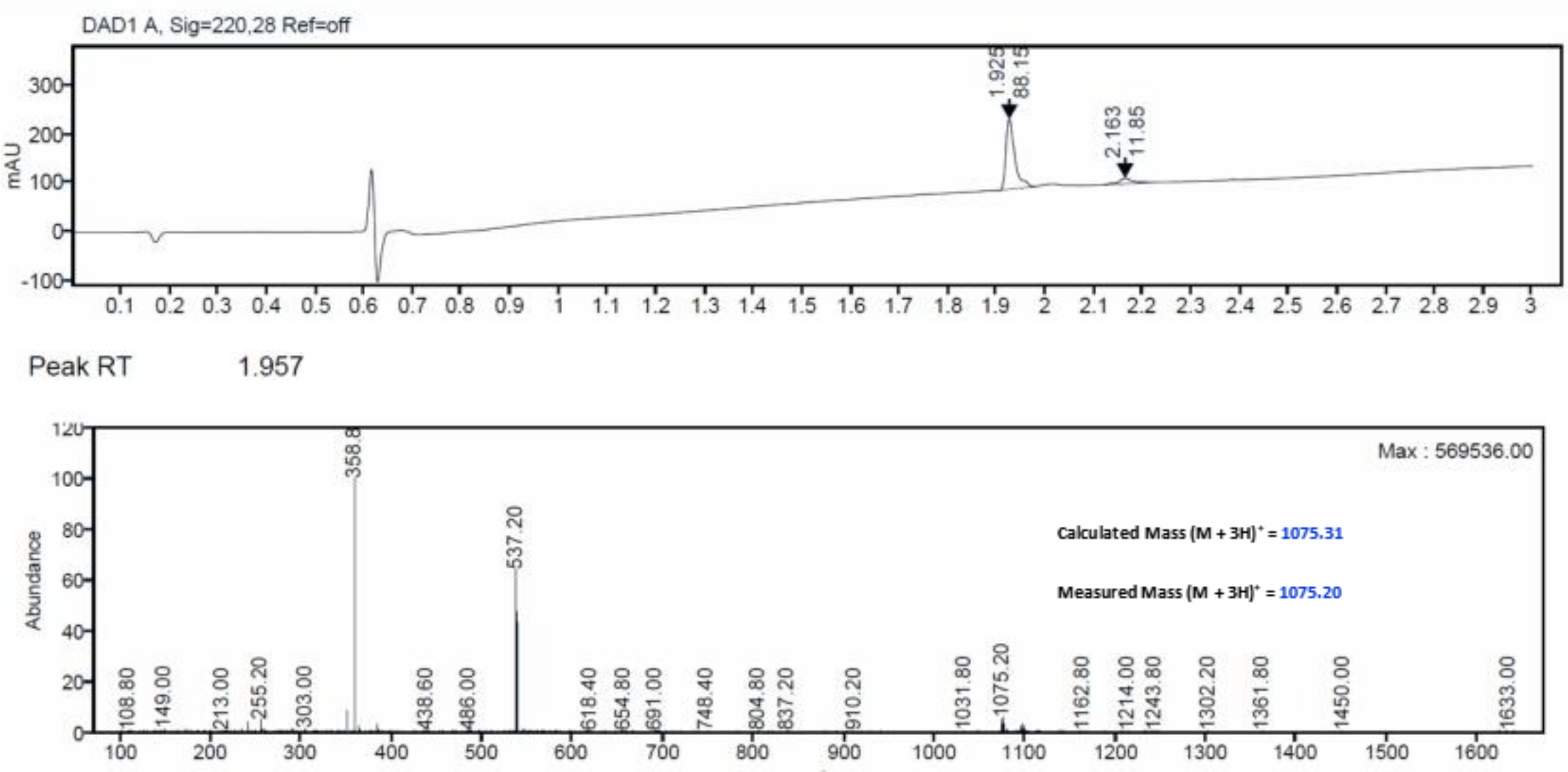

Supplementary Fig. 56 LCMS trace of **a5d**

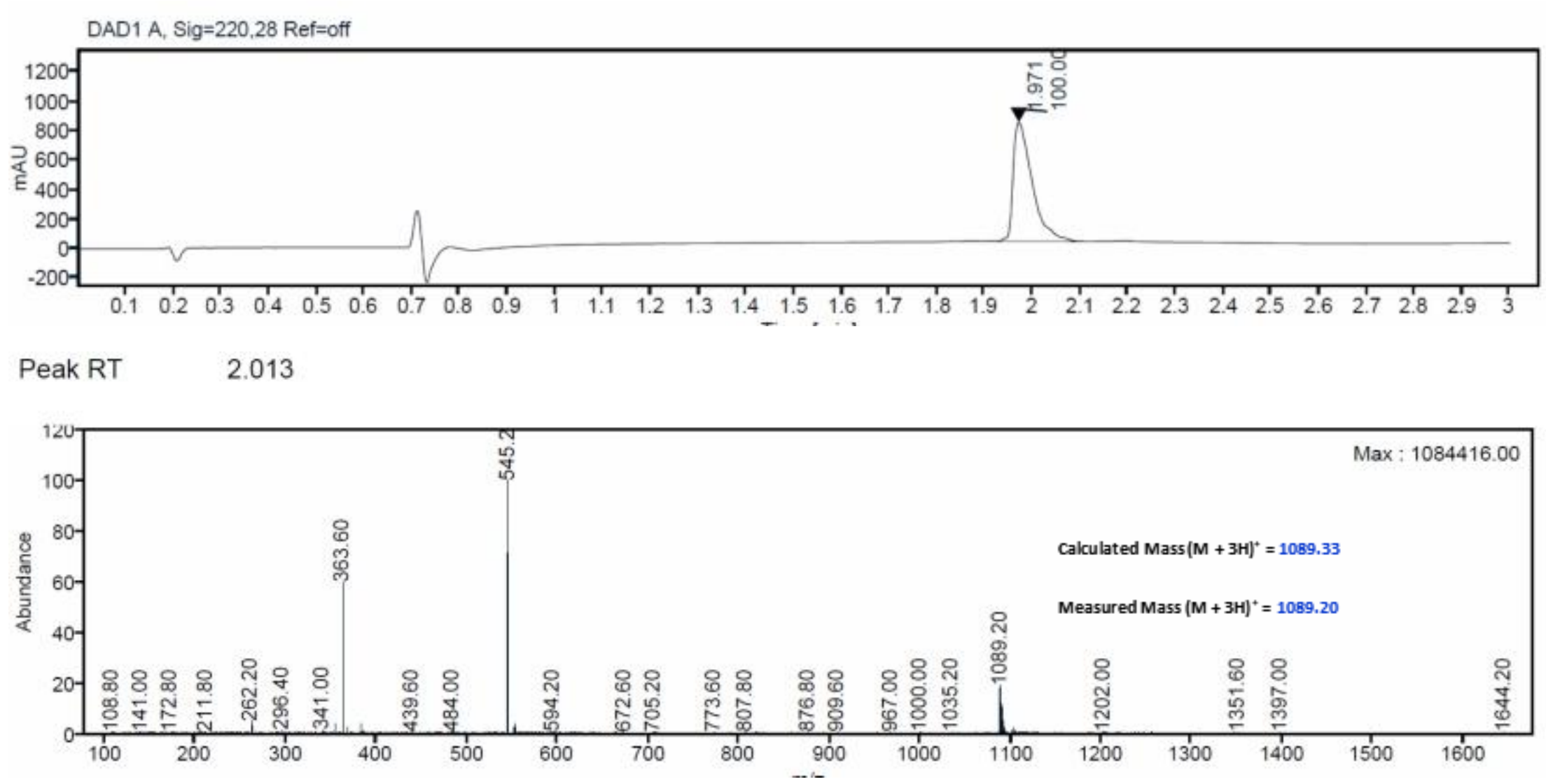

Supplementary Fig. 57 LCMS trace of **a6d**

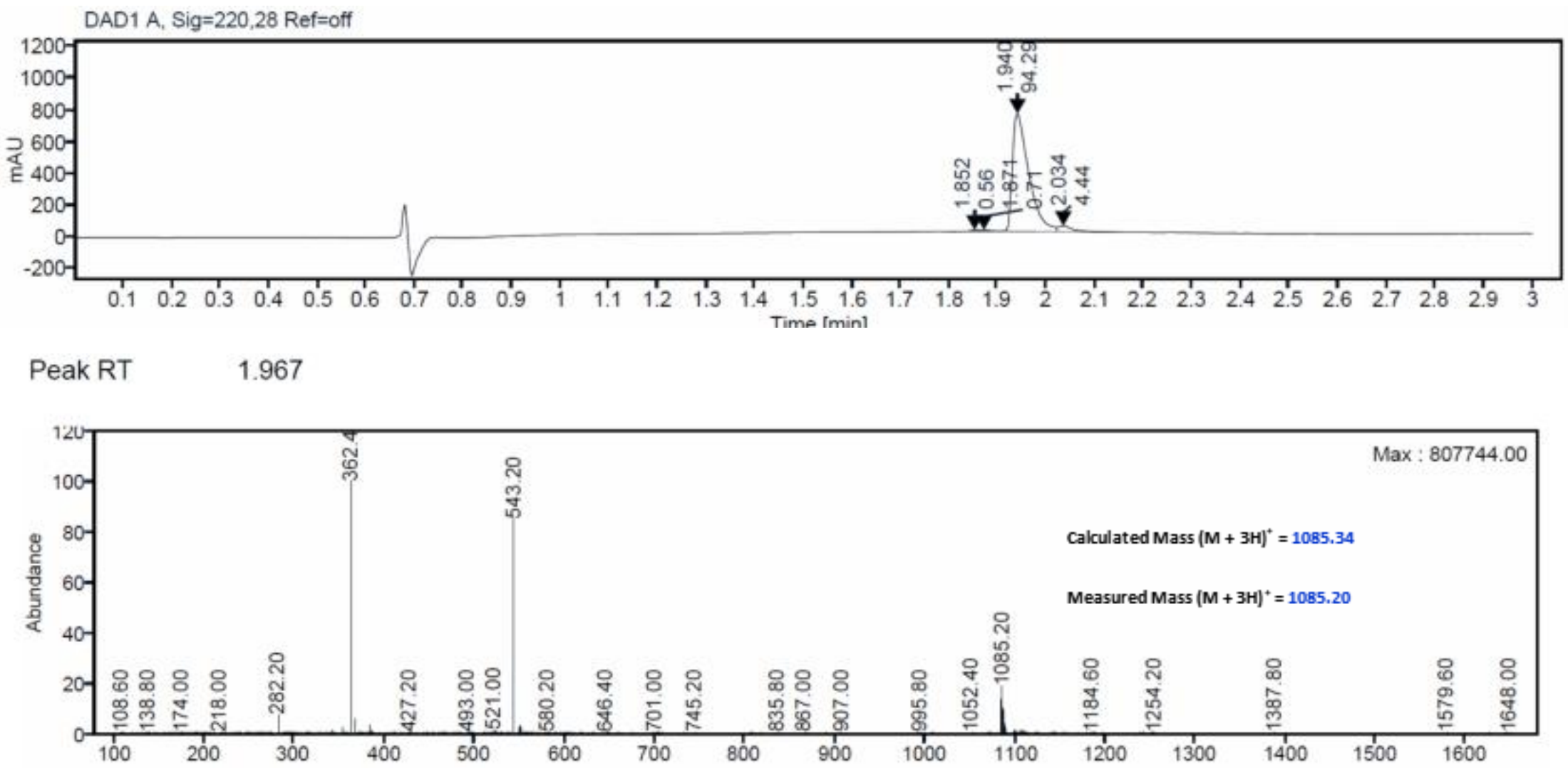

Supplementary Fig. 58 LCMS trace of **a7c**

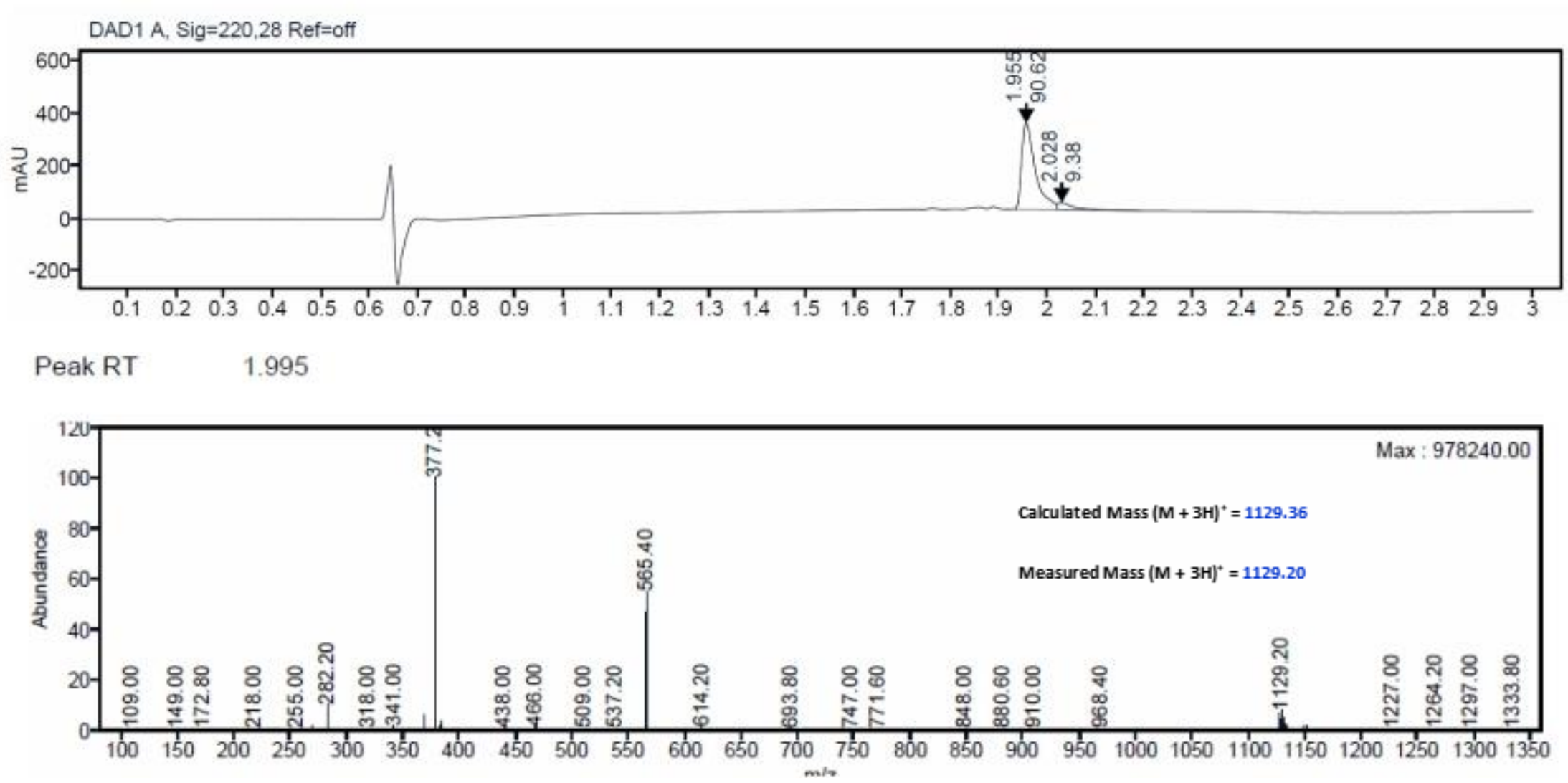

Supplementary Fig. 59 LCMS trace of **a7d**

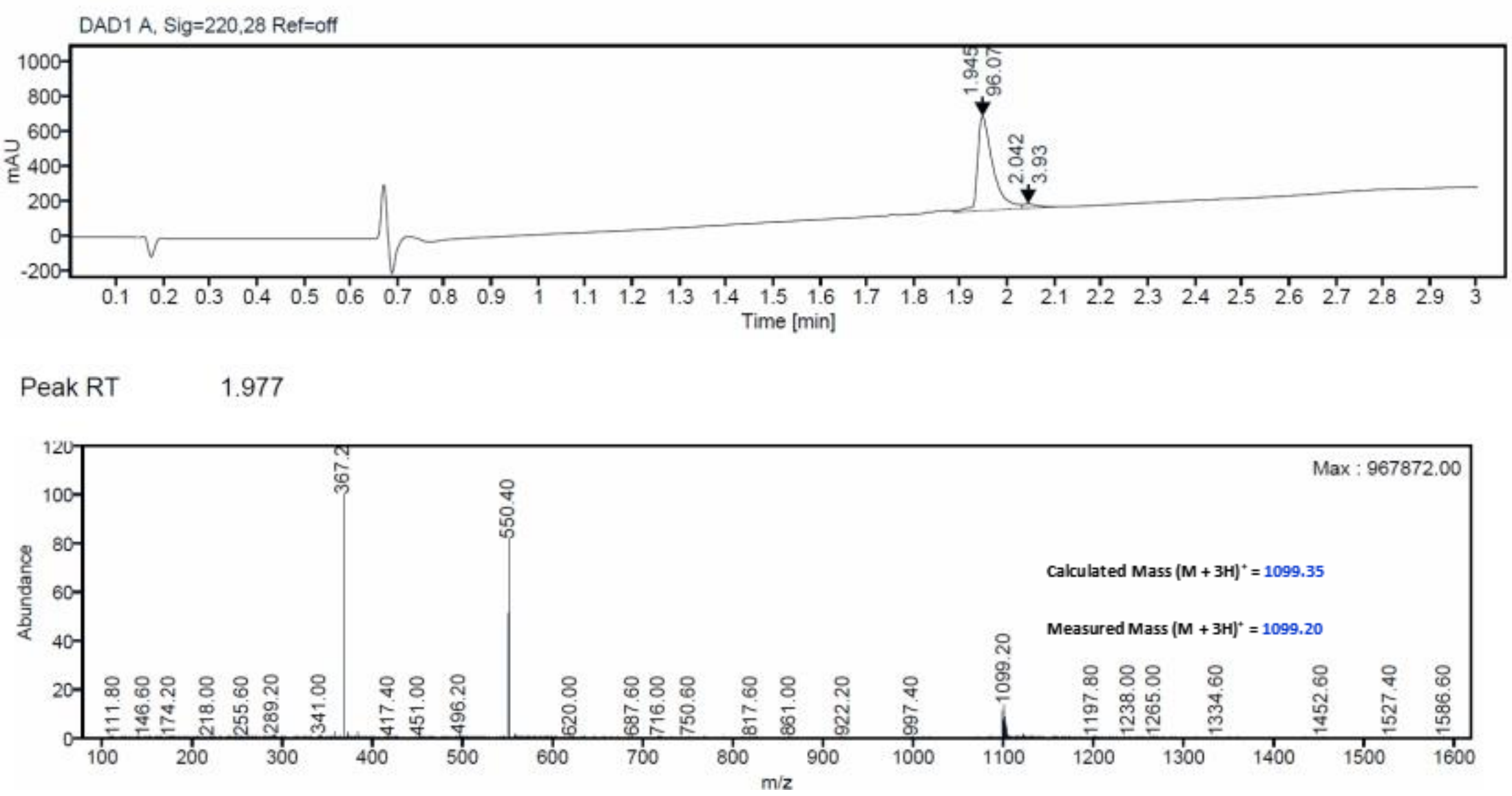

Supplementary Fig. 60 LCMS trace of **a8c**

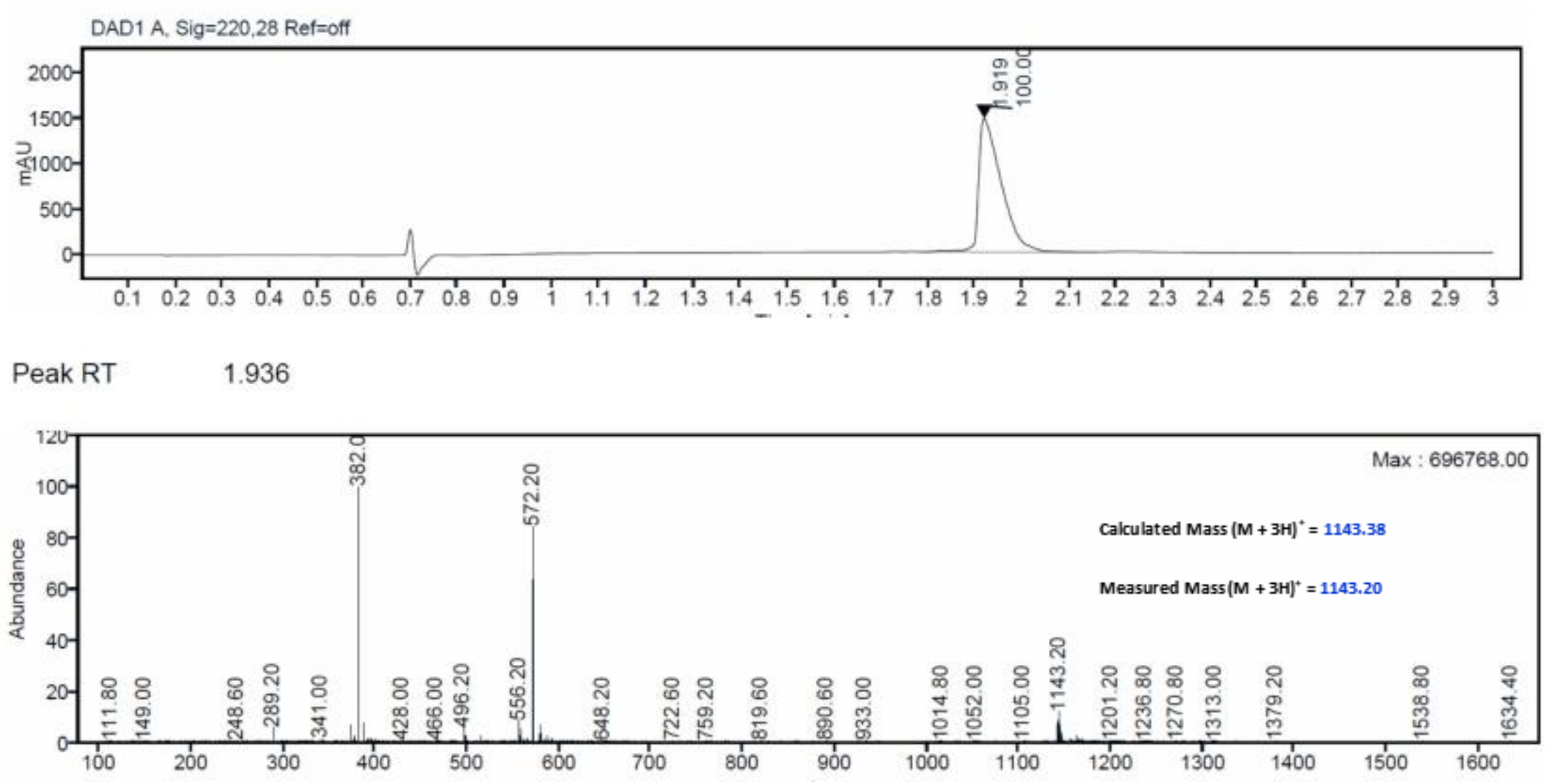

Supplementary Fig. 61 LCMS trace of **a8d**

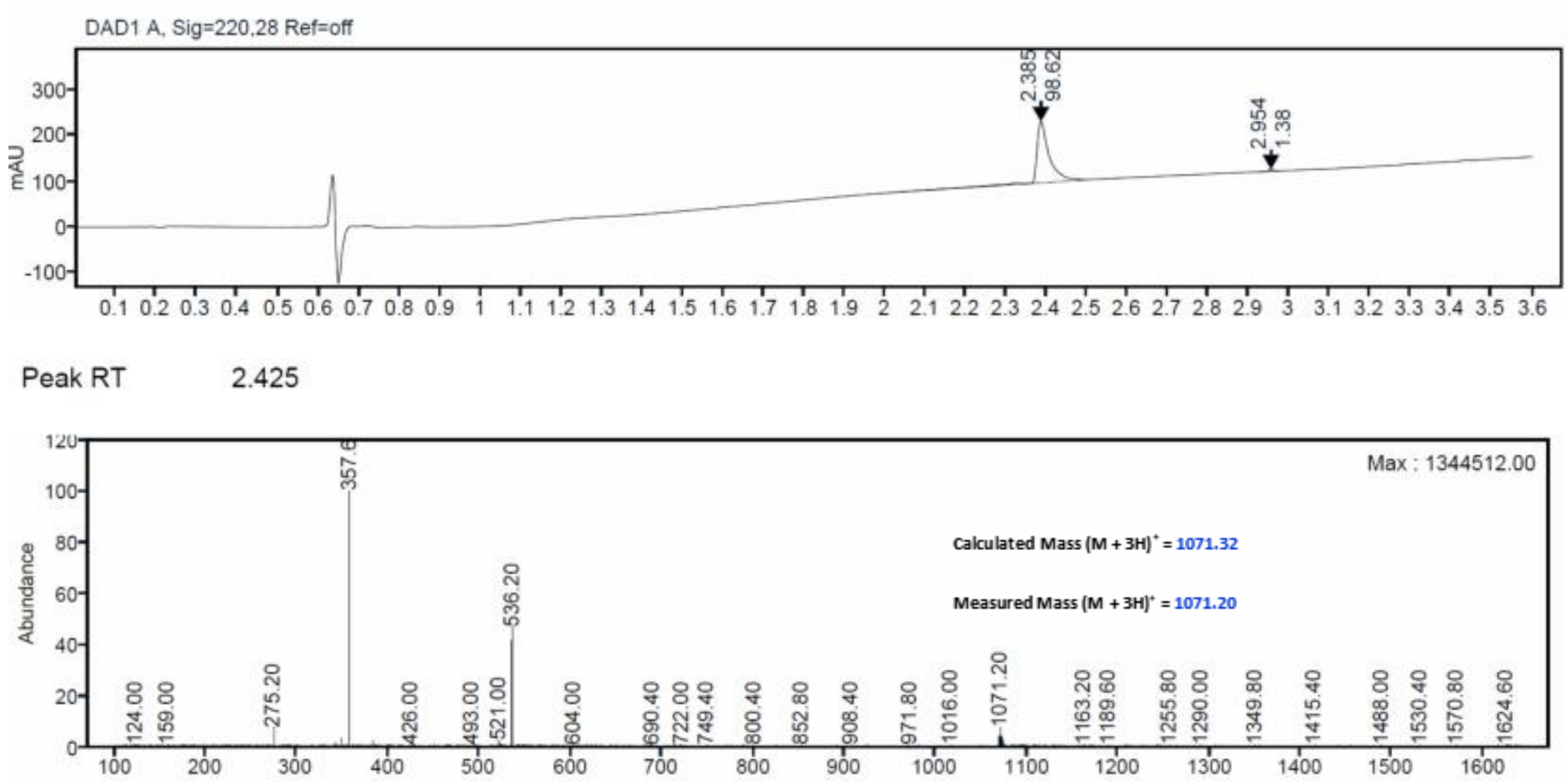

Supplementary Fig. 62 LCMS trace of **a9c**

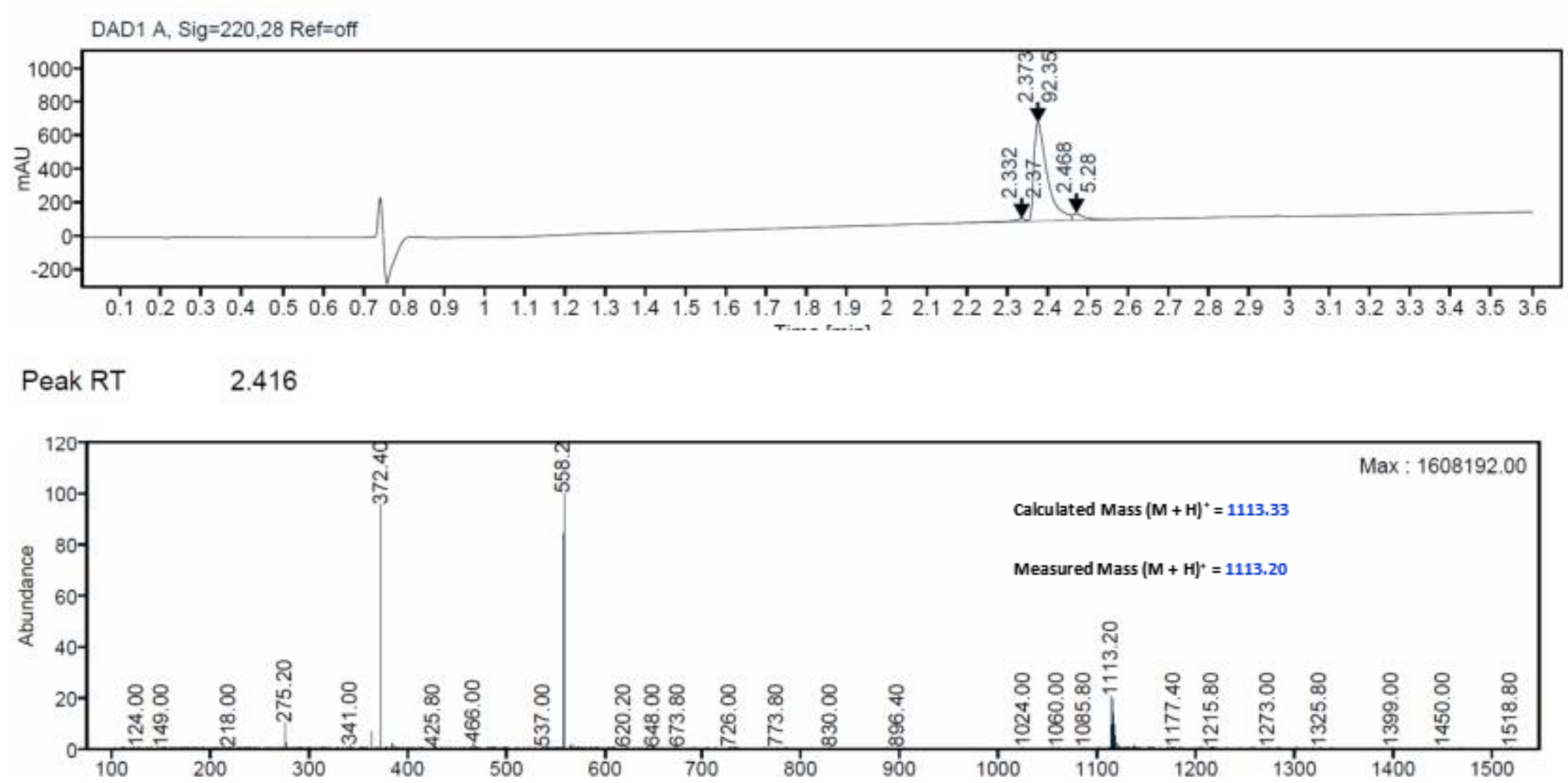

Supplementary Fig. 63 LCMS trace of **a9d**

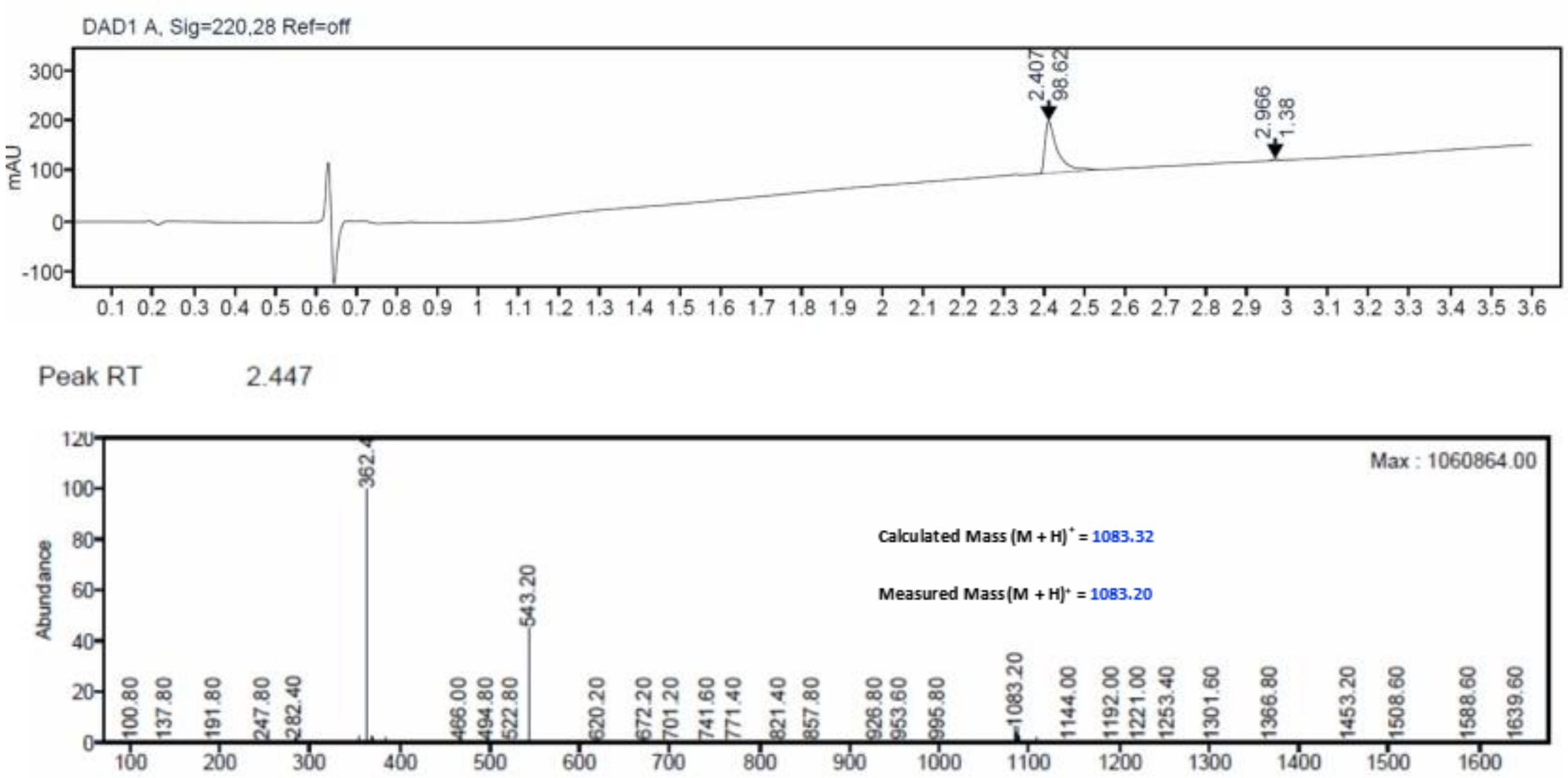

Supplementary Fig. 64 LCMS trace of **a10c**

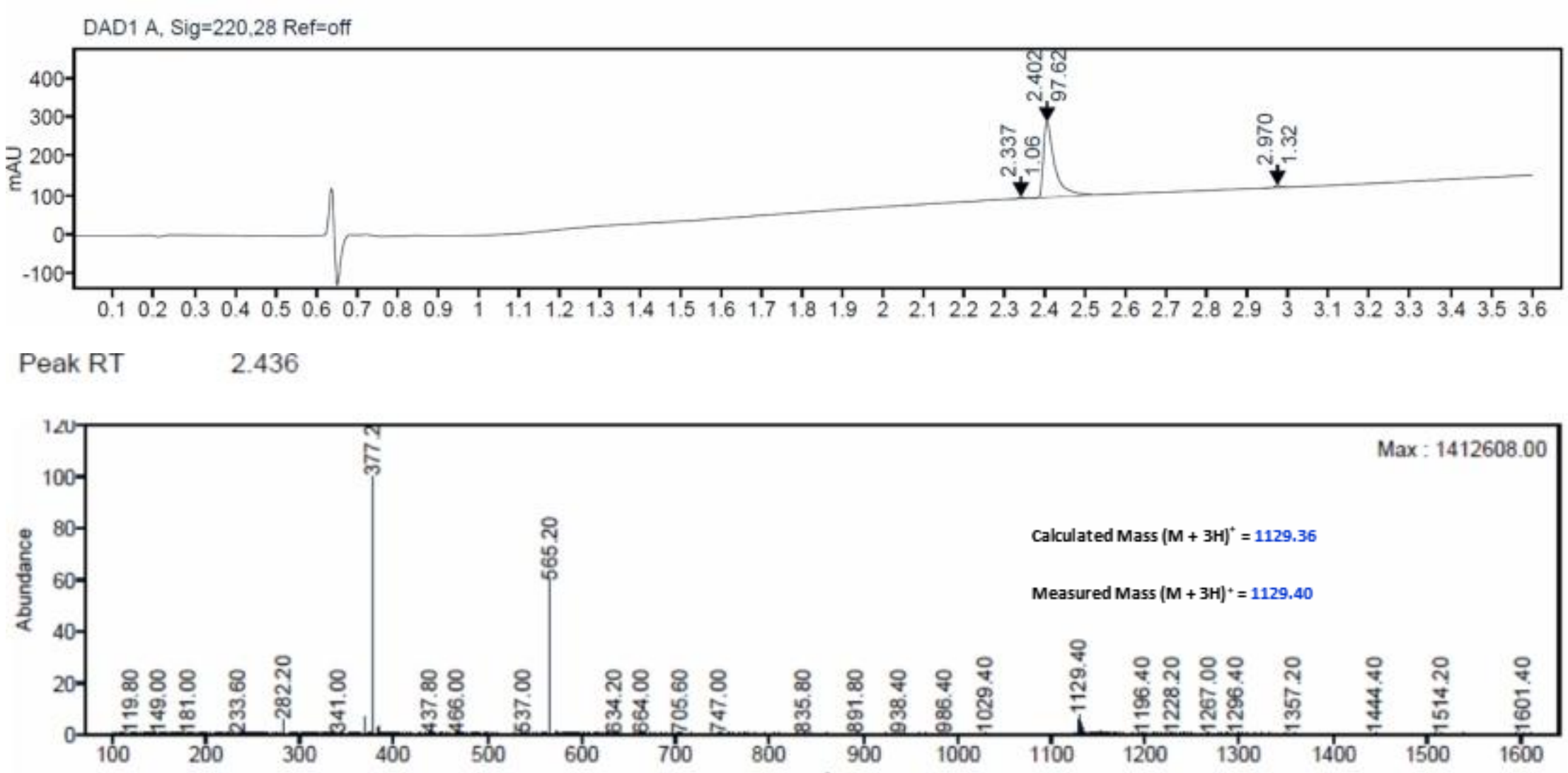

Supplementary Fig. 65 LCMS trace of **a10d**

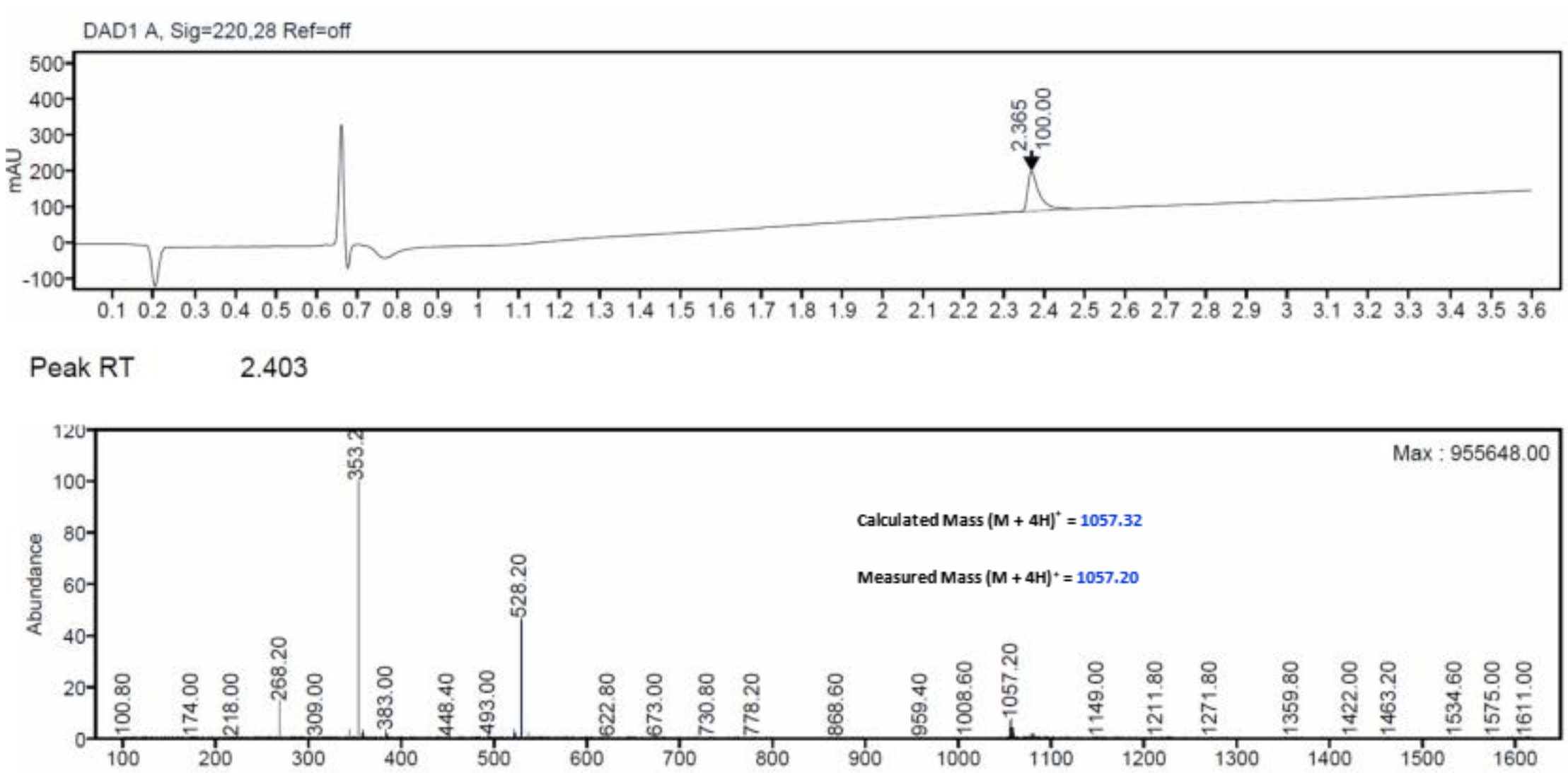

Supplementary Fig. 66 LCMS trace of **a11c**

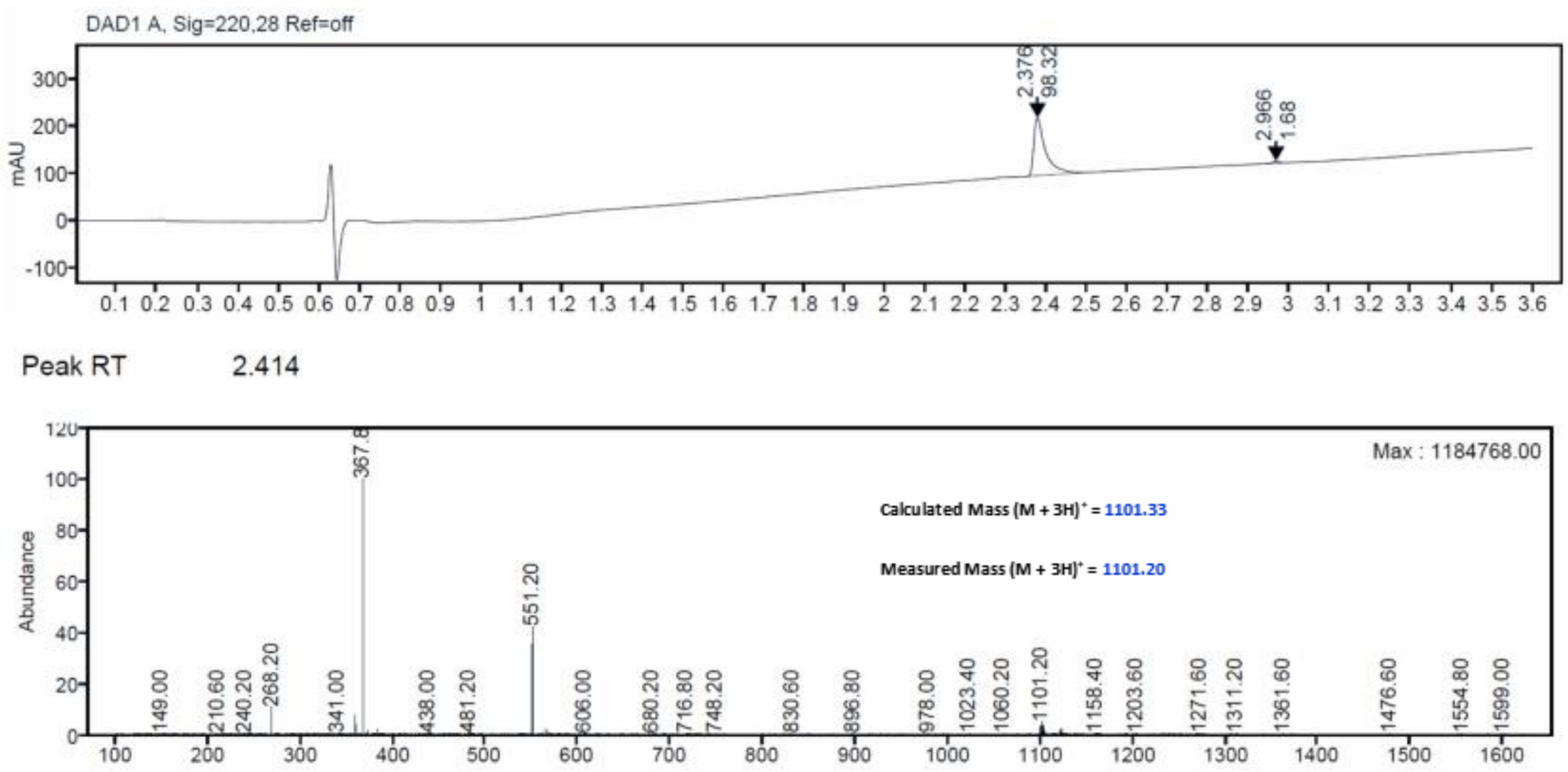

Supplementary Fig. 67 LCMS trace of **a11d**

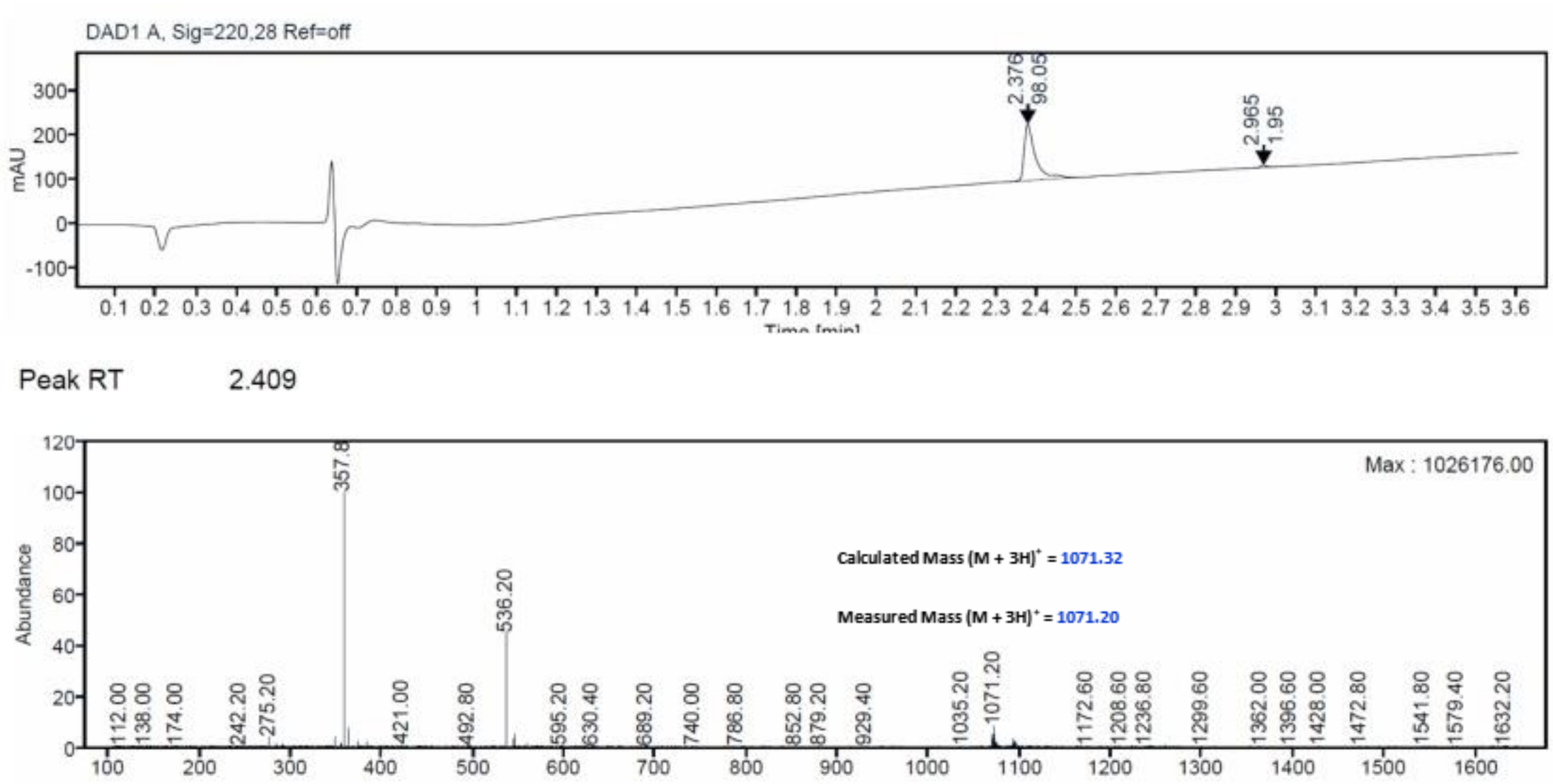

Supplementary Fig. 68 LCMS trace of **a12c**

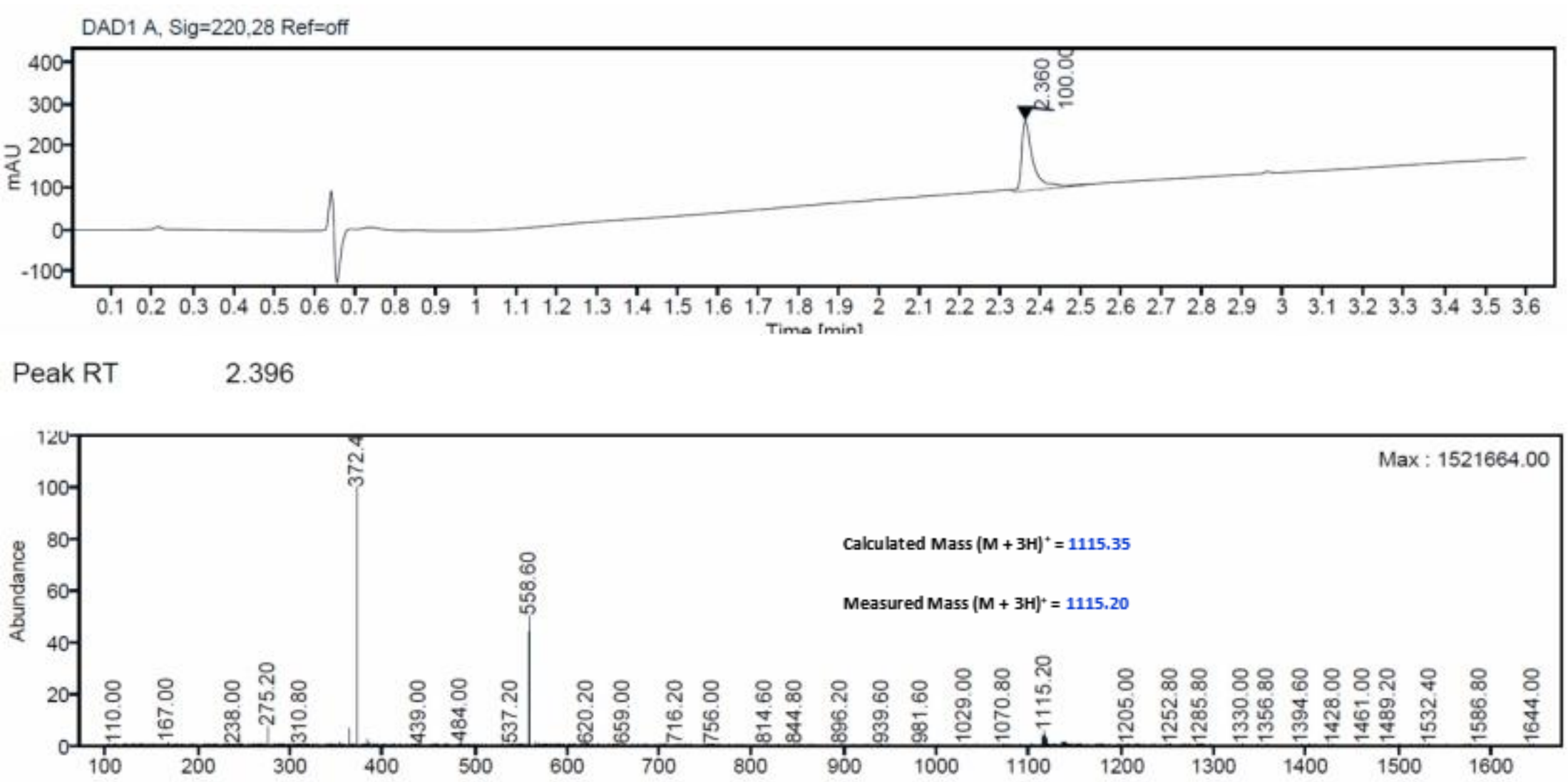

Supplementary Fig. 69 LCMS trace of **a12d**

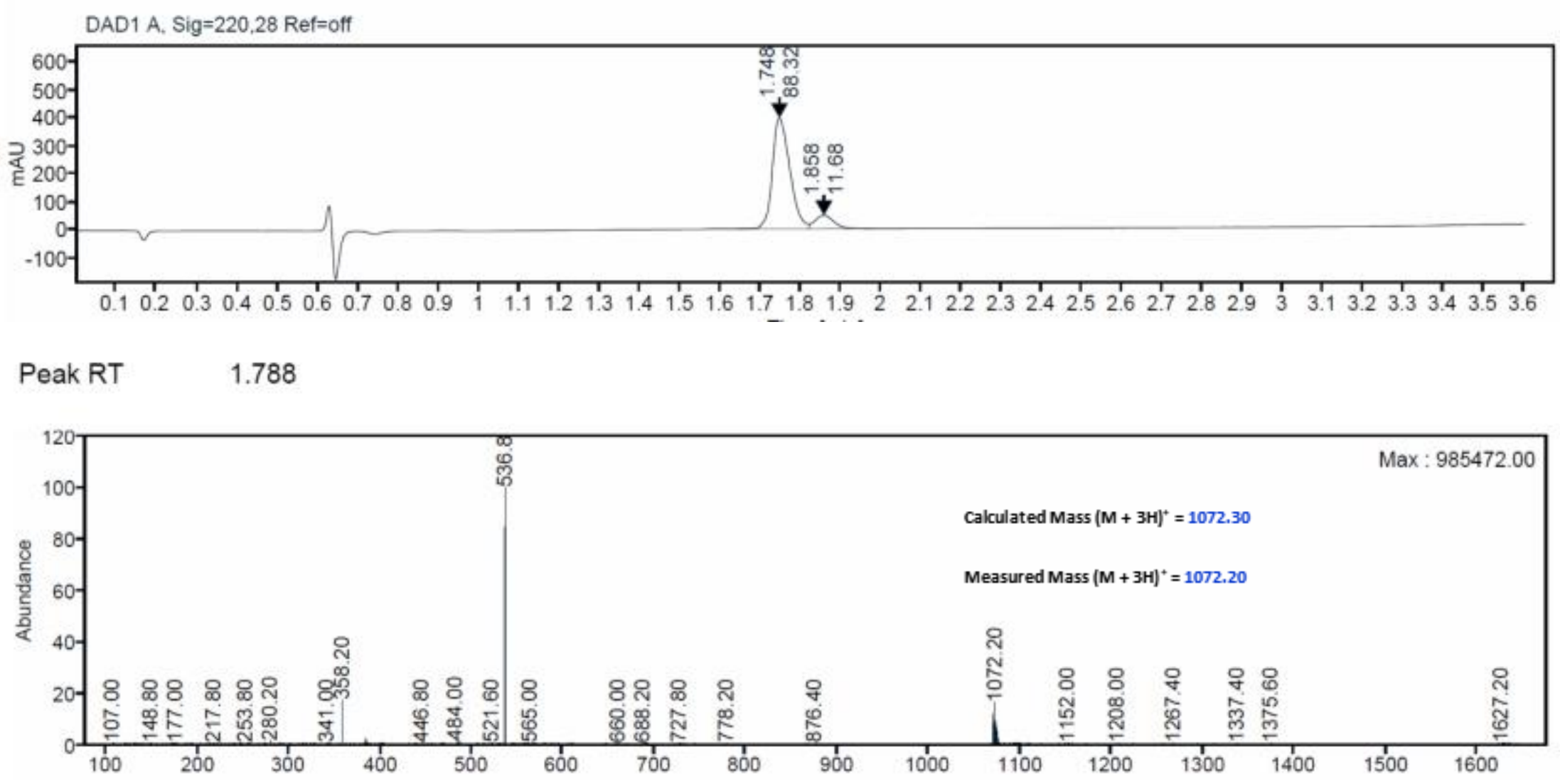

Supplementary Fig. 70 LCMS trace of **a13d**

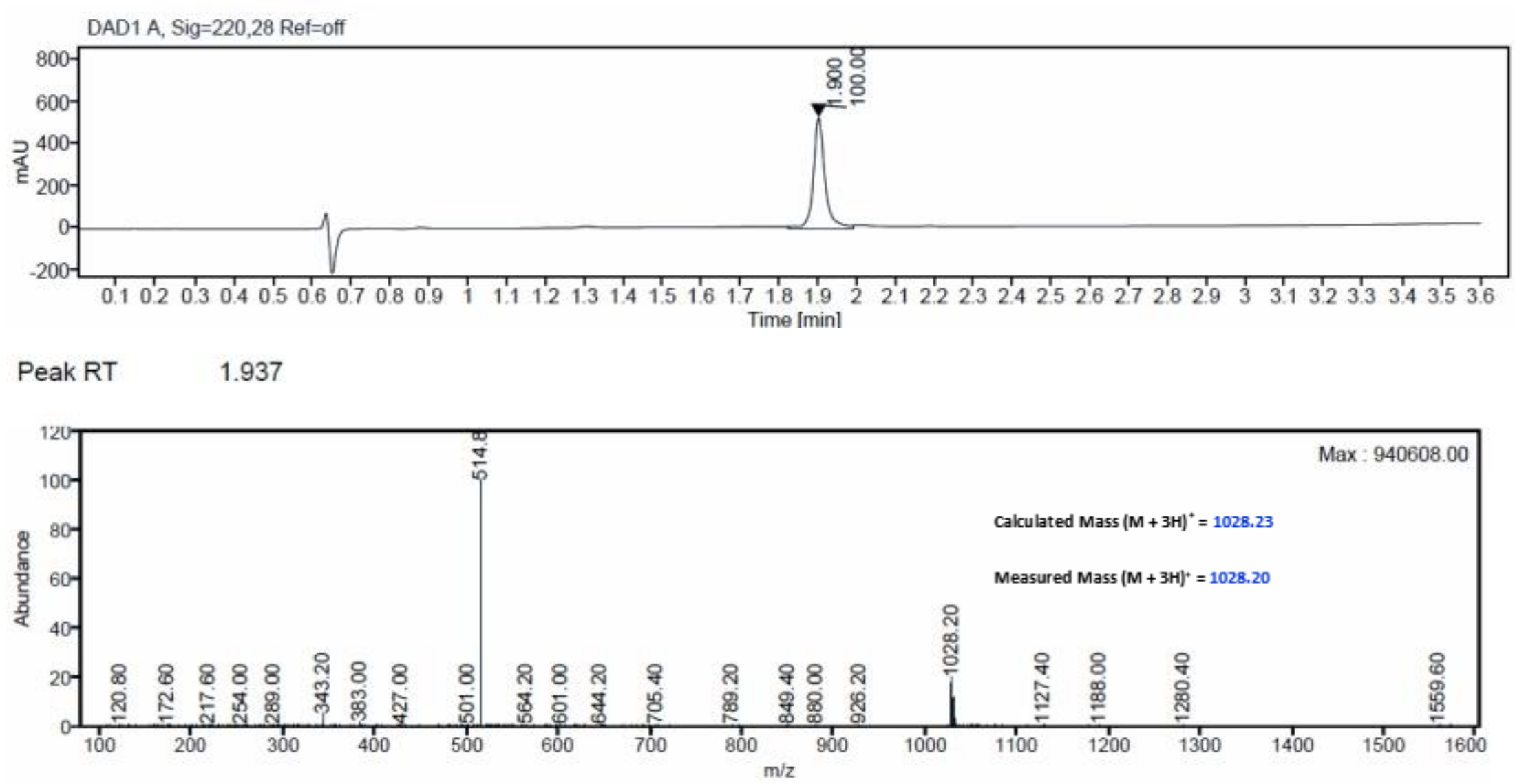

Supplementary Fig. 71 LCMS trace of **a13c**

2. Supplementary Tables

Supplementary Table 1 Characterization of BRD4-FKBP12-bifunctional ligands. Compounds were synthesized by amide coupling of the BRD4 ligand and the linker, followed by copper-catalyzed click reaction between resulting intermediate to the alkyne present in the FKBP12 ligand. Binding to purified BRD4 BD1 or BD2 bromo domains was determined by a competitive fluorescence polarization assay in the absence or presence of 15 µM FKBP12 using 2 nM of a fluorescently labeled JQ1-analog as a tracer. Binding to FKBP12 was determined by a competitive fluorescence polarization assay. NanoBRET data were determined in intact cells by expression of NanoLuc-fusion proteins and using fluorescently labeled FKBP12- or BRD4-ligands. α signifies the binding cooperativity factor calculated as the ratio for binding to BRD4 in the absence or presence of FKBP12.

Compound	BRD4 ligand	Linker	FKBP12 ligand	Binding to FKBP12	Binding to BRD4 BD1 -/+ FKBP12		Binding to BRD4 BD2 -/+ FKBP12		NanoBRET FKBP12	NanoBRET BRD4
				K_d [nM]	K_d [nM]	α	K_d [nM]	α	IC_{50} [nM]	IC_{50} [nM]
JQ1					37±5	1.0	72±13	1.0		
					39±5		64±10			
a1d	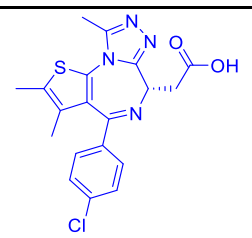	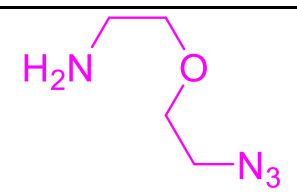	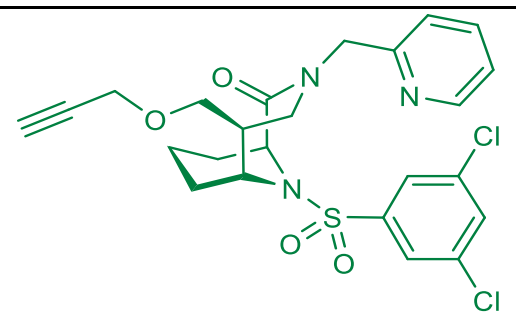	1.0	450±70	18	320±50	4.5	14	820
					25±4		70±13			
a1c	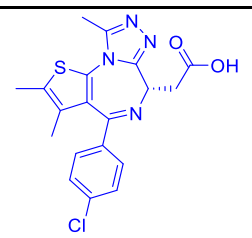	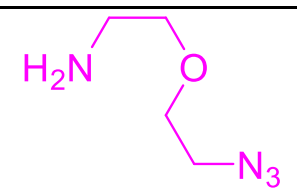	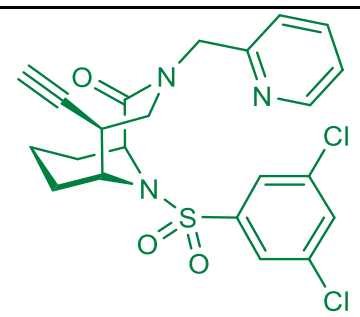	1.0	1300±200	0.3	590±110	0.4	22	940
					3900±500		1500±260			
a2c				0.6	440±70	1.8	510±110	0.5	17	620

	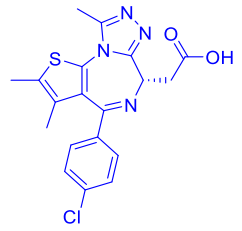	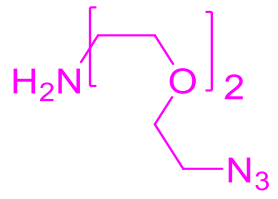	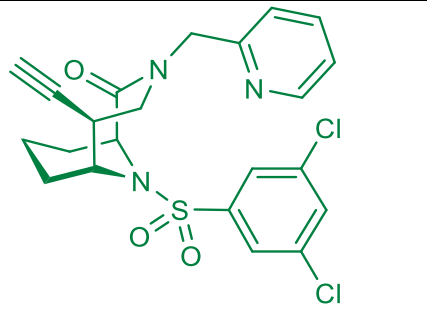		250±40		1100±140			
a1i	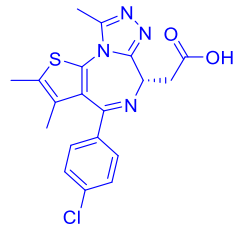	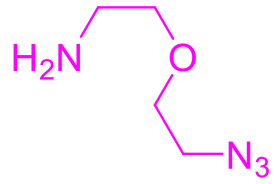	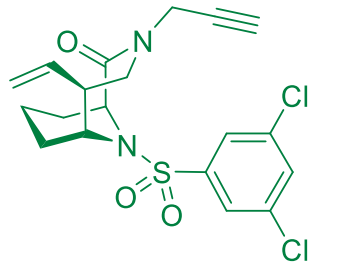	10	1800±270	0.5	910±160	0.3	200	560
					3900±500		2900±400			
a2i	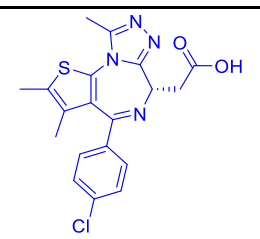	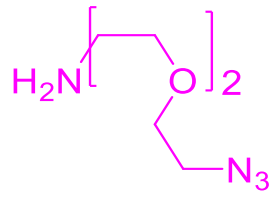	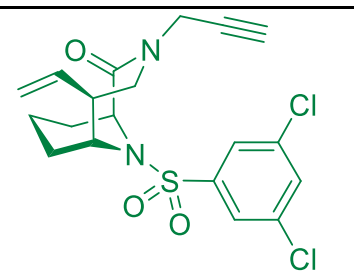	8.7	330±40	1.1	140±30	0.1	180	790
					290±30		960±120			
a1h	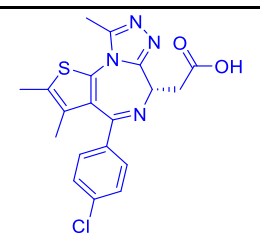	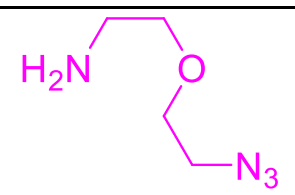	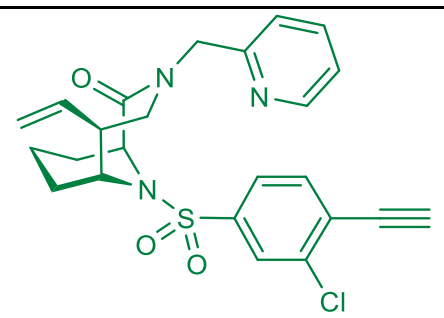	50	100±18	0.06	52±13	0.05	530	270
					1700±200		1100±200			
a2h	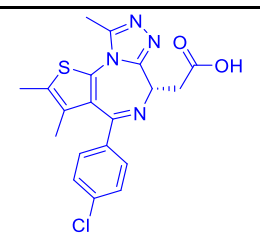	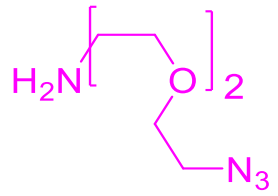	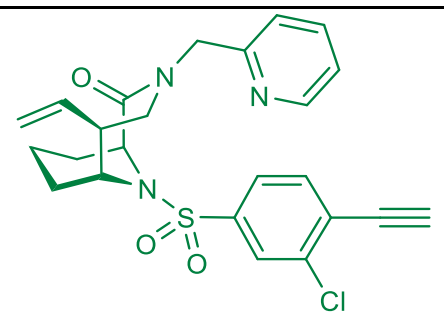	2.4	210±18	0.09	46±10	0.04	170	300
					2400±200		1300±200			
a1g	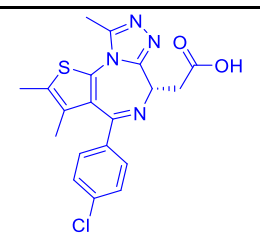	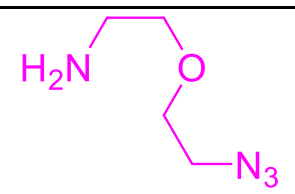	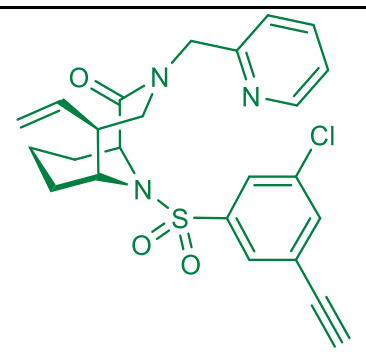	46	2800±500		1700±300	0.2	230	1500
					>20000		9000±2000			
a2g				76	1200±14	0.4	580±90	0.4		3066

	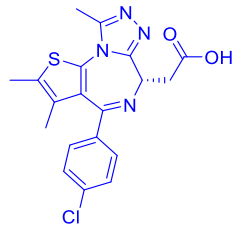	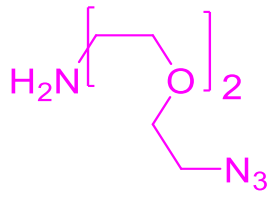	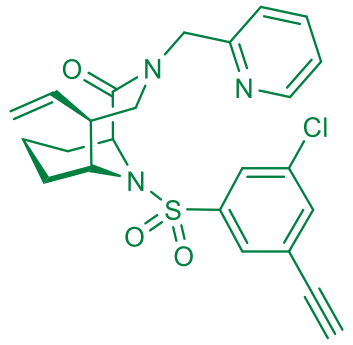		0					
					3100±30<br>0		1500±20<br>0			
a4d	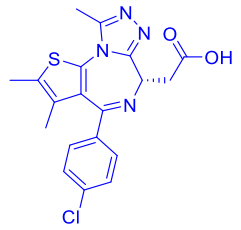	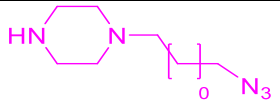	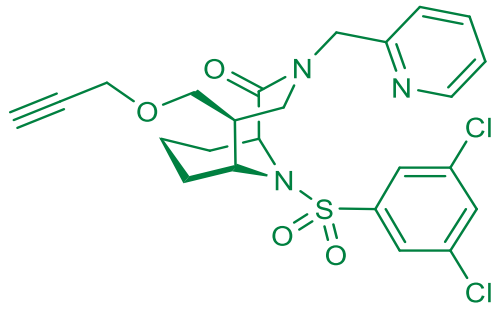	1.1	500±60	1.3	1200±20<br>0	1	89	none
					380±50		1200±10<br>0			
a5d	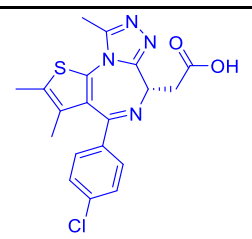	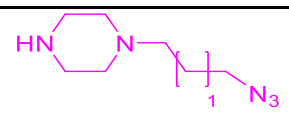	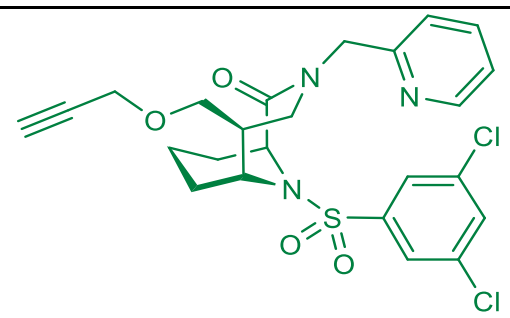	1.2	740±100	2.5	1100±20<br>0	1.8	93	1080
					300±50		600±100			
a6d	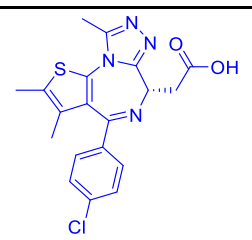	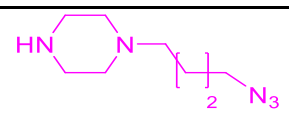	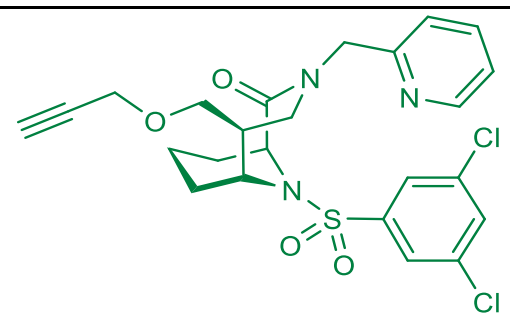	0.9	600±80	1.5	1400±30<br>0	1.8	141	787
					400±50		800±200			
a7c	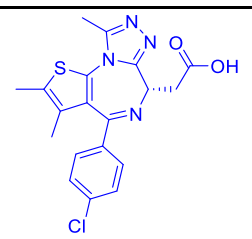	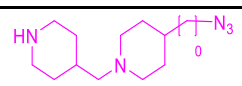	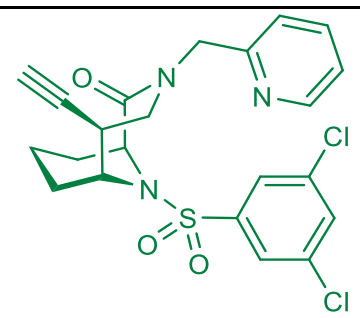	3.5	520±80	2.9	700±100	0.8	138	665
					180±30		900±200			
a7d	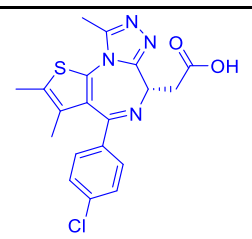	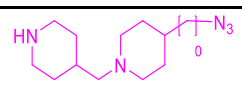	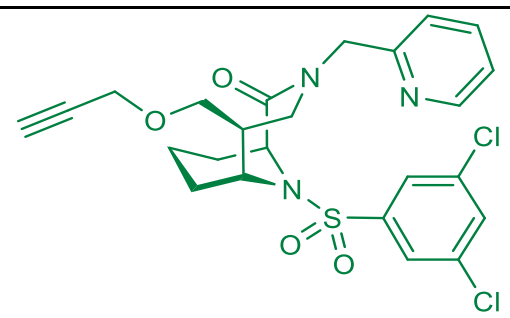	2.3	680±70	2.2	700±100	1	209	1115
					310±50		660±90			
a8c				2.1	170±20	1.1	190±30	1.2	178	541

	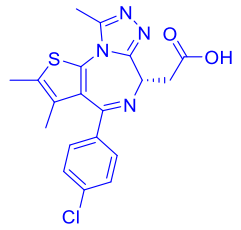	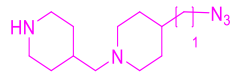	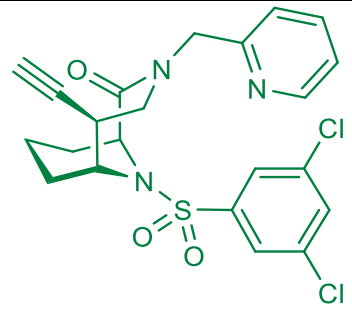		150±20		160±30			
a8d	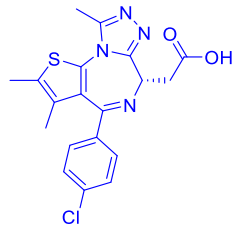	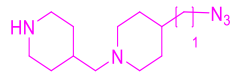	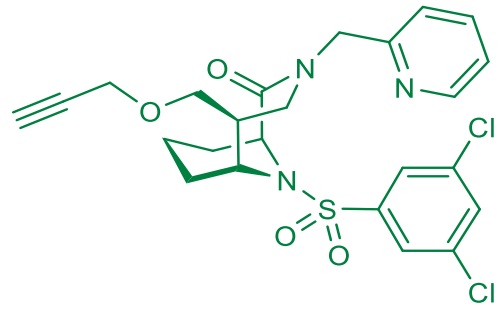	1.8	160±30<br>84±16	1.9	130±30<br>120±30	1	228	357
a15d	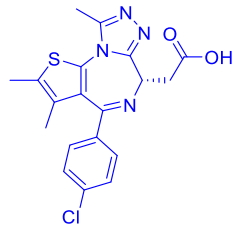	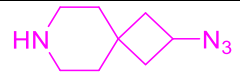	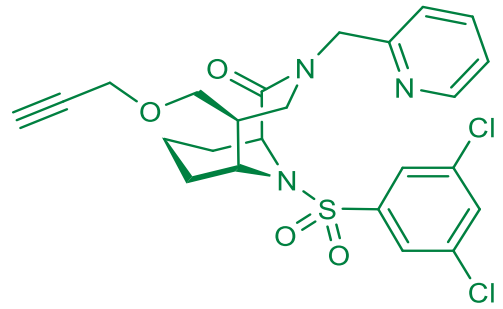	2.6	3000±700<br>420±60	7.1	5400±1300<br>520±80	10	157	5218
a13c	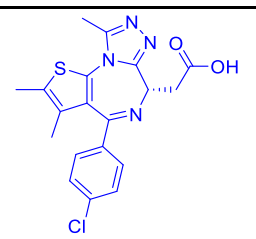	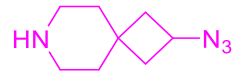	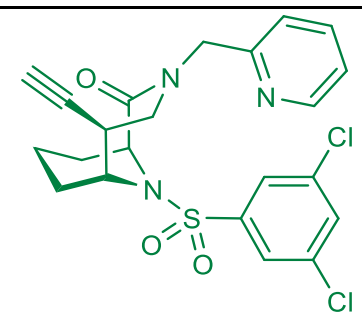	1.7	4600±800<br>35±5	131	2300±400<br>1900±300	1.2	76	9057
a1dj	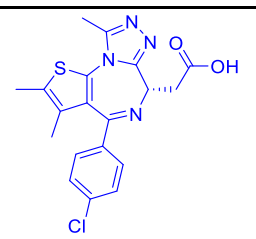	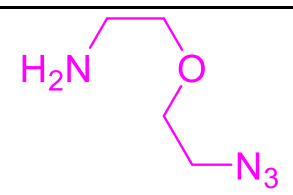	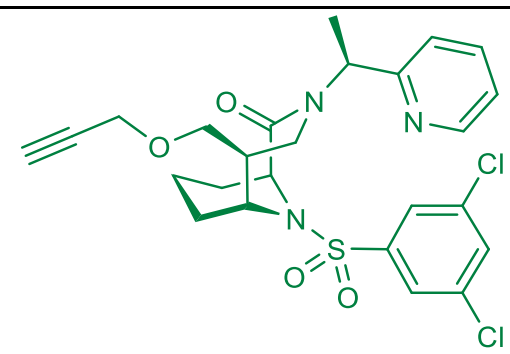	0.4	470±60<br>39±5	12	400±90<br>90±18	4.4	2.6	653
a1dk	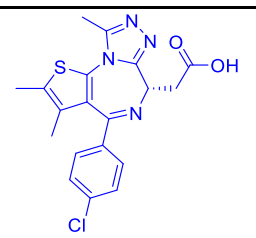	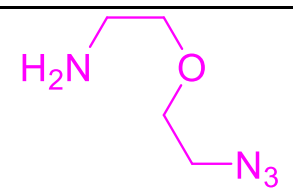	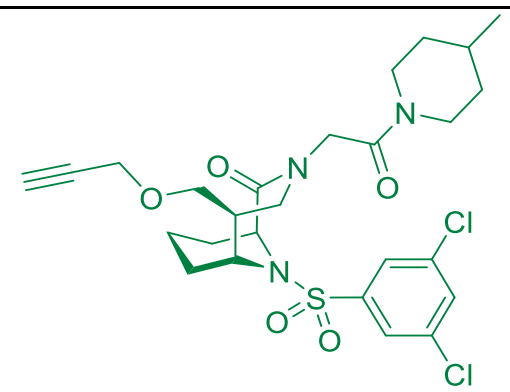	0.47	520±90<br>200±40	2.6	320±60<br>800±100	0.4	9.1	1189
a1e				8.9	70±10	1.5	33±9	0.6	103	390

	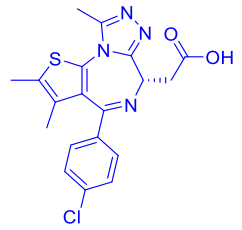	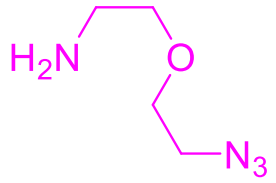	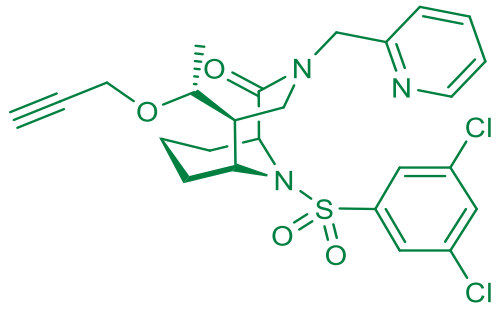		48±7		60±10			
a1f	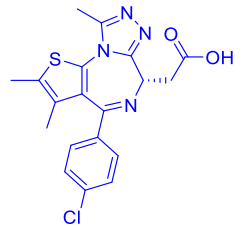	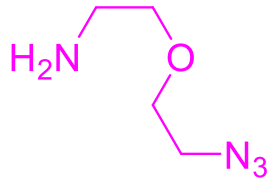	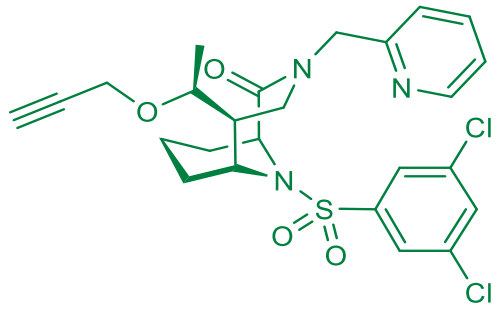	1.6	180±20<br>140±14	1.3	50±14<br>230±30	0.2	24	560
b3d	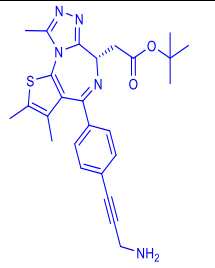	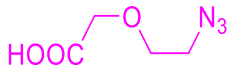	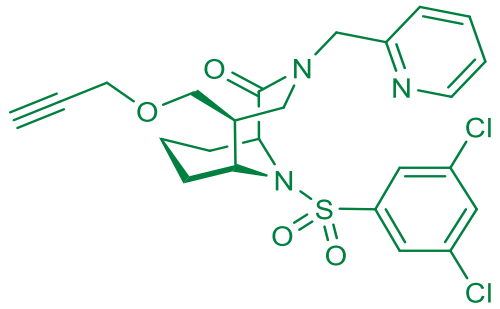	1.0	68±9<br>200±30	0.3	110±30<br>1100±100	0.1	15	266
b3c	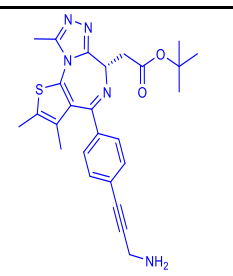	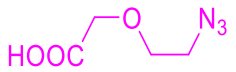	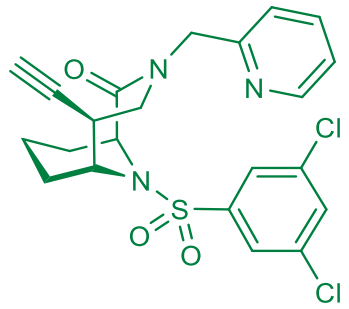	3.7	130±20<br>8±2	16	150±20<br>13±3	12	29	194
a10c	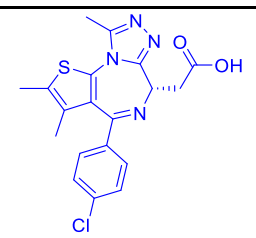	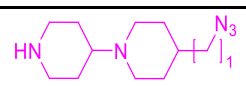	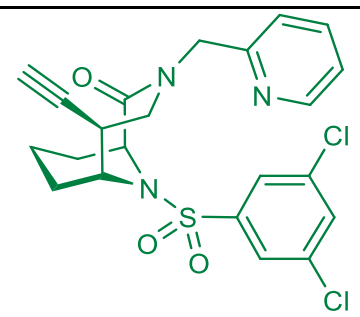	0.3	120±30<br>900±100	0.1	170±30<br>1800±300	0.09	288	497
a10d	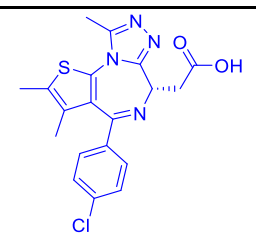	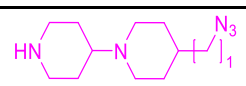	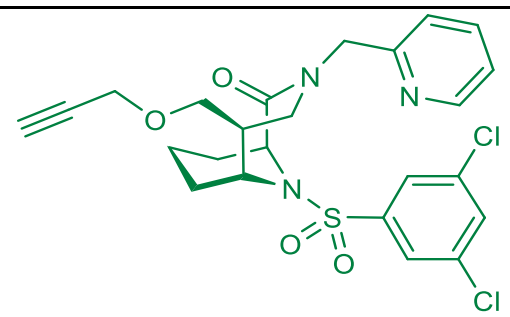	1.6	140±30<br>100±30	1.4	170±30<br>80±20	2.1	241	none
a9c				2.2	280±40	1.6	310±40	0.5	107	155

	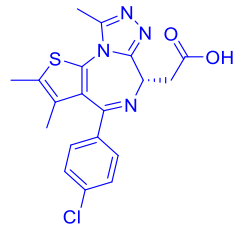	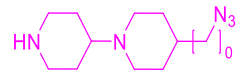	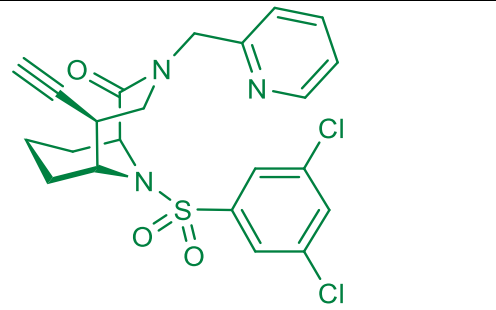		180±20		650±90			
a9d	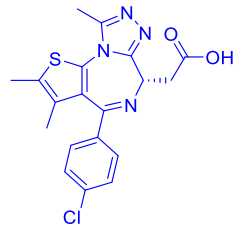	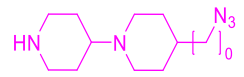	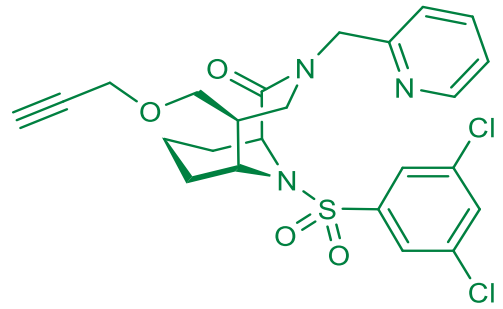	1.7	340±40<br>140±10	2.4	500±90<br>450±70	1.1	169	364
a12c	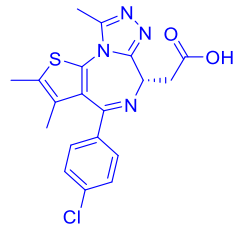	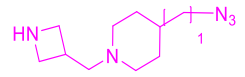	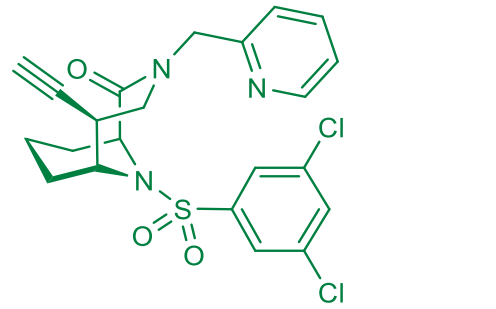	1.8	390±50<br>230±20	1.7	800±100<br>700±90	1.1	280	748
a12d	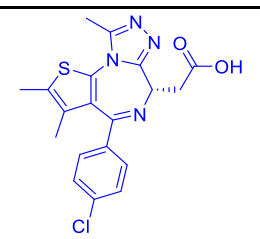	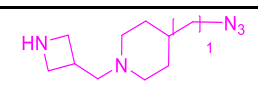	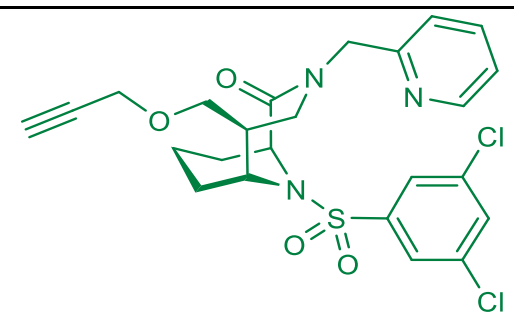	2.3	250±40<br>40±10	6.3	1100±100<br>590±70	1.9	406	514
a11c	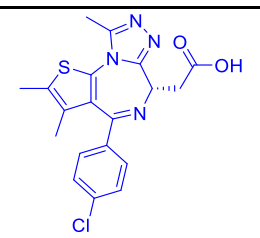	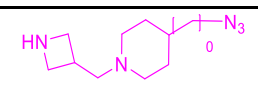	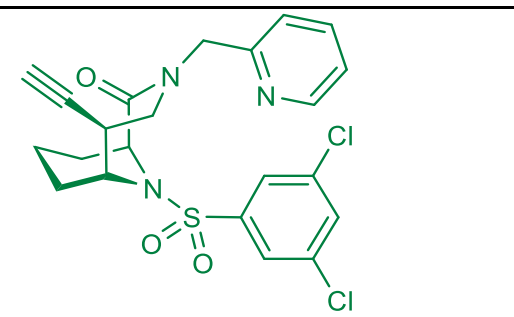	0.7	1000±200<br>190±30	5.3	2100±300<br>450±70	4.7	218	1229
a11d	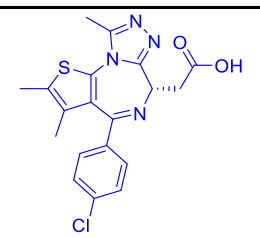	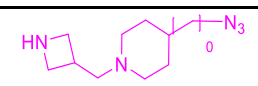	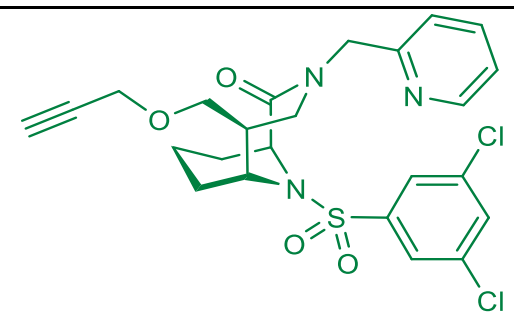	0.8	480±70<br>40±10	12	1250±150<br>700±100	1.8	165	687

Supplementary Table 2 Binding of BRD4-FKBP12 ligand conjugates to purified BRD4 tandem bromo domain contructs. The binding to $BRD4^{43\text{-}463}$, $BRD4^{43\text{-}463\ Y390A}$ and $BRD4^{43\text{-}463\ Y97A}$ was determined in a competitive fluorescence polarization assay in absence or presence of 15 µM FKBP12 using 2 nM of a fluorescently labeled JQ1-analog as a tracer. α signifies the binding cooperativity factor calculated as the ratio for binding to BRD4 in the absence or presence of FKBP12.

	Binding to $BRD4^{43\text{-}463}$ -/+FKBP12		Binding to $BRD4^{43\text{-}463\ Y390A}$ -/+FKBP12		Binding to $BRD4^{43\text{-}463\ Y97A}$ -/+FKBP12	
	K_d [nM]	α	K_d [nM]	α	K_d [nM]	α
JQ1	25±2	1.3	19±2	1.0	32±4	1.2
	19±2		19±2		26±4	
a1d	175±17	6.7	183±13	10	130±16	2.2
	26±3		18±2		59±5	
a1dj	266±16	8.6	106±7	12	100±17	1.2
	31±2		9±1		84±10	
b3c	79±5	5.6	25±4	15	178±20	8.1
	14±2		1.7±0.5		22±3	
a13c	790±120	20	860±140	57	800±110	1.2
	39±3		15±2		670±90	

Supplementary Table 3 Binding of BRD4-FKBP12-bifunctional ligands to BRD4 in presence of FKBP51FK1. Binding of FKBP12-BRD4 ligand conjugates to purified BRD4 BD1 or BD2 bromo domains ($BRD4^{BD1}$ and $BRD4^{BD2}$) was determined in a competitive fluorescence polarization assay in absence or presence of 15 µM FKBP51FK1 using 2 nM of a fluorescently labeled JQ1-analog as a tracer. α signifies the binding cooperativity factor calculated as the ratio for binding to BRD4 in the absence or presence of FKBP12.

	Binding to $BRD4^{BD1}$ -/+FKBP51FK1		Binding to $BRD4^{BD2}$ -/+FKBP51FK1	
	K_d [nM]	α	K_d [nM]	α
JQ1	50±9	1.2	35±12	0.5
	40±7		73±17	
a1d	300±50	0.6	180±30	0.4
	500±90		470±80	
a1dj	250±80	0.4	150±50	0.2
	680±100		1000±500	
b3c	73±20	1.5	120±40	0.5
	50±17		250±70	
a13c	1500±450	1.7	1600±400	8.4
	870±100		190±30	

Supplementary Table 4 Global proteomic analysis of FKBP and BRD4 family members in MV4-11 cells after PROTAC treatment, sorted according to decreasing abundance in control samples.

**Protein Name**	5a1 vs. Control	43 vs. Control
**FKBP12**	**7%**	102%
FKBP3	96%	91%
FKBP4	87%	91%
FKBP2	96%	92%
FKBP8	95%	93%
FKBP5	93%	91%
**BRD4**	124%	**10%**
FKBP15	93%	77%
**BRD3**	129%	**9%**
**BRD2**	114%	**7%**
BRD1	95%	84%
BRD7	89%	77%
BRD8	98%	79%
FKBP7	101%	93%
FKBP9	116%	110%

Supplementary Table 5 Binding of BRD4-FKBP12-bifunctional ligands to BRD2 and BRD3. Binding of FKBP12-BRD4 ligand conjugates to purified BRD2 or BRD3 BD1 or BD2 bromo domains ($BRD2^{BD1}$, $BRD2^{BD2}$, $BRD3^{BD1}$ and $BRD3^{BD2}$) was determined in a competitive fluorescence polarization assay in absence or presence of 15 µM FKBP12 using 2 nM of a fluorescently labeled JQ1-analog as a tracer. α signifies the binding cooperativity factor calculated as the ratio for binding to BRD4 in the absence or presence of FKBP12.

	Binding to $BRD2^{BD1}$ -/+FKBP12		Binding to $BRD2^{BD2}$ -/+FKBP12		Binding to $BRD3^{BD1}$ -/+FKBP12		Binding to $BRD3^{BD2}$ -/+FKBP12	
	K_d [nM]	α	K_d [nM]	α	K_d [nM]	α	K_d [nM]	α
JQ1	34±9	1.0	24±10	1.0	19±4	1.0	33±8	1.0
	35±9		23±7		18±4		36±5	
a1d	260±60	7.2	190±50	2.5	170±27	7.4	150±30	1.0
	36±9		75±20		23±5		140±30	
a1dj	610±90	16	140±65	2.9	290±35	7.6	170±50	1.1
	38±6		48±17		25±3		150±30	
b3c	74±20	8.2	150±50	38	35±7	2.7	100±30	3.8
	9±4		4±2		13±3		26±6	
a13c	3400±1600	34	960±350	1.6	2600±1000	46	950±100	0.3
	100±14		600±100		56±5		3000±350	

Supplementary Table 6: Data collection and refinement statistics for crystal structures of FKBP12 and BRD4/BRD2/BRD3 bromodomain complexes with bifunctional ligands (PDB entries: 9QW8, 9R5N, and 29QL). * Values in parentheses are for highest-resolution shell.

**PDB entry**	**9QW8**	**9R5N**	**29QL**
**Ligand name**	**a1d**	**b3c**	**a1d**
**Protein names**	**FKBP12 & BRD4^{BD1}**	**FKBP12 & BRD4^{BD1}**	**FKBP12 & BRD4^{BD2}**
**Data collection**			
Beamline	ESRF (ID23-1)	BESSY II (BL14.1)	BESSY II (BL14.1)
Wavelength	λ = 0.885603 Å	λ = 0.9184 Å	λ = 0.9184 Å
Space group	P1	$P2_12_12_1$	$P4_32_12$
Cell dimensions			
a, b, c (Å)	35.65, 35.65, 100.92	57.82, 90.51, 103.12	80.42, 80.42, 212.04
α, β, γ (°)	86.46, 84.22, 72.47	90, 90, 90	90, 90, 90
Resolution (Å)*	100.35-1.80 (1.84-1.80)	48.72-3.00 (3.18-3.00)	44.31-2.10 (2.16-2.10)
R_{merge}	0.094 (0.689)	0.306 (0.660)	0.191 (1.151)
R_{pim}	0.061 (0.467)	0.127 (0.269)	0.059 (0.450)
$I/\sigma(I)$	8.4 (1.7)	8.9 (4.3)	12.2 (1.4)
CC1/2	0.977 (0.563)	0.989 (0.936)	0.998 (0.896)
Completeness (%)	97.0 (94.6)	99.9 (100.0)	100.0 (99.9)
Redundancy	3.3 (3.1)	12.5 (13.2)	19. (13.9)
**Refinement**			

Resolution (Å)	100.35-1.80	48.77-3.00	44.30-2.10
No. of reflections	42396	11326	39726
R_{work}/R_{free} (%)	21.3/24.3	25.5/30.7	24.5/27.5
No. of atoms			
Protein	2024/1979/1568/1563	1654/1543/1553/1553	1644/1691/1612/1556
Ligand	110/110	120/120	110/110
Water	256		148
B-factors			
Protein	20.1/21.0/27.7/35.0	32.2/31.3/23.7/23.3	25.8/26.8/19.6/28.1
Ligand	20.1/29.7	24.3/22.1	21.1/21.8
Water	27.2		23.6
R.m.s. deviations			
Bond lengths (Å)	0.0115	0.0194	0.0184
Bond angles (°)	2.163	2.67	3.828
Ramachandran plot			
Favoured (%)	97.0	93.0	99.0
Allowed (%)	3.0	6.0	1.0
Outlier (%)	0.0	1.0	0.0

3. Supplementary Methods

3.1. Structure Solution and refinement

The crystallographic experiments were performed at the BL14.1 at the Helmholtz-Zentrum BESSYII synchrotron, Berlin, Germany[2] and at the beamline ID23-1 of the European Synchrotron Radiation Facility (ESRF) in Grenoble, France and the raw data can be accessed at https://doi.org/10.15151/ESRF-DC-2127908021[3]. Diffraction data were integrated with XDS and further processed with the implemented programs of the CCP4i and CCP4i2 interface[4–8]. The data reduction was conducted with Aimless[7,9,10]. Crystal structures were solved by molecular replacement using Phaser[11]. Iterative model improvement and refinement were performed with Coot and Refmac5[12–17]. The dictionaries for the compounds were generated with AceDRG implemented in CCP4i2[18]. Residues facing solvent channels without detectable side chain density were truncated.

3.2. General Synthesis Remarks

All reactions and manipulations which are sensitive towards air or moisture were performed under dry argon by using standard Schlenk techniques. All chemicals were purchased from Acros Organics, Sigma Aldrich, Alfa Aesar, TCI or ChemPUR. If not indicated otherwise, reagents and solvents were purchased from commercial suppliers and used without further treatment. All reactions were followed by TLC analysis or LC-MS. Flash silica gel column chromatography was performed with a Biotage® Isolera One system with Biotage® Sfär Silica HC D columns. Column chromatography was performed manually with silica gel 60 (0.04–0.063 mm) from Machery Nagel GmbH & Co. KG. Semi-preparative HPLC was performed with an Interchim PuriFlash 5250 system with a Luna® 5 µm C18(2) 100 Å, 250x21.2 mm column from Phenomenex. Eluents were 0.1 % TFA in water (Eluent A) and 0.1 % TFA in acetonitrile (Eluent B), methods are given in percentage B. All compounds were >95 % purity by HPLC if not noted otherwise. Preparative Separation by THAR SFC with Column Chiral Pak IE. Compound purity and low-resolution mass spectra were determined using an Agilent 1260 Infinity II system with a Poroshell 120 EC-C18 1.9 µm, 2.1 x 50 mm column from Agilent. Eluents were 0.1 % formic acid in water (Eluent A) and 0.1 % formic acid in acetonitrile (Eluent B), the used method was 5 % B to 100 % B in 2 min. MS was recorded with an Agilent InfinityLab G6125B LC/MSD. NMR spectroscopy was performed by the NMR department at TU Darmstadt. NMR spectra were recorded either on a 300 MHz Avance II NMR spectrometer from Bruker BioSpin GmbH (for ^{1}H-NMR only), a 300 MHz Avance III NMR

spectrometer from Bruker BioSpin GmbH (for ^{1}H-, ^{13}C-NMR), or a 500 MHz NMR spectrometer DRX 500 from Bruker BioSpin GmbH (for ^{1}H- and ^{13}C-NMR). NMR spectra were recorded at room temperature. The following abbreviations are used in the analysis of NMR spectra: s = singlet, d = doublet, t = triplet, q = quartet, hept = heptet, sept = septet, s_{nr} = broad singlet. Combination of these abbreviations is applied whenever more than one coupling is observed. HRMS was performed by the mass spectrometry department at TU Darmstadt. Mass spectra were recorded on an Impact II, quadrupole-time-of-flight spectrometer from Bruker Daltonics. TLC was performed on TLC Silica gel 60 F254 Aluminum sheets from Merck Millipore. All final test compounds had a purity ≥95 % as determined by HPLC and UV detection at 220 nm.

3.3. Compound Synthesis

General Procedure 1 (GP-1, HATU coupling)

Under Argon atmosphere, the acid (1.0 eq.) and the amine (1.2 eq.) were dissolved in dry DCM [0.05M], then HATU (2.0 eq.) and DIPEA (2.5 eq.) were added, and the reaction mixture was stirred at room temperature. After 16 h, the solution was treated by brine and extracted by DCM. The organic phase was dried over anhydrous Na_2SO_4 and concentrated under reduced pressure.

General Procedure 2 (GP-2, Click reaction**)**

Azid (1.0 eq.) and alkyne (1.0 eq.) were dissolved in DMSO/H_2O (v/v, 8/2), then aq. $CuSO_4$ (0.4 eq.) and sodium ascorbate (0.4 eq.) were added, and the reaction mixture was stirred at room temperature. The crude product was purified by prep. HPLC.

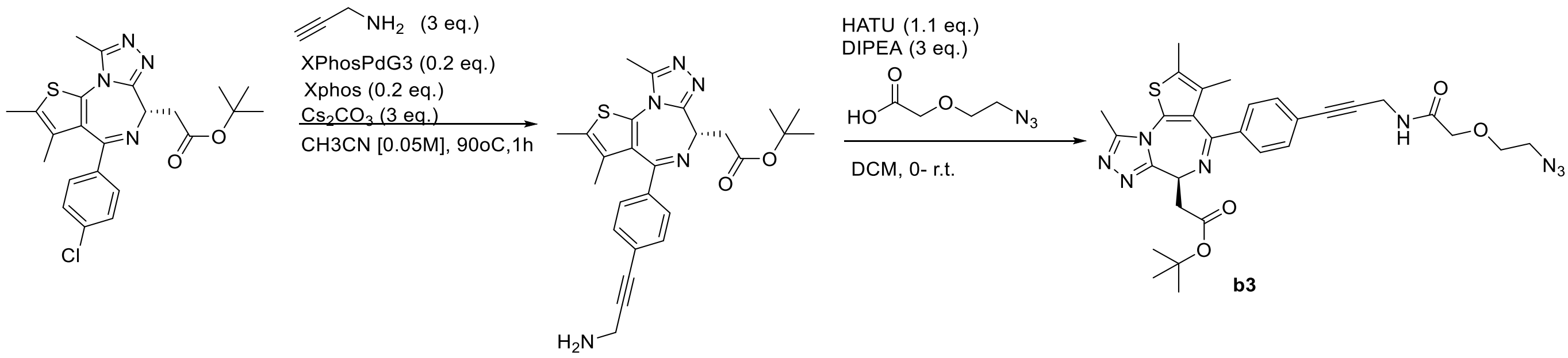

tert-butyl (*S*)-2-(4-(4-(3-aminoprop-1-yn-1-yl)phenyl)-2,3,9-trimethyl-6H-thieno[3,2-f][1,2,4]triazolo[4,3-a][1,4]diazepin-6-yl)acetate[19]

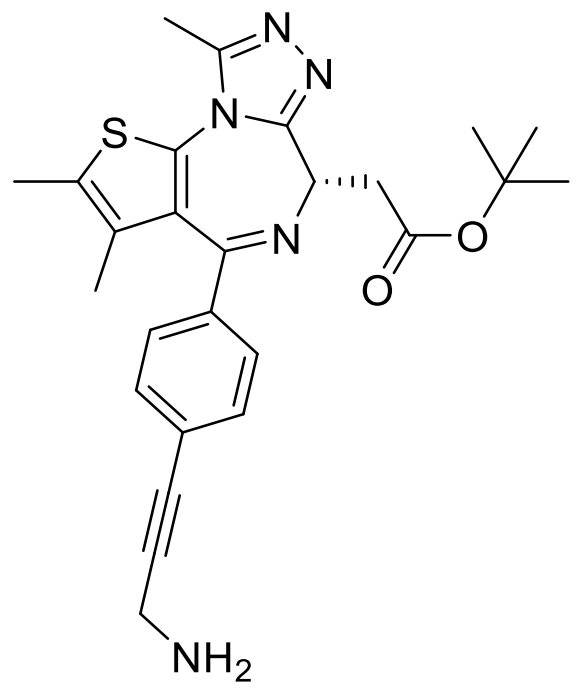

Under dry and argon atmosphere, JQ1 (228 mg, 0.5 mmol, 1.00 eq.), XPhos Pd G3 (85 mg, 0.1 mmol, 0.2 eq.), XPhos ligand (50 mg, 0.1 mmol, 0.2 eq.), propargylamine (83 mg, 1.5 mmol, 3.00 eq.) and Cs_2CO_3 (490 mg, 1.5 mmol, 3.00 eq.) were mixed in dry CH_3CN. The vial was capped and heated for 3h at 90 ºC to yield the title compound.

After completion of the reaction, the mixture was filtered through a pad of celite and purified by flash column chromatography on silica gel (0-100% MeOH in DCM). The obtained product was further purified by prep. HPLC (5-100% B), neutralized by $NaHCO_3$, extracted by *i*PrOH/$CHCl_3$ – 1/4, dried by lyophilization.

Yield: 28 mg (0.06 mmol, 12%).

Physical State: yellow solid.

R_f Value: 0.38 (DCM/MeOH – 10/1).

^{1}H NMR (500 MHz, $CDCl_3$) δ 7.39 (d, *J* = 2.2 Hz, 4H), 4.55 (dd, *J* = 7.7, 6.4 Hz, 1H), 3.65 (s, 2H), 3.54 (dd, *J* = 7.0, 1.8 Hz, 2H), 2.65 (s, 3H), 2.39 (s, 3H), 1.66 (s, 3H), 1.49 (s, 9H) ppm.

^{13}C NMR (126 MHz, $CDCl_3$) δ 170.85, 164.11, 155.55, 149.79, 137.69, 132.20, 131.61, 130.94, 130.56, 130.54, 128.40, 125.42, 92.26, 82.06, 80.91, 53.94, 37.87, 32.19, 28.17, 14.33, 13.09, 11.87. ppm.

HRMS (ESI, m/z) calcd. for $C_{26}H_{30}N_5O_2S$: 476.21147, found: 476.21157.

tert-butyl (*S*)-2-(4-(4-(3-(2-(2-azidoethoxy)acetamido)prop-1-yn-1-yl)phenyl)-2,3,9-trimethyl-6H-thieno[3,2-f][1,2,4]triazolo[4,3-a][1,4]diazepin-6-yl)acetate **b3**

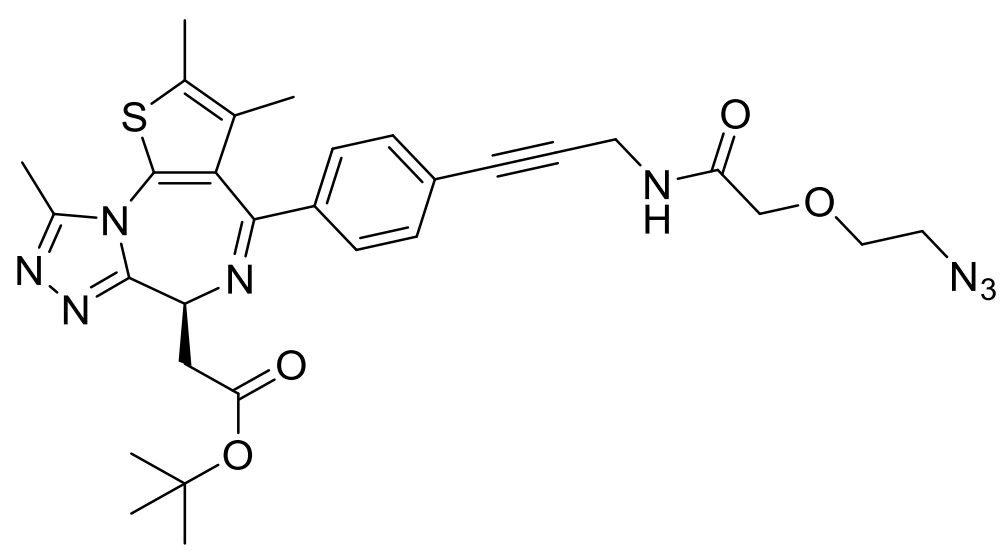

According to **GP-1**, the title compound **b3** was synthesized from *tert*-butyl (*S*)-2-(4-(4-(3-aminoprop-1-yn-1-yl)phenyl)-2,3,9-trimethyl-6H-thieno[3,2-f][1,2,4]triazolo[4,3-a][1,4]diazepin-6-yl)acetate[19](27 mg, 0.05 mmol, 1.00 eq.) and 2-(2-azidoethoxy)acetic acid (15 mg, 0.1 mmol, 2.00 eq.). The obtained product was further purified by prep. HPLC (5-100 % B).

Yield: 15 mg (0.025 mmol, 50%).
Physical State: yellow solid.
^{1}H NMR (500 MHz, $CDCl_3$)δ 7.41 (s, 4H), 6.90 (t, *J* = 5.3 Hz, 1H), 4.57 (dd, *J* = 7.9, 6.2 Hz, 1H), 4.33 (d, *J* = 5.4 Hz, 2H), 4.06 (s, 2H), 3.72 (dd, *J* = 5.4, 4.2 Hz, 2H), 3.55 – 3.52 (m, 2H), 3.50 – 3.45 (m, 2H), 2.70 (s, 3H), 2.41 (s, 3H), 1.66 (s, 3H), 1.49 (s, 9H).
^{13}C NMR (126 MHz, $CDCl_3$) δ 170.70, 168.80, 164.21, 155.43, 149.91, 137.86, 131.85, 131.72, 131.07, 131.05, 130.86, 128.42, 124.86, 86.52, 82.92, 81.08, 70.37, 70.34, 53.85, 50.66, 37.72, 29.49, 28.16, 14.34, 13.13, 11.67.
HRMS (ESI, m/z) calcd. for $C_{30}H_{35}N_8O_4S$ [M+H$^+$]: 603.24965, found: 603.24965.

tert-butyl 2-((6*S*)-4-(4-(3-(2-(2-(4-((((5*S*)-10-((3,5-dichlorophenyl)sulfonyl)-2-oxo-3-(pyridin-2-ylmethyl)-3,10-diazabicyclo[4.3.1]decan-5-yl)methoxy)methyl)-1H-1,2,3-triazol-1-yl)ethoxy)acetamido)prop-1-yn-1-yl)phenyl)-2,3,9-trimethyl-6H-thieno[3,2-f][1,2,4]triazolo[4,3-a][1,4]diazepin-6-yl)acetate **b3d**

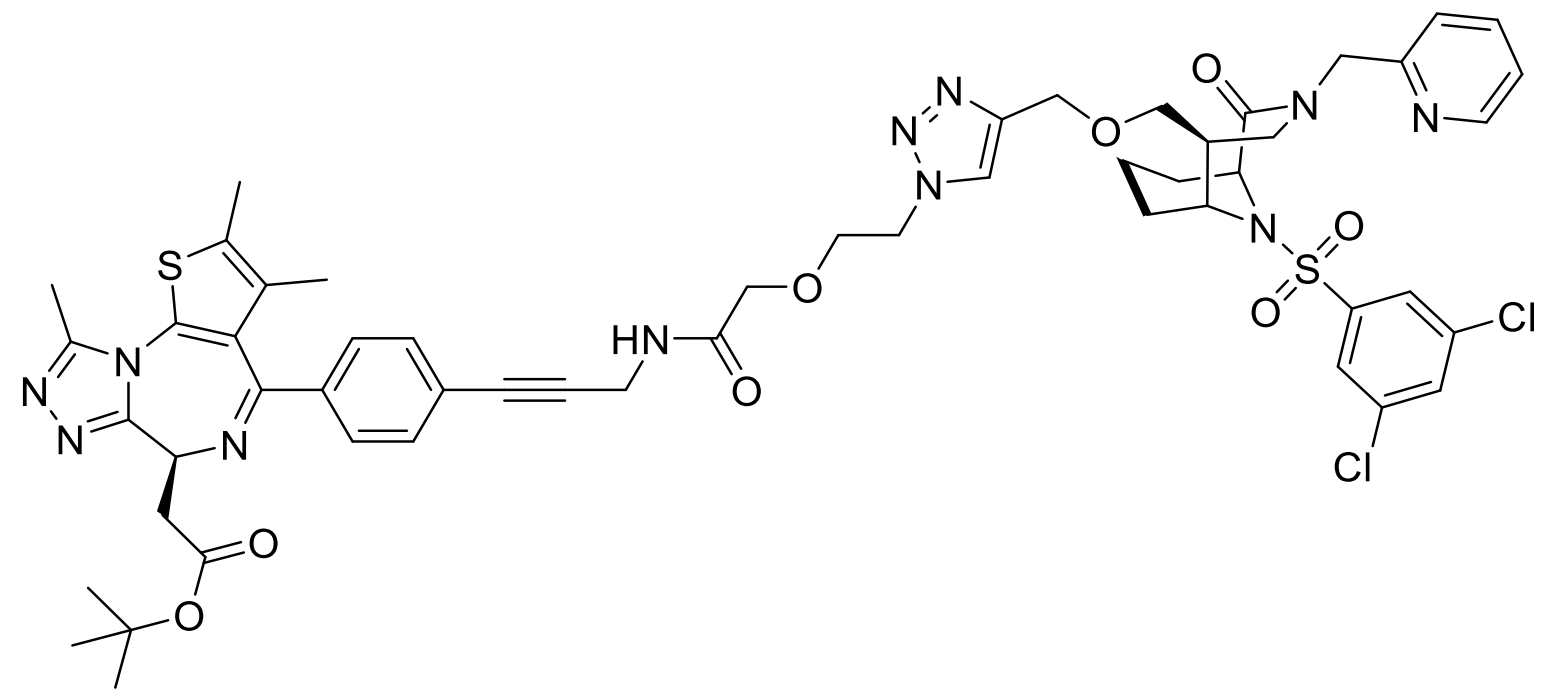

According to **GP-2**, the title compound **b3d** was synthesized synthesized from compound **b3** (6 mg, 10 μmol, 1.00 eq.) and (1S,5S,6R)-10-((3,5-dichlorophenyl)sulfonyl)-5-((prop-2-yn-1-yloxy)methyl)-3-(pyridin-2-ylmethyl)-3,10-diazabicyclo[4.3.1]decan-2-one[20] (5.2 mg, 10 μmol, 1.00 eq.). The obtained product was further purified by prep. HPLC (5-100 % B).

Yield: 4.5 mg (4 μmol, 40%).
Purity: 90 % (HPLC, UV-absorption 220 nm)
Physical State: yellow solid.

HRMS (ESI, m/z) calcd. for $C_{54}H_{61}Cl_2N_{11}O_8S_2$ [M+H$^+$]:1124.34393, found: 1124.34552.

tert-butyl 2-((6*S*)-4-(4-(3-(2-(2-(4-((5*S*)-10-((3,5-dichlorophenyl)sulfonyl)-2-oxo-3-(pyridin-2-ylmethyl)-3,10-diazabicyclo[4.3.1]decan-5-yl)-1H-1,2,3-triazol-1-yl)ethoxy)acetamido)prop-1-yn-1-yl)phenyl)-2,3,9-trimethyl-6H-thieno[3,2-f][1,2,4]triazolo[4,3-a][1,4]diazepin-6-yl)acetate **b3c**

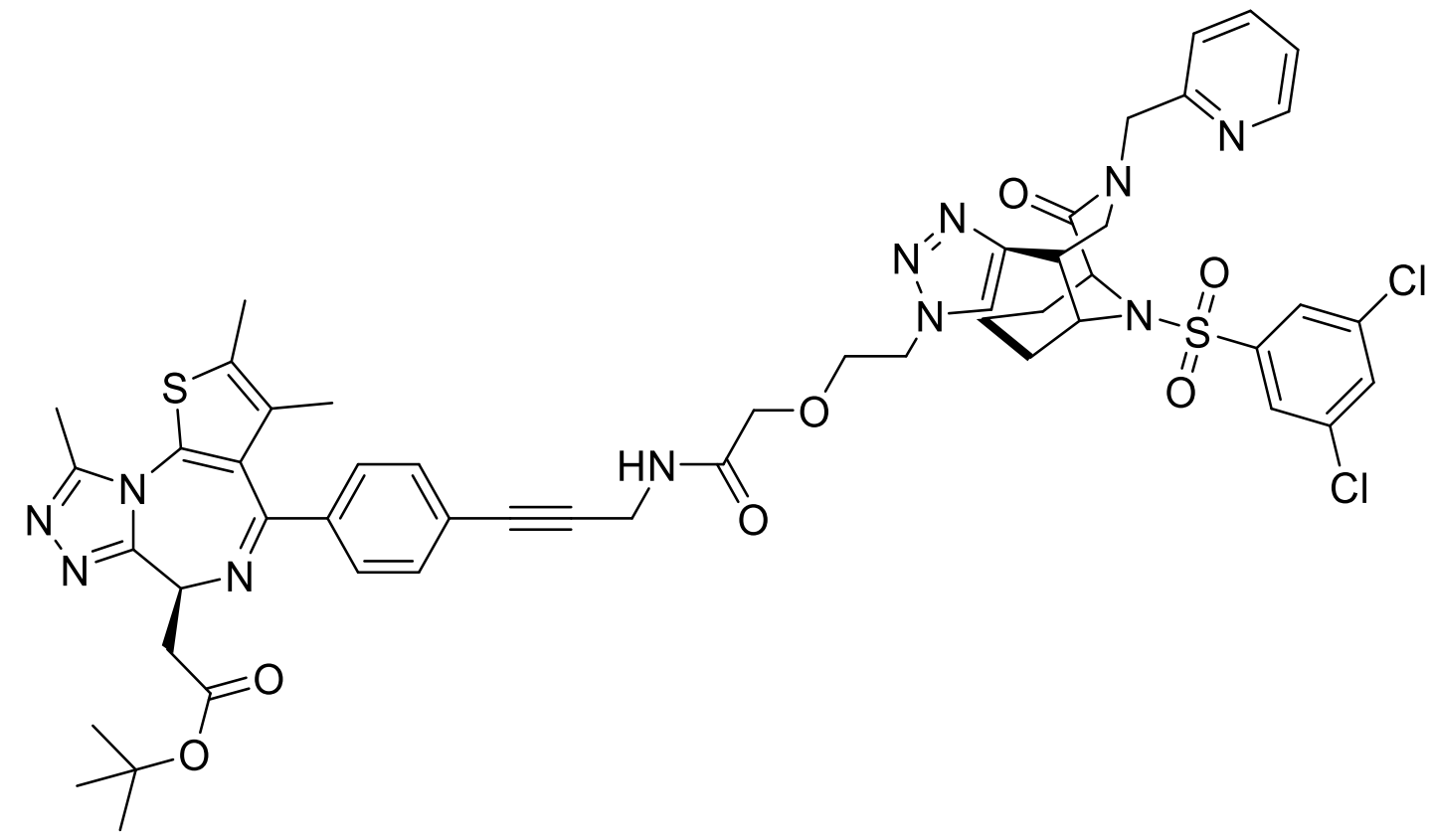

According to **GP-2**, the title compound **b3c** was synthesized from compound **b3** (6 mg, 10 µmol, 1.00 eq.) and *(1S,5S,6R)*-10-((3,5-dichlorophenyl)sulfonyl)-5-ethynyl-3-(pyridin-2-ylmethyl)-3,10-diazabicyclo[4.3.1]decan-2-one[20] (4.8 mg, 10 µmol, 1.00 eq.). The obtained product was further purified by prep. HPLC (5-100 % B).

Yield: 5.4 mg (4 µmol, 50%).
Purity: 99 % (HPLC, UV-absorption 220 nm)
Physical State: yellow solid.
HRMS (ESI, m/z) calcd. for $C_{52}H_{56}Cl_2N_{11}O_7S_2$ [M+H$^+$]:1080.31772, found: 1080.31935.
^{1}H NMR (500 MHz, DMSO) δ 8.56 (d, *J* = 4.4 Hz, 1H), 8.31 (t, *J* = 5.7 Hz, 1H), 8.11 – 7.96 (m, 4H), 7.89 (t, *J* = 7.2 Hz, 1H), 7.47 – 7.29 (m, 5H), 4.86 (d, *J* = 5.5 Hz, 1H),4.80 – 4.75 (m, 2H), 4.57 (t, *J* = 5.1 Hz, 2H), 4.50 – 4.48 (m, 1H) , 4.43 (dd, *J* = 8.1, 6.3 Hz, 1H), 4.15 (d, *J* = 5.4 Hz, 3H), 4.12 – 4.01 (m, 3H), 3.74 – 3.61 (m, 3H), 3.44 – 3.22 (m, 3H), 2.60 (s, 3H), 2.42 (s, 3H), 2.07 (d, *J* = 10.9 Hz, 1H), 1.63 - 1.60 (m, 4H), 1.41 (s, 9H), 1.40 – 1.12 (m, 4H).
^{13}C NMR (126 MHz, DMSO) δ 169.9, 169.7, 168.7, 163.4, 158.3, 158.0, 156.3, 154.7, 149.8, 148.0, 146.8, 143.7, 137.4, 135.5, 132.8, 132.1, 131.4, 130.8, 129.8, 129.6, 128.5, 125.1, 124.3, 122.8, 122.4, 121.7, 89.2, 80.8, 80.2, 69.5, 69.2, 56.1, 55.1, 54.8, 53.6, 52.2, 41.0, 37.4, 28.3, 27.7, 27.6, 27.1, 26.6, 14.7, 13.8, 12.6, 11.2.

(*S*)-2-(4-(4-chlorophenyl)-2,3,9-trimethyl-6H-thieno[3,2-f][1,2,4]triazolo[4,3-a][1,4]diazepin-

6-yl)acetic acid

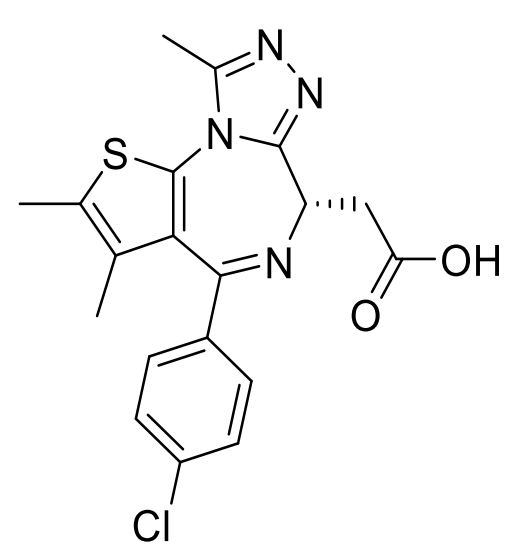

(+)-JQ1 (99 mg, 217 µmol, 1.0 eq.) was dissolved in formic acid / DCM (3 mL / 3 mL) and stirred at room temperature. After 2 hours, the solvent was evaporated *in vacuo* to afford the title compound.

Yield: 86 mg (99 %)

Purity: 94 % (HPLC, UV-absorption 220 nm)

Physical State: yellow solid

TLC: R_f = 0.11 (EA + 1 % FA)

^{1}H-NMR (500 MHz, $CDCl_3$) δ 7.42 (d, 2H, J = 8.5 Hz), 7.32 (d, 2H, J = 8.6 Hz), 4.60 (t, 1H, J = 6.9 Hz), 3.70 (dd, 1H, J = 16.9/6.8 Hz), 3.60 (dd, 1H, J = 16.9/7.2 Hz), 2.68 (s, 3H), 2.40 (s, 3H), 1.68 (s, 3H) ppm.

^{13}C-NMR (125 MHz, $CDCl_3$): δ 174.1, 164.2, 155.4, 150.1, 137.0, 136.5, 132.0, 131.3, 131.1, 130.7, 130.1, 128.8, 53.8, 36.9, 14.5, 13.2, 11.8 ppm.

(*S*)-N-((1-(2-(2-(2-(4-(4-chlorophenyl)-2,3,9-trimethyl-6H-thieno[3,2-f][1,2,4]triazolo[4,3-a][1,4]diazepin-6-yl)acetamido)ethoxy)ethyl)-1H-1,2,3-triazol-4-yl)methyl)-3',6'-dihydroxy-3-oxo-3H-spiro[isobenzofuran-1,9'-xanthene]-5-carboxamide

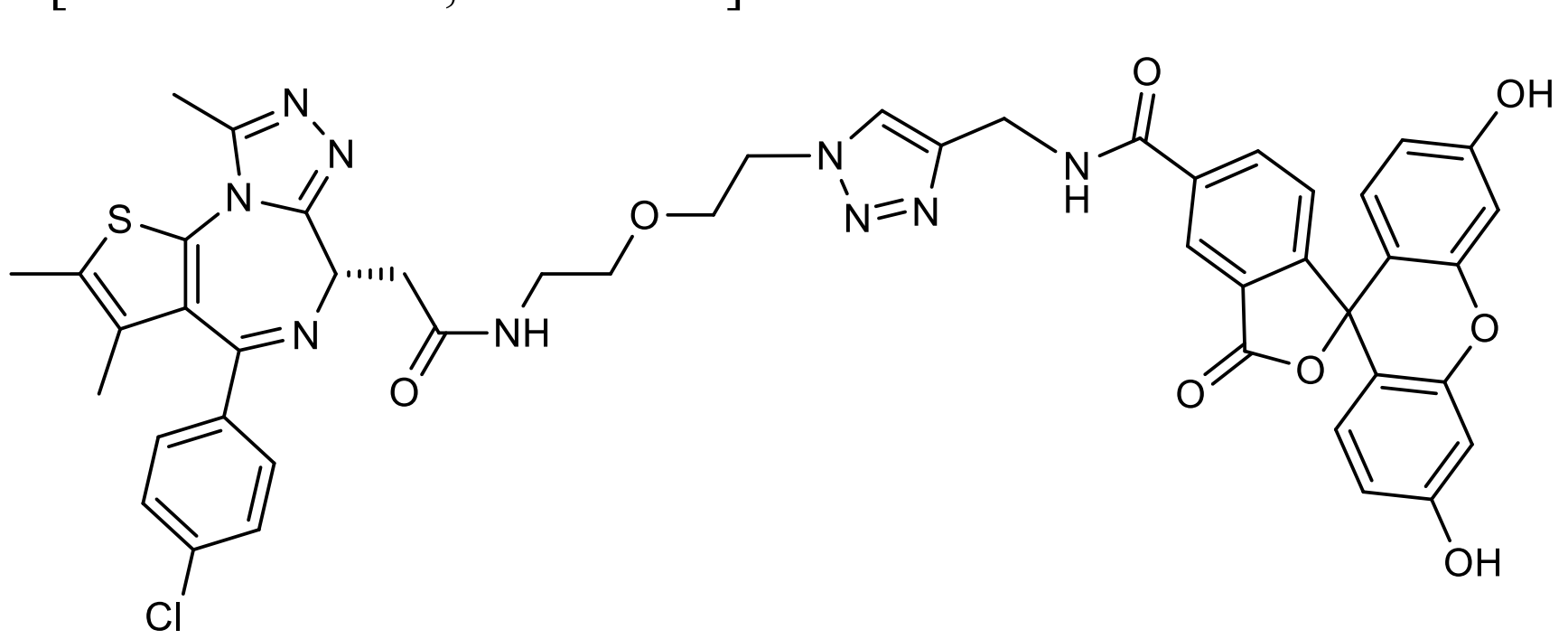

Compound **a1** (6.1 mg, 11.9 µmol, 1.0 eq.) and 3',6'-Dihydroxy-3-oxo-*N*-(prop-2-yn-1-yl)-3*H*-spiro[isobenzofuran-1,9'-xanthene]-5-carboxamide (5.0 mg, 12.1 µmol, 1.0 eq.) were dissolved in DMSO (1 mL), then ^{t}BuOH (100 µL), H_2O (100 µL), aq. $CuSO_4$ (1 M, 4.8 µL, 4.8 µmol, 0.4

eq.) and aq. sodium ascorbate (1 M, 4.8 µL, 4.8 µmol, 0.4 eq.) were added and the reaction mixture was stirred at room temperature. After 18 h, additional aq. $CuSO_4$ (1 M, 10 µL, 10 µmol, 0.8 eq.) and aq. sodium ascorbate (1 M, 10 µL, 10 µmol, 0.8 eq.) were added and the reaction mixture was stirred for another 4 h. Then brine was added and it was extracted with DCM, the organic phase was dried over $MgSO_4$, filtered and concentrated *in vacuo*. The crude product was purified by semi-preparative HPLC (20-70B, 9 CV) to afford the title compound.

Yield: 6.7 mg (61 %)
Purity: >99 % (HPLC, UV-absorption 220 nm)
Physical State: yellow solid
HR-MS (ESI): m/z calculated: $[M+H]^+$ = 926.24818, found: $[M+H]^+$ = 926.24934

(*S*)-*N*-(2-(2-azidoethoxy)ethyl)-2-(4-(4-chlorophenyl)-2,3,9-trimethyl-6*H*-thieno[3,2-*f*][1,2,4]triazolo[4,3-*a*][1,4]diazepin-6-yl)acetamide **a1**

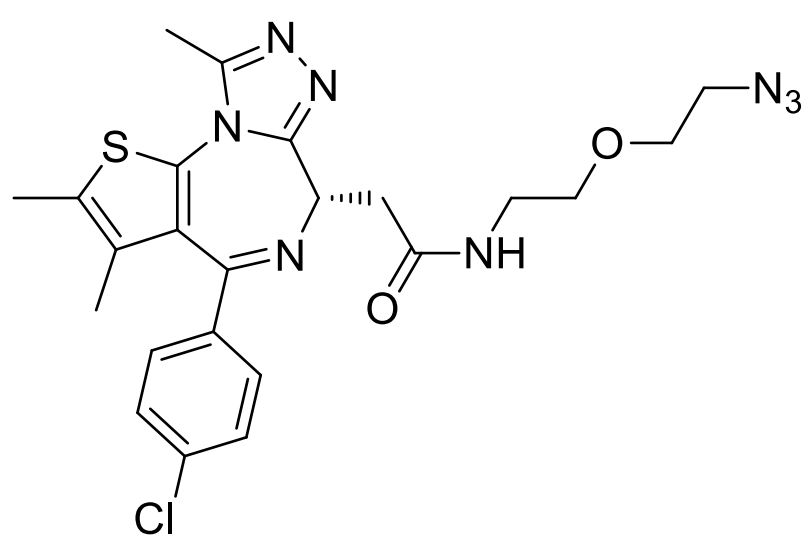

JQ1 acid (40 mg, 99.8 µmol, 1.0 eq.) and H_2N-PEG_1-N_3 (15.7 mg, 120 µmol, 1.2 eq.) were dissolved in dry DCM (2 mL), then HATU (77 mg, 202 µmol, 2.0 eq.) and DIPEA (42 µL, 247 µmol, 2.5 eq.) were added and the reaction mixture was stirred at room temperature. After 16 h, the solvent was evaporated *in vacuo* and the crude product was purified by semi-preparative HPLC (30-70B over 9 CV) to afford the title compound **a1**.

Yield: 54 mg (quant.)
Purity: 99 % (HPLC, UV-absorption 220 nm)
Physical State: yellow solid
MS (ESI): m/z calculated: $[M+H]^+$ = 513.2, found: $[M+H]^+$ = 513.2

^{1}H-NMR (500 MHz, $CDCl_3$): δ = 1.68 (s, 3H), 2.41 (s, 3H), 2.77 (s, 3H), 3.30-3.43 (m, 3H), 3.43-3.72 (m, 7H), 4.70 (t, 1H, J = 7.0 Hz), 7.02-7.10 (m, 1H), 7.34 (d, 2H, J = 8.6 Hz), 7.40 (d, 2H, J = 8.5 Hz) ppm.
^{13}C-NMR (125 MHz, $CDCl_3$): δ = 11.3, 13.2, 14.4, 38.2, 39.7, 50.7, 53.9, 69.5, 70.1, 128.9, 130.1, 130.9, 131.3, 131.5, 132.6, 135.8, 137.6, 150.4, 155.2, 164.9, 170.7 ppm.

(*S*)-*N*-(-(2-(2-(2-azidoethoxy)ethoxy)ethyl)-2-(4-(4-chlorophenyl)-2,3,9-trimethyl-6*H*-thieno[3,2-*f*][1,2,4]triazolo[4,3-*a*][1,4]diazepin-6-yl)acetamide **a2**

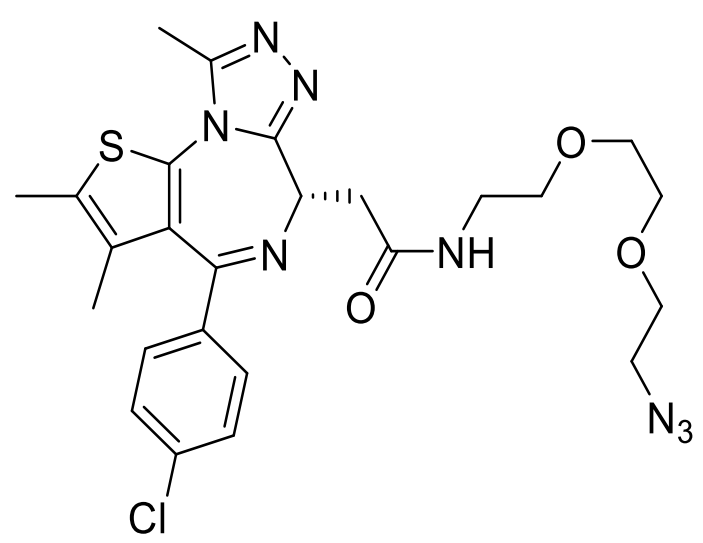

JQ1 acid (40 mg, 99.8 µmol, 1.0 eq.) and H_2N-PEG_2-N_3 (21.1 mg, 121 µmol, 1.2 eq.) were dissolved in dry DCM (2 mL), then HATU (77 mg, 202 µmol, 2.0 eq.) and DIPEA (42 µL, 247 µmol, 2.5 eq.) were added and the reaction mixture was stirred at room temperature. After 16 h, the solvent was evaporated *in vacuo* and the crude product was purified by semi-preparative HPLC (30-70B over 9 CV) to afford the title compound **a2**.

Yield: 43 mg (77 %)
Purity: >99 % (HPLC, UV-absorption 220 nm)
Physical State: yellow solid
MS (ESI): m/z calculated: $[M+H]^+$ = 557.2, found: $[M+H]^+$ = 557.2
^{1}H-NMR (500 MHz, $CDCl_3$): δ = 1.69 (s, 3H), 2.44 (s, 3H), 2.82 (s, 3H), 3.37-3.42 (m, 2H), 3.46 (m, 1H, J = 15.0/6.9 Hz), 3.49-3.73 (m, 11H), 4.78 (t, 1H, J = 7.0 Hz), 7.26-7.31 (m, 1H), 7.36 (d, 2H, J = 8.7 Hz), 7.41 (d, 2H, J = 8.5 Hz) ppm.
^{13}C-NMR (125 MHz, $CDCl_3$): δ = 11.2, 13.3, 14.4, 37.7, 40.0, 50.7, 53.6, 69.4, 70.1, 70.5, 70.6, 129.1, 130.3, 131.1, 131.2, 131.8, 133.3, 135.0, 138.2, 150.7, 154.9, 165.7, 170.9 ppm.

2-((*S*)-4-(4-chlorophenyl)-2,3,9-trimethyl-6*H*-thieno[3,2-*f*][1,2,4]triazolo[4,3-*a*][1,4]diazepin-6-yl)-*N*-(2-(2-(4-((((1*S*,5*S*,6*R*)-10-((3,5-dichlorophenyl)sulfonyl)-2-oxo-3-

(pyridin-2-ylmethyl)-3,10-diazabicyclo[4.3.1]decan-5-yl)methoxy)methyl)-1*H*-1,2,3-triazol-1-yl)ethoxy)ethyl)acetamide **a1d**

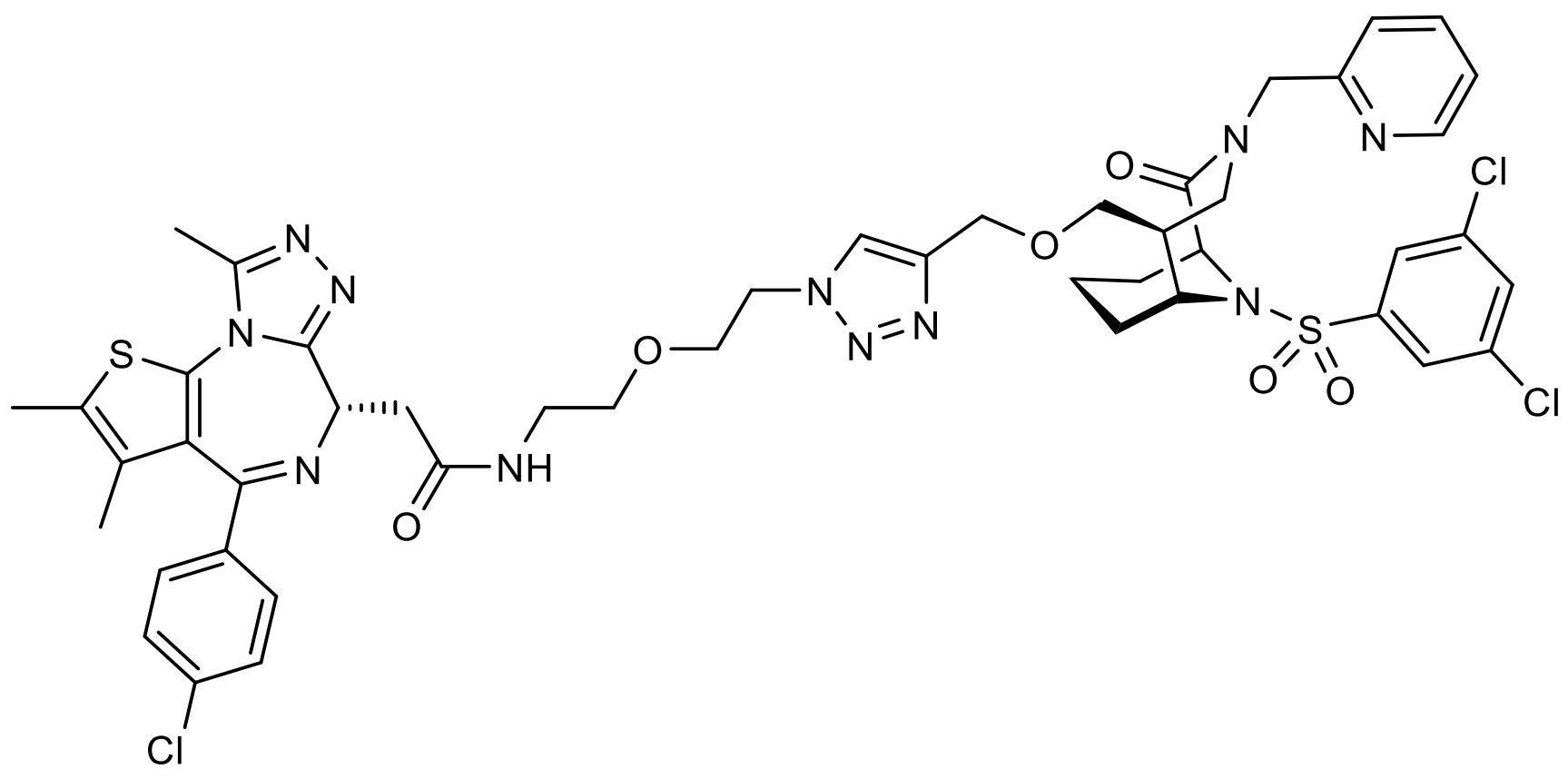

Compound **a1** (5.0 mg, 9.7 µmol, 1.0 eq.) and (*1S,5S,6R*)-10-((3,5-dichlorophenyl)sulfonyl)-5-((prop-2-yn-1-yloxy)methyl)-3-(pyridin-2-ylmethyl)-3,10-diazabicyclo[4.3.1]decan-2-one[20] (5.1 mg, 9.7 µmol, 1.0 eq.) were dissolved in DMSO (1 mL), then ^{t}BuOH (100 µL), H_2O (100 µL), aq. $CuSO_4$ (1 M, 3.9 µL, 3.9 µmol, 0.4 eq.) and aq. sodium ascorbate (1 M, 3.9 µL, 3.9 µmol, 0.4 eq.) were added and the reaction mixture was stirred at room temperature. After 17 h, additional aq. $CuSO_4$ and sodium ascorbate (each 10 µL, 10 µmol, 1.0 eq.) were added and it was stirred for another 4 h. Brine was added and it was extracted with DCM, the organic phase was dried over $MgSO_4$, filtered and concentrated *in vacuo*. The crude product was purified by semi-preparative HPLC (30-70B, 9 CV) to afford the title compound **a1d**.

Yield: 7.8 mg (77 %).
Purity: >99 % (HPLC, UV-absorption 220 nm).
Physical State: yellowish solid.
^{1}H NMR (500 MHz, $CDCl_3$): δ 8.74 (d, *J* = 5.6 Hz, 1H), 8.26 (td, *J* = 7.8, 1.6 Hz, 1H), 7.98 (s, 1H), 7.81 (d, *J* = 8.0 Hz, 1H), 7.75 (t, *J* = 6.7 Hz, 1H), 7.66 (d, *J* = 1.8 Hz, 2H), 7.56 (t, *J* = 1.8 Hz, 1H), 7.39 (d, *J* = 8.1 Hz, 3H), 7.33 (d, *J* = 8.3 Hz, 2H), 5.28 (d, *J* = 16.1 Hz, 1H), 4.91 (d, *J* = 16.1 Hz, 1H), 4.74 (t, *J* = 7.1 Hz, 1H), 4.68 (d, *J* = 6.0 Hz, 1H), 4.63 – 4.51 (m, 4H), 3.88 (td, *J* = 7.6, 4.5 Hz, 3H), 3.83 (dd, *J* = 14.5, 10.7 Hz, 1H), 3.61 – 3.46 (m, 4H), 3.44 – 3.15 (m, 5H), 2.79 (s, 3H), 2.43 (s, 3H), 2.28 – 2.18 (m, 2H), 1.69 (s, 3H), 1.57 – 1.17 (m, 6H).
^{13}C NMR (126 MHz, $CDCl_3$): δ 171.7, 170.7, 164.8, 155.4, 153.7, 150.5, 144.9, 144.0, 143.7, 142.7, 137.6, 136.6, 135.9, 133.1, 132.6, 131.6, 131.5, 130.9, 130.1, 129.0, 125.6, 125.4, 125.0, 124.8, 70.3, 69.7, 69.0, 64.1, 57.0, 53.9, 52.2, 52.1, 50.7, 50.3, 45.1, 39.5, 38.2, 27.6, 27.5, 15.5,

14.5, 13.3, 11.4.

HR-MS (ESI): m/z calculated: $[M+H]^+$ = 1034.25253, found: $[M+H]^+$ = 1034.25307.

2-((*S*)-4-(4-chlorophenyl)-2,3,9-trimethyl-6*H*-thieno[3,2-*f*][1,2,4]triazolo[4,3-*a*][1,4]diazepin-6-yl)-*N*-(2-(2-(4-((1*S*,5*S*,6*R*)-10-((3,5-dichlorophenyl)sulfonyl)-2-oxo-3-(pyridin-2-ylmethyl)-3,10-diazabicyclo[4.3.1]decan-5-yl)-1*H*-1,2,3-triazol-1-yl)ethoxy)ethyl)acetamide **a1c**

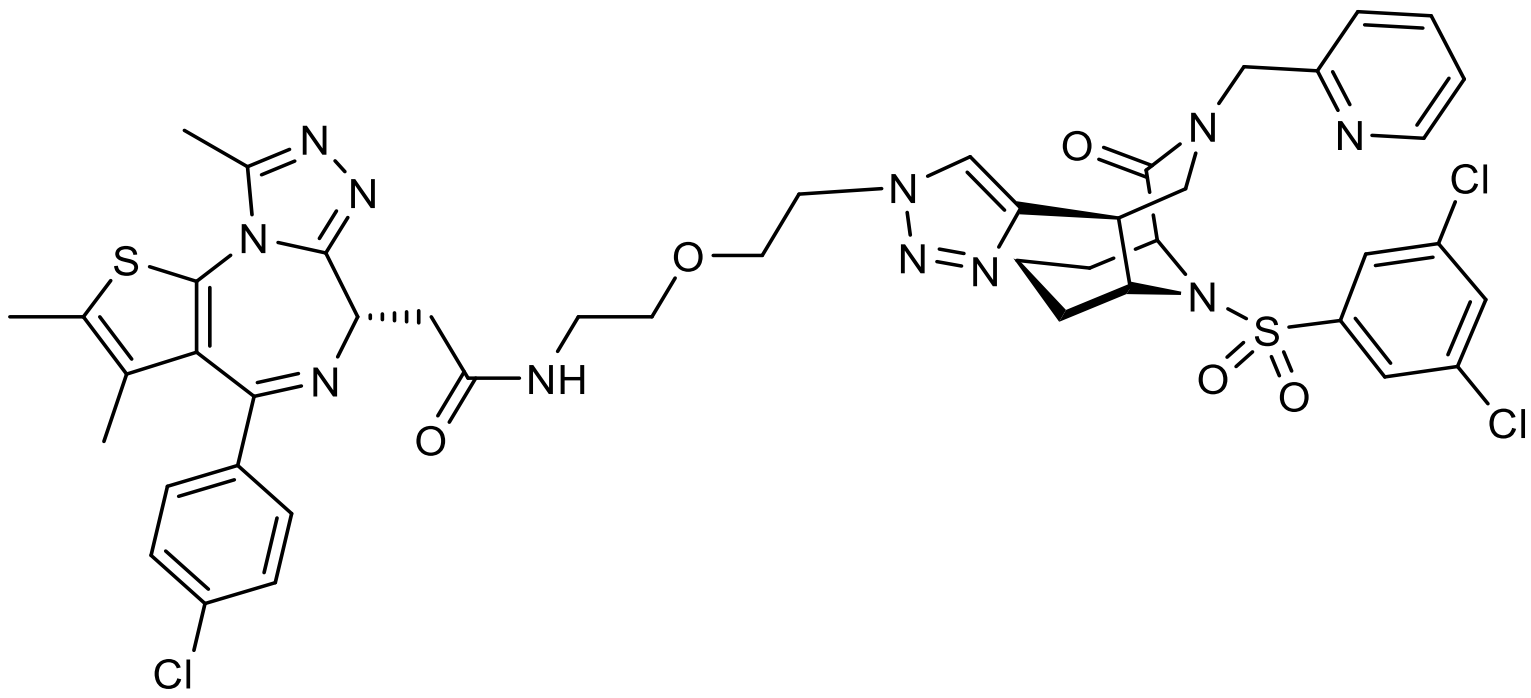

Compound **a1** (5.0 mg, 9.7 µmol, 1.0 eq.) and (*1S,5S,6R*)-10-((3,5-dichlorophenyl)sulfonyl)-5-ethynyl -3-(pyridin-2-ylmethyl)-3,10-diazabicyclo[4.3.1]decan-2-one [20] (4.8 mg, 9.9 µmol, 1.0 eq.) were dissolved in DMSO (1 mL), then ^{t}BuOH (100 µL), H_2O (100 µL), aq. $CuSO_4$ (1 M, 9.7 µL, 9.7 µmol, 1.0 eq.) and aq. sodium ascorbate (1 M, 9.7 µL, 9.7 µmol, 1.0 eq.) were added and the reaction mixture was stirred at room temperature. After 14 h, brine was added and it was extracted with DCM, the organic phase was dried over $MgSO_4$, filtered and concentrated *in vacuo*. The crude product was purified by semi-preparative HPLC (30-70B, 9 CV) to afford the title compound **a1c**.

Yield: 8.2 mg (85 %)

Purity: >99 % (HPLC, UV-absorption 220 nm)

Physical State: yellowish solid.

^{1}H NMR (500 MHz, $CDCl_3$): δ 8.78 (dd, *J* = 5.6, 1.6 Hz, 1H), 8.20 (td, *J* = 7.9, 1.7 Hz, 1H), 7.93 (s, 1H), 7.84 (d, *J* = 8.0 Hz, 1H), 7.74 – 7.60 (m, 3H), 7.43 – 7.28 (m, 6H), 5.40 (d, *J* = 15.9 Hz, 1H), 4.85 (d, *J* = 16.0 Hz, 1H), 4.74 – 4.64 (m, 2H), 4.63 – 4.49 (m, 3H), 4.29 (dd, *J* = 14.2, 10.9 Hz, 1H), 3.84 (t, *J* = 4.9 Hz, 2H), 3.72 – 3.46 (m, 6H), 3.38 (td, *J* = 14.5, 5.4 Hz, 2H), 2.74 (s, 3H), 2.41 (s, 3H), 2.23 (d, J = 13.7 Hz, 1H), 1.84 – 1.72 (m, 1H), 1.67 (s, 3H), 1.59 – 1.49 (m, 2H), 1.41 (dq, *J* = 12.2, 6.3 Hz, 1H), 1.27 (dp, *J* = 11.3, 6.4 Hz, 2H).

^{13}C NMR (126 MHz, $CDCl_3$): δ 171.6, 170.7, 164.3, 155.7, 153.9, 150.3, 147.1, 143.9, 143.6, 143.5, 137.3, 136.4, 136.1, 133.0, 132.1, 131.4, 131.3, 131.2, 130.1, 128.9, 125.8, 125.1, 125.0, 122.6, 69.1, 56.9, 55.7, 54.1, 53.1, 52.9, 50.7, 42.1, 39.6, 38.5, 27.7, 27.5, 15.3, 14.5, 13.3, 11.5.

HR-MS (ESI): m/z calculated: $[M+H]^+$ = 990.22632, found: $[M+H]^+$ = 990.22654.

2-((*S*)-4-(4-chlorophenyl)-2,3,9-trimethyl-6*H*-thieno[3,2-*f*][1,2,4]triazolo[4,3-*a*][1,4]diazepin-6-yl)-*N*-(2-(2-(2-(4-((1*S*,5*S*,6*R*)-10-((3,5-dichlorophenyl)sulfonyl)-2-oxo-3-(pyridin-2-ylmethyl)-3,10-diazabicyclo[4.3.1]decan-5-yl)-1*H*-1,2,3-triazol-1-yl)ethoxy)ethoxy)ethyl)acetamide **a2c**

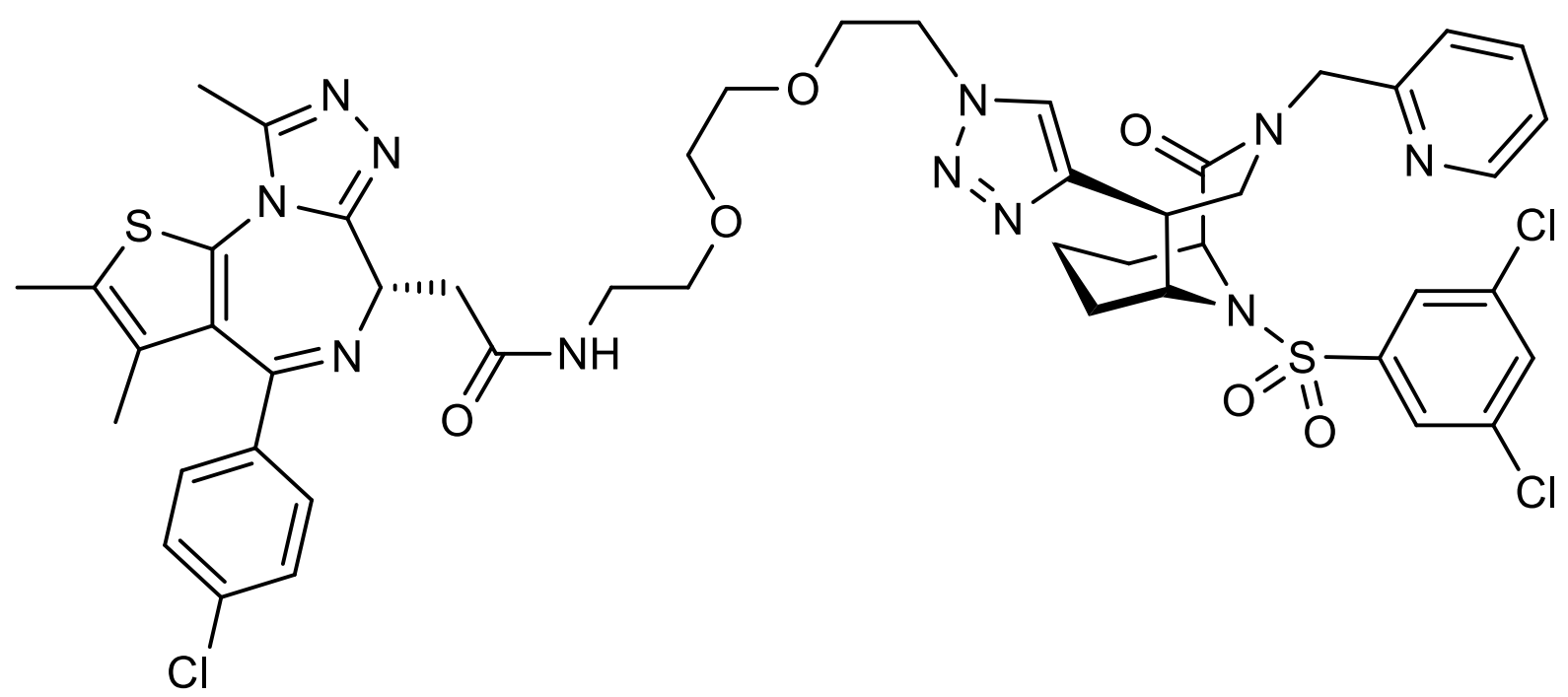

Compound **a2** (5.3 mg, 9.5 µmol, 1.0 eq.) and (*1S,5S,6R*)-10-((3,5-dichlorophenyl)sulfonyl)-5-ethynyl -3-(pyridin-2-ylmethyl)-3,10-diazabicyclo[4.3.1]decan-2-one [20] (4.4 mg, 9.2 µmol, 1.0 eq.) were dissolved in DMSO (1 mL), then ^{t}BuOH (100 µL), H_2O (100 µL), aq. $CuSO_4$ (1 M, 9.0 µL, 9.0 µmol, 1.0 eq.) and aq. sodium ascorbate (1 M, 9.0 µL, 9.0 µmol, 1.0 eq.) were added and the reaction mixture was stirred at room temperature. After 14 h, brine was added and it was extracted with DCM, the organic phase was dried over $MgSO_4$, filtered and concentrated *in vacuo*. The crude product was purified by semi-preparative HPLC (30-70B, 9 CV) to afford the title compound **a2c**.

Yield: 8.1 mg (85 %).

Purity: >99 % (HPLC, UV-absorption 220 nm).

Physical State: yellowish solid.

^{1}H NMR (500 MHz, $CDCl_3$): δ 8.81 (dd, *J* = 5.7, 1.7 Hz, 1H), 8.23 (td, *J* = 7.8, 1.7 Hz, 1H), 7.87-7.67 (m, 6H), 7.56 (t, *J* = 1.8 Hz, 1H), 7.42 (d, *J* = 8.3 Hz, 2H), 7.35 (d, *J* = 8.3 Hz, 2H), 5.44 (d, *J* = 16.2 Hz, 1H), 4.86 (d, *J* = 16.2 Hz, 1H), 4.78 – 4.69 (m, 2H), 4.57 (q, *J* = 5.4 Hz, 3H), 4.34 (dd, *J* = 14.3, 11.0 Hz, 1H), 3.99 – 3.88 (m, 2H), 3.75 – 3.36 (m, 12H), 2.77 (s, 3H), 2.44 (s, 3H), 2.32 (d, *J* = 13.7 Hz, 1H), 1.83 (d, *J* = 13.7 Hz, 1H), 1.70 (s, 3H), 1.66 – 1.57 (m, 2H), 1.51 – 1.38 (m, 1H), 1.37 – 1.25 (m, 1H).

^{13}C NMR (126 MHz, $CDCl_3$): δ 171.4, 170.5, 164.4, 155.3, 154.0, 150.2, 147.0, 143.6, 143.6, 137.2, 136.4, 136.0, 133.0, 132.0, 131.3, 131.1, 131.1, 129.9, 128.8, 124.9, 124.8, 124.8, 122.3, 70.5, 70.2, 69.6, 69.1, 56.8, 55.8, 54.0, 53.0, 52.8, 50.5, 42.1, 39.5, 38.5, 27.5, 27.3, 15.1, 14.3,

13.1, 11.4.

HR-MS (ESI): m/z calculated: $[M+H]^+$ = 1034.25253, found: $[M+H]^+$ = 1034.25306.

2-((*S*)-4-(4-chlorophenyl)-2,3,9-trimethyl-6*H*-thieno[3,2-*f*][1,2,4]triazolo[4,3-*a*][1,4]diazepin-6-yl)-*N*-(2-(2-(4-(((1*S*,5*S*,6*R*)-10-((3,5-dichlorophenyl)sulfonyl)-2-oxo-5-vinyl-3,10-diazabicyclo[4.3.1]decan-3-yl)methyl)-1*H*-1,2,3-triazol-1-yl)ethoxy)ethyl)acetamide **a1i**

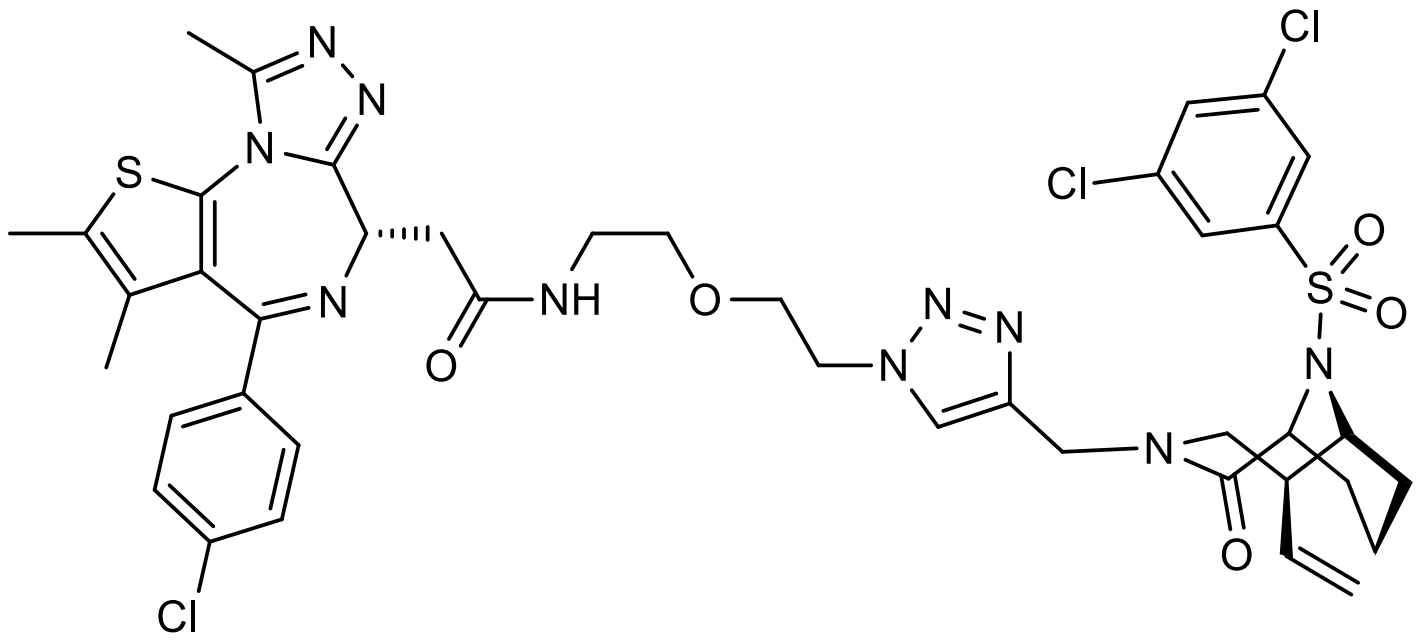

Compound **a1** (5.4 mg, 10.5 µmol, 1.1 eq.) and (1S,5S,6R)-10-((3,5-dichlorophenyl)sulfonyl)-3-(prop-2-yn-1-yl)-5-vinyl-3,10-diazabicyclo[4.3.1]decan-2-one [20] (4.3 mg, 9.9 µmol, 1.0 eq.) were dissolved in DMSO (1 mL), then ^{t}BuOH (100 µL), H_2O (100 µL), aq. $CuSO_4$ (1 M, 9.7 µL, 9.7 µmol, 1.0 eq.) and aq. sodium ascorbate (1 M, 9.7 µL, 9.7 µmol, 1.0 eq.) were added and the reaction mixture was stirred at room temperature. After 14 h, brine was added and it was extracted with DCM, the organic phase was dried over $MgSO_4$, filtered and concentrated *in vacuo*. The crude product was purified by semi-preparative HPLC (30-70B, 9 CV) to afford the title compound **a1i**.

Yield: 7.4 mg (78 %).
Purity: >99 % (HPLC, UV-absorption 220 nm).
Physical State: yellowish solid.
^{1}H NMR (500 MHz, $CDCl_3$): δ 7.94 (s, 1H), 7.65 (d, *J* = 1.9 Hz, 2H), 7.53 (d, *J* = 1.9 Hz, 1H), 7.45 – 7.30 (m, 5H), 5.74 (ddd, *J* = 16.9, 10.2, 8.6 Hz, 1H), 5.15 – 5.05 (m, 2H), 4.83 – 4.65 (m, 4H), 4.64 – 4.51 (m, 2H), 4.03 (dd, *J* = 14.3, 10.9 Hz, 1H), 3.94 (m, 1H), 3.90 – 3.79 (m, 2H), 3.66 (dd, *J* = 15.0, 8.7 Hz, 1H), 3.61 – 3.54 (m, 1H), 3.52 (t, *J* = 4.9 Hz, 2H), 3.41 (dd, *J* = 15.0, 5.8 Hz, 1H), 3.30 (dd, *J* = 14.5, 2.7 Hz, 2H), 2.80 (s, 3H), 2.70 – 2.61 (m, 1H), 2.43 (s, 3H), 2.20 – 2.11 (m, 1H), 1.69 (s, 3H), 1.54 – 1.35 (m, 3H), 1.30 – 1.15 (m, 2H).
^{13}C NMR (126 MHz, $CDCl_3$): δ 171.0, 165.0, 155.4, 150.5, 144.0, 143.4, 136.9, 136.5, 132.9, 132.7, 131.6, 130.2, 129.0, 125.0, 124.8, 117.4, 69.8, 69.1, 56.9, 55.0, 54.0, 52.6, 50.6, 49.1,

46.8, 39.8, 38.2, 27.6, 26.4, 15.4, 14.5, 13.3, 11.4.

HRMS (ESI): m/z calculated: $[M+H]^+$ = 939.21542, found: $[M+H]^+$ = 939.21479.

2-((*S*)-4-(4-chlorophenyl)-2,3,9-trimethyl-6*H*-thieno[3,2-*f*][1,2,4]triazolo[4,3-*a*][1,4]diazepin-6-yl)-*N*-(2-(2-(2-(4-(((1*S*,5*S*,6*R*)-10-((3,5-dichlorophenyl)sulfonyl)-2-oxo-5-vinyl-3,10-diazabicyclo[4.3.1]decan-3-yl)methyl)-1*H*-1,2,3-triazol-1-yl)ethoxy)ethoxy)ethyl)acetamide **a2i**

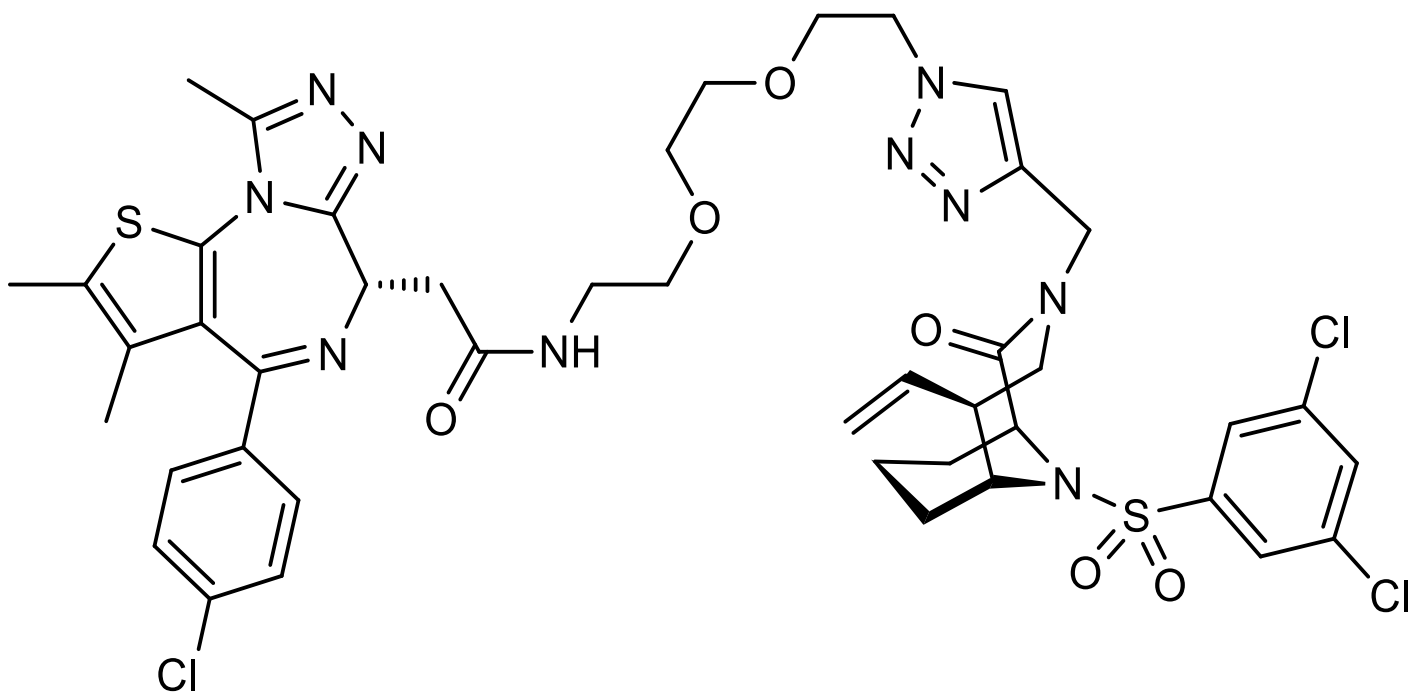

Compound **a2** (5.2 mg, 9.4 µmol, 1.0 eq.) and (*1S,5S,6R*)-10-((3,5-dichlorophenyl)sulfonyl)-3-(prop-2-yn-1-yl)-5-vinyl-3,10-diazabicyclo[4.3.1]decan-2-one [20] (3.8 mg, 9.0 µmol, 1.0 eq.) were dissolved in DMSO (1 mL), then ^{t}BuOH (100 µL), H_2O (100 µL), aq. $CuSO_4$ (1 M, 9.0 µL, 9.0 µmol, 1.0 eq.) and aq. sodium ascorbate (1 M, 9.0 µL, 9.0 µmol, 1.0 eq.) were added and the reaction mixture was stirred at room temperature. After 14 h, brine was added and it was extracted with DCM, the organic phase was dried over $MgSO_4$, filtered and concentrated *in vacuo*. The crude product was purified by semi-preparative HPLC (40-80B, 9 CV) to afford the title compound **a2i**.

Yield: 6.2 mg (70 %)
Purity: 95 % (HPLC, UV-absorption 220 nm)
Physical State: yellowish solid.
^{1}H NMR (500 MHz, $CDCl_3$): δ 7.87 (s, 1H), 7.65 (d, *J* = 1.8 Hz, 2H), 7.57-7.47 (m, 1H), 7.40 (d, *J* = 8.3 Hz, 2H), 7.34 (d, *J* = 8.4 Hz, 3H), 5.73 (dt, *J* = 17.9, 9.1 Hz, 1H), 5.09 (m, 2H), 4.81 (d, *J* = 15.0 Hz, 1H), 4.75 (t, *J* = 7.1 Hz, 1H), 4.71 – 4.64 (m, 2H), 4.63-4.49 (m, 2H), 4.00 (dd, *J* = 14.3, 10.9 Hz, 1H), 3.92 (dt, *J* = 13.5, 5.4 Hz, 3H), 3.69 – 3.41 (m, 10H), 3.30 (dd, *J* = 14.4, 2.1 Hz, 1H), 2.80 (s, 3H), 2.68 – 2.53 (m, 1H), 2.43 (s, 3H), 2.27 – 2.16 (m, 1H), 1.69 (s, 3H), 1.54 – 1.37 (m, 3H), 1.34 – 1.45 (m, 2H).
^{13}C NMR (126 MHz, $CDCl_3$): δ 171.0, 165.0, 155.3, 150.5, 144.0, 143.5, 137.7, 136.9, 136.5,

132.9, 131.6, 130.2, 129.0, 125.0, 124.6, 117.3, 70.6, 70.5, 69.8, 69.2, 56.9, 55.0, 53.9, 52.4, 50.7, 49.1, 46.5, 40.0, 38.2, 27.6, 26.4, 15.4, 14.5, 13.3, 11.4.

HR-MS (ESI): m/z calculated: $[M+H]^+$ = 983.24163, found: $[M+H]^+$ = 953.24137.

N-(2-(2-(4-(2-chloro-4-(((1*S*,5*S*,6*R*)-2-oxo-3-(pyridin-2-ylmethyl)-5-vinyl-3,10-diazabicyclo[4.3.1]decan-10-yl)sulfonyl)phenyl)-1*H*-1,2,3-triazol-1-yl)ethoxy)ethyl)-2-((*S*)-4-(4-chlorophenyl)-2,3,9-trimethyl-6*H*-thieno[3,2-*f*][1,2,4]triazolo[4,3-*a*][1,4]diazepin-6-yl)acetamide **a1h**

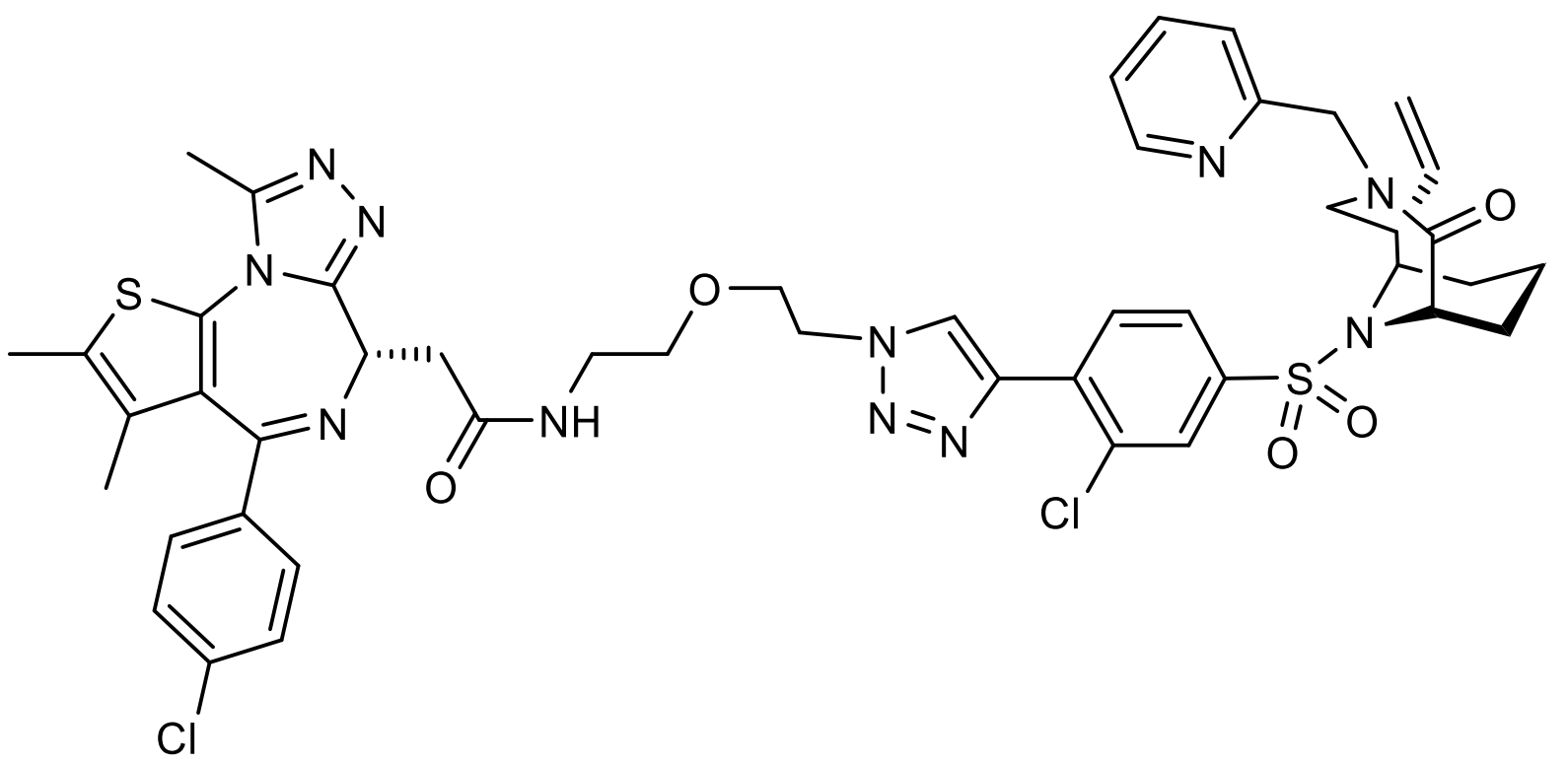

Compound **a1** (5.1 mg, 9.9 µmol, 1.1 eq.) and (*1S,5S,6R*)-10-((3-chloro-4-ethynylphenyl)sulfonyl)-3-(pyridin-2-ylmethyl)-5-vinyl-3,10-diazabicyclo[4.3.1]decan-2-one[20] (4.6 mg, 9.7 µmol, 1.0 eq.) were dissolved in DMSO (1 mL), then *ᵗ*BuOH (100 µL), H_2O (100 µL), aq. $CuSO_4$ (1 M, 9.7 µL, 9.7 µmol, 1.0 eq.) and aq. sodium ascorbate (1 M, 9.7 µL, 9.7 µmol, 1.0 eq.) were added and the reaction mixture was stirred at room temperature. After 14 h, brine was added and it was extracted with DCM, the organic phase was dried over $MgSO_4$, filtered and concentrated *in vacuo*. The crude product was purified by semi-preparative HPLC (30-70B, 9 CV) to afford the title compound **a1h**.

Yield: 3.6 mg (38 %)

Purity: 99 % (HPLC, UV-absorption 220 nm)

Physical State: yellowish solid.

^{1}H NMR (500 MHz, $CDCl_3$): δ 8.80 (dd, *J* = 5.7, 1.7 Hz, 1H), 8.47 (s, 1H), 8.43 (d, *J* = 8.4 Hz, 1H), 8.24 (td, *J* = 7.9, 1.7 Hz, 1H), 7.85 (d, *J* = 1.9 Hz, 1H), 7.77 (d, *J* = 8.1 Hz, 1H), 7.66 (t, *J* = 6.7 Hz, 1H), 7.63 (dd, *J* = 8.3, 2.0 Hz, 1H), 7.40 (d, *J* = 8.3 Hz, 2H), 7.35 (d, *J* = 8.4 Hz, 2H), 6.98 (t, *J* = 5.5 Hz, 1H), 5.75 (dt, *J* = 17.2, 9.4 Hz, 1H), 5.62 (d, *J* = 16.6 Hz, 1H), 5.32 – 4.91 (m, 4H), 4.75 – 4.78 (m, 5H), 4.14 – 4.02 (m, 2H), 3.94 (t, *J* = 5.0 Hz, 2H), 3.64 – 3.49 (m, 4H), 3.42 – 3.29 (m, 2H), 3.12 (dd, *J* = 14.1, 2.1 Hz, 1H), 2.81 – 2.74 (m, 1H), 2.73 (s, 3H), 2.42 (s,

3H), 2.21 – 2.11 (m, 1H), 1.68 (s, 3H), 1.61 – 1.48 (m, 3H), 1.42-1.31 (m, 1H), 1.30 – 1.20 (m, 1H).

^{13}C NMR (126 MHz, $CDCl_3$): δ 172.0, 170.6, 164.6, 155.4, 154.4, 143.6, 142.5, 137.5, 136.5, 136.1, 132.0, 131.4, 131.3, 130.9, 129.0, 128.5, 125.7, 124.8, 124.7, 117.7, 69.9, 69.3, 56.8, 54.8, 54.2, 53.3, 53.2, 50.7, 49.2, 39.5, 38.9, 27.2, 26.4, 15.5, 14.5, 13.3, 11.5.

HR-MS (ESI): m/z calculated: $[M+H]^+$ = 982.28094, found: $[M+H]^+$ = 982.28242.

N-(2-(2-(2-(4-(2-chloro-4-(((1*S*,5*S*,6*R*)-2-oxo-3-(pyridin-2-ylmethyl)-5-vinyl-3,10-diazabicyclo[4.3.1]decan-10-yl)sulfonyl)phenyl)-1*H*-1,2,3-triazol-1-yl)ethoxy)ethoxy)ethyl)-2-((*S*)-4-(4-chlorophenyl)-2,3,9-trimethyl-6*H*-thieno[3,2-*f*][1,2,4]triazolo[4,3-*a*][1,4]diazepin-6-yl)acetamide **a2h**

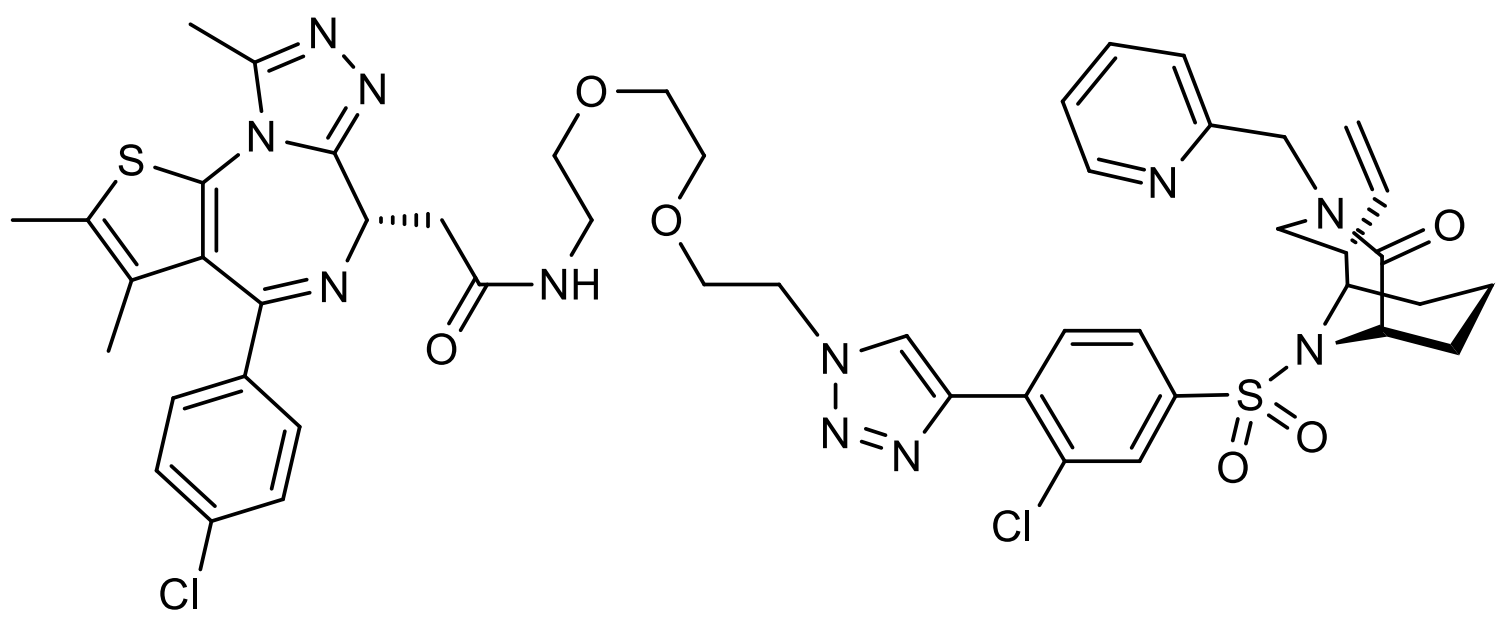

Compound **a2** (5.1 mg, 9.2 μmol, 1.0 eq.) and (*1S,5S,6R*)-10-((3-chloro-4-ethynylphenyl)sulfonyl)-3-(pyridin-2-ylmethyl)-5-vinyl-3,10-diazabicyclo[4.3.1]decan-2-one[20] (4.2 mg, 9.0 μmol, 1.0 eq.) were dissolved in DMSO (1 mL), then ^{t}BuOH (100 μL), H_2O (100 μL), aq. $CuSO_4$ (1 M, 9.0 μL, 9.0 μmol, 1.0 eq.) and aq. sodium ascorbate (1 M, 9.0 μL, 9.0 μmol, 1.0 eq.) were added and the reaction mixture was stirred at room temperature. After 14 h, brine was added and it was extracted with DCM, the organic phase was dried over $MgSO_4$, filtered and concentrated *in vacuo*. The crude product was purified by semi-preparative HPLC (30-70B, 9 CV) to afford the title compound **a2h**.

Yield: 3.8 mg (41 %)

Purity: >99 % (HPLC, UV-absorption 220 nm)

Physical State: yellowish solid

HR-MS (ESI): m/z calculated: $[M+H]^+$ = 1026.30715, found: $[M+H]^+$ = 1026.30723

N-(2-(2-(4-(3-chloro-5-(((1*S*,5*S*,6*R*)-2-oxo-3-(pyridin-2-ylmethyl)-5-vinyl-3,10-

diazabicyclo[4.3.1]decan-10-yl)sulfonyl)phenyl)-1*H*-1,2,3-triazol-1-yl)ethoxy)ethyl)-2-((*S*)-4-(4-chlorophenyl)-2,3,9-trimethyl-6*H*-thieno[3,2-*f*][1,2,4]triazolo[4,3-*a*][1,4]diazepin-6-yl)acetamide **a1g**

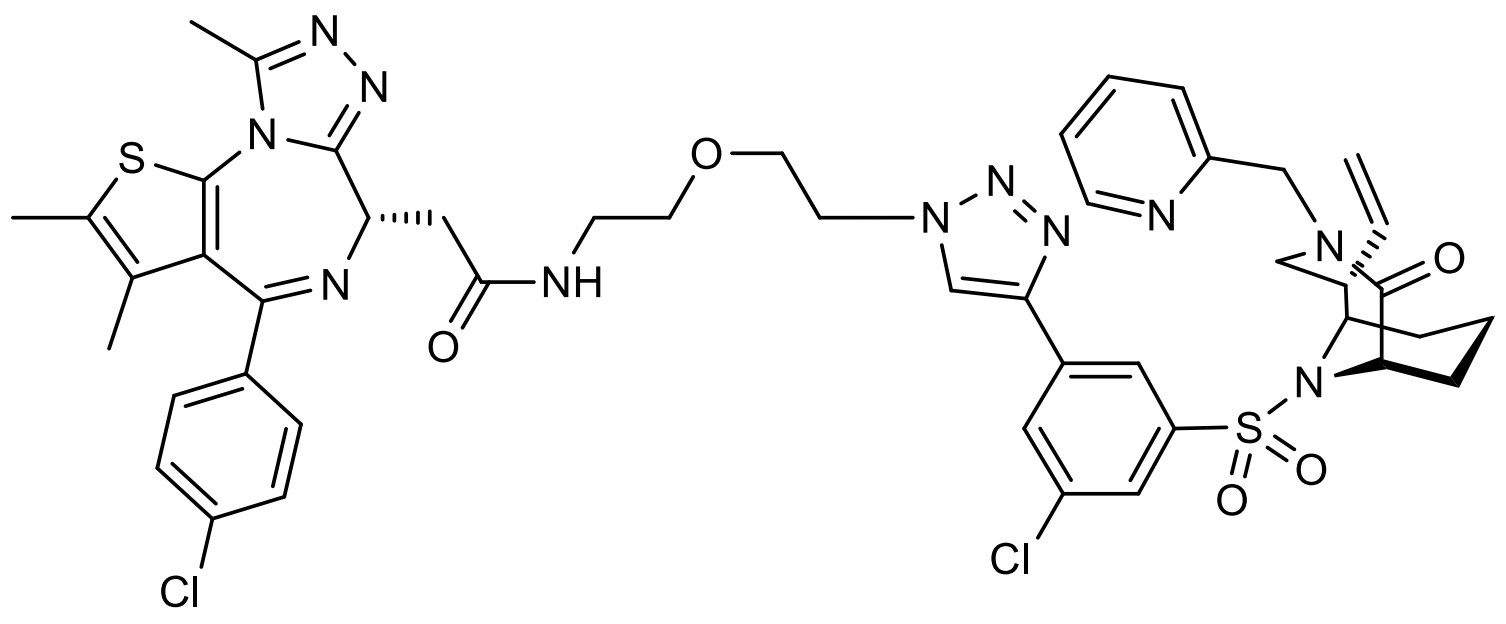

Compound **a1** (5.1 mg, 9.9 µmol, 1.1 eq.) and (*1S,5S,6R*)-10-((3-chloro-5-ethynylphenyl)sulfonyl)-3-(pyridin-2-ylmethyl)-5-vinyl-3,10-diazabicyclo[4.3.1]decan-2-one[20] (4.6 mg, 9.7 µmol, 1.0 eq.) were dissolved in DMSO (1 mL), then *t*BuOH (100 µL), H_2O (100 µL), aq. $CuSO_4$ (1 M, 9.7 µL, 9.7 µmol, 1.0 eq.) and aq. sodium ascorbate (1 M, 9.7 µL, 9.7 µmol, 1.0 eq.) were added and the reaction mixture was stirred at room temperature. After 14 h, brine was added and it was extracted with DCM, the organic phase was dried over $MgSO_4$, filtered and concentrated *in vacuo*. The crude product was purified by semi-preparative HPLC (35-75B, 9 CV) to afford the title compound **a1g**.

Yield: 7.3 mg (76 %)
Purity: 99 % (HPLC, UV-absorption 220 nm)
Physical State: yellowish solid.
^{1}H NMR (500 MHz, $CDCl_3$): δ 8.87 – 8.67 (m, 1H), 8.42 (s, 1H), 8.31 (td, *J* = 7.9, 1.6 Hz, 1H), 8.14 (t, *J* = 1.6 Hz, 1H), 8.04 (d, *J* = 1.6 Hz, 1H), 7.78 (d, *J* = 8.1 Hz, 1H), 7.71 (t, *J* = 6.7 Hz, 1H), 7.35 (t, *J* = 7.9 Hz, 4H), 7.29 – 7.15 (m, 1H), 6.74 (t, *J* = 5.7 Hz, 1H), 5.69 (ddd, *J* = 16.8, 10.3, 8.6 Hz, 1H), 5.54 (d, *J* = 16.5 Hz, 1H), 5.24 – 5.03 (m, 2H), 4.79 – 4.51 (m, 5H), 4.01 (dd, *J* = 14.1, 10.9 Hz, 1H), 3.92 (t, *J* = 5.3 Hz, 3H), 3.79 – 3.46 (m, 3H), 3.29 (dd, *J* = 16.3, 8.1 Hz, 2H), 3.20 (dd, *J* = 14.7, 5.2 Hz, 1H), 3.10 (dd, *J* = 14.1, 2.0 Hz, 1H), 2.72 (s, 4H), 2.39 (s, 3H), 2.14 (d, *J* = 13.5 Hz, 1H), 1.64 (s, 3H), 1.53 (d, *J* = 13.9 Hz, 3H), 1.38 – 1.20 (m, 2H).
^{13}C NMR (126 MHz, $CDCl_3$): δ 172.2, 170.5, 164.9, 155.1, 154.1, 150.6, 144.8, 144.6, 143.0, 142.6, 137.6, 136.4, 135.9, 135.7, 134.1, 132.7, 131.6, 131.5, 130.6, 130.3, 129.8, 129.0, 125.2, 125.1, 125.0, 123.2, 121.9, 117.7, 69.8, 69.0, 56.6, 54.7, 53.9, 53.3, 53.0, 50.7, 49.1, 39.3, 38.6, 27.2, 26.4, 15.5, 14.5, 13.3, 11.3.

HR-MS (ESI): m/z calculated: $[M+H]^+$ = 982.28094, found: $[M+H]^+$ = 982.28149

N-(2-(2-(2-(4-(3-chloro-5-(((1*S*,5*S*,6*R*)-2-oxo-3-(pyridin-2-ylmethyl)-5-vinyl-3,10-diazabicyclo[4.3.1]decan-10-yl)sulfonyl)phenyl)-1*H*-1,2,3-triazol-1-yl)ethoxy)ethoxy)ethyl)-2-((*S*)-4-(4-chlorophenyl)-2,3,9-trimethyl-6*H*-thieno[3,2-*f*][1,2,4]triazolo[4,3-*a*][1,4]diazepin-6-yl)acetamide **a2g**

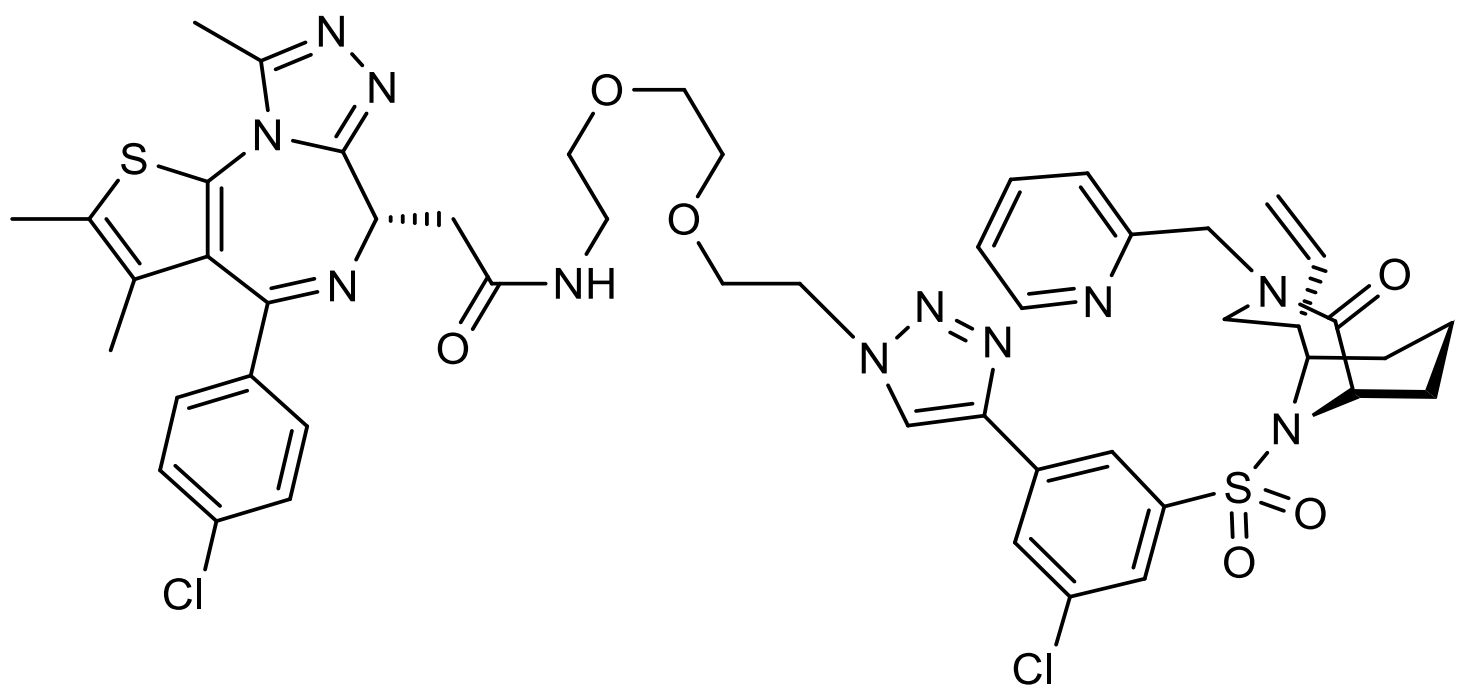

Compound **a2** (5.0 mg, 9.0 μmol, 1.0 eq.) and (*1S,5S,6R*)-10-((3-chloro-5-ethynylphenyl)sulfonyl)-3-(pyridin-2-ylmethyl)-5-vinyl-3,10-diazabicyclo[4.3.1]decan-2-one[20] (4.2 mg, 9.0 μmol, 1.0 eq.) were dissolved in DMSO (1 mL), then ^{t}BuOH (100 μL), H_2O (100 μL), aq. $CuSO_4$ (1 M, 9.0 μL, 9.0 μmol, 1.0 eq.) and aq. sodium ascorbate (1 M, 9.0 μL, 9.0 μmol, 1.0 eq.) were added and the reaction mixture was stirred at room temperature. After 14 h, brine was added and it was extracted with DCM, the organic phase was dried over $MgSO_4$, filtered and concentrated *in vacuo*. The crude product was purified by semi-preparative HPLC (33-73B, 9 CV) to afford the title compound **a2g**.

Yield: 6.1 mg (66 %)

Purity: >99 % (HPLC, UV-absorption 220 nm)

Physical State: yellowish solid.

^{1}H NMR (500 MHz, $CDCl_3$): δ 8.79 (d, *J* = 5.6 Hz, 1H), 8.44 – 8.20 (m, 2H), 8.11 (dt, *J* = 8.8, 1.6 Hz, 2H), 7.77 (d, *J* = 8.1 Hz, 1H), 7.67 (t, *J* = 6.7 Hz, 1H), 7.64 (s, 1H), 7.39 (d, *J* = 8.3 Hz, 2H), 7.32 (d, *J* = 8.2 Hz, 2H), 7.16 (t, *J* = 5.5 Hz, 1H), 5.73 (dt, *J* = 17.2, 9.3 Hz, 2H), 5.56 (d, *J* = 16.6 Hz, 1H), 5.20 – 5.08 (m, 2H), 4.77 – 4.61 (m, 5H), 4.14 – 4.04 (m, 2H), 3.98 (t, *J* = 5.0 Hz, 2H), 3.73 – 3.29 (m, 10H), 3.11 (dd, *J* = 14.3, 2.0 Hz, 1H), 2.84 – 2.74 (m, 1H), 2.73 (s, 3H), 2.41 (s, 3H), 2.23 – 2.13 (m, 1H), 1.67 (s, 3H), 1.55 (q, *J* = 11.2 Hz, 3H), 1.47 – 1.34 (m, 1H), 1.29 (td, *J* = 14.7, 7.7 Hz, 1H).

HR-MS (ESI): m/z calculated: $[M+H]^+$ = 1026.30715, found: $[M+H]^+$ = 1026.30718.

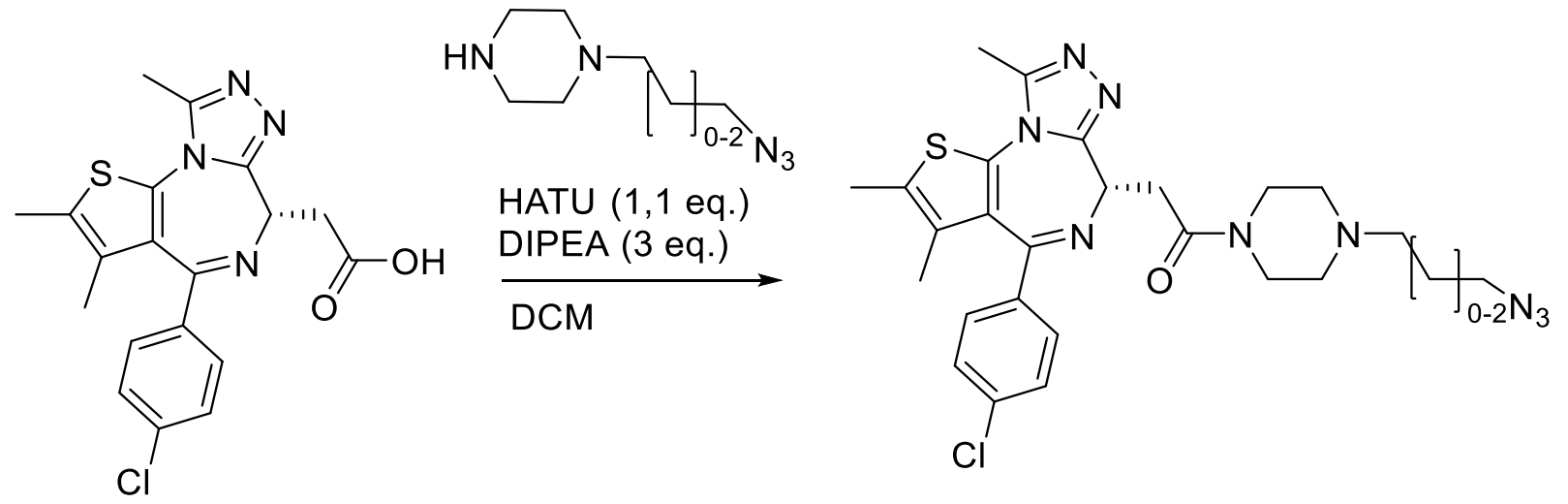

(*S*)-1-(4-(2-azidoethyl)piperazin-1-yl)-2-(4-(4-chlorophenyl)-2,3,9-trimethyl-6H-thieno[3,2-f][1,2,4]triazolo[4,3-a][1,4]diazepin-6-yl)ethan-1-on **a4**

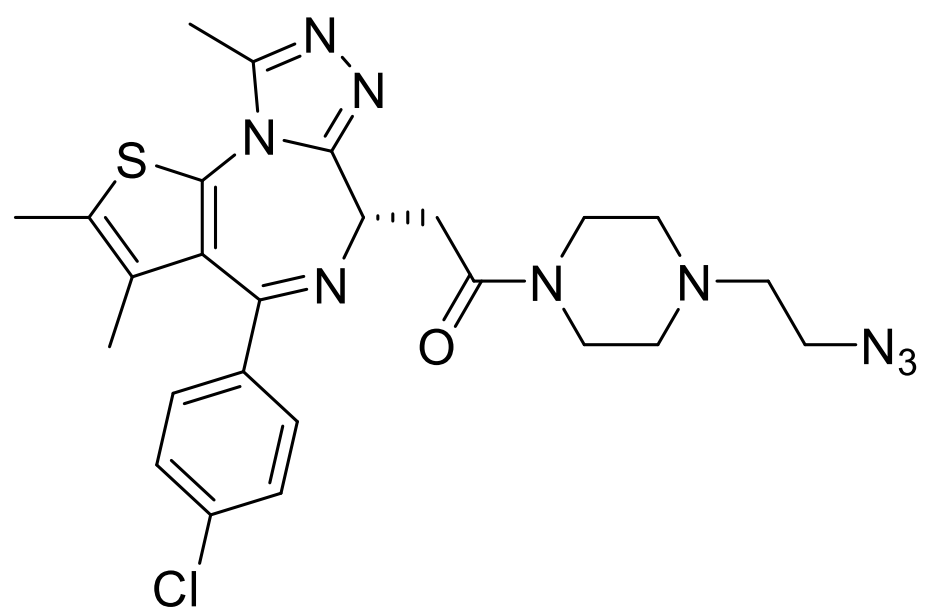

According to **GP-1**, the title compound **a4** was synthesized from JQ1 acid (30 mg, 75 µmol, 1.00 eq.) and 1-(2-azidoethyl)piperazine (19 mg, 100 µmol, 1.00 eq.). The obtained product was further purified by prep. HPLC (5-100 % B).

Yield: 19 mg (36 µmol, 48%).

Physical State: yellow solid.

^{1}H NMR (500 MHz, $CDCl_3$) δ 7.48 – 7.31 (m, 4H), 4.81 (dd, *J* = 9.7, 5.2 Hz, 1H), 4.53 - 4.43 (m, 1H), 4.36 (d, *J* = 14.9 Hz, 1H), 4.04 – 3.80 (m, 4H), 3.66 – 3.18 (m, 8H), 2.72 (s, 3H), 2.43 (s, 3H), 1.70 (s, 3H).

^{13}C NMR (126 MHz, $CDCl_3$) 168.9, 164.2, 155.4, 150.1, 137.0, 136.5, 132.0, 131.8, 131.1, 130.8, 129.8, 128.9, 55.5, 54.7, 52.1, 51.7, 45.9, 43.4, 39.4, 34.5, 14.5, 13.2, 11.5 ppm.

HRMS (ESI, m/z) calcd. for $C_{25}H_{29}ClN_9OS$ [M+H$^+$]:538.18988, found: 538.19029.

(*1S,5S,6R)*-5-(((1-(2-(4-(2-((S)-4-(4-chlorophenyl)-2,3,9-trimethyl-6H-thieno[3,2-f][1,2,4]triazolo[4,3-a][1,4]diazepin-6-yl)acetyl)piperazin-1-yl)ethyl)-1H-1,2,3-triazol-4-yl)oxy)methyl)-10-((3,5-dichlorophenyl)sulfonyl)-3-(pyridin-2-ylmethyl)-3,10-diazabicyclo[4.3.1]decan-2-one **a4d**

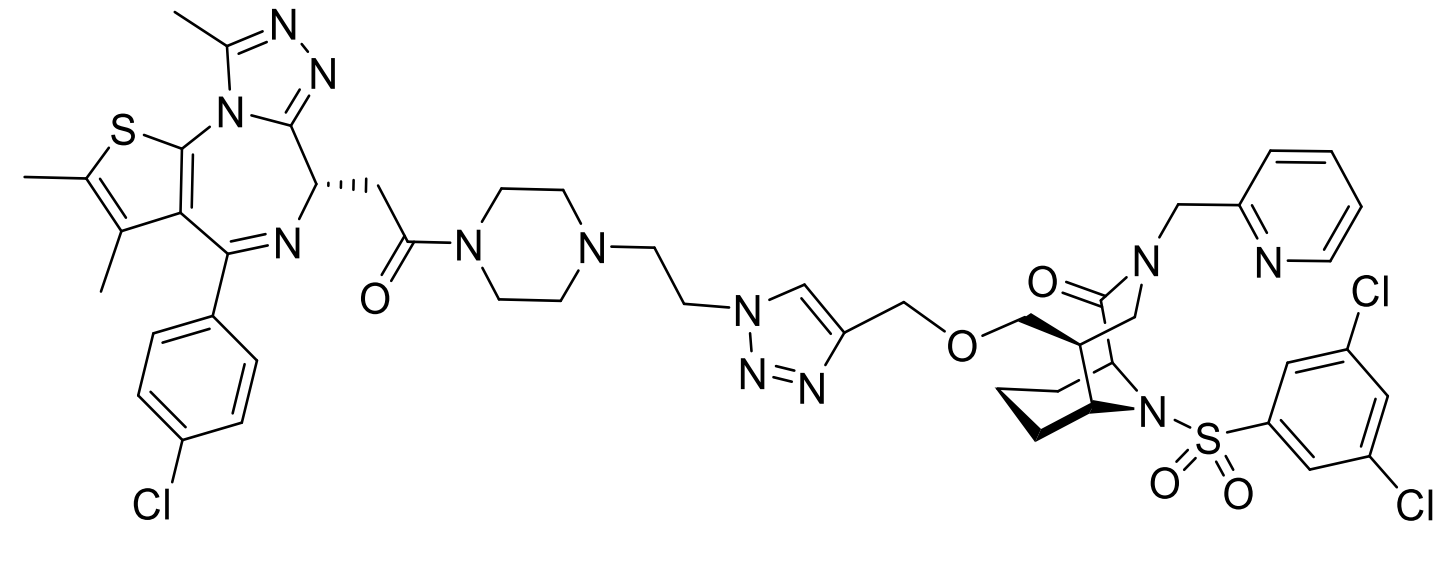

According to **GP-2**, the title compound **a4d** was synthesized from **a4** (5.4 mg, 10 µmol, 1.00 eq.) and (*1S,5S,6R*)-10-((3,5-dichlorophenyl)sulfonyl)-5-((prop-2-yn-1-yloxy)methyl)-3-(pyridin-2-ylmethyl)-3,10-diazabicyclo[4.3.1]decan-2-one[20] (5.2 mg, 10 µmol, 1.00 eq.). The obtained product was further purified by prep. HPLC (5-100 % B).

Yield: 5.5 mg (4 µmol, 50%).

Purity: 91 % (HPLC, UV-absorption 220 nm)

Physical State: yellow solid.

HRMS (ESI, m/z) calcd. for $C_{49}H_{54}Cl_3N_{12}O_5S_2$ [M+H$^+$]:1059.28416, found: 1059.28544.

(*S*)-1-(4-(3-azidopropyl)piperazin-1-yl)-2-(4-(4-chlorophenyl)-2,3,9-trimethyl-6H-thieno[3,2-f][1,2,4]triazolo[4,3-a][1,4]diazepin-6-yl)ethan-1-o **a5**

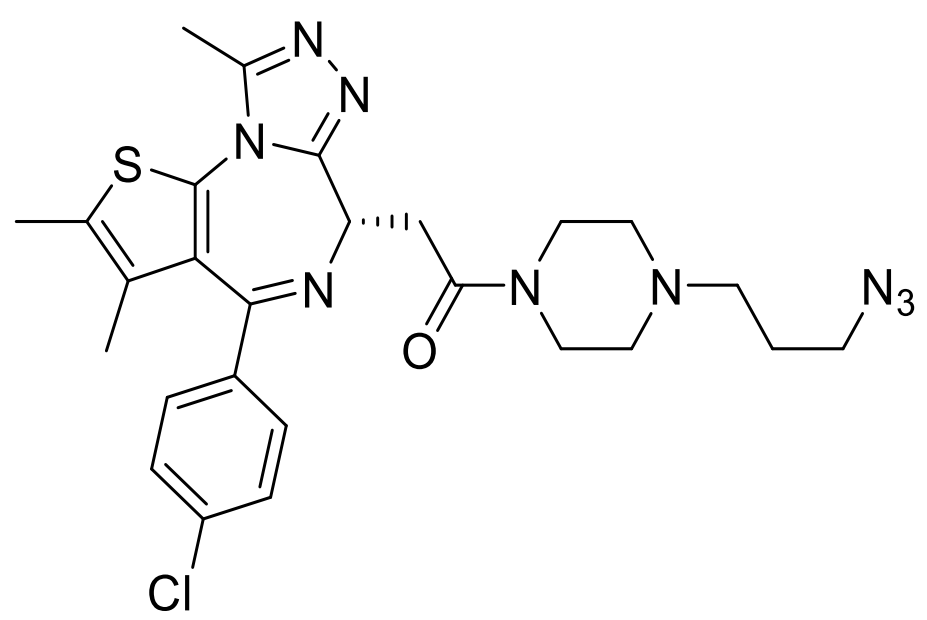

According to **GP-1**, the title compound **a5** was synthesized from JQ1 acid (40 mg, 100 µmol, 1.00 eq.) and 1-(3-azidopropyl)piperazine (19 mg, 100 µmol, 1.00 eq.). The obtained product was further purified by prep. HPLC (5-100 % B).

Yield: 6 mg (10 µmol, 10%).

Physical State: yellow solid.

^{1}H NMR (500 MHz, $CDCl_3$) δ 7.48 – 7.31 (m, 4H), 4.81 (dd, *J* = 9.7, 5.2 Hz, 1H), 4.53 - 4.43 (m, 1H), 4.55 – 4.41 (m, 1H), 4.04 – 3.80 (m, 4H), 3.66 – 3.18 (m, 10H), 2.72 (s, 3H), 2.43 (s, 3H), 1.70 (s, 3H).

^{13}C NMR (126 MHz, $CDCl_3$) 168.9, 164.2, 155.4, 150.1, 137.0, 136.5, 132.0, 131.8, 131.1, 130.8, 129.8, 128.9, 55.5, 54.7, 51.4, 51.6, 48.4, 43.4, 38.9, 34.5, 23.4, 14.5, 13.2, 11.5 ppm.

HRMS (ESI, m/z) calcd. for $C_{26}H_{31}ClN_9OS$ [M+Na$^+$]:574.18748, found: 574.18772.

(*1S,5S,6R*)-5-(((1-(3-(4-(2-((*S*)-4-(4-chlorophenyl)-2,3,9-trimethyl-6H-thieno[3,2-f][1,2,4]triazolo[4,3-a][1,4]diazepin-6-yl)acetyl)piperazin-1-yl)propyl)-1H-1,2,3-triazol-4-yl)oxy)methyl)-10-((3,5-dichlorophenyl)sulfonyl)-3-(pyridin-2-ylmethyl)-3,10-diazabicyclo[4.3.1]decan-2-one **a5d**

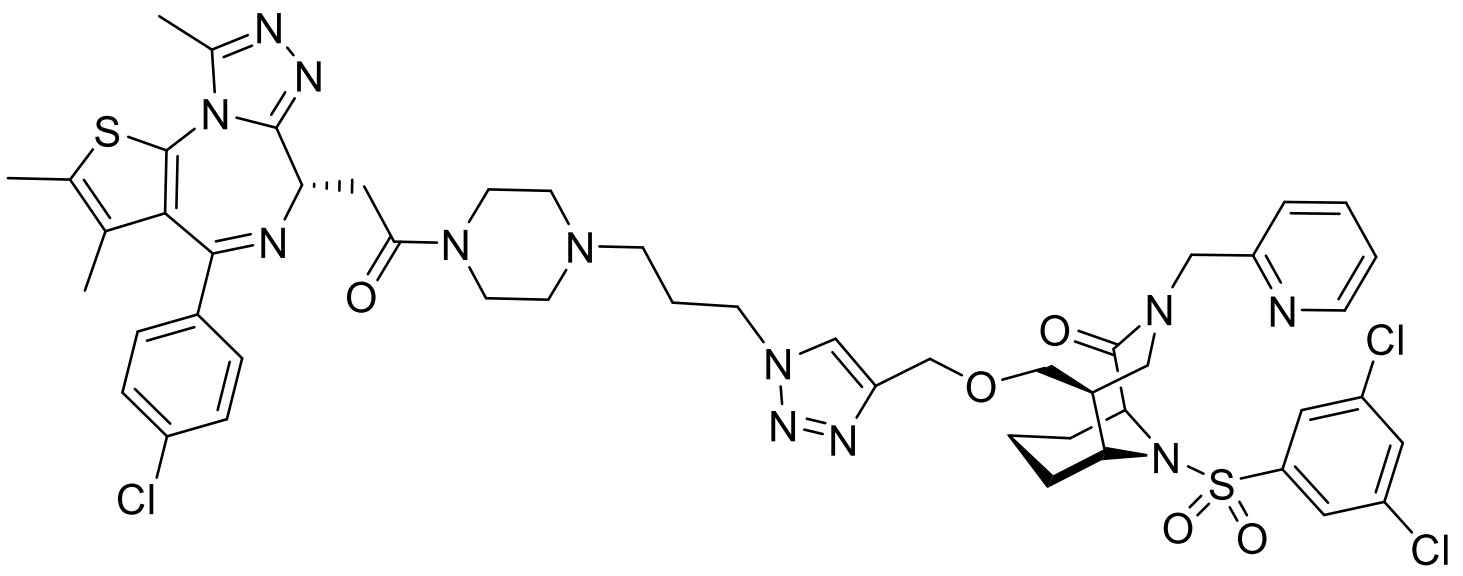

According to **GP-2**, the title compound **a5d** was synthesized from **a5** (5.4 mg, 10 µmol, 1.00 eq.) and (*1S,5S,6R*)-10-((3,5-dichlorophenyl)sulfonyl)-5-((prop-2-yn-1-yloxy)methyl)-3-(pyridin-2-ylmethyl)-3,10-diazabicyclo[4.3.1]decan-2-one[20] (5.2 mg, 10 µmol, 1.00 eq.). The obtained product was further purified by prep. HPLC (5-100 % B).

Yield: 5.7 mg (5.3 µmol, 53%).

Purity: 88 % (HPLC, UV-absorption 220 nm)

Physical State: yellow solid.

HRMS (ESI, m/z) $C_{50}H_{56}Cl_3N_{12}O_5S_2$ [M+H$^+$]:1073.29981, found: 1073.30181.

(*S*)-1-(4-(4-azidobutyl)piperazin-1-yl)-2-(4-(4-chlorophenyl)-2,3,9-trimethyl-6H-thieno[3,2-f][1,2,4]triazolo[4,3-a][1,4]diazepin-6-yl)ethan-1-one **a6**

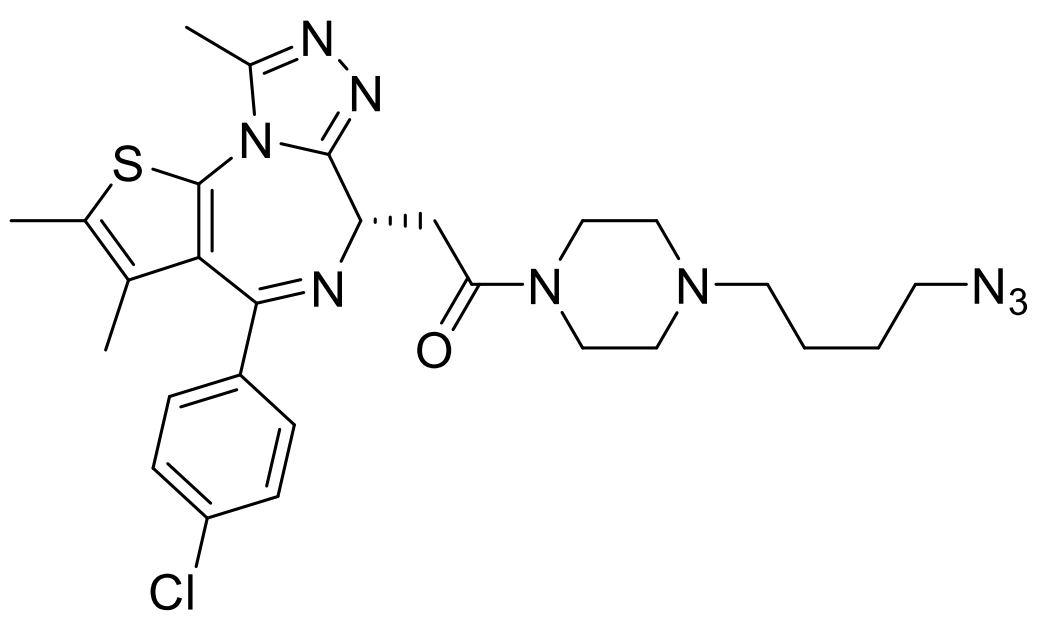

According to **GP-1**, the title compound **a6** was synthesized from JQ1 acid (20 mg, 50 µmol, 1.00 eq.) and 1-(4-azidobutyl)piperazine(18 mg, 100 µmol, 2.00 eq.). The obtained product was further purified by prep. HPLC (5-100 % B).

Yield: 12 mg (20 µmol, 43%).

Physical State: yellow solid.

^{1}H NMR (500 MHz, $CDCl_3$) δ 7.48 – 7.31 (m, 4H), 4.81 (dd, *J* = 9.7, 5.2 Hz, 1H), 4.53 - 4.43 (m, 1H), 4.55 – 4.41 (m, 1H), 4.04 – 3.80 (m, 4H), 3.66 – 3.18 (m, 12H), 2.72 (s, 3H), 2.43 (s, 3H), 1.70 (s, 3H).

^{13}C NMR (126 MHz, $CDCl_3$) 168.9, 164.2, 155.4, 150.1, 137.0, 136.5, 132.0, 131.8, 131.1, 130.8, 129.8, 128.9, 56.7, 51.5, 51.2, 50.5, 48.4, 43.4, 38.9, 34.5, 25.9, 21.1, 14.5, 13.2, 11.5 ppm.

HRMS (ESI, m/z) calcd. for $C_{27}H_{33}ClN_9O_{10}$ [M+H$^+$]:566.22118, found: 566.22120.

(*1S,5S,6R*)-5-(((1-(4-(4-(2-((*S*)-4-(4-chlorophenyl)-2,3,9-trimethyl-6H-thieno[3,2-f][1,2,4]triazolo[4,3-a][1,4]diazepin-6-yl)acetyl)piperazin-1-yl)butyl)-1H-1,2,3-triazol-4-yl)oxy)methyl)-10-((3,5-dichlorophenyl)sulfonyl)-3-(pyridin-2-ylmethyl)-3,10-diazabicyclo[4.3.1]decan-2-one **a6d**

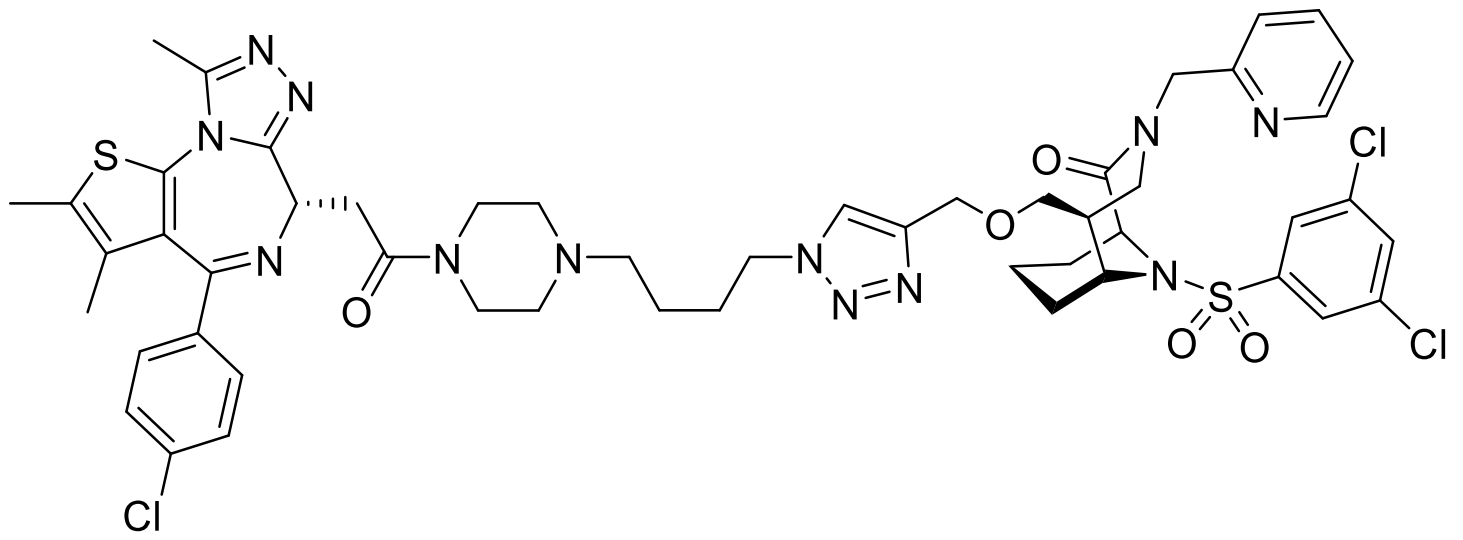

According to **GP-2**, the title compound **a6d** was synthesized from **a6** (5.7 mg, 10 µmol, 1.00 eq.) and (*1S,5S,6R*)-10-((3,5-dichlorophenyl)sulfonyl)-5-((prop-2-yn-1-yloxy)methyl)-3-(pyridin-2-ylmethyl)-3,10-diazabicyclo[4.3.1]decan-2-one[20] (5.2 mg, 10 µmol, 1.00 eq.). The obtained product was further purified by prep. HPLC (5-100 % B).

Yield: 9 mg (8.3 µmol, 83%).

Purity: 99 % (HPLC, UV-absorption 220 nm)

Physical State: yellow solid.

HRMS (ESI, m/z) calcd. for $C_{51}H_{58}Cl_3N_{12}O_{10}S_2$ [M+H$^+$]:1087.31456, found: 1087.31535.

2-((*S*)-4-(4-chlorophenyl)-2,3,9-trimethyl-6H-thieno[3,2-f][1,2,4]triazolo[4,3-a][1,4]diazepin-6-yl)-N-(2-(2-(4-(((1S,5S,6R)-10-((3,5-dichlorophenyl)sulfonyl)-2-oxo-3-((S)-1-(pyridin-2-yl)ethyl)-3,10-diazabicyclo[4.3.1]decan-5-yl)methoxy)-1H-1,2,3-triazol-1-yl)ethoxy)ethyl)acetamide **a1dj**

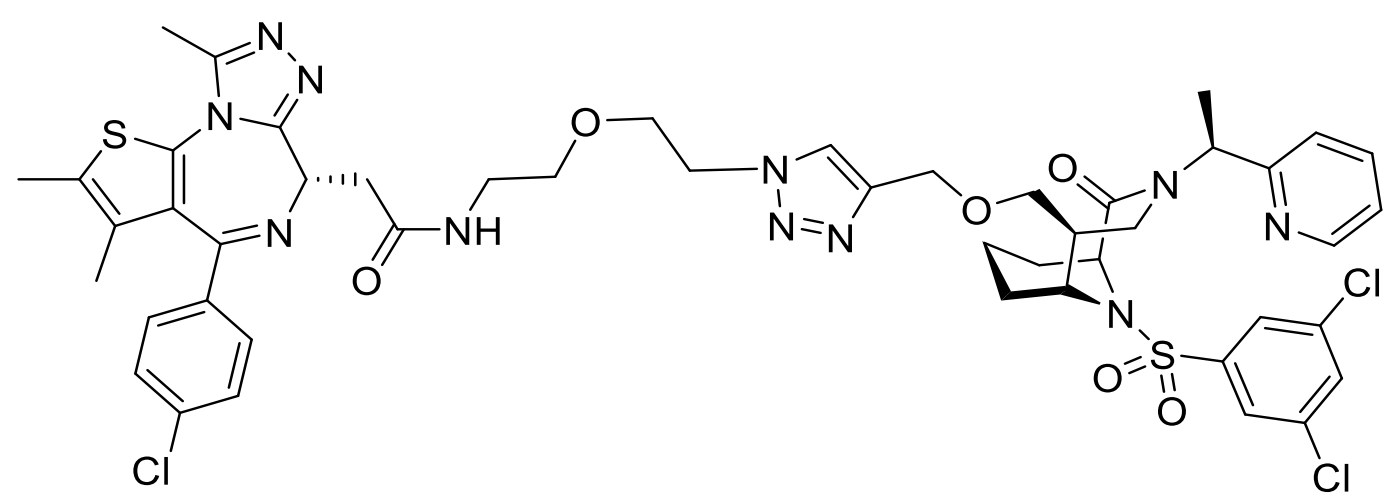

According to **GP-2**, the title compound **a1dj** was synthesized from compound **a1** (5.1 mg, 10 µmol, 1.00 eq.) and (*1S,5S,6R*)-10-((3,5-dichlorophenyl)sulfonyl)-5-((prop-2-yn-1-yloxy)methyl)-3-((*S*)-1-(pyridin-2-yl)ethyl)-3,10-diazabicyclo[4.3.1]decan-2-one [21] (5.2 mg, 10 µmol, 1.00 eq.). The obtained product was further purified by prep. HPLC (5-100 % B).

Yield: 6 mg (6 µmol, 60%).

Purity: 89 % (HPLC, UV-absorption 220 nm)

Physical State: yellow solid.

HRMS (ESI, m/z) calcd. for $C_{48}H_{53}Cl_3N_{11}O_6S_2$ [M+H$^+$]:1048.26818, found: 1048.26826.

^{1}H NMR (500 MHz, DMSO) δ 8.57 (d, *J* = 4.9 Hz, 1H), 8.24 (t, *J* = 5.7 Hz, 1H), 8.06 (s, 1H), 7.96 (dd, *J* = 4.9, 1.8 Hz, 3H), 7.88 – 7.80 (m, 1H), 7.47 (d, *J* = 8.4 Hz, 2H), 7.42 – 7.38 (m, 2H), 7.37 – 7.32 (m, 1H), 7.18 (dd, *J* = 8.4, 2.7 Hz, 1H), 5.90 (q, *J* = 6.9 Hz, 1H), 4.80 (d, *J* = 5.5 Hz, 1H), 4.56 – 4.45 (m, 5H), 3.44 (t, *J* = 5.8 Hz, 3H), 3.35 (dd, *J* = 10.0, 4.9 Hz, 2H), 3.25 (ddd, *J* = 15.1, 8.8, 5.7 Hz, 5H), 2.96 (qd, *J* = 14.6, 6.0 Hz, 2H), 2.59 (s, 3H), 2.40 (s, 3H), 2.20 – 2.04 (m, 2H), 1.60 (s, 3H), 1.55 – 1.38 (m, 6H), 1.31 – 1.13 (m, 3H).

^{13}C NMR (126 MHz, DMSO) δ 169.6, 169.3, 163.1, 158.7, 158.3, 158.0, 157.7, 155.0, 149.9, 148.4, 143.6, 143.5, 136.6, 135.4, 135.2, 132.6, 132.2, 130.8, 130.1, 129.8, 129.5, 128.4, 125.1, 124.2, 122.7, 121.8, 69.8, 68.9, 68.5, 63.5, 56.4, 54.3, 53.7, 51.7, 49.3, 44.6, 43.2, 38.4, 37.4, 27.9, 27.2, 15.1, 15.0, 14.0, 12.6, 11.3.

2-((*S*)-4-(4-chlorophenyl)-2,3,9-trimethyl-6H-thieno[3,2-f][1,2,4]triazolo[4,3-a][1,4]diazepin-6-yl)-N-(2-(2-(4-(((*1S,5S,6R*)-10-((3,5-dichlorophenyl)sulfonyl)-3-(2-(1-methylpiperidin-4-yl)-2-oxoethyl)-2-oxo-3,10-diazabicyclo[4.3.1]decan-5-yl)methoxy)-1H-1,2,3-triazol-1-yl)ethoxy)ethyl)acetamide **a1dk**

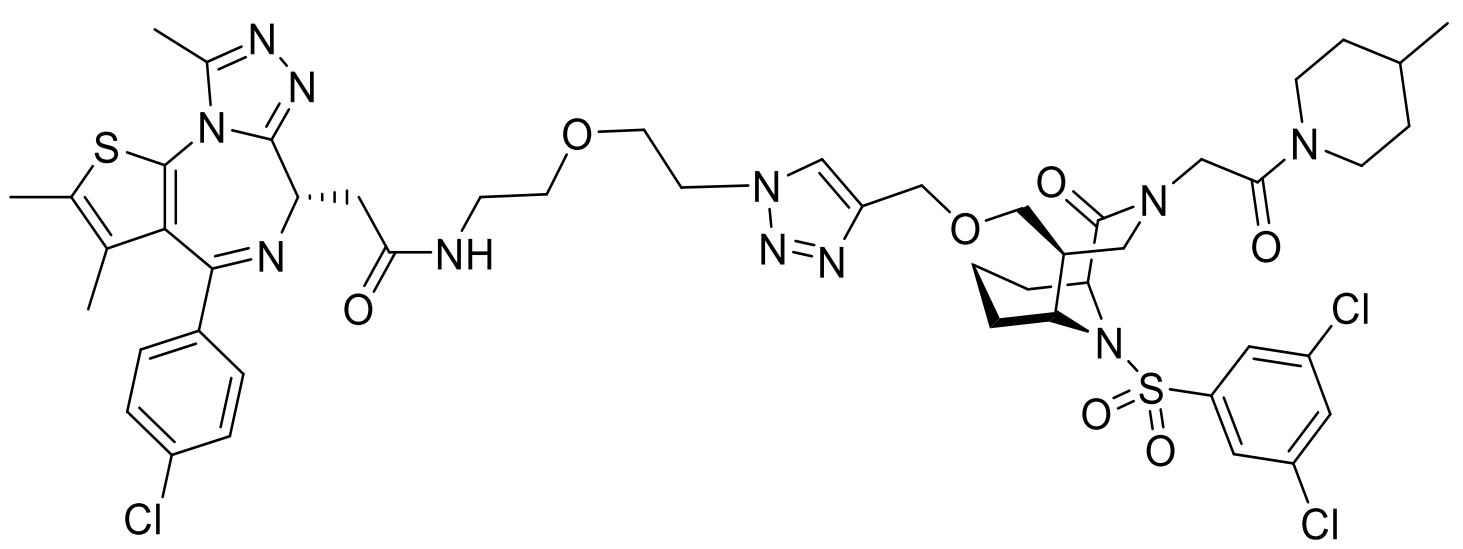

According to **GP-2**, the title compound **a1dk** was synthesized from compound **a1** (5.1 mg, 10 µmol, 1.00 eq.) and (*1S,5S,6R*)-10-((3,5-dichlorophenyl)sulfonyl)-3-(3-(4-methylpiperidin-1-yl)-3-oxopropyl)-5-((prop-2-yn-1-yloxy)methyl)-3,10-diazabicyclo[4.3.1]decan-2-one [22] (5.7 mg, 10 µmol, 1.00 eq.). The obtained product was further purified by prep. HPLC (5-100 % B).

Yield: 6 mg (6 µmol, 60 %).

Purity: 96 % (HPLC, UV-absorption 220 nm)

Physical State: yellow solid.

HRMS (ESI, m/z) calcd. for $C_{49}H_{59}Cl_3N_{11}O_7S_2$ [M+H$^+$]:1082.31004, found: 1082.31015.

(5*S*)-10-((3,5-dichlorophenyl)sulfonyl)-5-((*R*)-1-hydroxyethyl)-3-(pyridin-2-ylmethyl)-3,10-diazabicyclo[4.3.1]decan-2-one[22]

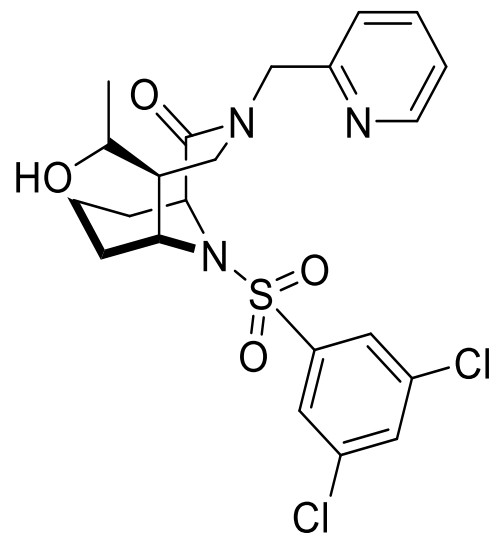

In a heatgun-dried 50 ml round flask 1.36 g of the (5*S*)-10-((3,5-dichlorophenyl)sulfonyl)-2-oxo-3-(pyridin-2-ylmethyl)-3,10-diazabicyclo[4.3.1]decane-5-carbaldehyde[22] was dissolved in 28 ml THF under argon atmosphere and cooled to 0°C. To the solution 0.91 ml (2.72 mmol, 1.2 eq) MeMgBr (3M solution in Et_2O) was added and the reaction mixture stirred for 17 hours at room temperature. The reaction was quenched with (100 ml) sat. NH_4Cl solution. The aqueous phase was separated and extracted with DCM (3 x 60 ml). The combined organic layers were dried over $MgSO_4$ and the solvent removed under reduced pressure. The crude product was applied on (3 g) Silica and purified by column chromatography (50 g SiO_2, 40-100 % EA in Cy) to obtain the desired product.

Yield: 234 mg (6 µmol, 21 %).

Purity: 96 % (HPLC, UV-absorption 220 nm)

Physical State: off white foam.

HRMS (ESI, m/z) **:** calculated for $C_{22}H_{26}Cl_2N_3O_4S^+$ [M+H]$^+$: 498.1016, found: 484.1015.

^{1}H-NMR (500 MHz, $CDCl_3$,) δ 8.51 (dt, *J* = 5.8, 2.3 Hz, 1H), 7.74 – 7.65 (m, 3H), 7.56 (t, *J*

= 1.9 Hz, 1H), 7.36 – 7.30 (m, 1H), 7.24 – 7.18 (m, 1H), 4.97 (dd, *J* = 15.0, 2.2 Hz, 1H), 4.76 (tq, *J* = 6.1, 1.9 Hz, 1H), 4.63 (dd, *J* = 15.0, 10.0 Hz, 1H), 4.20 – 4.11 (m, 1H), 3.94 (dd, *J* = 14.3, 10.6 Hz, 0.5H), 3.85 (dd, *J* = 14.6, 10.5 Hz, 0.5H), 3.75 (ddd, *J* = 10.8, 6.6, 4.9 Hz, 1H), 3.36 (ddd, *J* = 14.3, 11.3, 1.8 Hz, 1H), 2.34 – 2.25 (m, 1H), 1.58 – 1.39 (m, 5H), 1.29 – 1.21 (m, 2H), 1.16 (d, *J* = 6.4 Hz, 1.5H), 1.12 (d, *J* = 6.3 Hz, 1.5H) ppm.

^{13}C-NMR (125 MHz, $CDCl_3$) δ = 170.3, 170.4, 157.1, 157.1, 149.3, 149.2, 144.1, 144.1, 137.3, 137.2, 136.5, 132.9, 125.1, 125.1, 122.9, 122.8, 122.6, 66.8, 57.2, 57.2, 56.1, 52.7, 52.1, 50.8, 50.6, 48.9, 48.1, 28.6, 28.5, 28.2, 28.1, 21.0, 20.1, 15.6, 15.5 ppm.

(5*S*)-10-((3,5-dichlorophenyl)sulfonyl)-5-((*R*)-1-(prop-2-yn-1-yloxy)ethyl)-3-(pyridin-2-ylmethyl)-3,10-diazabicyclo[4.3.1]decan-2-one[22]

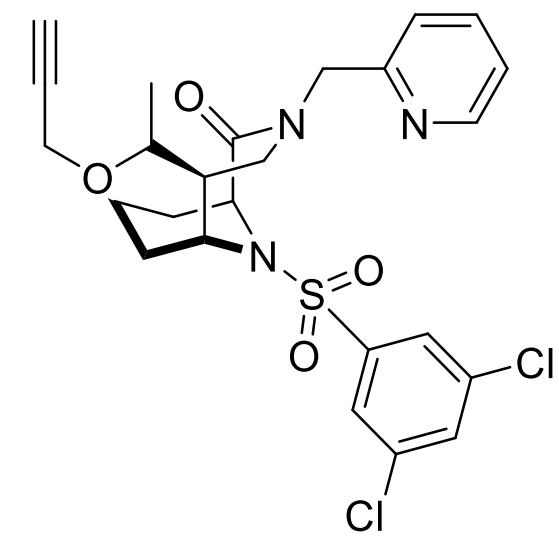

(5*S*)-10-((3,5-dichlorophenyl)sulfonyl)-5-((*R*)-1-hydroxyethyl)-3-(pyridin-2-ylmethyl)-3,10-diazabicyclo[4.3.1]decan-2-one (50 mg, 100 µmol, 1.0 eq.), was dissolved in DMF (dry, 5 mL) at 0 °C. Sodium hydride (13 mg, 300 µmol, 3.0 eq.) was added and the mixture was stirred for 30 min at 0 °C. 3-Bromoprop-1-yne (48 mg, 400 µmol, 4.0 eq.) and tetrabutylammonium iodide (1.8 mg, 5.0 µmol, 0.05 eq.) were added, the mixture was allowed to warm to room temperature and was stirred for 18 h. Saturated ammonium chloride solution (30 mL) was added and the aqueous solution was extracted with EA (3 x 30 mL). The combined organic phases were dried over MgSO4 and concentrated under reduced pressure. The obtained product was purified by chromatography (25 g SiO2, CH:EA = 1:1) and 2 times prep.HPLC (5-100 % B).

Yield: 25 mg (50 µmol, 25 %).

Purity: 95 % (HPLC, UV-absorption 220 nm)

Physical State: White solid.

HRMS (ESI, m/z) **:** calculated for $C_{25}H_{27}Cl_2N_3O_4S^+$ $[M+H]^+$: 536.1178, found: 536.1176.
The mixture was then separated by Chiral HPLC (Eluent A: CO_2 70% Eluent B: Isopropanol 30%) and the separated 2 isomers were applied to click reaction.

The mixture: **^{1}H NMR** (500 MHz, $CDCl_3$) δ 9.02 – 8.71 (m, 1H), 8.35 (ddd, *J* = 9.8, 3.6, 1.8 Hz, 1H), 7.81 (td, *J* = 14.3, 7.3 Hz, 2H), 7.71 (dd, *J* = 7.9, 1.8 Hz, 2H), 7.58 (dt, *J* = 4.4, 1.8 Hz, 1H), 5.61 (d, *J* = 16.5 Hz, 0.5H), 5.52 (d, *J* = 16.5 Hz, 0.5H), 4.82 – 4.62 (m, 2H), 4.28 (d, *J* = 6.2 Hz, 0.5H), 4.22 (d, *J* = 2.4 Hz, 0.5H), 4.18 (dd, *J* = 8.9, 4.5 Hz, 1H), 4.09 (dd, *J* = 4.5, 2.3 Hz, 0.5H), 4.06 (dd, *J* = 4.3, 2.3 Hz, 0.5H), 3.97 (dd, *J* = 14.2, 10.8 Hz, 0.5H), 3.82 (dd, *J* = 14.3, 10.6 Hz, 0.5H), 3.74 – 3.67 (m, 0.5H), 3.66 – 3.59 (m, 0.5H), 3.29 (dd, *J* = 14.1, 3.0 Hz, 1H), 2.45 (q, *J* = 2.3 Hz, 1H), 2.36 – 2.17 (m, 2H), 1.70 – 1.39 (m, 4H), 1.36 – 1.25 (m, 1H), 1.19 (d, *J* = 6.3 Hz, 3H).

^{13}C NMR (126 MHz, $CDCl_3$) δ 172.03, 171.95, 161.23, 160.92, 153.65, 153.58, 145.33, 145.28, 143.56, 142.49, 142.44, 136.41, 133.00, 132.96, 125.33, 125.25, 125.03, 124.97, 116.72, 114.42, 79.55, 79.43, 74.73, 74.35, 74.01, 56.82, 56.81, 55.82, 55.71, 52.51, 52.38, 52.14, 51.80, 49.76, 49.67, 49.19, 48.83, 28.19, 28.01, 27.61, 27.58, 15.64, 15.33, 15.29, 15.20.

e or f ^{1}H-NMR (500 MHz, $CDCl_3$,) *δ* 8.54 (ddd, *J* = 5.0, 1.9, 1.0 Hz, 1H), 7.77 (d, *J* = 1.8 Hz, 2H), 7.69 (td, *J* = 7.7, 1.8 Hz, 1H), 7.57 (t, *J* = 1.9 Hz, 1H), 7.38 – 7.24 (m, 1H), 7.21 (ddd, *J* = 7.5, 4.9, 1.2 Hz, 1H), 4.98 (d, *J* = 15.1 Hz, 1H), 4.88 (dt, *J* = 6.1, 1.9 Hz, 1H), 4.61 (d, *J* = 15.0 Hz, 1H), 4.28 – 4.25 (m, 1H), 4.21 – 4.17 (m, 1H), 4.05 – 4.02 (dd, *J* = 14.4, 1.6 Hz, 1H), 3.65 – 3.57 (m, 2H), 3.19 (dd, *J* = 14.4, 1.6 Hz, 1H), 2.45 (t, *J* = 2.4 Hz, 1H), 2.35 – 2.34 (m, 1H), 2.19 – 2.13 (m, 1H), 1.62 – 1.45 (m, 5H), 1.05 (d, *J* = 6.3 Hz, 3H).

^{13}C-NMR (126 MHz, CDCl3) δ 170.39, 156.99, 149.17, 143.94, 136.96, 136.14, 132.58, 125.21, 122.51, 122.22, 79.61, 74.55, 74.20, 57.00, 55.90, 55.83, 51.89, 48.46, 47.76, 28.12, 27.92, 15.78, 15.58.

2-((*S*)-4-(4-chlorophenyl)-2,3,9-trimethyl-6H-thieno[3,2-f][1,2,4]triazolo[4,3-a][1,4]diazepin-6-yl)-N-(2-(2-(4-(((*R*)-1-((1*S*,5*R*,6*R*)-10-((3,5-dichlorophenyl)sulfonyl)-2-oxo-3-(pyridin-2-ylmethyl)-3,10-diazabicyclo[4.3.1]decan-5-yl)ethoxy)methyl)-1H-1,2,3-triazol-1-yl)ethoxy)ethyl)acetamide **a1e** and **a1f**

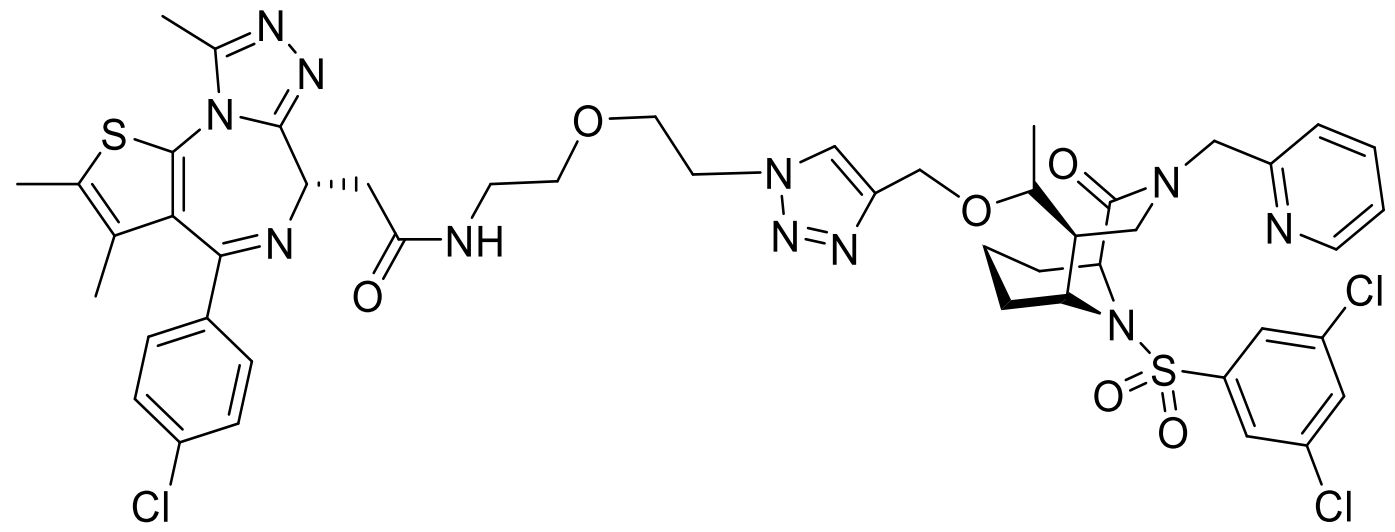

According to **GP-2**, the title compounds were synthesized from **a1** (5.7 mg, 10 µmol, 1.00 eq.)

and the separated above isomers (5.2 mg, 10 µmol, 1.00 eq.) respectively. The corresponding obtained product was further purified by prep. HPLC (5-100 % B).

Yield: 6.6 mg (7 µmol, 70 %).

Purity: 91 % (HPLC, UV-absorption 220 nm)

Physical State: yellow solid.

HRMS (ESI, m/z) calcd. for $C_{48}H_{53}Cl_3N_{11}O_6S_2$ [M+H$^+$]:1048.26818, found: 1048.26923.

Yield: 3 mg (2.8 µmol, 28 %).

Purity: 70 % (HPLC, UV-absorption 220 nm)

Physical State: yellow solid.

HRMS (ESI, m/z) calcd. for $C_{48}H_{53}Cl_3N_{11}O_6S_2$ [M+H$^+$]:1048.26818, found: 1048.26923.

2-[(9*S*)-7-(4-chlorophenyl)-4,5,13-trimethyl-3-thia-1,8,11,12-tetraazatricyclo[8.3.0.0^{2,6}]trideca-2(6),4,7,10,12-pentaen-9-yl]-1-[4-(dimethoxymethyl)piperidin-1-yl]ethan-1-one

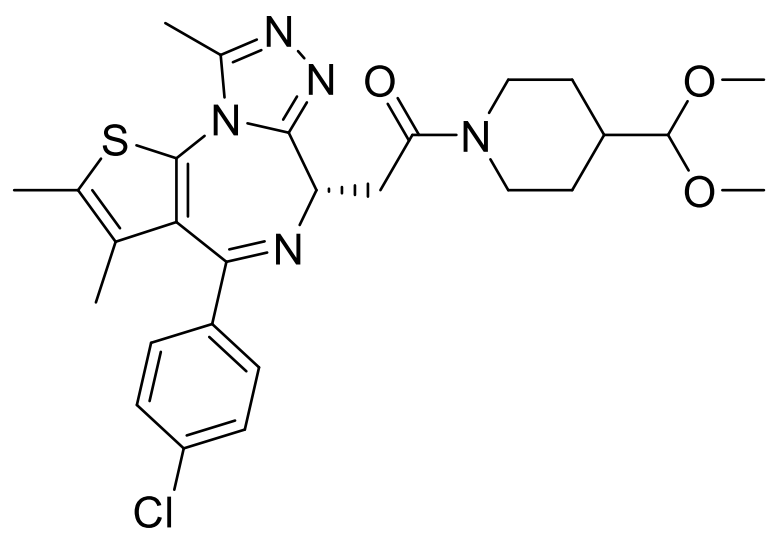

2-[(9S)-7-(4-chlorophenyl)-4,5,13-trimethyl-3-thia-1,8,11,12-tetraazatricyclo[8.3.0.0²,6]trideca-2(6),4,7,10,12-pentaen-9-yl]acetic acid (500 mg; 1.13 mmol; 1.00 eq.) and HATU (517 mg; 1.36 mmol; 1.20 eq) were given to the vial and dissolved in N,N-Dimethylformamide (10 ml;), next N-ethyldiisopropylamine (491 µl; 2.83 mmol; 2.50 eq) was added. The mixture was stirred at RT for 15 minutes and 4-(dimethoxymethyl)piperidine (202 mg; 1.25 mmol; 1.10 eq.) added afterwards. The vial was sealed and stirred over night at room temperature

To the reaction mixture was added demineralized water and extraction carried out three times with EtOAc till there was no remaining product in water layer. The combined organic layers were washed with saturated NaCl-solution twice. Thereafter the organic layers were dried over sodium sulphate, filtered and all volatile ingredients were removed under reduced pressure. Next the crude product was purified with NP column chromatography, the pure fractions were combined and concentrated under reduced pressure to give 2-[(9S)-7-(4-chlorophenyl)-4,5,13-trimethyl-3-thia-1,8,11,12-tetraazatricyclo[8.3.0.0^{2,6}]trideca-2(6),4,7,10,12-pentaen-9-yl]-

1-[4-(dimethoxymethyl)piperidin-1-yl]ethan-1-one (468 mg; 0.830 mmol; 73%).

Yield: 468 mg (0.830 mmol; 73%)
Purity: 96 % (HPLC, UV-absorption 254 nm)
Physical State: brown solid.
LC-MS (ESI, m/z) calcd. for C27H33ClN5O3S [M+H+]:542.2, found: 542.2.

1-{2-[(9*S*)-7-(4-chlorophenyl)-4,5,13-trimethyl-3-thia-1,8,11,12-tetraazatricyclo[8.3.0.0^{2,6}]trideca-2(6),4,7,10,12-pentaen-9-yl]acetyl}piperidine-4-carbaldehyde

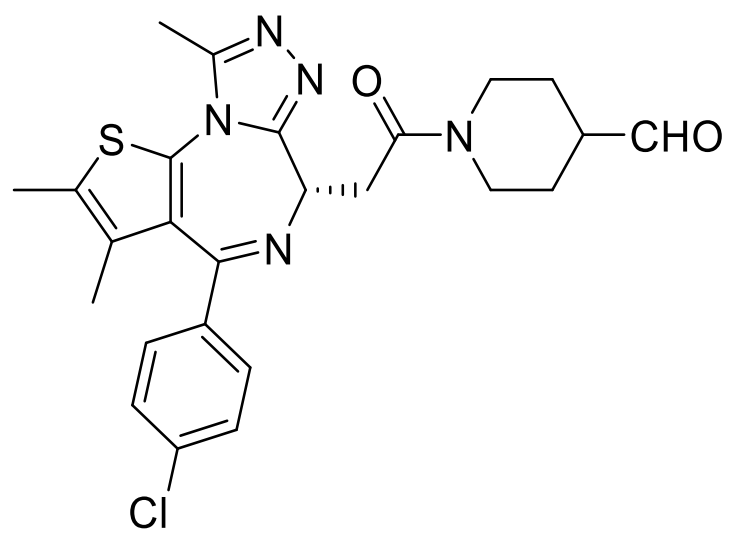

To a stirred solution of 2-[(9*S*)-7-(4-chlorophenyl)-4,5,13-trimethyl-3-thia-1,8,11,12-tetraazatricyclo[8.3.0.0^{2,6}]trideca-2(6),4,7,10,12-pentaen-9-yl]-1-[4-(dimethoxymethyl)piperidin-1-yl]ethan-1-one (200 mg; 0.353 mmol; 1.00 eq) in DCM (2.50 ml) was added trifluoroacetic acid (1.00 ml) and water (100 µl) dropwise and the reaction mixture was stirred at room temperature for 2 h.
The reaction mixture was concentrated under reduced pressure to furnish 1-{2-[(9*S*)-7-(4-chlorophenyl)-4,5,13-trimethyl-3-thia-1,8,11,12-tetraazatricyclo[8.3.0.0^{2,6}]trideca-2(6),4,7,10,12-pentaen-9-yl]acetyl}piperidine-4-carbaldehyde (311 mg; 0.350 mmol; 100%) as crude product.

Yield: 311 mg (0.350 mmol; 100%)
Purity: 56 % (HPLC, UV-absorption 254 nm)
Physical State: yellow solid.
LC-MS (ESI, m/z) calcd. for $C_{25}H_{27}ClN_5O_2S$ [M+H+]:496.3, found: 496.1.

tert-butyl 4-azidopiperidine-1-carboxylate

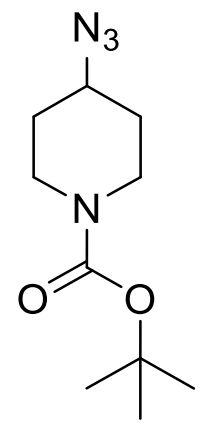

To a solution of 1-Boc-4-bromopiperidine (1.70 g; 6.43 mmol; 1.00 eq) in *N,N*-Dimethylformamide (17,0 ml) was added sodium azide (481 mg; 7,40 mmol; 1,15 eq) and the reaction mixture was stirred at 70°C and for 16 h.

The reaction mixture was poured into brine + demineralized water and extraction carried out three times with EtOAc. The organic layers were washed with brine twice, dried via sodium sulphate and concentrated under reduced pressure to give *tert*-butyl 4-azidopiperidine-1-carboxylate (1.37 g; 5.63 mmol; 88%).

Yield: 226 mg (1.28 mmol; 88%)
Purity: 93 % (HPLC, UV-absorption 220 nm)
Physical State: colourless oil

4-azidopiperidine hydrochloride

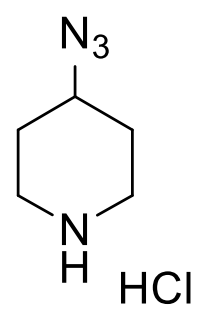

tert-butyl 4-azidopiperidine-1-carboxylate (310 mg; 1.28 mmol; 1.00 eq) was dissolved in D (600 µl) and 4M-HCl in Dioxane (1.59 mL; 6.38 mmol; 5.00 eq.) added dropwise to the stirred solution, the vial was purged with argon and sealed afterwards. The reaction mixture was stirred at RT for 2.5h.

The reaction mixture was evaporated under reduced pressure to give 4-azidopiperidine hydrochloride (226 mg; 1.28 mmol; 100%).

Yield: 1.37 g (5.63 mmol; 88%)
Purity: 93 % (HPLC, UV-absorption 220 nm)
Physical State: colourless oil

1-{4-[(4-azidopiperidin-1-yl)methyl]piperidin-1-yl}-2-[(9*S*)-7-(4-chlorophenyl)-4,5,13-

trimethyl-3-thia-1,8,11,12-tetraazatricyclo[8.3.0.0^{2,6}]trideca-2(6),4,7,10,12-pentaen-9-yl]ethan-1-one **a7**

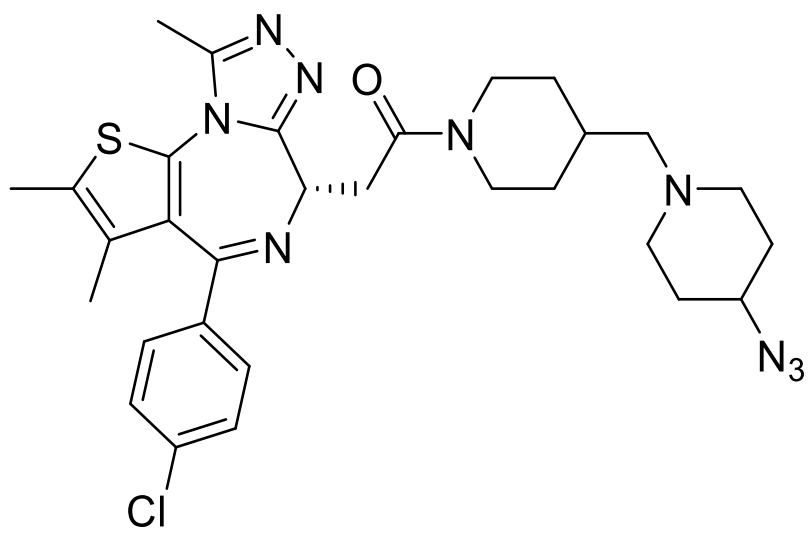

To a solution of 4-Azidopiperidine hydrochloride (60.0 mg; 0.339 mmol; 1.00 eq) and 1-{2-[(9*S*)-7-(4-chlorophenyl)-4,5,13-trimethyl-3-thia-1,8,11,12-tetraazatricyclo[8.3.0.0^{2,6}]trideca-2(6),4,7,10,12-pentaen-9-yl]acetyl}piperidine-4-carbaldehyde (311 mg; 0.353 mmol; 1.04 eq) in 1,2-DCE (1 mL) was added *N*-ethyldiisopropylamine (0.86 ml; 5.1 mmol; 15 eq) and the reaction mixture was stirred over night at RT. Sodium triacetoxyborohydride (222 mg; 1.02mmol; 3.00 eq.) was added and the reaction mixture stirred for 1.5h.

The reaction mixture was poured into demineralized water + $NaHCO_3$ and extraction carried out three times with EtOAc, till there was no remaining product in aq. layer. The combined organic layers were dried via phase separator tube concentrated under reduced pressure to give a crude material. The crude product was purified by NP column chromatography to furnish 1-{4-[(4-azidopiperidin-1-yl)methyl]piperidin-1-yl}-2-[(9S)-7-(4-chlorophenyl)-4,5,13-trimethyl-3-thia-1,8,11,12-tetraazatricyclo[8.3.0.0^{2,6}]trideca-2(6),4,7,10,12-pentaen-9-yl]ethan-1-one (91 mg; 0.15 mmol; 44%; colorless solid).

Yield: 91 mg (0.15 mmol; 45%)
Purity: > 99 % (HPLC, UV-absorption 254 nm)
Physical State: colourless solid.
LC-MS (ESI, m/z) calcd. for $C_{30}H_{37}ClN_9OS$ [M+H+]:606.3, found: 606.2.
^{1}H NMR (700 MHz, DMSO) δ 7.53 – 7.46 (m, 2H), 7.45 – 7.41 (m, 2H), 4.59 – 4.54 (m, 1H), 4.38 – 4.31 (m, 1H), 4.14 -4.09 (m, 1H), 3.63 – 3.51 (m 2H), 3.39 – 3.35 (m, 1H), 3.15 – 3.07 (m, 1H), 2.72 – 2.63 (s, 2H), 2.62 - 2.56 (m, 4H), 2.41 (s, 3H), 2.18 – 2.03 (m, 4H), 1.86 – 1.75 (m, 4H), 1.72 – 1.66 (m, 1H), 1.63 (s, 3H), 1.53 – 1.46 (m, 2H), 1.20 – 1.05 (m, 1H), 0.97 – 0.85 (m, 1H).

1-(4-{[4-(azidomethyl)piperidin-1-yl]methyl}piperidin-1-yl)-2-[(9*S*)-7-(4-chlorophenyl)-4,5,13-trimethyl-3-thia-1,8,11,12-tetraazatricyclo[8.3.0.0^{2,6}]trideca-2(6),4,7,10,12-

pentaen-9-yl]ethan-1-one **a8**

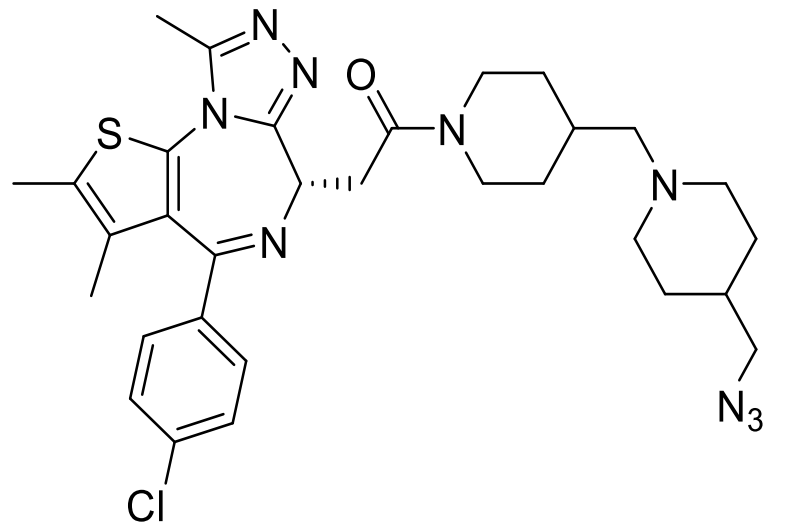

4(azidomethyl)piperidine hydrochloride (30.0 mg; 0.167 mmol; 1.00 eq), and 12-[(9*S*)-7-(4-chlorophenyl)-4,5,13-trimethyl-3-thia-1,8,11,12-tetraazatricyclo[8.3.0.0^{2,6}]trideca-2(6),4,7,10,12-pentaen-9-yl]acetyl}piperidine-4-carbaldehyde (162 mg; 0.183 mmol; 1.10 eq) and N-Ethyldiisopropylamine (0.43 ml; 2.5 mmol; 15 eq) were given into the vial, which was sealed afterwards. Next the vial was purged with argon and 1,2-dichloroethane (0.5 ml) was added. The reaction mixture was stirred at RT over night.

Next NaBH(OAc)3 (110 mg; 0.500 mmol; 3.00 eq) was added and the reaction mixture stirred for further 5 h.

Water (10 mL) and DCM (20 mL) were added to the reaction mixture, the phases were separated and the water phase was washed with DCM (2 x 20 mL). The combined organic layers were dried via phase separator tube concentrated under reduced pressure to give a crude material. The crude product was purified by NP column chromatography to furnish 1-(4-{[4-(azidomethyl)piperidin-1-yl]methyl}piperidin-1-yl)-2-[(9*S*)-7-(4-chlorophenyl)-4,5,13-trimethyl-3-thia-1,8,11,12-tetraazatricyclo[8.3.0.0^{2,6}]trideca-2(6),4,7,10,12-pentaen-9-yl]ethan-1-one (63 mg; 0.10 mmol; 56 %).

Yield: 63 mg (0.10 mmol, 56%).

Purity: 95 % (HPLC, UV-absorption 254 nm)

Physical State: white solid solid.

LC-MS (ESI, m/z) calcd. for $C_{31}H_{38}ClN_9OS$ [M+H+]:620.3, found: 620.3.

^{1}H NMR (700 MHz, DMSO) δ 7.51 – 7.46 (m, 2H), 7.46 – 7.41 (m, 2H), 4.60 – 4.55 (m, 1H), 4.38 – 4.32 (m, 1H), 4.14 – 4.09 (m, 1H), 3.60 (td, *J* = 16.9, 7.3 Hz, 1H), 3.36 (ddd, *J* = 16.4, 6.3, 2.2 Hz, 1H), 3.25 (d, *J* = 6.7 Hz, 2H), 3.15 – 3.08 (m, 1H), 2.84 (b, 2H), 2.63 – 2.54 (m, 4H), 2.42 (s, 3H), 2.13 (b, 2H), 1.91 – 1.75 (m, 2H), 1.71 – 1.61 (m, 5H), 1.53 – 1.47 (m, 1H), 1.30 – 1.06 (m, 3H), 0.95 – 0.85 (m, 1H).

(1S,5S,6R)-5-(((1-(1-((1-(2-((*S*)-4-(4-chlorophenyl)-2,3,9-trimethyl-6H-thieno[3,2-f][1,2,4]triazolo[4,3-a][1,4]diazepin-6-yl)acetyl)piperidin-4-yl)methyl)piperidin-4-yl)-1H-1,2,3-triazol-4-yl)methoxy)methyl)-10-((3,5-dichlorophenyl)sulfonyl)-3-(pyridin-2-ylmethyl)-3,10-diazabicyclo[4.3.1]decan-2-one **a7d**

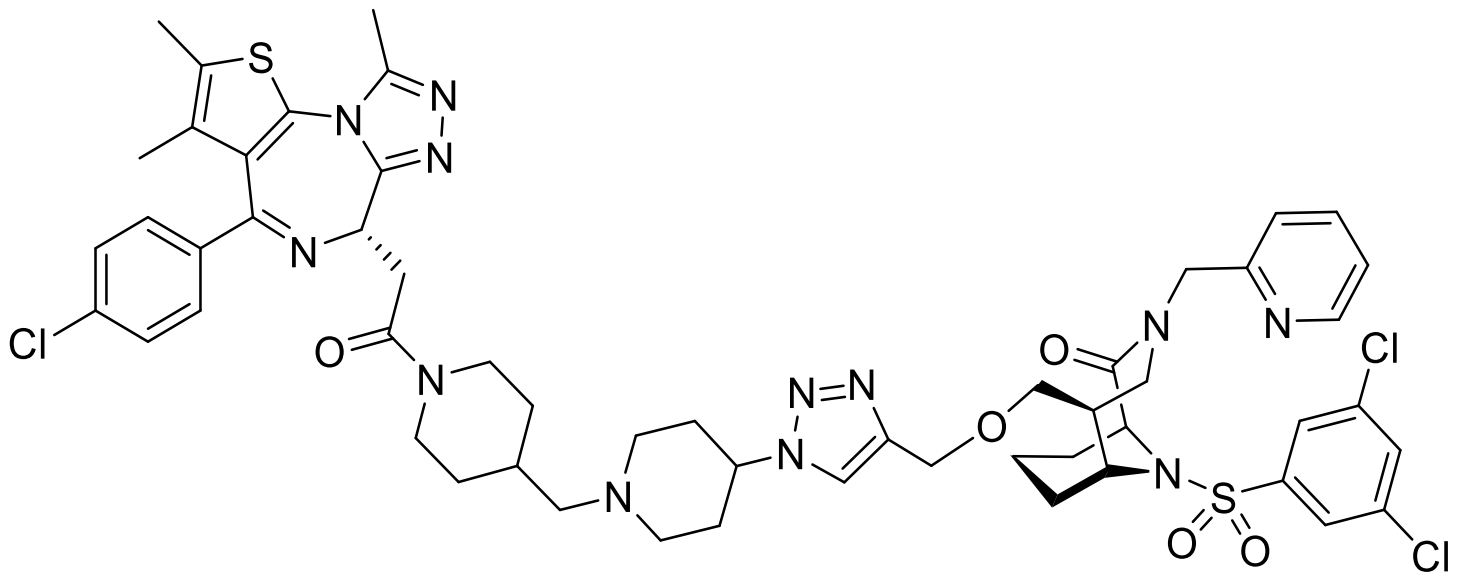

According to **GP-2**, the title compound **a7d** was synthesized from **a7** (6.1 mg, 10 µmol, 1.00 eq.) and (*1S,5S,6R*)-10-((3,5-dichlorophenyl)sulfonyl)-5-((prop-2-yn-1-yloxy)methyl)-3-(pyridin-2-ylmethyl)-3,10-diazabicyclo[4.3.1]decan-2-one [20] (5.2 mg, 10 µmol, 1.00 eq.). The obtained product was further purified by prep. HPLC (5-100 % B).

Yield: 10 mg (9 µmol, 90%).

Purity: 87 % (HPLC, UV-absorption 220 nm)

Physical State: yellow solid.

HRMS (ESI, m/z) calcd. for $C_{54}H_{62}Cl_3N_{12}O_5S_2$ [M+H$^+$]:1127.34676, found: 1127.34833.

(1S,5S,6R)-5-(1-(1-((1-(2-((*S*)-4-(4-chlorophenyl)-2,3,9-trimethyl-6H-thieno[3,2-f][1,2,4]triazolo[4,3-a][1,4]diazepin-6-yl)acetyl)piperidin-4-yl)methyl)piperidin-4-yl)-1H-1,2,3-triazol-4-yl)-10-((3,5-dichlorophenyl)sulfonyl)-3-(pyridin-2-ylmethyl)-3,10-diazabicyclo[4.3.1]decan-2-one **a7c**

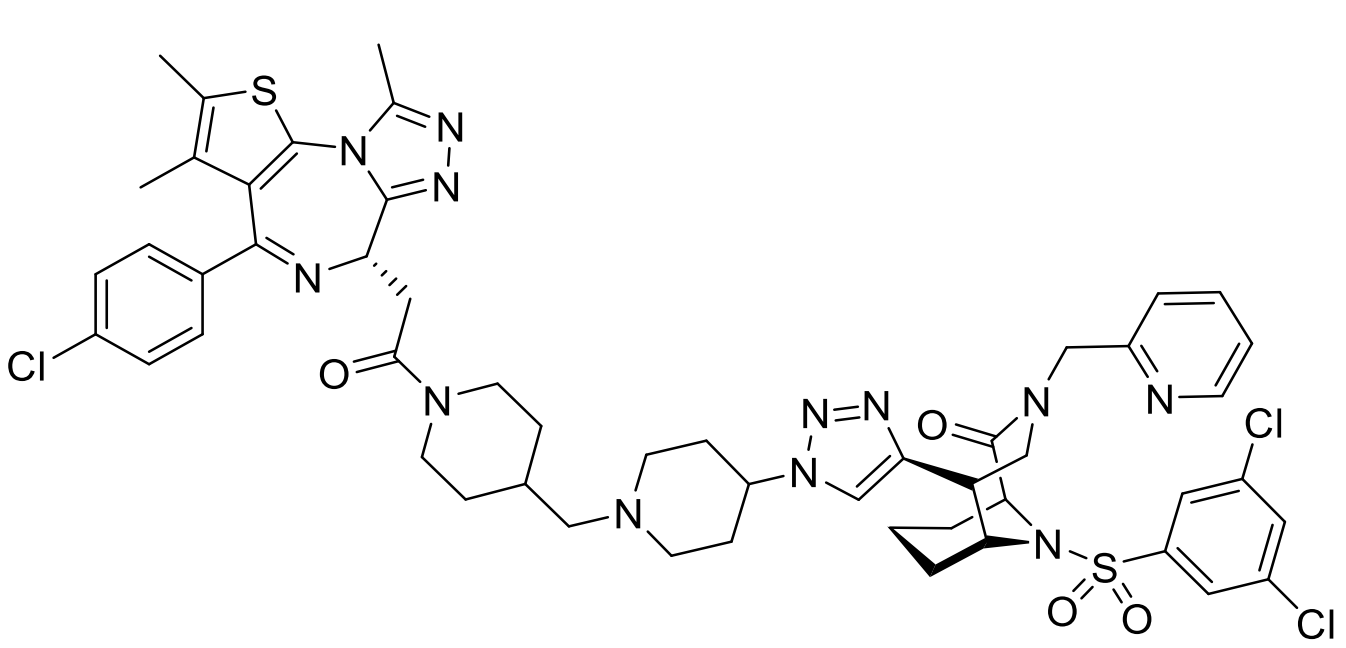

According to **GP-2**, the title compound **a7c** was synthesized from **a7** (6.1 mg, 10 µmol, 1.00 eq.) and (*1S,5S,6R*)-10-((3,5-dichlorophenyl)sulfonyl)- 5-ethynyl -3-(pyridin-2-ylmethyl)-3,10-diazabicyclo[4.3.1]decan-2-one [20] (5.2 mg, 10 µmol, 1.00 eq.). The obtained

product was further purified by prep. HPLC (5-100 % B).

Yield: 9 mg (8.4 µmol, 84%).

Purity: 94 % (HPLC, UV-absorption 220 nm)

Physical State: yellow solid.

HRMS (ESI, m/z) calcd. for $C_{52}H_{58}Cl_3N_{12}O_4S_2$ [M+H$^+$]:1083.32055, found: 1083.32190.

(1S,5S,6R)-5-(((1-((1-((1-(2-((*S*)-4-(4-chlorophenyl)-2,3,9-trimethyl-6H-thieno[3,2-f][1,2,4]triazolo[4,3-a][1,4]diazepin-6-yl)acetyl)piperidin-4-yl)methyl)piperidin-4-yl)methyl)-1H-1,2,3-triazol-4-yl)methoxy)methyl)-10-((3,5-dichlorophenyl)sulfonyl)-3-(pyridin-2-ylmethyl)-3,10-diazabicyclo[4.3.1]decan-2-one **a8d**

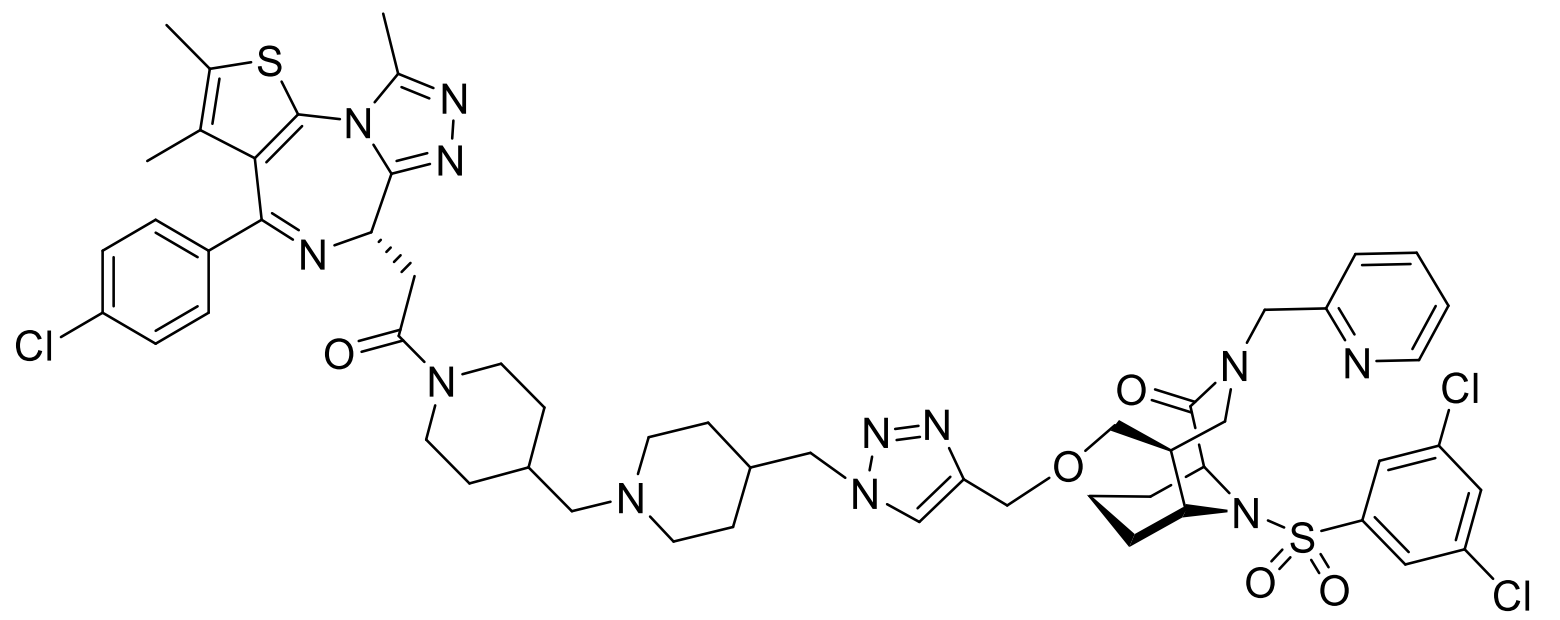

According to **GP-2**, the title compound **a8d** was synthesized from **a8** (6.2 mg, 10 µmol, 1.00 eq.) and (*1S,5S,6R*)-10-((3,5-dichlorophenyl)sulfonyl)-5-((prop-2-yn-1-yloxy)methyl)-3-(pyridin-2-ylmethyl)-3,10-diazabicyclo[4.3.1]decan-2-one [20] (5.2 mg, 10 µmol, 1.00 eq.). The obtained product was further purified by prep. HPLC (5-100 % B).

Yield: 6 mg (5.2 µmol, 52%).

Purity: 99 % (HPLC, UV-absorption 220 nm)

Physical State: yellow solid.

HRMS (ESI, m/z) calcd. for $C_{55}H_{64}Cl_3N_{12}O_5S_2$ [M+H$^+$]:1141.36241, found: 1141.36370.

(1S,5S,6R)-5-(1-((1-((1-(2-((*S*)-4-(4-chlorophenyl)-2,3,9-trimethyl-6H-thieno[3,2-f][1,2,4]triazolo[4,3-a][1,4]diazepin-6-yl)acetyl)piperidin-4-yl)methyl)piperidin-4-yl)methyl)-1H-1,2,3-triazol-4-yl)-10-((3,5-dichlorophenyl)sulfonyl)-3-(pyridin-2-ylmethyl)-3,10-diazabicyclo[4.3.1]decan-2-one **a8c**

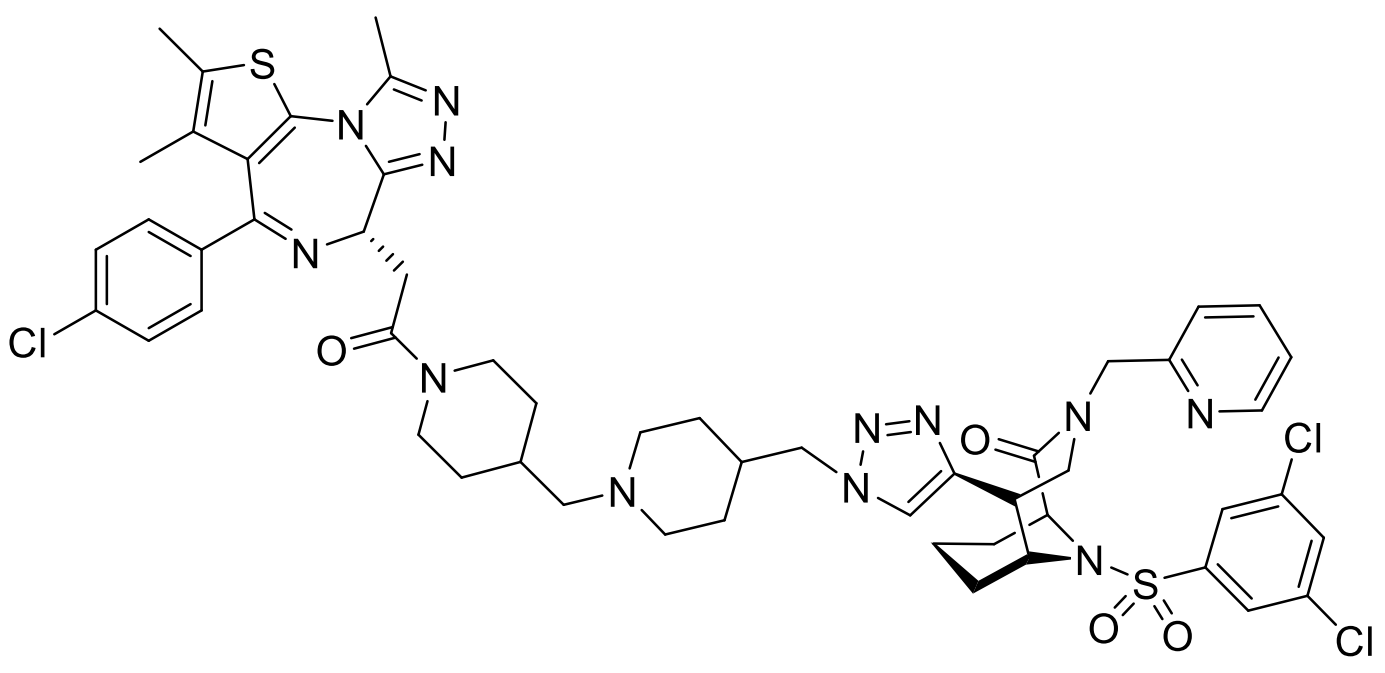

According to **GP-2**, the title compound **a8c** was synthesized from **a8** (6.2 mg, 10 µmol, 1.00 eq.) and (*1S,5S,6R*)-10-((3,5-dichlorophenyl)sulfonyl)- 5-ethynyl -3-(pyridin-2-ylmethyl)-3,10-diazabicyclo[4.3.1]decan-2-one [20] (5.2 mg, 10 µmol, 1.00 eq.). The obtained product was further purified by prep. HPLC (5-100 % B).

Yield: 8 mg (7.3 µmol, 73%).

Purity: 93 % (HPLC, UV-absorption 220 nm)

Physical State: yellow solid.

HRMS (ESI, m/z) calcd. for $C_{53}H_{60}Cl_3N_{12}O_4S_2$ [M+H$^+$]:1097.33620, found: 1097.33758.

tert-butyl *N*-(7-{2-[(9*S*)-7-(4-chlorophenyl)-4,5,13-trimethyl-3-thia-1,8,11,12-tetraazatricyclo[8.3.0.0^{2,6}]trideca-2(6),4,7,10,12-pentaen-9-yl]acetyl}-7-azaspiro[3.5]nonan-2-yl)carbamate

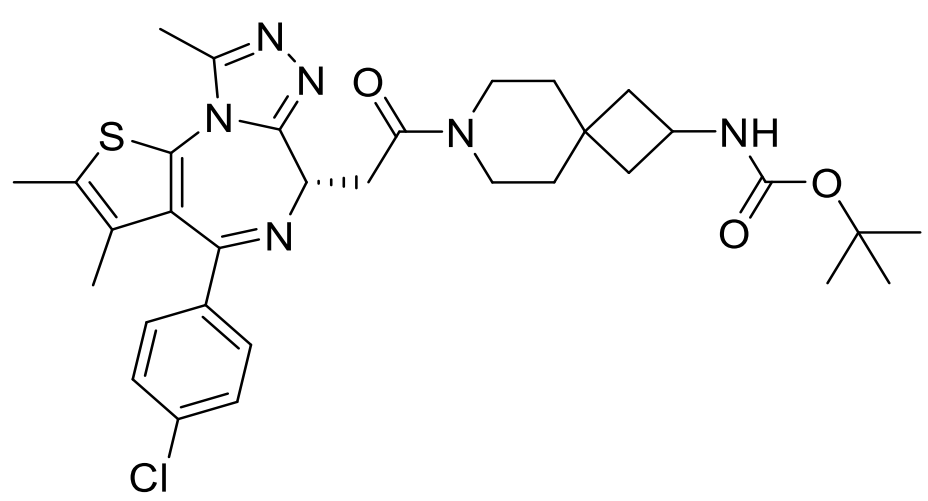

To a solution of 2-[(9*S*)-7-(4-chlorophenyl)-4,5,13-trimethyl-3-thia-1,8,11,12-tetraazatricyclo[8.3.0.0²,6]trideca-2(6),4,7,10,12-pentaen-9-yl]acetic acid (172 mg; 0.41 mmol; 0.86 eq.) and HATU (209 mg; 0.548 mmol; 1.15 eq.) in DMF (4 mL) was added *N*Ethyldiisopropylamine (207 µl; 1.19 mmol; 2.50 eq.). The mixture was stirred at RT for 15 minutes and *t*-butyl *N*-{7-azaspiro[3.5]nonan-2-yl}carbamate (133 mg; 0.53 mmol; 1.10 eq.) was added and it was stirred for 90 min at room temperature.

To the reaction mixture was added demineralized water and extraction carried out three times with DCM till there was no remaining product in water layer. Thereafter the organic layers were combined and dried via phase separator tube and concentrated in under reduced pressure. Next the crude product was purified with NP-column chromatography, the clean fractions were combined and concentrated under reduced pressure to furnish tert-butyl N-(7-{2-[(9S)-7-(4-

chlorophenyl)-4,5,13-trimethyl-3-thia-1,8,11,12-tetraazatricyclo[8.3.0.0^{2,6}]trideca-2(6),4,7,10,12-pentaen-9-yl]acetyl}-7-azaspiro[3.5]nonan-2-yl)carbamate (335 mg; 0.43 mmol; 91%).

Yield: 355 mg (0.43 mmol, 91%).
Purity: 81 % (HPLC, UV-absorption 254 nm)
Physical State: pale yellow solid.
LC-MS (ESI, m/z) calcd. for $C_{32}H_{40}ClN_6O_3S$ [M+H+]:623.2, found: 623.2.

1-{2-amino-7-azaspiro[3.5]nonan-7-yl}-2-[(9*S*)-7-(4-chlorophenyl)-4,5,13-trimethyl-3-thia-1,8,11,12-tetraazatricyclo[8.3.0.0^{2,6}]trideca-2(6),4,7,10,12-pentaen-9-yl]ethan-1-one hydrochloride

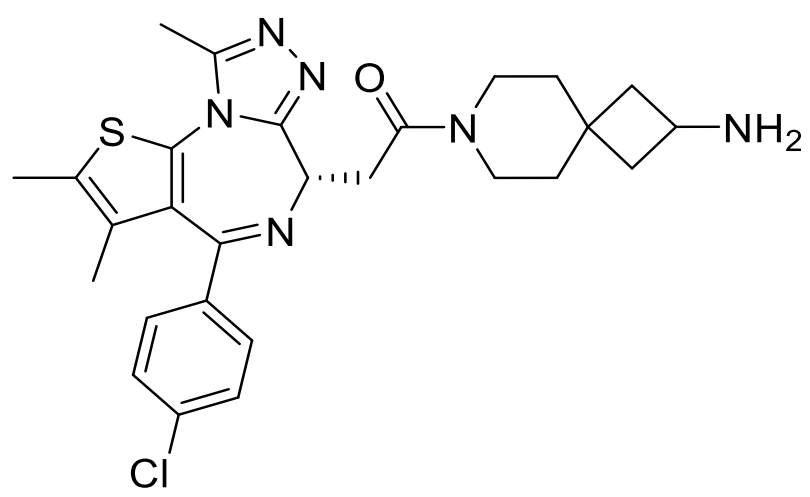

tert-butyl *N*-(7-{2-[(9*S*)-7-(4-chlorophenyl)-4,5,13-trimethyl-3-thia-1,8,11,12-tetraazatricyclo[8.3.0.0^{2,6}]trideca-2(6),4,7,10,12-pentaen-9-yl]acetyl}-7-azaspiro[3.5]nonan-2-yl)carbamate (335 mg; 0.433 mmol; 1.00 eq.) was dissolved in D (5 ml) and 4M-HCl in Dioxane (5 ml) added dropwise to the stirred solution. The reaction mixture was stirred at RT for 1.5h.
The reaction mixture was concentrated under reduced pressure to give a crude material, 1-{2-amino-7-azaspiro[3.5]nonan-7-yl}-2-[(9S)-7-(4-chlorophenyl)-4,5,13-trimethyl-3-thia-1,8,11,12-tetraazatricyclo[8.3.0.0^{2,6}]trideca-2(6),4,7,10,12-pentaen-9-yl]ethan-1-one hydrochloride (399 mg; 0.430 mmol; 100%).

Yield: 399 mg (0.43 mmol, 100%).
Purity: 61 % (HPLC, UV-absorption 254 nm)
Physical State: pale yellow solid.
LC-MS (ESI, m/z) calcd. for $C_{27}H_{32}ClN_6OS$ [M+H+]:523.2, found: 523.2.

1-{2-azido-7-azaspiro[3.5]nonan-7-yl}-2-[(9*S*)-7-(4-chlorophenyl)-4,5,13-trimethyl-3-thia-1,8,11,12-tetraazatricyclo[8.3.0.0^{2,6}]trideca-2(6),4,7,10,12-pentaen-9-yl]ethan-1-one **a13**

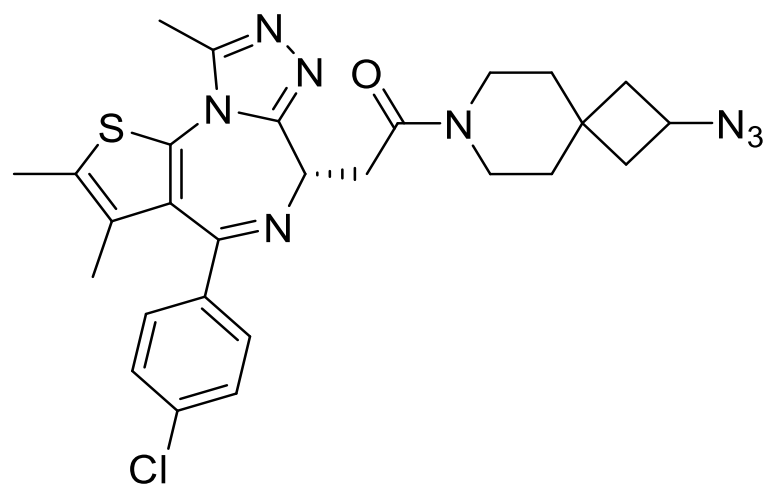

To a solution of 1-{2-amino-7-azaspiro[3.5]nonan-7-yl}-2-[(9*S*)-7-(4-chlorophenyl)-4,5,13-trimethyl-3-thia-1,8,11,12-tetraazatricyclo[8.3.0.0^{2,6}]trideca-2(6),4,7,10,12-pentaen-9-yl]ethan-1-one hydrochloride (399 mg; 0.433 mmol; 1.00 eq.) in DMSO & MTBE (1:1) (v/v) (9 ml) was added potassium hydroxide solution (aq.) (2.6 mmol; 6.00 eq.; 865.66 µl). *N*-diazosulfamoyl fluoride (5 eq) in MTBE (4 ml) and Dimethyl sulfoxide (4 ml) was added and the reaction mixture was stirred at RT for 1 h

The reaction mixture was acidified with 1M HCl till pH = 1 was met. Next the reaction mixture was poured into demineralized water and extraction carried out three times with EtOAc, till there was no remaining product in aq. layer. The combined organic layers were dried via phase separator tube and concentrated under reduced pressure to give a crude material. The crude product was fused onto Isolute H-MN and purified with RP-chromatography (see attached file), next the pure fractions were combined and concentrated. Next added demineralized water basified with a saturated sodium bicarbonate solution and extraction carried out three times with EtOAc till there was no remaining product in water layer. Thereafter the combined organic layers were concentrated under reduced pressure, resolved in MeCN/water, freezed in a dry-ice/acetone bath and lyophilized overnight in freeze dryer to give 1-{2-azido-7-azaspiro[3.5]nonan-7-yl}-2-[(9S)-7-(4-chlorophenyl)-4,5,13-trimethyl-3-thia-1,8,11,12-tetraazatricyclo[8.3.0.0^{2,6}]trideca-2(6),4,7,10,12-pentaen-9-yl]ethan-1-one (172 mg; 0.310 mmol; 34%).

Yield: 172 mg (0.31 mmol, 34%).

Purity: 100 % (HPLC, UV-absorption 254 nm)

Physical State: pale yellow solid.

LC-MS (ESI, m/z) calcd. for $C_{27}H_{30}ClN_8OS$ [M+H+]:549.2, found: 549.2.

^{1}H NMR (500 MHz, DMSO) δ 7.51 – 7.47 (m, 2H), 7.45 – 7.41 (m, 2H), 4.58 – 4.54j (t, *J* =

6.7 Hz, 1H), 4.12 – 4.00 (m, 1H), 3.65 – 3.56 (m, 2H), 3.53 – 3.50 (m, 1H), 3.46 – 3.35 (m, 3H), 2.59 (s, 3H), 2.41 (s, 3H), 2.31 – 2.22 (m, 2H), 1.87 – 1.77 (m, 2H), 1.70 – 1.58 (m, 5H), 1.51 – 1.44 (m, 2H).

(*1S,5S,6R*)-5-(((1-(7-(2-((*S*)-4-(4-chlorophenyl)-2,3,9-trimethyl-6H-thieno[3,2-f][1,2,4]triazolo[4,3-a][1,4]diazepin-6-yl)acetyl)-7-azaspiro[3.5]nonan-2-yl)-1H-1,2,3-triazol-4-yl)methoxy)methyl)-10-((3,5-dichlorophenyl)sulfonyl)-3-(pyridin-2-ylmethyl)-3,10-diazabicyclo[4.3.1]decan-2-one **a13d**

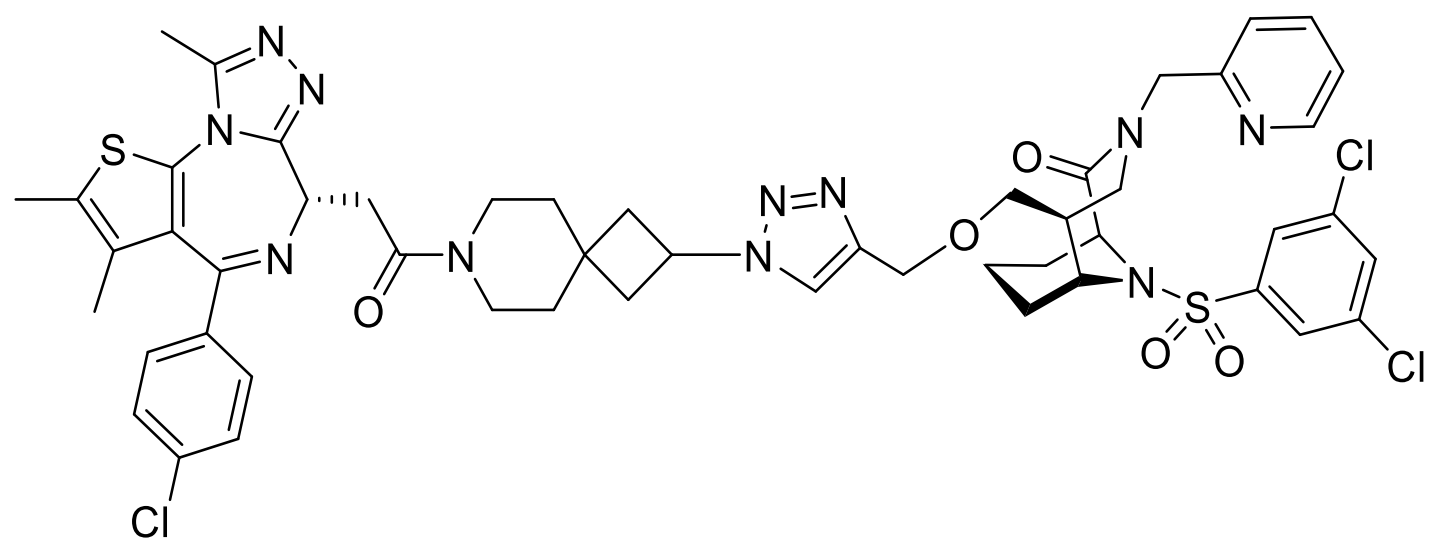

According to **GP-2**, the title compound **a13d** was synthesized from **a13** (5.5 mg, 10 µmol, 1.00 eq.) and (1S,5S,6R)-10-((3,5-dichlorophenyl)sulfonyl)-5-((prop-2-yn-1-yloxy)methyl)-3-(pyridin-2-ylmethyl)-3,10-diazabicyclo[4.3.1]decan-2-one [20] (5.2 mg, 10 µmol, 1.00 eq.). The obtained product was further purified by prep. HPLC (5-100 % B).

Yield: 6.4 mg (6.0 µmol, 60 %).

Purity: 88 % (HPLC, UV-absorption 220 nm)

Physical State: yellow solid.

HRMS (ESI, m/z) calcd. for $C_{51}H_{55}Cl_3N_{11}O_5S_2$ [M+H$^+$]:1070.28892, found: 1070.29133.

(*1S,5S,6R*)-5-(1-(7-(2-((*S*)-4-(4-chlorophenyl)-2,3,9-trimethyl-6H-thieno[3,2-f][1,2,4]triazolo[4,3-a][1,4]diazepin-6-yl)acetyl)-7-azaspiro[3.5]nonan-2-yl)-1H-1,2,3-triazol-4-yl)-10-((3,5-dichlorophenyl)sulfonyl)-3-(pyridin-2-ylmethyl)-3,10-diazabicyclo[4.3.1]decan-2-one **a13c**

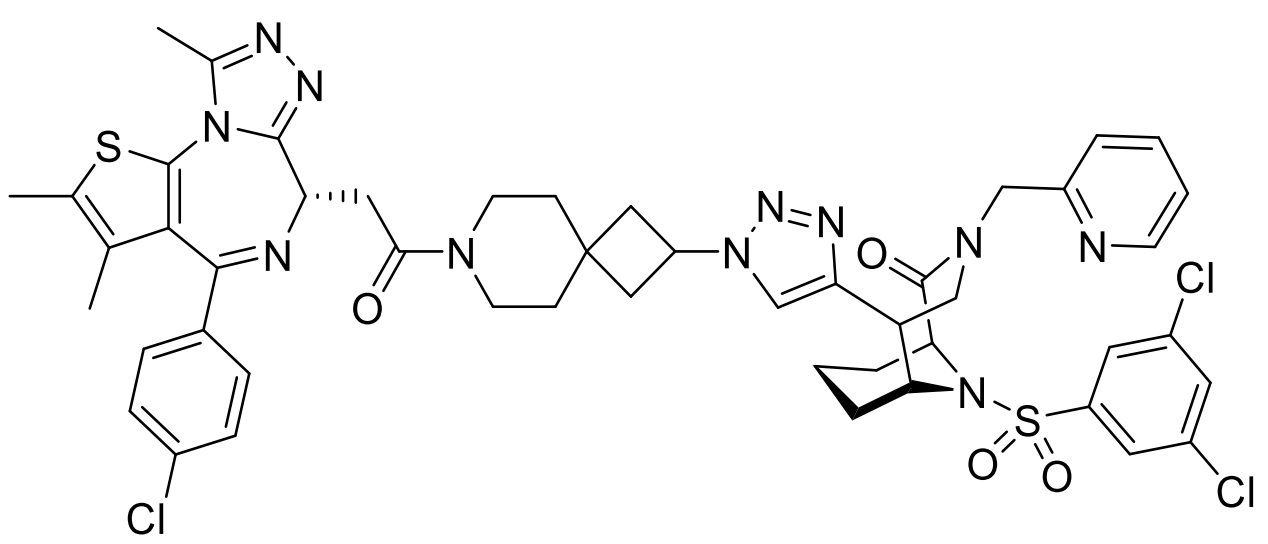

According to **GP-2**, the title compound **a13c** was synthesized from **a13** (8.3 mg, 15 µmol, 1.00 eq.) and (1S,5S,6R)-10-((3,5-dichlorophenyl)sulfonyl)- 5-ethynyl -3-(pyridin-2-

ylmethyl)-3,10-diazabicyclo[4.3.1]decan-2-one [20] (7.2 mg, 15 µmol, 1.00 eq.). The obtained product was further purified by prep. HPLC (5-100 % B).

Yield: 11 mg (11 µmol, 73%).

Purity: 97 % (HPLC, UV-absorption 220 nm)

Physical State: yellow solid.

HRMS (ESI, m/z) calcd. for $C_{49}H_{51}Cl_3N_{11}O_4S_2$ [M+H$^+$]:1026.26270, found: 1026.26406.

^{1}H NMR (500 MHz, DMSO) δ 8.61 (d, *J* = 5.0 Hz, 1H), 8.13 (d, *J* = 11.6 Hz, 1H), 8.05 (d, *J* = 1.8 Hz, 2H), 8.02 – 7.95 (m, 2H), 7.48 – 7.32 (m, 6H), 5.25 – 5.12 (m, 1H), 4.89 (dd, *J* = 18.3, 3.8 Hz, 2H), 4.76 (d, *J* = 16.0 Hz, 1H), 4.59 (t, *J* = 6.8 Hz, 1H), 4.48 (t, *J* = 6.0 Hz, 1H), 4.09 (dd, *J* = 14.2, 10.8 Hz, 2H), 3.52 – 3.30 (m, 7H), 2.60 (s, 3H), 2.54-2.52 (m, 2H), 2.41 (s, 3H), 2.36 – 2.22 (m, 2H), 2.18 – 2.00 (m, 1H), 1.91 – 1.41 (m, 10H), 1.42 – 1.12 (m, 2H).

^{13}C NMR (126 MHz, DMSO) δ 170.1, 167.9, 163.0, 158.7, 158.4, 158.1, 157.8, 156.0, 155.3, 149.8, 147.3, 147.1, 143.7, 139.3, 136.7, 135.5, 135.2, 132.8, 132.1, 130.8, 130.2, 129.9, 129.7, 128.5, 125.2, 123.1, 122.0, 120.6, 56.1, 54.8, 54.2, 52.2, 49.0, 42.5, 42.1, 41.0, 40.4, 38.3, 38.2, 35.4, 34.8, 34.7, 32.2, 32.2, 27.1, 26.7, 14.7, 14.0, 12.7, 11.2.

1-{2-[(9*S*)-7-(4-chlorophenyl)-4,5,13-trimethyl-3-thia-1,8,11,12-tetraazatricyclo[8.3.0.0^{2,6}]trideca-2(6),4,7,10,12-pentaen-9-yl]acetyl}piperidin-4-one

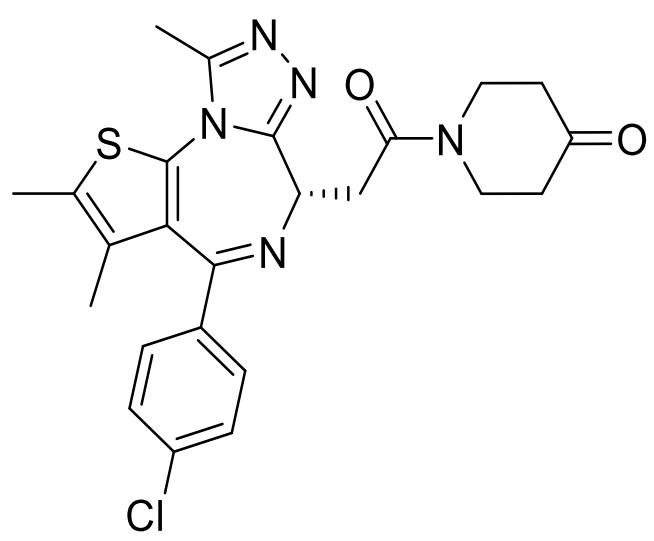

To a solution of 2-[(9S)-7-(4-chlorophenyl)-4,5,13-trimethyl-3-thia-1,8,11,12-tetraazatricyclo[8.3.0.0^2,6]trideca-2(6),4,7,10,12-pentaen-9-yl]acetic acid (500 mg; 1.13 mmol; 1.00 eq.) and HATU (495 mg; 1.30 mmol; 1.15 eq.) in *N,N*-Dimethylformamide (10 ml) was added *N*-ethyldiisopropylamine (491 µl; 2.83 mmol; 2.50 eq.), the mixture was stirred at RT for 15 min and piperidin-4-one hydrochloride (169 mg; 1.25 mmol; 1.10 eq.) was added afterwards. The vial was sealed and stirred for 16 h at room temperature.

To the reaction mixture was added demineralized water and extraction carried out three times with EtOAc till there was no remaining product in water layer. Thereafter the organic layers were combined and dried via phase separator tube and concentrated under reduced pressure. Next the crude product was purified with NP-column chromatography, the clean fractions were

combined and concentrated under reduced pressure to furnish 1-{2-[(9*S*)-7-(4-chlorophenyl)-4,5,13-trimethyl-3-thia-1,8,11,12-tetraazatricyclo[8.3.0.0^{2,6}]trideca-2(6),4,7,10,12-pentaen-9-yl]acetyl}piperidin-4-one (483 mg; 1.00 mmol; 89%).

Yield: 483 mg (1.00 mmol, 89%).
Purity: 100 % (HPLC, UV-absorption 254 nm)
Physical State: pale yellow solid.
LC-MS (ESI, m/z) calcd. for $C_{24}H_{25}ClN_5O_2S$ [M+H+] 482.1, found: 482.1.

1-[4-(azidomethyl)-[1,4'-bipiperidin]-1'-yl]-2-[(9*S*)-7-(4-chlorophenyl)-4,5,13-trimethyl-3-thia-1,8,11,12-tetraazatricyclo[8.3.0.0^{2,6}]trideca-2(6),4,7,10,12-pentaen-9-yl]ethan-1-one **a10**

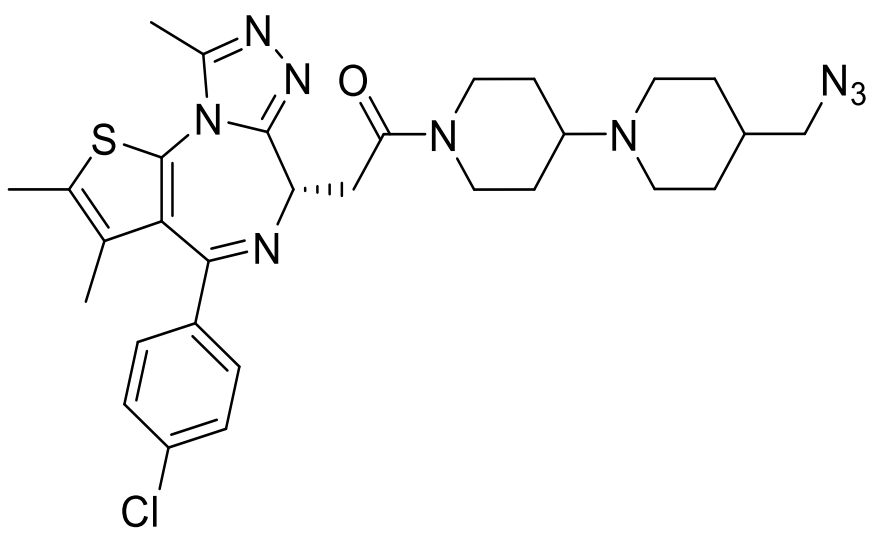

4-(azidomethyl)piperidine hydrochloride (60.0 mg; 0.329 mmol; 1.00 eq.), 1-{2-[(9*S*)-7-(4-chlorophenyl)-4,5,13-trimethyl-3-thia-1,8,11,12-tetraazatricyclo[8.3.0.0^{2,6}]trideca-2(6),4,7,10,12-pentaen-9-yl]acetyl}piperidin-4-one (175 mg; 0.362 mmol; 1.10 eq.) and *N*-Ethyldiisopropylamine (0.84 ml; 4.9 mmol; 15 eq.) were given into the vial, which was purged with argon and 1,2-dichloroethane (0.90 ml) was added. The reaction mixture was warmed up to 50°C and stirred at these temperature for 18h, therafter the reaction mixture was allowed to cool down to ambient temperature and stirred for the rest of weekend.
Next $NaBH(OAc)_3$ (218 mg; 0.987 mmol; 3.00 eq. -> repeated after several hours) was added and the reaction mixture stirred again for 48h at room temperature.
To the reaction mixture was added demineralized water and extraction carried out three times with EtOAc till there was no remaining product in water layer. Thereafter the organic layers were combined and dried via phase separator tube and concentrated under reduced pressure. Next the crude product was purified with RP-column chromatography, the clean fractions were combined and concentrated under reduced pressure to furnish 1-[4-(azidomethyl)-[1,4'-bipiperidin]-1'-yl]-2-[(9S)-7-(4-chlorophenyl)-4,5,13-trimethyl-3-thia-1,8,11,12-tetraazatricyclo[8.3.0.0^{2,6}]trideca-2(6),4,7,10,12-pentaen-9-yl]ethan-1-one (24 mg;

0.039 mmol; 12%).

Yield: 23.7 mg (0.04 mmol, 12%).
Purity: 99 % (HPLC, UV-absorption 254 nm)
Physical State: pale yellow solid.
LC-MS (ESI, m/z) calcd. for $C_{30}H_{37}ClN_9OS$ [M+H+]:606.3, found: 606.3.
^{1}H NMR (500 MHz, DMSO) δ 7.50 – 7.47 (m, 2H), 7.46 – 7.41 (m, 2H), 4.56 (td, *J* = 6.5, 1.5 Hz, 1H), 4.41 – 4.35 (m, *J* = 12.9 Hz, 1H), 4.19 – 4.13 (d, *J* = 13.6 Hz, 1H), 3.64 – 3.56 (m, 1H), 3.42 – 3.33 (m, 1H), 3.24 (dd, *J* = 6.7, 3.6 Hz, 2H), 3.14 – 3.06 (m, 1H), 2.89 – 2.83 (m, 1H), 2.59 (s, 3H), 2.42 (s, 3H), 2.16 – 2.08 (m, 2H), 1.84 – 1.68 (m, 2H), 1.67 – 1.61 (m, 5H), 1.57 – 1.43 (m, 2H), 1.30 – 1.11 (m, 4H).

1-{4-azido-[1,4'-bipiperidin]-1'-yl}-2-[(9*S*)-7-(4-chlorophenyl)-4,5,13-trimethyl-3-thia-1,8,11,12-tetraazatricyclo[8.3.0.0^{2,6}]trideca-2(6),4,7,10,12-pentaen-9-yl]ethan-1-one **a9**

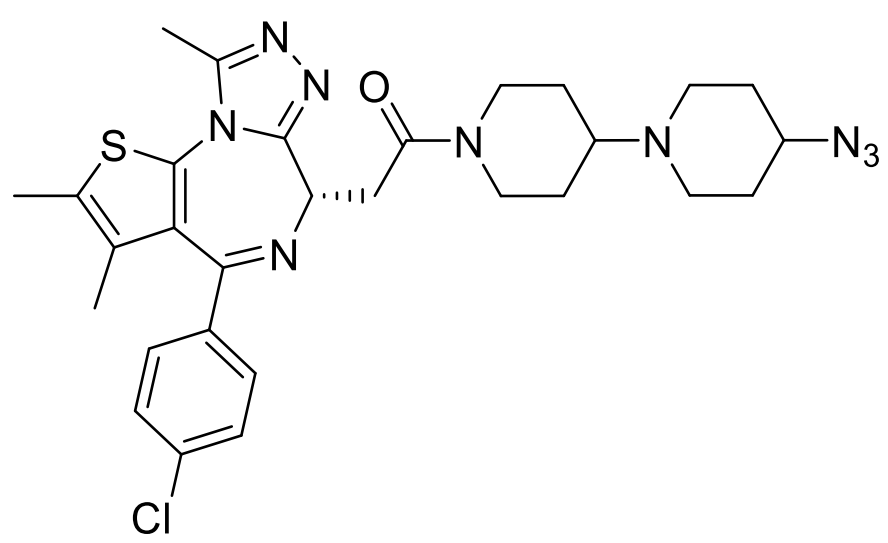

To a solution of 1-{2-[(9*S*)-7-(4-chlorophenyl)-4,5,13-trimethyl-3-thia-1,8,11,12-tetraazatricyclo[8.3.0.0^{2,6}]trideca-2(6),4,7,10,12-pentaen-9-yl]acetyl}piperidin-4-one (200 mg; 415 µmol; 1.00 eq.) and *N*Ethyldiisopropylamine for synthesis (353 µl; 2.07 mmol; 5.00 eq.) in 1,2-Dichloroethane (5.00 ml) was added 4-azidopiperidine hydrochloride (142 mg; 830 µmol; 2.00 eq.) and the reaction mixture was stirred for 1 h at 80°C. Sodium triacetoxyborohydride (177 mg; 833 µmol; 3.00 eq.) was added and it was stirred for 1 h room temperature.

The reaction mixture was evapurated on celite and NP-column chromatography furnished 1-{4-azido-[1,4'-bipiperidin]-1'-yl}-2-[(9*S*)-7-(4-chlorophenyl)-4,5,13-trimethyl-3-thia-1,8,11,12-tetraazatricyclo[8.3.0.0^{2,6}]trideca-2(6),4,7,10,12-pentaen-9-yl]ethan-1-one (174 mg; 297 µmol) as a pale yellow foam.

Yield: 174 mg (0.294 mmol, 71%).
Purity: 100 % (HPLC, UV-absorption 254 nm)

Physical State: pale yellow foam.

LC-MS (ESI, m/z) calcd. for $C_{29}H_{35}ClN_9OS$ [M+H+]:592.2, found: 592.2.

^{1}H NMR (500 MHz, DMSO) δ 7.50 – 7.47 (m, 2H), 7.46 – 7.42 (m, 2H), 4.57 (t, *J* = 6.7 Hz, 1H), 3.86 (tt, *J* = 8.8, 3.9 Hz, 1H), 3.68 – 3.57 (m, 1H), 3.23 – 3.18 (m, 2H), 2.97 (ddd, *J* = 13.1, 9.8, 3.5 Hz, 2H), 2.60 (s, 3H), 2.42 (s, 3H), 2.02 (m, 2H), 1.71 – 1.64 (m, 2H), 1.63 (s, 3H), 1.30 – 1.22 (m, 6H).

(*1S,5S,6R*)-5-(1-((1'-(2-((*S*)-4-(4-chlorophenyl)-2,3,9-trimethyl-6H-thieno[3,2-f][1,2,4]triazolo[4,3-a][1,4]diazepin-6-yl)acetyl)-[1,4'-bipiperidin]-4-yl)methyl)-1H-1,2,3-triazol-4-yl)-10-((3,5-dichlorophenyl)sulfonyl)-3-(pyridin-2-ylmethyl)-3,10-diazabicyclo[4.3.1]decan-2-one **a10c**

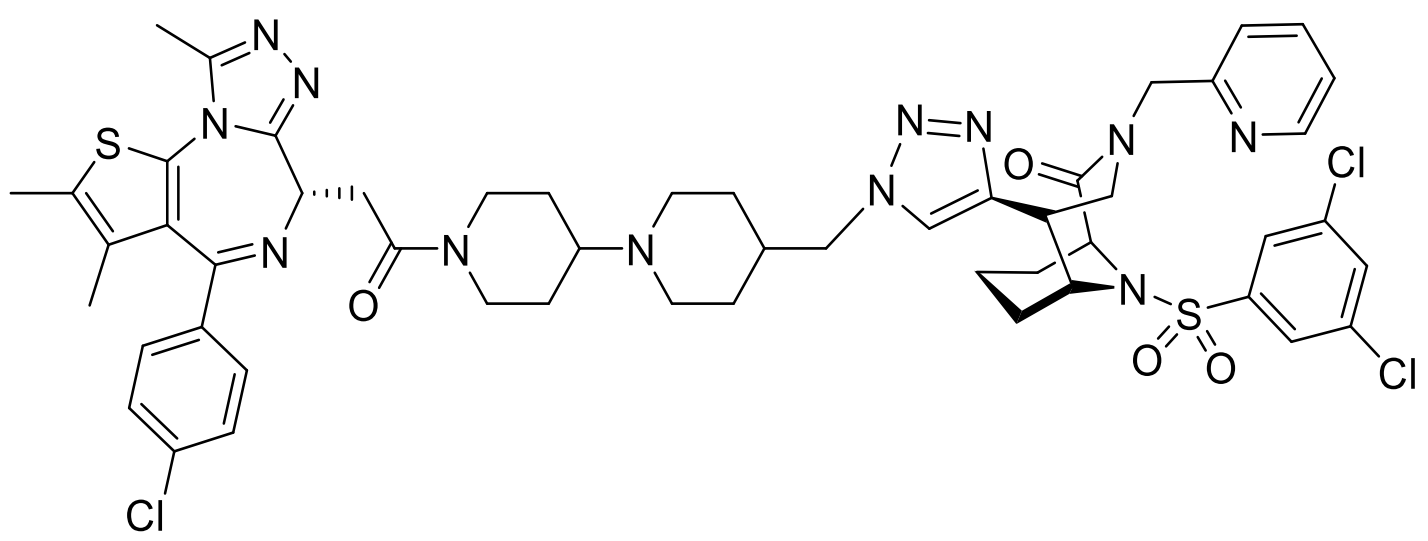

According to **GP-2**, the title compound **a10c** was synthesized from **a10** (6.3 mg, 10 µmol, 1.00 eq.) and (1S,5S,6R)-10-((3,5-dichlorophenyl)sulfonyl)- 5-ethynyl -3-(pyridin-2-ylmethyl)-3,10-diazabicyclo[4.3.1]decan-2-one [20] (4.8 mg, 10 µmol, 1.00 eq.). The obtained product was further purified by prep. HPLC (5-100 % B).

Yield: 9.2 mg (9.0 µmol, 90 %).

Purity: 98 % (HPLC, UV-absorption 220 nm)

Physical State: yellow solid.

HRMS (ESI, m/z) calcd. for $C_{52}H_{58}Cl_3N_{12}O_4S_2$ [M+H$^+$]:1083.32055, found: 1083.32267.

(*1S,5S,6R*)-5-(((1-((1'-(2-((*S*)-4-(4-chlorophenyl)-2,3,9-trimethyl-6H-thieno[3,2-f][1,2,4]triazolo[4,3-a][1,4]diazepin-6-yl)acetyl)-[1,4'-bipiperidin]-4-yl)methyl)-1H-1,2,3-triazol-4-yl)methoxy)methyl)-10-((3,5-dichlorophenyl)sulfonyl)-3-(pyridin-2-ylmethyl)-3,10-diazabicyclo[4.3.1]decan-2-one **a9d**

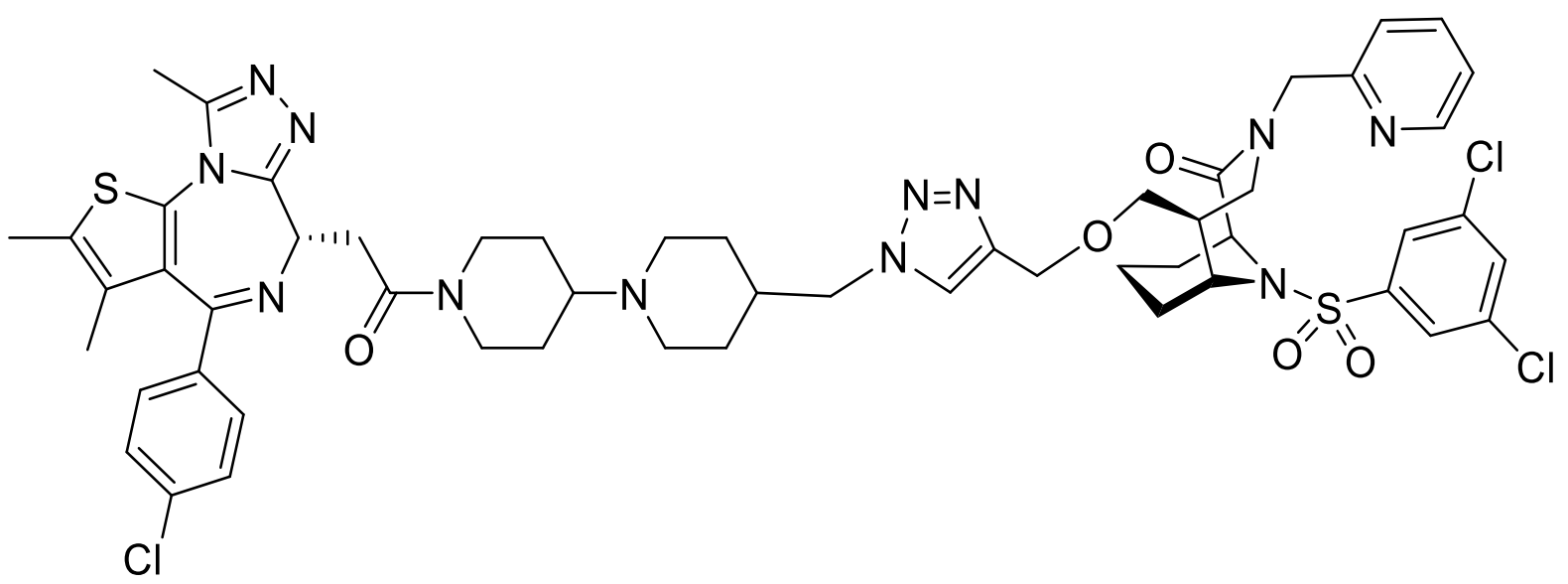

According to **GP-2**, the title compound **a9d** was synthesized from **a9** (6.1 mg, 10 µmol, 1.00 eq.) and (1S,5S,6R)-10-((3,5-dichlorophenyl)sulfonyl)-5-((prop-2-yn-1-yloxy)methyl)-3-(pyridin-2-ylmethyl)-3,10-diazabicyclo[4.3.1]decan-2-one [20] (5.2 mg, 10 µmol, 1.00 eq.). The obtained product was further purified by prep. HPLC (5-100 % B).

Yield: 8 mg (7.0 µmol, 71%).

Purity: 97 % (HPLC, UV-absorption 220 nm)

Physical State: yellow solid.

HRMS (ESI, m/z) calcd. for $C_{54}H_{62}Cl_3N_{12}O_5S_2$ [M+H$^+$]:1127.34676, found: 1127.34866.

(*1S,5S,6R*)-5-(1-(1'-(2-((*S*)-4-(4-chlorophenyl)-2,3,9-trimethyl-6H-thieno[3,2-f][1,2,4]triazolo[4,3-a][1,4]diazepin-6-yl)acetyl)-[1,4'-bipiperidin]-4-yl)-1H-1,2,3-triazol-4-yl)-10-((3,5-dichlorophenyl)sulfonyl)-3-(pyridin-2-ylmethyl)-3,10-diazabicyclo[4.3.1]decan-2-one **a9c**

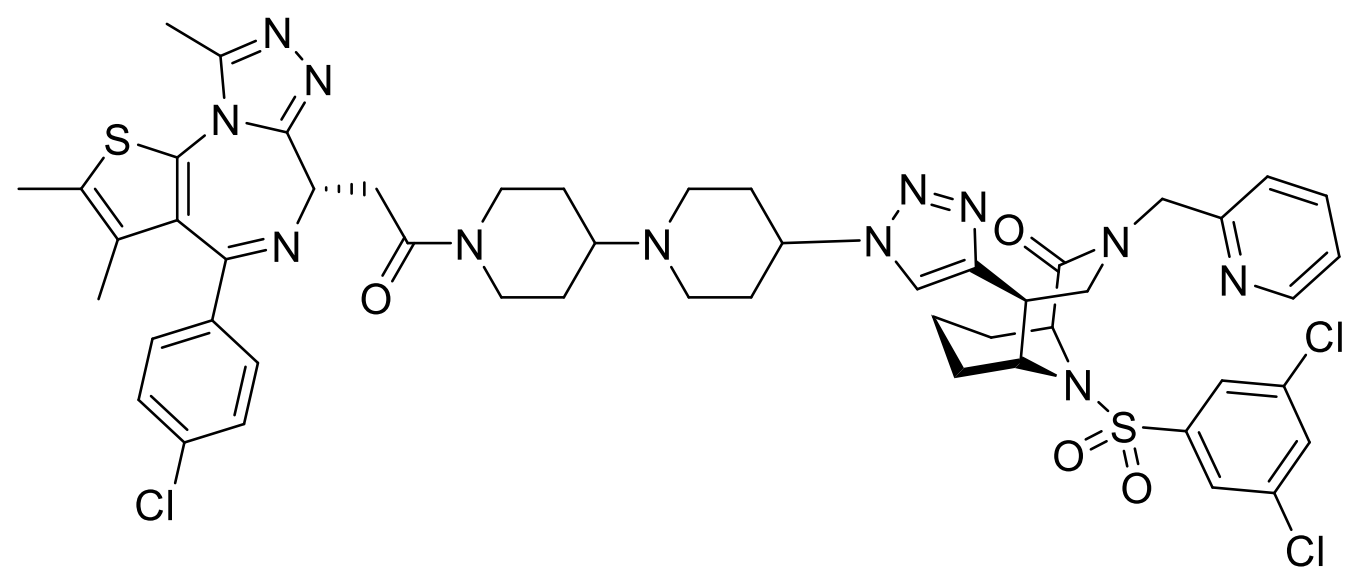

According to **GP-2**, the title compound **a9c** was synthesized from **a9** (5.9 mg, 10 µmol, 1.00 eq.) and (1S,5S,6R)-10-((3,5-dichlorophenyl)sulfonyl)- 5-ethynyl -3-(pyridin-2-ylmethyl)-3,10-diazabicyclo[4.3.1]decan-2-one [20] (4.8 mg, 10 µmol, 1.00 eq.). The obtained product was further purified by prep. HPLC (5-100 % B).

Yield: 6.7 mg (7.0 µmol, 70%).

Purity: 98 % (HPLC, UV-absorption 220 nm)

Physical State: yellow solid.

HRMS (ESI, m/z) calcd. for $C_{51}H_{56}Cl_3N_{12}O_4S_2$ [M+H$^+$]:1069.30490, found: 1069.30667.

(*1S,5S,6R*)-5-(((1-(1'-(2-((*S*)-4-(4-chlorophenyl)-2,3,9-trimethyl-6H-thieno[3,2-

f][1,2,4]triazolo[4,3-a][1,4]diazepin-6-yl)acetyl)-[1,4'-bipiperidin]-4-yl)-1H-1,2,3-triazol-4-yl)methoxy)methyl)-10-((3,5-dichlorophenyl)sulfonyl)-3-(pyridin-2-ylmethyl)-3,10-diazabicyclo[4.3.1]decan-2-one **a9d**

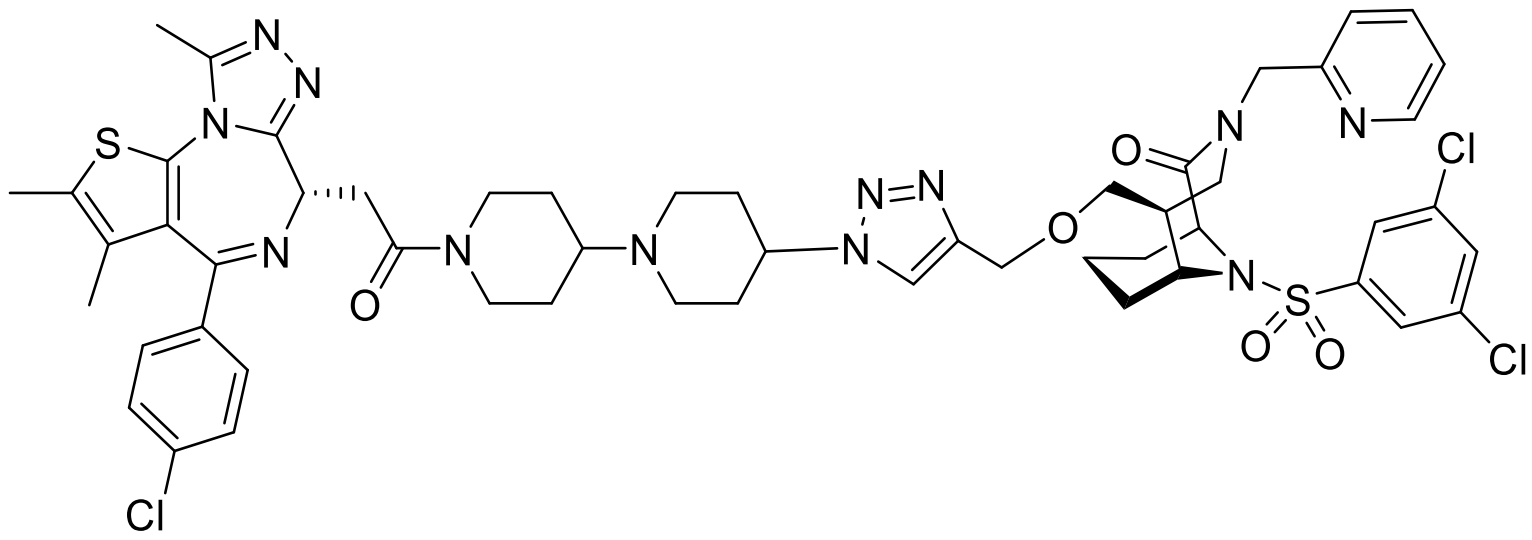

According to **GP-2**, the title compound **a9d** was synthesized from **a9** (12 mg, 20 µmol, 1.00 eq.) and (1S,5S,6R)-10-((3,5-dichlorophenyl)sulfonyl)-5-((prop-2-yn-1-yloxy)methyl)-3-(pyridin-2-ylmethyl)-3,10-diazabicyclo[4.3.1]decan-2-one [20] (11 mg, 20 µmol, 1.00 eq.). The obtained product was further purified by prep. HPLC (5-100 % B).

Yield: 11 mg (10 µmol, 50%).

Purity: 92 % (HPLC, UV-absorption 220 nm)

Physical State: yellow solid.

HRMS (ESI, m/z) calcd. for $C_{53}H_{60}Cl_3N_{12}O_5S_2$ [M+H$^+$]:1113.33111, found: 1113.33254.

2-[(9*S*)-7-(4-chlorophenyl)-4,5,13-trimethyl-3-thia-1,8,11,12-tetraazatricyclo[8.3.0.0^{2,6}]trideca-2(6),4,7,10,12-pentaen-9-yl]-1-[3-(hydroxymethyl)azetidin-1-yl]ethan-1-one

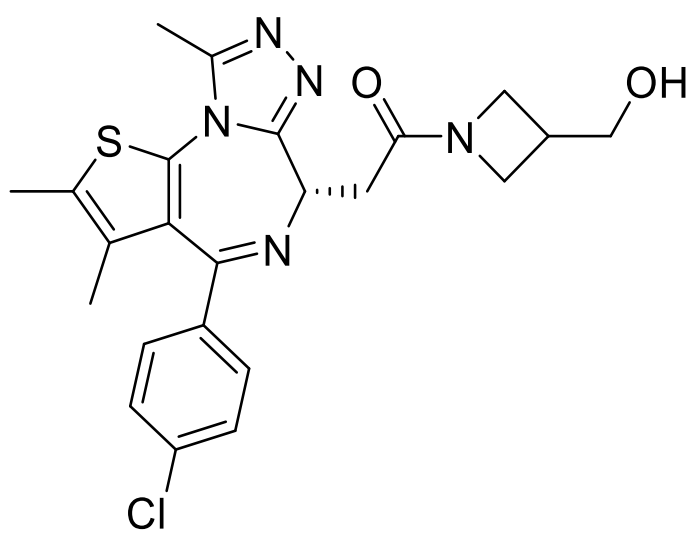

To a solution of 2-[(9*S*)-7-(4-chlorophenyl)-4,5,13-trimethyl-3-thia-1,8,11,12-tetraazatricyclo[8.3.0.0²,6]trideca-2(6),4,7,10,12-pentaen-9-yl]acetic acid (450 mg; 1.07 mmol; 1.00 eq.) and *N*-ethyldiisopropylamine (464 µl; 2.67 mmol; 2.50 eq.) in *N,N*-Dimethylformamide (5.00 ml) was added HATU (468 mg; 1.23 mmol; 1.15 eq.) and the mixture was stirred for 30 min at room temperature. azetidin-3-ylmethanol hydrochloride (153 mg; 1.18 mmol; 1.1 eq.) was added and it was stirred over night.

EtOAc (100 mL) and NaOH-solution (100 mL; 1 M aq.) were added, the phases were separated and the water phase was washed with EtOAc (2 x 40 mL). the combined organic phases were

dried over Na_2SO_4 and all volatile ingredients were removed under reduced pressure to furnish crude product 2-[(9*S*)-7-(4-chlorophenyl)-4,5,13-trimethyl-3-thia-1,8,11,12-tetraazatricyclo[8.3.0.0^{2,6}]trideca-2(6),4,7,10,12-pentaen-9-yl]-1-[3-(hydroxymethyl)azetidin-1-yl]ethan-1-one (570 mg; 1.08 mmol) as a pale yellow solid.

Yield: 570 mg (1.80 mmol, 100%).
Purity: 89 % (HPLC, UV-absorption 254 nm)
Physical State: pale yellow solid.
LC-MS (ESI, m/z) calcd. for $C_{23}H_{25}ClN_5O_2S$ [M+H+]:470.1, found: 470.1.

1-{2-[(9*S*)-7-(4-chlorophenyl)-4,5,13-trimethyl-3-thia-1,8,11,12-tetraazatricyclo[8.3.0.0^{2,6}]trideca-2(6),4,7,10,12-pentaen-9-yl]acetyl}azetidine-3-carbaldehyde

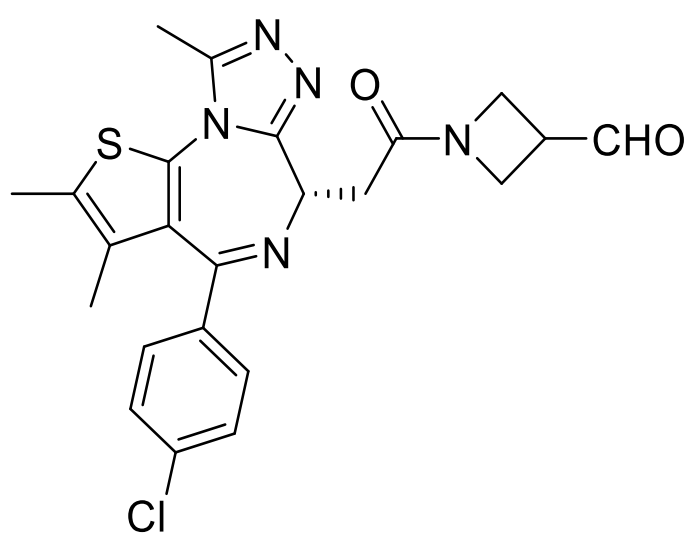

To a suspension of 2-[(9*S*)-7-(4-chlorophenyl)-4,5,13-trimethyl-3-thia-1,8,11,12-tetraazatricyclo[8.3.0.0^{2,6}]trideca-2(6),4,7,10,12-pentaen-9-yl]-1-[3-(hydroxymethyl)azetidin-1-yl]ethan-1-one (570 mg; 1.09 mmol; 1.00 eq.) and sodium hydrogen carbonate (367 mg; 4.37 mmol; 4.00 eq.) in D (5.00 ml) was added Dess-Martin periodinane (509 mg; 1.20 mol; 1.10 eq.) and the reaction mixture was stirred for 2 h at room temperature.
Water (10 ml) and $Na_2S_2O_3$-solution (aq) was added and the mixture was stirred for 30 min. The phases were separated and the water phase was washed with EtOAc (2 x 20 ml). The combined organic phases were dried over Na_2SO_4 and all volatile ingredients were removed under reduced pressure. NP-column chromatography furnished 1-{2-[(9*S*)-7-(4-chlorophenyl)-4,5,13-trimethyl-3-thia-1,8,11,12-tetraazatricyclo[8.3.0.0^{2,6}]trideca-2(6),4,7,10,12-pentaen-9-yl]acetyl}azetidine-3-carbaldehyde (268 mg; 573 mmol) as colourless oil.

Yield: 268 mg (0.573 mmol, 53%).
Purity: 100 % (HPLC, UV-absorption 254 nm)

Physical State: colourless oil.

LC-MS (ESI, m/z) calcd. for $C_{23}H_{23}ClN_5O_2S$ [M+H+]:468.1, found: 468.1.

1-{3-[(4-azidopiperidin-1-yl)methyl]azetidin-1-yl}-2-[(9*S*)-7-(4-chlorophenyl)-4,5,13-trimethyl-3-thia-1,8,11,12-tetraazatricyclo[8.3.0.0^{2,6}]trideca-2(6),4,7,10,12-pentaen-9-yl]ethan-1-one **a11**

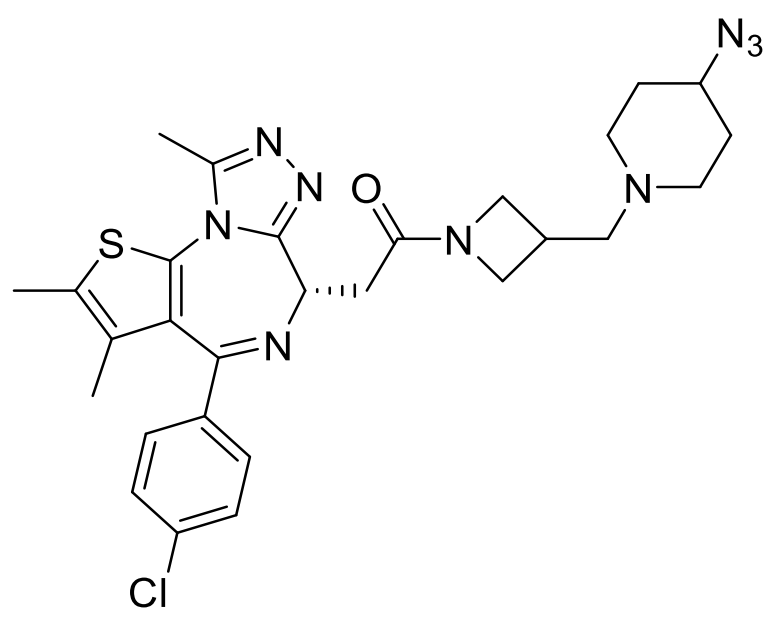

To a solution of 1-{2-[(9*S*)-7-(4-chlorophenyl)-4,5,13-trimethyl-3-thia-1,8,11,12-tetraazatricyclo[8.3.0.0^{2,6}]trideca-2(6),4,7,10,12-pentaen-9-yl]acetyl}azetidine-3-carbaldehyde (110 mg; 235 µmol; 1.00 eq.) and N-ethyldiisopropylamine (200 µl; 1.18 mmol; 5.00 eq.) in DCM (1 ml) was added 4azidopiperidine hydrochloride (80.4 mg; 470 µmol; 2.00 eq.) and the reaction mixture was stirred for 1 h at room temperature. Sodium triacetoxyborohydride (149 mg; 705 mmol; 3.00 eq.) was added and it was stirred 1 h.

The reaction mixture was evapurated on celite and NP-column chromatography furnished 1-{3-[(4-azidopiperidin-1-yl)methyl]azetidin-1-yl}-2-[(9S)-7-(4-chlorophenyl)-4,5,13-trimethyl-3-thia-1,8,11,12-tetraazatricyclo[8.3.0.0^{2,6}]trideca-2(6),4,7,10,12-pentaen-9-yl]ethan-1-one (150 mg; 252 µmol) as a colourless oil.

Yield: 150 mg (0.252 mmol, 107%).

Purity: 97 % (HPLC, UV-absorption 254 nm)

Physical State: colourless oil.

LC-MS (ESI, m/z) calcd. for $C_{28}H_{33}ClN_9OS$ [M+H+]:578.2, found: 578.3.

^{1}H NMR (500 MHz, DMSO) δ 7.50 (dd, *J* = 8.7, 2.8 Hz, 2H), 7.44 (dd, *J* = 8.6, 1.7 Hz, 2H), 4.51 – 4.34 (m, 2H), 4.06 – 3.90 (m, 2H), 3.63 – 3.45 (m, 1H), 3.28-3.20 (m, 1H), 3.13-3.06 (m, 1H), 2.82 (b, 1H), 2.71 (b, 2H), 2.60 (s, 3H), 2.41 (s, 3H), 1.91 (s, 1H), 1.85 (b, 2H), 1.63 (s, 3H), 1.52 (b, 2H), 1.24 (b, 4H).

1-(3-{[4-(azidomethyl)piperidin-1-yl]methyl}azetidin-1-yl)-2-[(9*S*)-7-(4-chlorophenyl)-4,5,13-trimethyl-3-thia-1,8,11,12-tetraazatricyclo[8.3.0.0^{2,6}]trideca-2(6),4,7,10,12-pentaen-9-yl]ethan-1-one **a12**

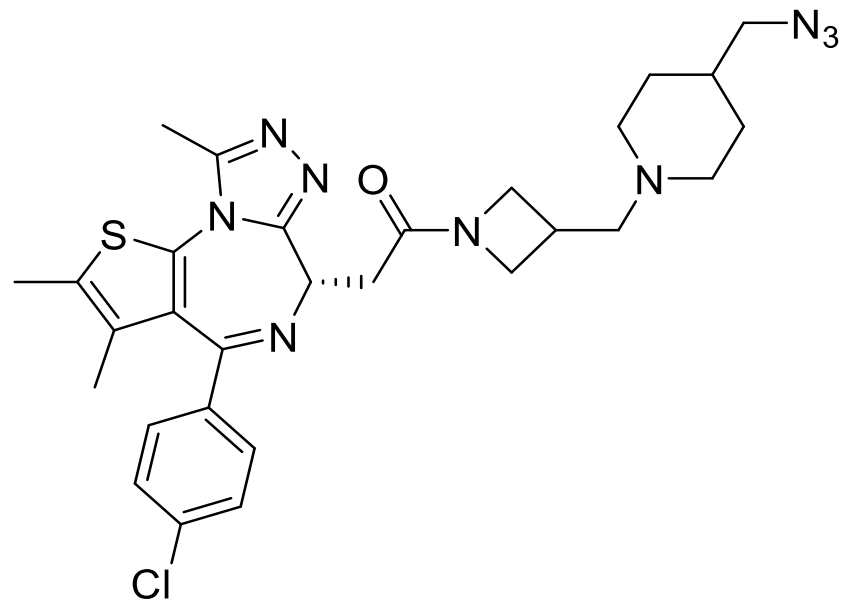

To a solution of 1-{2-[(9*S*)-7-(4-chlorophenyl)-4,5,13-trimethyl-3-thia-1,8,11,12-tetraazatricyclo[8.3.0.0^{2,6}]trideca-2(6),4,7,10,12-pentaen-9-yl]acetyl}azetidine-3-carbaldehyde (130 mg; 278 µmol; 1.00 eq.) and *N*-ethyldiisopropylamine (236 µl; 1.39 mmol; 5.00 eq.) in 1,2-dichloroethane (3.0 ml) and the reaction mixture was stirred for 1 h at room temperature. Sodium triacetoxyborohydride (177 mg; 833 µmol; 3.00 eq.) was added and it was stirred 1 h.
The reaction mixture was evapurated on celite and NP-column chromatography furnished 1-(3-{[4-(azidomethyl)piperidin-1-yl]methyl}azetidin-1-yl)-2-[(9S)-7-(4-chlorophenyl)-4,5,13-trimethyl-3-thia-1,8,11,12-tetraazatricyclo[8.3.0.0^{2,6}]trideca-2(6),4,7,10,12-pentaen-9-yl]ethan-1-one (160 mg; 270 µmol) as a colourless oil.

Yield: 160 mg (0.270 mmol, 97%).
Purity: 100 % (HPLC, UV-absorption 254 nm)
Physical State: colourless oil.
LC-MS (ESI, m/z) calcd. for $C_{29}H_{35}ClN_9OS$ [M+H+]:592.2, found: 592.2.
^{1}H NMR (500 MHz, DMSO) δ 7.50 (dd, *J* = 8.7, 3.2 Hz, 2H), 7.44 (dd, *J* = 8.6, 1.7 Hz, 2H), 4.51 – 4.36 (m, 2H), 4.09 – 3.88 (m, 2H), 3.54 (b, 2H), 3.29-3.20 (m, 3H), 3.14 – 3.06 (m, 1H), 2.89 (s, 3H), 2.62 – 2.58 (m, 3H), 2.41 (s, 3H), 1.91 (s, 1H), 1.63 (s, 3H), 1.38 – 1.10 (m, 5H).

(*1S,5S,6R*)-5-(1-((1-((1-(2-((*S*)-4-(4-chlorophenyl)-2,3,9-trimethyl-6H-thieno[3,2-f][1,2,4]triazolo[4,3-a][1,4]diazepin-6-yl)acetyl)azetidin-3-yl)methyl)piperidin-4-yl)methyl)-1H-1,2,3-triazol-4-yl)-10-((3,5-dichlorophenyl)sulfonyl)-3-(pyridin-2-ylmethyl)-3,10-

diazabicyclo[4.3.1]decan-2-one **a12c**

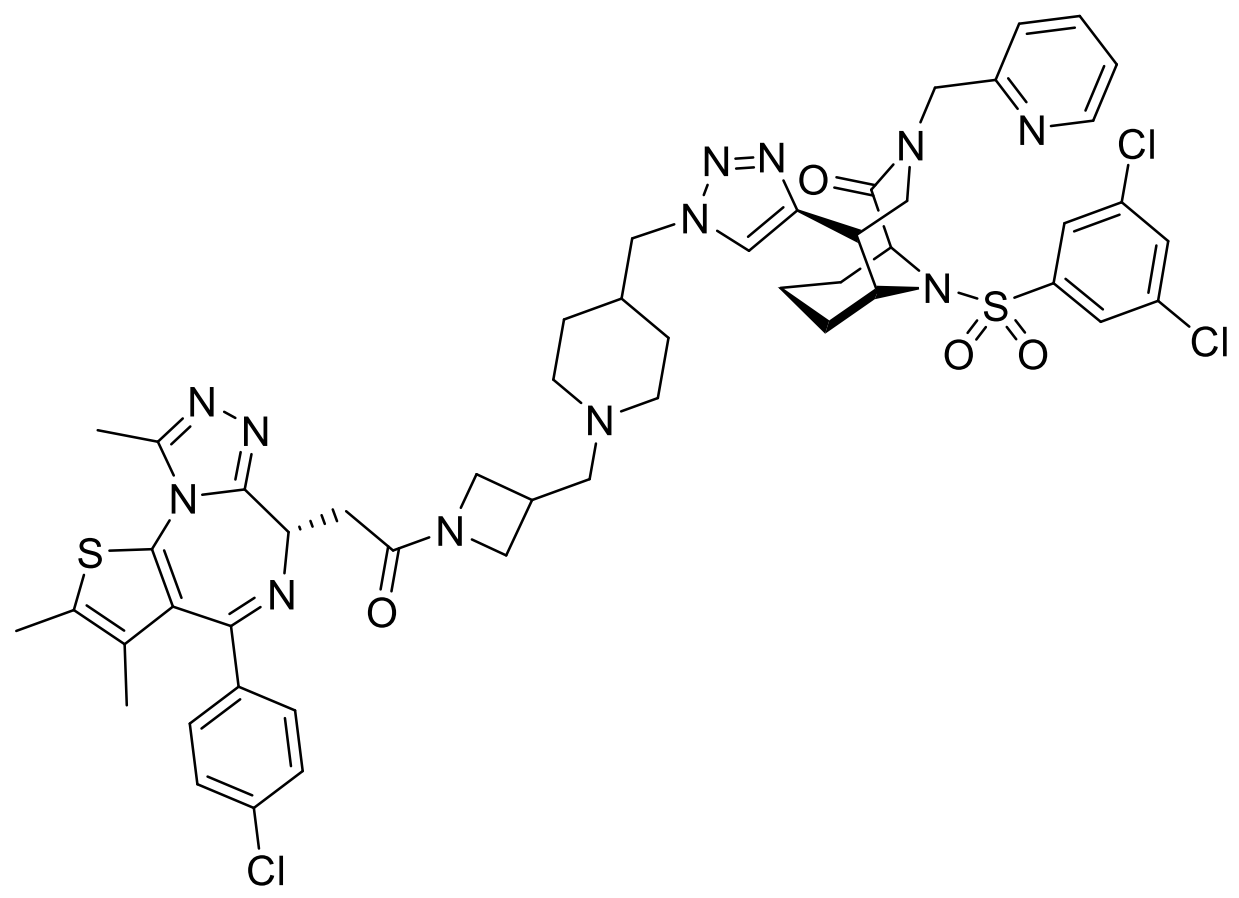

According to **GP-2**, the title compound **a12c** was synthesized from **a12** (6.8 mg, 12 µmol, 1.00 eq.) and (1S,5S,6R)-10-((3,5-dichlorophenyl)sulfonyl)- 5-ethynyl -3-(pyridin-2-ylmethyl)-3,10-diazabicyclo[4.3.1]decan-2-one [20] (5.0 mg, 12 µmol, 1.00 eq.). The obtained product was further purified by prep. HPLC (5-100 % B).

Yield: 12 mg (14 µmol, 95%).

Purity: 98 % (HPLC, UV-absorption 220 nm)

Physical State: yellow solid.

HRMS (ESI, m/z) calcd. for $C_{51}H_{56}Cl_3N_{12}O_4S_2$ [M+H$^+$]:1069.30490, found: 1069.30695.

(*1S,5S,6R*)-5-(((1-((1-((1-(2-((*S*)-4-(4-chlorophenyl)-2,3,9-trimethyl-6H-thieno[3,2-f][1,2,4]triazolo[4,3-a][1,4]diazepin-6-yl)acetyl)azetidin-3-yl)methyl)piperidin-4-yl)methyl)-1H-1,2,3-triazol-4-yl)methoxy)methyl)-10-((3,5-dichlorophenyl)sulfonyl)-3-(pyridin-2-ylmethyl)-3,10-diazabicyclo[4.3.1]decan-2-one **a12d**

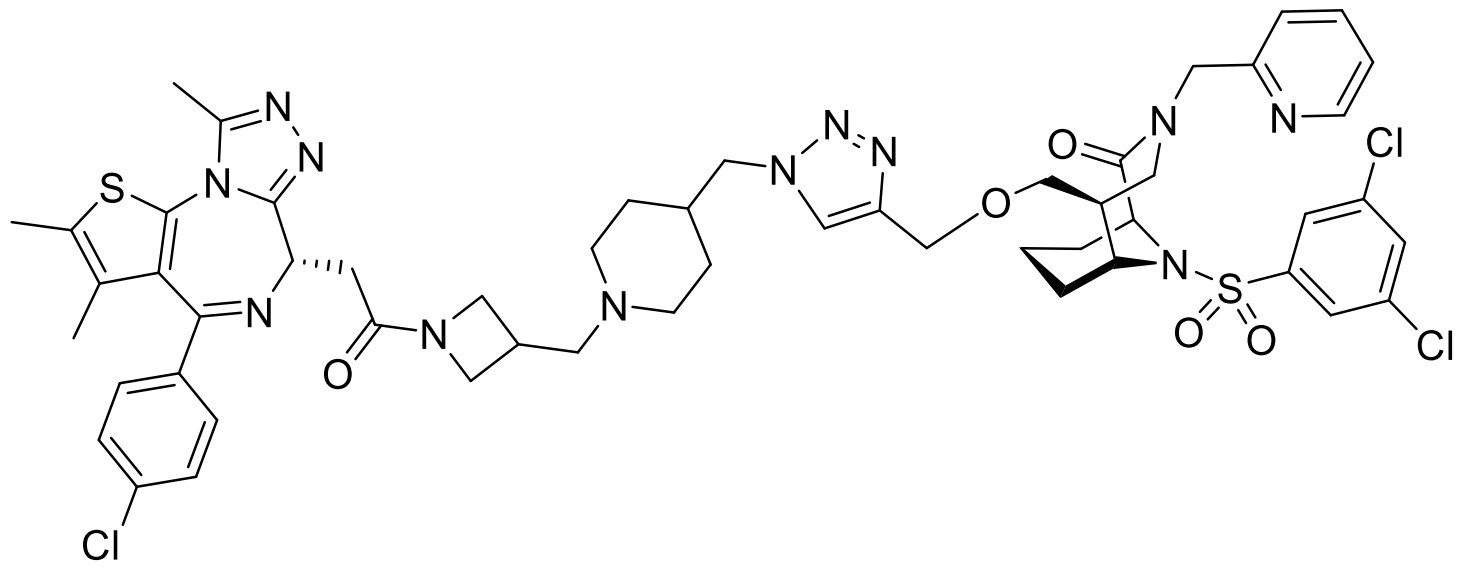

According to **GP-2**, the title compound **a12d** was synthesized from **a12** (7.1 mg, 12 µmol, 1.00 eq.) and (1S,5S,6R)-10-((3,5-dichlorophenyl)sulfonyl)-5-((prop-2-yn-1-yloxy)methyl)-3-(pyridin-2-ylmethyl)-3,10-diazabicyclo[4.3.1]decan-2-one [20] (6.2 mg, 12 µmol, 1.00 eq.). The obtained product was further purified by prep. HPLC (5-100 % B).

Yield: 11 mg (10 µmol, 84%).

Purity: 98 % (HPLC, UV-absorption 220 nm)

Physical State: yellow solid.

HRMS (ESI, m/z) calcd. for $C_{53}H_{60}Cl_3N_{12}O_5S_2$ [M+H$^+$]:1113.33111, found: 1113.33305.

(*1S,5S,6R*)-5-(1-(1-((1-(2-((*R*)-4-(4-chlorophenyl)-2,3,9-trimethyl-6H-thieno[3,2-f][1,2,4]triazolo[4,3-a]azepin-6-yl)acetyl)azetidin-3-yl)methyl)piperidin-4-yl)-1H-1,2,3-triazol-4-yl)-10-((3,5-dichlorophenyl)sulfonyl)-3-(pyridin-2-ylmethyl)-3,10-diazabicyclo[4.3.1]decan-2-one **a11c**

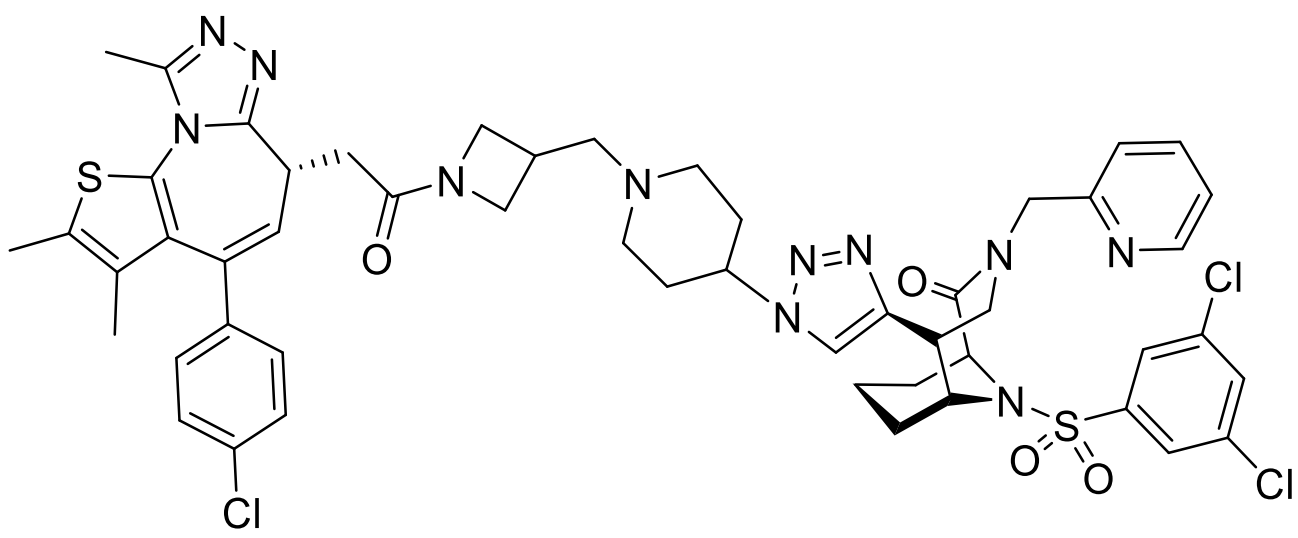

According to **GP-2**, the title compound **a11c** was synthesized from **a11** (6.3 mg, 10 µmol, 1.00 eq.) and (1S,5S,6R)-10-((3,5-dichlorophenyl)sulfonyl)- 5-ethynyl -3-(pyridin-2-ylmethyl)-3,10-diazabicyclo[4.3.1]decan-2-one [20] (5.0 mg, 10 µmol, 1.00 eq.). The obtained product was further purified by prep. HPLC (5-100 % B).

Yield: 7 mg (7.0 µmol, 70%).

Purity: 99 % (HPLC, UV-absorption 220 nm)

Physical State: yellow solid.

HRMS (ESI, m/z) calcd. for $C_{50}H_{54}Cl_3N_{12}O_4S_2$ [M+H$^+$]:1055.28925, found: 1055.29127.

(*1S,5S,6R*)-5-(((1-(1-((1-(2-((*S*)-4-(4-chlorophenyl)-2,3,9-trimethyl-6H-thieno[3,2-f][1,2,4]triazolo[4,3-a][1,4]diazepin-6-yl)acetyl)azetidin-3-yl)methyl)piperidin-4-yl)-1H-1,2,3-triazol-4-yl)methoxy)methyl)-10-((3,5-dichlorophenyl)sulfonyl)-3-(pyridin-2-ylmethyl)-3,10-diazabicyclo[4.3.1]decan-2-one **a11d**

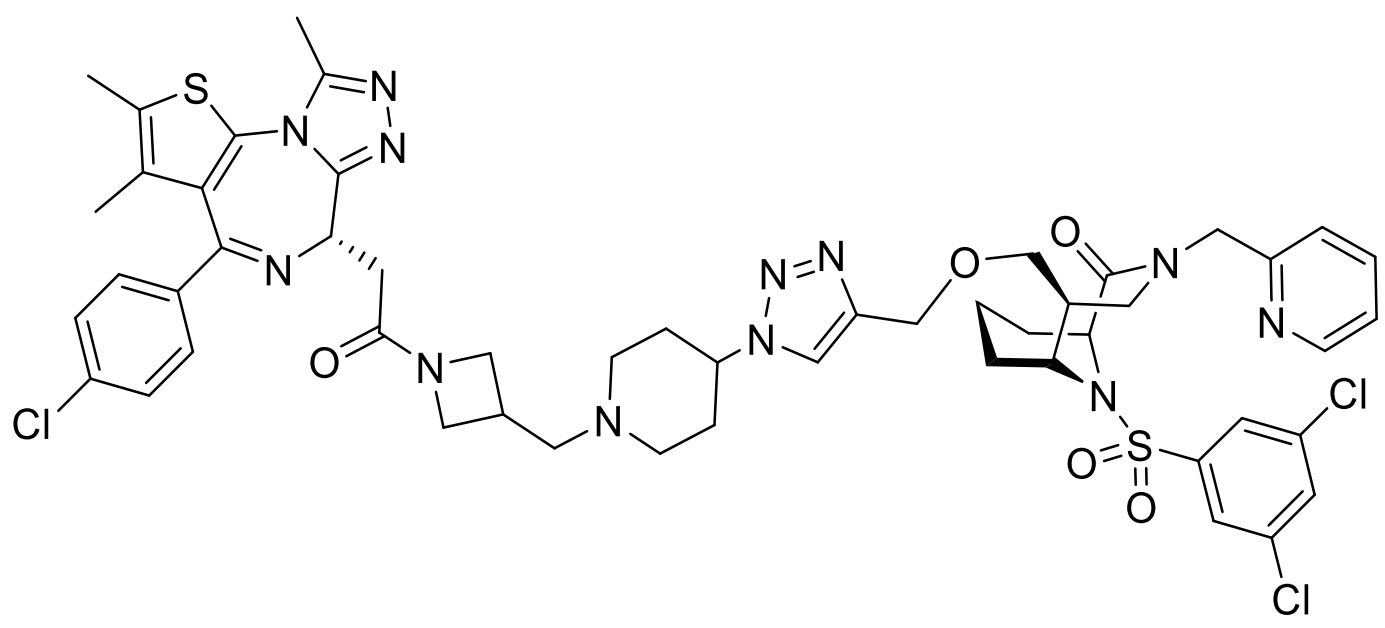

According to **GP-2**, the title compound **a11d** was synthesized from **a11** (6.1 mg, 10 µmol, 1.00 eq.) and (1S,5S,6R)-10-((3,5-dichlorophenyl)sulfonyl)-5-((prop-2-yn-1-yloxy)methyl)-3-(pyridin-2-ylmethyl)-3,10-diazabicyclo[4.3.1]decan-2-one [20] (5.2 mg, 10 µmol, 1.00 eq.). The obtained product was further purified by prep. HPLC (5-100 % B).

Yield: 7.4 mg (6.7 µmol, 67 %).

Purity: 98 % (HPLC, UV-absorption 220 nm)

Physical State: yellow solid.

HRMS (ESI, m/z) calcd. for $C_{52}H_{58}Cl_3N_{12}O_5S_2$ [M+H$^+$]:1099.31546, found: 1099.31775.

4. References

1. Moran, U., Phillips, R. & Milo, R. SnapShot: Key numbers in biology. *Cell* **141**, 1–2 (2010).
2. Gerlach, M., Mueller, U. & Weiss, M. S. The MX beamlines BL14.1-3 at BESSY II. *J. large-scale Res. Facil. JLSRF* **2**, (2016).
3. Gabadinho, J. *et al.* MxCuBE: A synchrotron beamline control environment customized for macromolecular crystallography experiments. *J. Synchrotron Radiat.* **17**, 700–707 (2010).
4. The CCP4 suite: Programs for protein crystallography. *Acta Crystallogr. Sect. D Biol. Crystallogr.* **50**, 760–763 (1994).
5. Potterton, E., Briggs, P., Turkenburg, M. & Dodson, E. A graphical user interface to the CCP4 program suite. *Acta Crystallogr. - Sect. D Biol. Crystallogr.* **59**, 1131–1137 (2003).
6. Winn, M. D. *et al.* Overview of the CCP4 suite and current developments. *Acta Crystallographica Section D: Biological Crystallography* vol. 67 235–242 at https://doi.org/10.1107/S0907444910045749 (2011).
7. Potterton, L. *et al.* CCP 4 i 2: The new graphical user interface to the CCP 4 program suite. *Acta Crystallogr. Sect. D Struct. Biol.* **74**, 68–84 (2018).
8. Winter, G. *et al.* DIALS: Implementation and evaluation of a new integration package. *Acta Crystallogr. Sect. D Struct. Biol.* **74**, 85–97 (2018).
9. Evans, P. R. An introduction to data reduction: Space-group determination, scaling and intensity statistics. *Acta Crystallogr. Sect. D Biol. Crystallogr.* **67**, (2011).
10. Evans, P. R. & Murshudov, G. N. How good are my data and what is the resolution? *Acta Crystallogr. Sect. D Biol. Crystallogr.* **69**, 1204–1214 (2013).
11. McCoy, A. J. *et al.* Phaser crystallographic software. *J. Appl. Crystallogr.* **40**, 658–674 (2007).
12. Vagin, A. A. *et al.* REFMAC5 dictionary: Organization of prior chemical knowledge and guidelines for its use. *Acta Crystallogr. Sect. D Biol. Crystallogr.* **60**, 2184–2195 (2004).

13. Emsley, P., Lohkamp, B., Scott, W. G. & Cowtan, K. Features and development of Coot. *Acta Crystallogr. Sect. D Biol. Crystallogr.* **66**, 486–501 (2010).
14. Murshudov, G. N. *et al.* REFMAC5 for the refinement of macromolecular crystal

structures. *Acta Crystallogr. Sect. D Biol. Crystallogr.* **67**, 355–367 (2011).

15. Nicholls, R. A., Long, F. & Murshudov, G. N. Low-resolution refinement tools in REFMAC5. *Acta Crystallogr. Sect. D Biol. Crystallogr.* **68**, 404–417 (2012).

16. Murshudov, G. N., Vagin, A. A. & Dodson, E. J. Refinement of macromolecular structures by the maximum-likelihood method. *Acta Crystallographica Section D: Biological Crystallography* vol. 53 240–255 at https://doi.org/10.1107/S0907444996012255 (1997).

17. Winn, M. D., Murshudov, G. N. & Papiz, M. Z. Macromolecular TLS Refinement in REFMAC at Moderate Resolutions. *Methods Enzymol.* **374**, 300–321 (2003).

18. Long, F. *et al.* AceDRG: A stereochemical description generator for ligands. *Acta Crystallogr. Sect. D Struct. Biol.* **73**, 112–122 (2017).

19. Shergalis, A. G. *et al.* CRISPR Screen Reveals BRD2/4 Molecular Glue-like Degrader via Recruitment of DCAF16. *ACS Chem. Biol.* **18**, 331–339 (2022).

20. Geiger, T. M. *et al.* Discovery of a Potent Proteolysis Targeting Chimera Enables Targeting the Scaffolding Functions of FK506-Binding Protein 51 (FKBP51). *Angew. Chemie - Int. Ed.* **63**, (2024).

21. Kolos, J. M. *et al.* Picomolar FKBP inhibitors enabled by a single water-displacing methyl group in bicyclic [4.3.1] aza-amides. *Chem. Sci.* **12**, 14758–14765 (2021).

22. Deutscher, R. C. E. *et al.* [4.3.1]Bicyclic FKBP Ligands Inhibit Legionella Pneumophila Infection by LpMip-Dependent and LpMip-Independent Mechanisms. *ChemBioChem* **24**, 1–12 (2023).
