# Supplementary material for: Cell type–selective targeting by heterobifunctional protein binders via in-cell enrichment
Source: Nat Commun. 2026 Sep 17;17:9929. doi: 10.1038/s41467-026-77460-w (PMC13586308; doi:10.1038/s41467-026-77460-w)
Supplement: Supplementary file 3 [file 41467_2026_77460_MOESM3_ESM.pdf]

nature portfolio

Peer Review File

Cell Type–Selective Targeting by Heterobifunctional Protein Binders via In-cell Enrichment

Corresponding Author: Professor Felix Hausch

This file contains all reviewer reports in order by version, followed by all author rebuttals in order by version.

Version 0:

Reviewer comments:

Reviewer #1

(Remarks to the Author)
This study explores the functional effects of heterobifunctional molecules. The authors designed a number of compounds combining BRD4 inhibitor and FKBP12 ligands with various linkers, which were analyzed in depth by biochemical approaches including crystal structures. The key biological consequences triggered by these bifunctionals is a "CellTrap" effect: high cellular FKBP12 levels act like a trap, pulling more drugs into the cell and boosting its concentration and BRD4 inhibition. Th trap effect makes the molecules work at very low doses and allows them to distinguish between similar BRD4 domains. The study includes a model showing how FKBP12 abundance and drug affinity affect cell killing, proving that using common proteins as "presenters" can improve drug selectivity. This offers a new way to design drugs that target specific cell types with fewer side effects. However, the mathematical model proposed should be described in greater detail to allow for proper evaluation of its assumptions. Moreover, the experimental data do not fully support the proposed model, particularly the suggested correlation between FKBP12 abundance and compound efficacy. Additional experimental validation and quantitative analysis are needed to substantiate the model's biological relevance. Therefore, although the study reveals interesting findings, the molecular insights remain limited.

Major concerns:
1 ．The authors propose that FKBP12 binding enriches bifunctional compounds intracellularly, enhancing their efficacy compared to JQ1. However, this conjecture requires stronger support. Specifically, direct measurements of intracellular compound concentrations are needed to confirm increased accumulation, which is dependent on FKBP12 abundance.
2. Although the bifunctional compounds exhibit higher affinity for FKBP12 than for BRD4, the authors should examine whether FKBP12 is present at a significantly higher abundance than BRD4. This point is critical because the proposed mechanism—whereby FKBP12 acts as a “cellular trap” that enriches bifunctional compounds upon cell entry—relies on the assumption that FKBP12 is in large excess over BRD4. If the two proteins are present at similar levels, the compound may instead form binary complexes with BRD4 directly, and the contribution of FKBP12 to compound enrichment would be diminished.
3. The study's model assumes an "undepletable extracellular drug reservoir" (i.e., constant [L]extracellular), but the authors also report sub-nanomolar IC50 values for bifunctionals. This seems paradoxical because: low effective concentrations imply limited drug availability: If the model requires excess drug to drive intracellular accumulation, how do bifunctionals work at picomolar levels?
4. The model assumes that bifunctional compounds enter cells via unrestricted free exchange. However, recent studie (Wang Z, et al, Cell, 2025, 14:S0092-8674(25)00386-1.) shows that PROTACs enter cells via CD36-mediated endocytosis rather than free diffusion. Given the similar size and physicochemical properties between PROTACs and bifunctionals, it is likely that the compounds in this study also rely on endocytic uptake. The authors should experimentally verify whether their bifunctional molecules enter cells through endocytosis, as this would fundamentally challenge the core assumption of free diffusion in their proposed model.

Minor points:
1. In Figures 5 and S8, the authors used an FKBP12-targeting PROTAC to reduce FKBP12 protein levels in order to illustrate the importance of FKBP12 abundance for the activity of these heterobifunctional molecules. The authors should treat cells with different concentrations of the FKBP12-targeting PROTAC to examine how varying FKBP12 abundance affects the activity of the heterobifunctional molecules. Moreover, it would be important to test whether these molecules remain effective in FKBP12 CRISPR knockout cells to determine whether FKBP12 is essential for their function.

2. For Figure 5E–H, Figure S8C–D and Figure S9E-H, a DMSO-treated control group should be included to serve as a proper baseline. In addition, treatment groups with 1 nM JQ1, a13c, and a1dj should also be included to allow for a more comprehensive comparison of compound efficacy at lower concentrations similar to the 1 nM treatment used for b3c. Additionally, all statistical analyses should be clearly indicated.
3. When describing the results, the authors should clearly indicate the corresponding figure panels (e.g., In THP1 cells, JQ1 showed comparable 391 cytotoxic activity as in MV4-11 (SI Fig. 9A, IC50 THP1: 70 nM; IC50 MV4-11: 34 nM (fig. 5A) etc.) to help readers follow the data.
4. The colors chosen for different treatment groups in the figures are too close, making them difficult to distinguish. The authors should consider using more contrasting colors to help readers more easily differentiate the groups.
5. The specific IC_{50} values for the data shown in Fig. 7D, S11A, and S11B should be provided. As currently presented, it is difficult to evaluate whether there is a clear positive correlation between FKBP12 abundance and the efficacy of the bifunctional compounds.
6. The Western blot data in Figure S11C should indicate the specific cell line used. Additionally, a proper control group should be included in Figure S11E.
7. There are a few typos (e.g., compasrtment).
8. Some experiments (e.g., Fig. 3) were done twice instead of typical 3 independent tests.

Reviewer #2

(Remarks to the Author)
Bulldan, Zheng and Meyners et al. describe the synthesis and characterization of non-catalytic heterobifunctional compounds that link the target protein BRD4 to the presenter protein FKBP12. They show that selected compounds induce the formation of ternary BRD4:compound:FKBP12 complexes, partially with substantial positive cooperativity based on stabilizing protein:protein-, protein:non-cognate-ligand 'crossover'-, and protein:linker- contacts.
Using FKBP12-depletion via a PROTAC, FKBP12 KO cells and a panel of myeloma cells expressing various levels of FKBP12, they show that the bioactivity of the non-catalytic heterobifunctional compounds is dependent on the presence of the presenter protein FKBP12. This opens a therapeutic window to selectively kill cells that express high levels of the presenter protein, as demonstrated in co-culture assays.
The pronounced increase in potency in the presence of the presenter protein cannot be explained by the positive cooperativity of the ternary complex alone. The authors conclude that the high affinity of the heterobifunctional compounds to the presenter protein results in an intracellular accumulation of the compounds, a concept they term 'CellTrap effect'. They suggest that this effect underlies the enhanced potency and selectivity of the heterobifunctional compounds compared to the monovalent parental BET inhibitor.

Originality and significance
Non-catalytic heterobifunctional compounds are conceptually not new. E.g. Raina et al. recently published a similar concept ("RIPTACs"), showing that heterobifunctional compounds that recruit ectopically expressed HaloTag-FKBP12 to various effector proteins, including BRD4, selectively kill HaloTag-FKBP12 expressing cells. They show 1) positive cooperativity of the ternary target:compound:effector protein complex and 2) accumulation of the compound in target protein expressing cells as two mechanisms contributing to enhanced selectivity and potency of heterobifunctional over parental monovalent compounds. Here, Bulldan, Zheng and Meyners et al. solidify the concept of non-catalytic heterobifunctional compounds and expand it to an endogenous presenter:target pair. They furthermore confirm positive cooperativity of the most potent compounds and present a mathematical model to explain how intracellular compound accumulation can drive enhanced potency.
However, alternative hypotheses for the mechanism of action of non-catalytic heterobifunctional compounds are plausible and data that convincingly shows that the CellTrap effect is causal for the enhanced efficacy of these compounds is lacking. To justify publication in Nature Communications, the authors should attempt to definitively solve the mechanism of action underlying enhanced potency of non-catalytic heterobifunctional compounds by a) solidifying evidence for the CellTrap effect and b) test contributions of alternative mechanisms.

Major points
1) Using LC-MS/MS or another feasible method, the authors should quantify intracellular compound concentrations to test if heterobifunctional compounds selectively accumulate in FKBP12 expressing over FKBP12 deficient cells. If yes, is this selective accumulation of compounds (in combination with positive cooperativity) sufficient to explain the drastically increased potency observed e.g. in INA-6 WT vs FKBP12 KO cells?
2) It seems at least plausible to assume that BRD4 regions or domains outside of isolated bromodomains could contribute contacts that stabilize the ternary BRD4:compound:FKBP12 complex. The in vitro binding assays using isolated BD1 or BD2 presented in this study are blind to such effects and might underestimate the true cooperativity. Cellular nanoBRET assays using full length BRD4-NanoLuc are reported in the study, but the data is not shown. The authors should include this data (plots) and use this or a similar assay to compare binding in the presence vs absence of FKBP12 (i.e. WT vs KO or +/- PROTAC treatment) to determine cooperativity of ternary complexes involving full length BRD4.
3) BRD4 is famously prone to induced degradation and several compounds have been shown to induce BRD4 degradation by unexpected mechanisms. Unexpected BRD4 degradation could contribute to the enhanced potency of the heterobifunctional compounds. Using immunoblotting or quantitative methods (e.g. fluorescent stability reporter or HiBiT/luciferase assays), the authors should test if the heterobifunctional compounds induce unexpected BRD4 degradation that could explain the enhanced potency.
4) Recruitment of the presenter protein by the heterobifunctional compounds could disrupt target protein function more

effectively than inhibition by the JQ1 warhead of the heterobifunctional compounds alone, for example via steric hindrance of endogenous protein-protein interactions (Schultze, Seamon and Zhao et al.), changes in subcellular localization (Ng et al.) or changes in chromatin localization. Using methods like co-IP/MS or proximity labelling, fluorescence microscopy and ChIP-Seq or Cut&Run, the authors should test if these alternative mechanisms contribute to the increased potency of the compounds in presence of the presenter protein.

Minor points:

The authors assume that the CellTrap effect requires excess presenter over target protein. It would be informative to titrate presenter protein levels, e.g. via PROTAC dilution series, to quantify the impact of the presenter:target protein ratio on the CellTrap effect.

Fig.4 would benefit from labels clarifying the protein names.

Fig.7D, mid panel, y-axis is missing a unit (i.e. is this % or fold enhancement?), and a measure for correlation (i.e. R value) should be added.

Line 417: “as the FKBP12-assisted biochemical affinities were only 2-5-fold higher (Tab.1)”. What number does this refer to? The Kd values for compound binding to BRD4 BDs given in Table 1 are 7-285-fold higher with FKBP12 compared to without FKBP12.

Reviewer #3

(Remarks to the Author)

I co-reviewed this manuscript with one of the reviewers who provided the listed reports. This is part of the Nature Communications initiative to facilitate training in peer review and to provide appropriate recognition for Early Career Researchers who co-review manuscripts.

Reviewer #4

(Remarks to the Author)

The manuscript builds upon the RIPTAC work pioneered by the Crews lab, with a focus on utilizing endogenously expressed targets. The authors have identified BRD4-FKBP12-based bifunctional molecules that demonstrate strong cooperative binding, resulting in the selective killing of cells with high FKBP12 expression, a mechanism termed CellTrap. These findings underscore the potential of leveraging highly expressed proteins to engage tumor-dependent targets, thereby inhibiting their activity to enhance drug efficacy and specificity. The paper is well-written and presents compelling data regarding the BRD4-driven mechanism. However, a key limitation is the translatability of this mechanism to other targets. Additionally, the concept itself is not entirely novel. I would recommend the manuscript for publication if the authors can address the following comments, specifically by demonstrating the CellTrap concept with another target and/or through in vivo applications.

Major Points

•The cell selectivity seems to hinge on weak binding affinity to BRD4. Although very interesting, the fact that the JQ1 portion has poor affinity to BRD4 seems serendipitous. The paper would be stronger if the authors could expand this application to other targets. At the very least, can the authors speculate if the learnings from their structural study on cooperativity can be more broadly applied? Can a retrospective analysis of these ternary structures be used to predict other targets with complementarity?

•To strengthen the paper, the authors should consider conducting in vivo experiments to demonstrate the efficacy and specificity of their molecules in animal models. This would provide a more comprehensive understanding of the therapeutic potential and pharmacokinetic properties of CellTrap.

•Are there specific residues essential for the observed cooperativity? If so, empirically mapping these mutants and testing for a reduction in binding would help validate the structures and provide clues to the translatability of the CellTrap mechanism.

•The authors focus their analysis on BRD4, but JQ1 engages other BET family members, such as BRD2 and BRD3. Did the authors test whether their bifunctionals also engage these proteins (both in their binary form and in conjunction with FKBP12)? This would provide insight into whether the glue-like mechanism extends beyond BRD4.

•The lack of BRD4 binding of the bifunctionals isn’t intuitive. Can the structure help explain this lack of binding? If so, it would be beneficial to add an explanation to the text.

•Figure 5: The IC50 to BRD4 in the presence of FKBP12 is similar to that of JQ1 (e.g. a1dj), yet it is over 300 times more potent in the cell-killing assay. Can the authors explain this discrepancy? Does the ternary complex have a slower off-rate to BRD4 compared to JQ1 alone? Does the ternary complex disrupt other protein-protein interactions, leading to more potent cell killing? Explaining this difference would improve the manuscript.

Minor points

•Line 301: Table 2 cited, but there is no Table 2 (referring to Table 1?)
•Line 308: Consider removing reference 26 (inaccessible without login credentials)
•Line 360: Remove citation to SI Fig. 8 in this sentence (no reference to a1d)
•Line 521: Define “ultra high-affinity” ligands
•Line 626: Remove PMID reference

•Line 630: Remove PMID reference
•Figure 3A and C are not cited in the text
•Figure 4B and C are not cited in the text
•Figure 4: Switch A), B), and C) panels with D) and F) so that the flow of the figure moves alphabetically from left to right.
•Figure 8: Caption does not match image. Switch caption for panels A) and B)
•Figure 8D is not cited in the text
•SI Figure 1: Fix typos in legend
•SI Figure 3: Would like to see negative control compounds (JQ1 and FKBP12 ligand alone)
•SI Figure 11C: Add cell lines above western blot image
•SI Figure 14C: Check compound labeling “1adj”
•SI Table 1: Clarify exit vector on JQ1 and FKBP12 ligands – remove alkyne handle if it is not present in the degrader conjugate
•SI Table 1: Fix typos in legend

Version 1:

Reviewer comments:

Reviewer #1

(Remarks to the Author)
The authors have addressed the major concerns raised. However, several relatively minor issues remain to be addressed.

1. In Fig 8D, the increase in a1dj levels in the culture medium upon treatment with the FKBP12 degrader 5a1 appears negligible, yet in SI Fig 11B, the identical treatment with 5a1 causes a remarkably prominent elevation in extracellular a1dj levels. The inconsistency between the results of Fig 8D and SI Fig 11B contradicts the authors’ claim that FKBP-mediated intracellular enrichment exerts only a mild effect on the concentration of a1dj in the culture medium. What is the explanation? Also it'd be important to provide more detailed statistical analysis (including the number of independent experimental replicates, applied statistical test methods, and exact P values). In addition, the figure legends for Fig 8D and SI Fig 11B should provide more detailed information on the drug treatment time and the exact administration concentration of all reagents used in the experiments.

2. Please provide statistical analysis for Fig 5E–H, Fig S8B and Fig S9F–J.

3. Please provide the IC50 values for Fig S12A, Fig S12B and Fig 8E.

Reviewer #2

(Remarks to the Author)
In this revised version of ‘Heterobifunctional Protein Binders Enable Cell Type-Specific Killing Through In-cell Enrichment’, the authors have by and large addressed the reviewer concerns by providing additional data.

In particular, they have solidified evidence for the claimed 'CellTrap' effect by quantifying intracellular accumulation of a bifunctional compound and found roughly 5 times higher intracellular compound concentration in the presence vs absence of presenter protein. They have also quantified compound binding and positive cooperativity of the ternary complex with a BRD4 construct harboring both bromodomains, and ruled out BRD4 degradation and changes in subcellular localization as potential alternative explanations for the enhanced compound potency.

Additional presenter protein-dependent mechanisms, most notably disruption of BRD4 protein-protein interactions or interference with chromatin engagement by recruitment of the presenter protein are not investigated in this work. This is problematic, because the extraordinary potency increase observed in the presence vs absence of presenter protein (e.g. 1144-fold for a1dj; Fig. 5C) does not align with the relatively mild presenter protein-dependent increase in intracellular compound concentration (~5-fold for a1dj; Fig. 8d), even when factoring in positive cooperativity of the ternary complex (~10-fold for a1dj; Table1 and SI Table 2). It thus seems likely that additional presenter protein-dependent effects, such as disruption of BRD4 protein-protein and protein-chromatin interactions play a role in the extraordinary cellular potency of the bifunctional compounds in the presence of presenter protein. This is currently only very briefly discussed in the manuscript (lines 521 – 523).
Before publication, I recommend that the text should be adjusted to clarify that the enhanced cellular potency in the presence of presenter protein is likely due to a combination of positive cooperativity, intracellular enrichment ('CellTrap') and other effects (e.g. disruption of PPIs, chromatin eviction,…), rather than attributing it entirely to the 'CellTrap effect' (lines 54-56, 474-475).

Reviewer #3

(Remarks to the Author)
I co-reviewed this manuscript with one of the reviewers who provided the listed reports. This is part of the Nature

Communications initiative to facilitate training in peer review and to provide appropriate recognition for Early Career Researchers who co-review manuscripts.

Reviewer #4

(Remarks to the Author)
As mentioned before, this is nice work and contributes to the generalizability of the RIPTAC approach. I maintain that the work appears to be an incremental advancement of RIPTAC, and that moving to in vivo study to test the CellTrap approach would have significantly increased the impact and scientific value of the manuscript. Other reviewers seem to share this sentiment. That said, I appreciate the authors' thorough responses and the robustness of the data, and I am generally supportive of moving forward with final publication.

Reviewer #5

(Remarks to the Author)
Bifunctional compounds that recruit FKBP12, including approaches such as dTAG, are likely to become increasingly important. While degron-based systems enable efficient protein degradation, they may not always achieve complete depletion. In this context, the strategy presented here—trapping target proteins via bifunctional compounds to modulate their function—provides a complementary and potentially valuable approach.
The ternary complex structures reported in this study offer important insights into FKBP12-mediated recruitment and, to the best of my knowledge, represent among the first such structural examples. These data provide a useful framework for understanding how protein–protein interactions and complex stability can be tuned in future compound design.
Notably, two distinct binding modes are observed, which are consistent with and help explain the biochemical and cellular data. The observed differences in orientation, including a tilted FKBP12 conformation in one structure, suggest that linker attachment at the exit vector of the JQ1 moiety may confer a degree of conformational flexibility. In contrast, alternative linker attachment (as in b3c) may impose more restricted orientations.
Overall, these findings provide a coherent structural basis for the reported activities. Further clarification and analysis of the points outlined below would help to strengthen the conclusions.

Major Comment 1
The structural interpretation of the linker region and its contribution to FKBP12 rotation would benefit from further clarification.
Two distinct structures of the ternary complex containing a1d are reported. In one structure, the N=N double bond within the triazole ring forms three hydrogen bonds (Fig. 4G; Supplementary Fig. 6F, H), which may stabilize protein–protein interactions. In contrast, these interactions are absent in the second structure, suggesting that the linker retains conformational flexibility.
The two structures differ by an approximately 30° rotation of FKBP12. Notably, the orientations of both the JQ1 moiety and the [4.3.1] bicyclic high-affinity ligand moiety remain largely unchanged, implying that linker flexibility may contribute to the observed FKBP12 rotation.
In addition, for BRD4BD2, the triazole ring may introduce steric interference with residue H437, potentially contributing to BD1/BD2 selectivity. Given that H437 lies along the exit vector of the acetyl-lysine moiety of JQ1, a similar effect may also apply to a13c (line 461).
To improve clarity, it would be helpful to more explicitly discuss the role of triazole-mediated hydrogen bonding in the main text. Furthermore, a structural alignment in which FKBP12 is superimposed and the relative rotation of BRD4BD1 is highlighted may provide a more intuitive visualization of the reported ~30° rotation. Such a representation would also strengthen the connection to the proposed linker flexibility and CellTrap mechanism.

Major Comment 2
The interpretation of the decreased HTRF signal for the FKBP12:b3c:BRD4BD1 ternary complex may require further clarification.
The decrease in HTRF signal is attributed to changes in distance (lines 233 and 235); however, the structural data suggest that the distance between the N-termini of FKBP12 and BRD4BD1 is shortened rather than increased compared to the FKBP12:adj:BRD4BD1 complex. This raises the possibility that the signal reduction may not be explained solely by a distance-dependent effect.
Notably, b3c exhibits the strongest binding affinity to BRD4BD1 in the presence of FKBP12 among the compounds listed in Table 1 ($K_d = 8 \pm 2$). This may indicate that the corresponding ternary complex adopts a more rigid conformation relative to those formed by other compounds.
In this context, it may be helpful to consider additional factors that could contribute to the reduced HTRF signal, such as potential steric interference between BRD4 and the antibody recognizing the His tag.

Major Comment 3
The relationship between the mathematical model and its interpretation appears to require clarification.
In Fig. 8C, line 380 states that "an increased intracellular concentration of ligand species available for BRD4 inhibition (free ligand + FKBP12-bound ligand)" correlates with FKBP12 levels. However, in the corresponding equations, a ligand term is included in addition to the FKBP12 term, and this ligand term does not appear to correlate with FKBP12 levels.
Similarly, the "intracellular concentration enhancement scales" described in line 478 do not appear to exhibit a linear relationship with presenter protein levels.
Taken together, these observations suggest that the current interpretation may not be fully consistent with the mathematical formulation of the model. Clarifying the assumptions underlying the model, or revising the description of these relationships,

would improve consistency and interpretability.

Major Comment 4
The possibility of simultaneous engagement of both bromodomains by the bifunctional compounds could be more explicitly discussed.
The bifunctional compounds bind BRD2/3/4 BD1 and BD2 in the presence of FKBP12 with affinities comparable to or slightly weaker than JQ1, and SI Table 2 further evaluates binding to both bromodomains. Binding is retained even when mutations that disrupt interaction with the JQ1 moiety on one bromodomain are introduced. In addition, the Kd values observed without mutations fall between those measured for each individual bromodomain.
These observations suggest that a fraction of the compounds may simultaneously engage both bromodomains. Furthermore, structural data indicate that FKBP12 binding does not fully occlude the space surrounding BRD4, raising the possibility that, in cells, FKBP12 could engage both domains via the bifunctional compound.
Such a binding mode could enhance the “CellTrap Effect” described in the manuscript and may be particularly relevant for proteins containing multiple homologous domains, such as BRDs. This interpretation is also consistent with the observation that higher FKBP12 levels potentiate the activity of the bifunctional compounds.

Minor Comments
1. Figure 4 layout and labeling
The arrangement of panels (A–J) in Figure 4 appears uneven in both vertical and horizontal alignment, and the overall panel sizes seem relatively small. In addition, the font size used for protein names in panels B and C appears small. Including explicit labels for compounds (e.g., “a1d”) within the structural representations would improve interpretability. If other compounds (e.g., b3c or JQ1) are shown, consistent labeling across panels would further enhance clarity.

2. Figure 5G color scheme
The color scheme in the bar graph of Figure 5G appears to be reversed and may need correction.

3. Use of italic formatting in figure labels
In Figures 5 and 9, as well as several Supplementary figures, panel labels (A, B, C, etc.) are presented in italics. If this formatting does not carry specific meaning, using a non-italic style may improve consistency across figures.

4. Equation notation in Figure 8C
In Figure 8C, the term “[L・FKBL]” should be revised to “[FKBL・L]” in the concentration enhancement equation for consistency.

5. Identification of compounds in structural figures
The two binding modes mediated by a1d and b3c exhibit different orientations. Clearly indicating which compound is shown in each structural representation would improve clarity. In particular, labeling compounds (e.g., “a1d”) in Supplementary Figures 4–6 is recommended.

6. Annotation of IC_{50} values
In Figures 5 and 8, as well as Supplementary Figures 9 and 11, explicitly indicating that the reported values correspond to IC_{50} would improve clarity (as shown in Supplementary Figure 9, panels M and N). In addition, the numerical values in Figure 6B appear to represent IC_{50} but are not clearly defined.

7. Representation style in Supplementary Figure 4
Sphere representations are used in Supplementary Figure 4. If there is no specific reason for this choice, a cartoon representation may improve clarity and consistency with other figures.

8. Supplementary Figure 6 presentation
In Supplementary Figure 6, labeling protein and compound names within the structural diagrams would improve clarity. Additionally, the orientations of BRD4 in panels E and F appear similar to those in panels A and B, and the 90° side view may therefore be redundant.

9. Reference correction (Line 217)
“SI Fig. 5A,B” should be corrected to “Fig. 4D,E and SI Fig. 6A,B”.

10. Description of interacting residues (Lines 217–220)
The FKBP–BRD4BD1 interacting residues are described such that residues in the 80s loop are presented consecutively, whereas residues in the β2 strand are listed separately. It would be helpful to clarify whether this distinction has specific significance. In addition, repositioning “FKBP12” later in the sentence may improve readability.

11. Consistency in residue presentation (Line 219)
For consistency, the β2 strand residues could be presented in the same format as those of FKBP12.

12. Residue labeling in structural figures
In Fig. 4 and Supplementary Fig. 6, adding labels to indicate the positions of residues involved in protein–protein interactions, as described in the main text, would improve clarity.

13. Overall figure formatting consistency

Across several figures, italic formatting appears overused, and label positioning and sizing are inconsistent. Standardizing font style, label placement, and size would improve overall clarity and readability.

Version 2:

Reviewer comments:

Reviewer #5

(Remarks to the Author)
The authors have made considerable efforts to address my comments and those of the other reviewers.

The additional a1d induced ternary structure of FKBP12 and BRD4BD2 indicates the linker around the triazole moiety can also participate in hydrogen-bonding interactions with BRD4BD2. Despite the similar a1d-mediated binding orientation observed for BRD4BD1 and BRD4BD2 toward FKBP12, these offer a further perspective of a1d-induced cooperativity and help explain the improved CellTrap efficacy without a strong preference for either BD domain.

Other comments have been addressed in the revised manuscript, and I support this study being added to the field of heterobifunctional molecules and molecular glues.

Open Access This Peer Review File is licensed under a Creative Commons Attribution 4.0 International License, which permits use, sharing, adaptation, distribution and reproduction in any medium or format, as long as you give appropriate credit to the original author(s) and the source, provide a link to the Creative Commons license, and indicate if changes were made.
In cases where reviewers are anonymous, credit should be given to 'Anonymous Referee' and the source.
The images or other third party material in this Peer Review File are included in the article's Creative Commons license, unless indicated otherwise in a credit line to the material. If material is not included in the article's Creative Commons license and your intended use is not permitted by statutory regulation or exceeds the permitted use, you will need to obtain permission directly from the copyright holder.
To view a copy of this license, visit https://creativecommons.org/licenses/by/4.0/

Revision summary 'Heterobifunctional Protein Binders Enable Cell Type-Specific Killing Through In-cell Enrichment'

We thank the reviewers and the editor for their constructive feedback, which we address as outlined below:

Reviewer #1 (Remarks to the Author) This study explores the functional effects of heterobifunctional molecules. The authors designed a number of compounds combining BRD4 inhibitor and FKBP12 ligands with various linkers, which were analyzed in depth by biochemical approaches including crystal structures. The key biological consequences triggered by these bifunctionals is a "CellTrap" effect: high cellular FKBP12 levels act like a trap, pulling more drugs into the cell and boosting its concentration and BRD4 inhibition. Th trap effect makes the molecules work at very low doses and allows them to distinguish between similar BRD4 domains. The study includes a model showing how FKBP12 abundance and drug affinity affect cell killing, proving that using common proteins as "presenters" can improve drug selectivity. This offers a new way to design drugs that target specific cell types with fewer side effects.

However, the mathematical model proposed should be described in greater detail to allow for proper evaluation of its assumptions. Moreover, the **experimental data do not fully support the proposed model, particularly the suggested correlation between FKBP12 abundance and compound efficacy. Additional experimental validation and quantitative analysis are needed to substantiate the model's biological relevance. Therefore, although the study reveals interesting findings, the molecular insights remain limited**.

Major concerns:

1.1. The authors propose that FKBP12 binding enriches bifunctional compounds intracellularly, enhancing their efficacy compared to JQ1. However, this conjecture requires stronger support. Specifically, **direct measurements of intracellular compound concentrations** are needed to confirm increased accumulation, which is dependent on FKBP12 abundance.

We agree that this is a key point, which we addressed with two complementary approaches. First, we directly measured the concentrations of the key compound a1dj in cell lysates of our primary model system MV4-11 by mass spectrometry with and without FKBP12 depletion as suggested by the reviewer. Second, we performed wash-off experiment that indicate cellular retention of bifunctional molecules through FKBP12 binding as shown by persistent myc repression. Taken together, these results confirm the postulated substantial FKBP12-dependent accumulation of bifunctional compounds in cells. We discussed this in the manuscript as:

To experimentally test the CellTrap hypothesis, we directly determined cellular concentrations of a1dj in MV4-11 cells. This compound accumulated in the cellular fraction up to an approximated concentration of >1.8 µM (Fig. 8D, SI Fig. 11A, see SI Fig. 11C for approximation parameters[28]), consistent with the estimated intracellular FKBP12 levels in these cells (SI Fig. 11F-H). 58% of the cellular accumulation was FKBP12-mediated (abolished by prior FKBP12 depletion). An additional 22% of cellular enrichment was mediated by other FKBPs, which was abolished by competition with a pan-FKBP ligand (18-(S)Me))[19]. The cellular enrichment was not mediated by BRD4 binding, since prior BRD4 depletion did not affect cellular compound levels (SI Fig. 11A). The extracellular compound levels were only mildly affected by the FKBP-mediated cellular enrichment, indicating negligible compound depletion in the extracellular compartment under these conditions (Fig. 8D and SI Fig. 11B). To further support the CellTrap concept, we performed a wash-off experiment in MV4-11 cells (SI Fig. 11D,E). The results demonstrate intracellular retention of the bifunctional compound a1dj via FKBP12 binding, as evidenced by persistent MYC repression up to 24 hours even after the compound was washed out.

1.2. Although the bifunctional compounds exhibit higher affinity for FKBP12 than for BRD4, the authors should examine **whether FKBP12 is present at a significantly higher abundance than BRD4**. This point is critical because the proposed mechanism—whereby FKBP12 acts as a "cellular trap" that enriches bifunctional compounds upon cell entry—relies on the assumption that FKBP12 is in large excess over BRD4. If the two proteins are present at similar levels, the compound may instead form binary complexes with BRD4 directly, and the contribution of FKBP12 to compound enrichment would be diminished.

This is indeed a key prerequisite for our study. We quantified the levels of FKBP12 and BRD4 in MV4-11 cells using global proteomics measurements performed through liquid chromatography-tandem mass spectrometry (LC-MS/MS). Specifically, for FKBP12, we obtained an intensity-based absolute quantification (iBAQ) value of 84,143 counts, indicating a relatively high abundance of this protein. In contrast, BRD4 exhibited an iBAQ value of 3,143 counts, reflecting its lower abundance in the same cellular context.

We also determined cellular concentrations by quantitative western blot and obtained a value of about 1.2 µM (SI Fig. 11 H). Due to the lack of a suitable combination of antibodies and recombinantly expressed BRD4 domains, we were unfortunately not able to determine BRD4 concentrations with this method. While more sophisticated measurements for BRD4 are needed for more precise assessment of the FKBP12 vs BRD4 ratio, our data support a substantial excess of FKBP12 over BRD4.

1.3. The study's model assumes an "undepletable extracellular drug reservoir" (i.e., constant [L]extracellular), but the authors also report sub-nanomolar IC50 values for bifunctionals. This seems paradoxical because: low effective concentrations imply limited drug availability: If the model requires excess drug to drive intracellular accumulation, how do bifunctionals work at picomolar levels?

The extracellular drug reservoir is treated as a constant in classical pharmacology. However, in a CellTrap scenario, depletion of extracellular drug levels can indeed become relevant. To address this, we measured extracellular drug levels (at the lower limits of detectability) and found them to be only mildly affected by intracellular drug accumulation. We discussed this as:

The extracellular compound levels were only mildly affected by the FKBP-mediated cellular enrichment, indicating negligible compound depletion in the extracellular compartment under these conditions (Fig. 8D and SI Fig. 11B).

Extracellular concentrations of compounds couldn't be analyzed at the sub-nanomolar levels used in the functional cellular assays. However, the applied amount of compounds (e,g, 100pM in 100µL = 10fMol) still exceeded the total amount of FKBP12 present in the cells (4000 cells x 1.1pL/cell x 1.25µM [FKBP12^{intracell}] = 5.5fMol), suggesting that the functional effects are still possible without FKBP12-induced depletion of the extracellular reservoir (note that only approx. 3% of FKBP12 needs to be occupied in cells to reach sufficiently high intracellular concentrations of the FKBP12 - compound complexes). We discussed this as:

We assumed an undepletable extracellular reservoir in our mathematical model (Fig. 8C). This was valid in the direct compound uptake assay, which used higher concentrations to allow compound quantification (Fig. 8D). The 'non-depletion' assumption might not be valid for highly potent compounds such as a1dj and b3c that are active at picomolar concentrations. However, mathematical modelling of a1dj and b3c at 100pM nominal concentration revealed that sufficient intracellular concentrations of FKBP12-bound compounds could be reached without substantial depletion of the extracellular reservoir.

In the present examples, the total amount of a1dj and b3c applied at 100pM robustly exceeds the levels of drug-loaded FKBP12 that are expected to be needed for BRD4 inhibition, as estimated from the data in Fig. 8D and the model in SI Fig. 11.

1.4. The model assumes that bifunctional compounds enter cells via unrestricted free exchange. However, recent studie (Wang Z, et al, Cell, 2025, 14:S0092-8674(25)00386-1.) shows that PROTACs enter cells via CD36-mediated endocytosis rather than free diffusion. Given the similar

size and physicochemical properties between PROTACs and bifunctionals, it is likely that the compounds in this study also rely on endocytic uptake. The authors should experimentally verify whether their bifunctional molecules enter cells through endocytosis, as this would fundamentally challenge the core assumption of free diffusion in their proposed model.

While an endocytosis-mediated uptake would help the bifunctionals to reach their target proteins (pharmacokinetic effect), it cannot explain the striking FKBP12-dependence (a pharmacodynamic effect), which is a consistent core finding for all our bifunctional molecules. We nevertheless evaluated the importance of CD36-mediated endocytosis by determining CD36 levels using western blots (as shown below). Strikingly, only MV4-11 cells showed substantial CD36 expression. The protein was almost non-detectable in other cell lines such as PC3, which showed similarly strong FKBP12-dependent BRD4 inhibition upon treatment with bifunctionals as MV4-11. To further determine the contribution of CD36-mediated processes to the observed cellular effects, we employed the published CD36 inhibitor sulfosuccinimidyl oleate (SSO) in MV4-11 cells. However, we did not observe any effect on myc repression upon a1dj treatment in these cells. This contrasts with the strong attenuation of BRD4 inhibition upon FKBP12 depletion (Fig. 5). Taken together, we observed no evidence for regulated uptake of bifunctional compounds that would explain the differential sensitivities of the investigated cell lines or the FKBP12-dependence of cellular effects. We are therefore confident that the proposed model assuming free diffusion remains relevant.

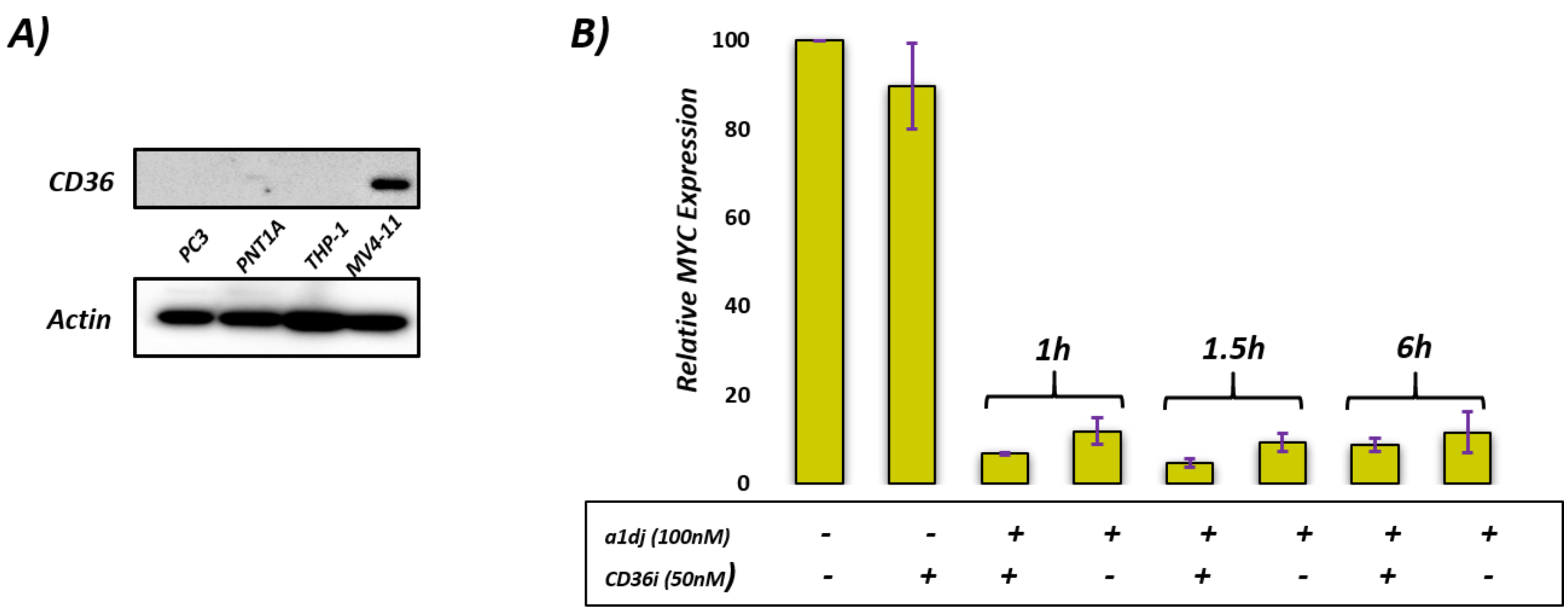

1.5 Minor points:

a) In Figures 5 and S8, the authors used an FKBP12-targeting PROTAC to reduce FKBP12 protein levels in order to illustrate the importance of FKBP12 abundance for the activity of these heterobifunctional molecules. The authors should treat cells with different concentrations of the

FKBP12-targeting PROTAC to examine how varying FKBP12 abundance affects the activity of the heterobifunctional molecules. Moreover, it would be important to test whether these molecules remain effective in FKBP12 CRISPR knockout cells to determine whether FKBP12 is essential for their function.

As suggested by the reviewer, we treated MV4-11 cells with increasing concentration of our FKBP12-targeting PROTAC and monitored the cellular effect of the bifunctional a1dj (SI Fig. 8C-D. Due to the catalytic nature of the PROTAC, it is not trivial to obtain a graded response on the level of the target protein. Nevertheless, we observed an increasing loss of a1dj activity when cells were treated with PROTAC concentrations ranging from 10 - 200nM. We discuss this in the manuscript as:

Notably, the influence of FKBP12 was concentration dependent as demonstrated by titration of the FKBP12-targeting PROTAC (SI Fig. 8C-D).

We tested our compounds in FKBP12-deficent INA-6 cells (Fig. 6, FKBP1A KO), which confirmed the strict FKBP12-depecence of our molecules. We also prepared a negative control of the key compound b3c where FKBP12-binding was dramatically reduced by a ring contraction (SI Fig. 9K-L). Compound b3c-neg was more than 1000-fold less active in MV4-11 and THP1 cells compared to b3c and the remaining activity was unaffected by FKBP12 depletion (SI Fig. 9M-N). We discuss this in the manuscript as:

To further confirm the FKBP12-independent residual activity of b3c, we synthesized a variant of the compound in which FKBP12-binding was dramatically reduced by a ring contraction (SI Fig. 9K-L) and determined its effect on cell survival. Strikingly, the IC_{50} of this compound was three orders of magnitude higher in MV4-11 and THP1 cells compared to b3c and the remaining activity was unaffected by FKBP12 depletion (SI Fig. 9M-N).

b) For Figure 5E–H, Figure S8C–D and Figure S9E-H, a DMSO-treated control group should be included to serve as a proper baseline. In addition, treatment groups with 1 nM JQ1, a13c, and a1dj should also be included to allow for a more comprehensive comparison of compound efficacy at lower concentrations similar to the 1 nM treatment used for b3c. Additionally, all statistical analyses should be clearly indicated.

As suggested by the reviewer, we updated Figure 5, SI Figure 8 and SI Figure 9 to include appropriate control conditions treated with solvent only (labeled “0 nM”) as well as treatment groups with 1 nM for the additional compounds besides b3c.

c) When describing the results, the authors should clearly indicate the corresponding figure panels (e.g., In THP1 cells, JQ1 showed comparable 391 cytotoxic activity as in MV4-11 (SI Fig. 9A, IC50 THP1: 70 nM; IC50 MV4-11: 34 nM (fig. 5A) etc.) to help readers follow the data.

We specified the respective figure panels showing the original data more closely as suggested, e.g.: 'In THP1 cells, JQ1 showed comparable cytotoxic activity as in MV4-11 (SI Fig. 9A: IC_{50}^{THP1}= 70 nM; Fig. 5A: $IC_{50}^{MV4\text{-}11}$= 34 nM).'

d) The colors chosen for different treatment groups in the figures are too close, making them difficult to distinguish. The authors should consider using more contrasting colors to help readers more easily differentiate the groups.

We increased the light/dark contrast of the different lines and colors to facilitate differentiation. We preferred to stick to similar colors for the same compounds as this helps the annotation to the specific compounds.

e) The specific IC_{50} values for the data shown in Fig. 7D, S11A, and S11B should be provided. As currently presented, it is difficult to evaluate whether there is a clear positive correlation between FKBP12 abundance and the efficacy of the bifunctional compounds.

The IC_{50} values for the data shown in Fig. 7D (now updated to 8D), S11A, and S11B were provided in the source data:

	IC50 values															
	JQ1-	error	JQ1+	error	a1dj-	error	a1dj+	error	a13c-	error	a13c+	error	b3c-	error	b3c+	error
ANBL6	240	160	230	150	1,6	0,4	200	50	350	80	1200	460	2,8	0,4	60	7
IH1	110	40	110	30	19	5	430	60	400	70	1200	120	4,9	0,6	50	3
INA6	89	6	77	8	1,4	0,3	210	80	28	14	960		3,2	0,4	54	8
JJN3	120	10	105	9	2,3	0,5	140	20	160	40	680	50	3,7	0,5	80	7
KJON	140	30	120	20	11	6	280	90	1100	260	1500	1000	4	0,5	36	6
OH2	110	20	110	20	2,4	0,6	100	20	100	20	950	100	1,8	0,2	24	4
RPMI	110		110		0,6	0,1	52	9	68	20	670	90	1,4	0,2	29	6

	Mean FKBP12 levels (Norm to GAPDH)	SE	JQ1/a1dj-PROTAC	error	JQ1/a1dj+ PROTAC	error	JQ1/a13c-PROTAC	error	JQ1/a13c+ PROTAC	error	JQ1/b3c-PROTAC	error	JQ1/b3c + PROTAC	error
ANBL6	2,60	0,22	150,00	106,80	1,15	0,80	0,69	0,48	0,19	0,14	85,71	58,44	3,83	2,54
IH1	1,27	0,24	5,79	2,60	0,26	0,08	0,28	0,11	0,09	0,03	22,45	8,61	2,20	0,61
INA6	3,85	0,90	63,57	14,28	0,37	0,14	3,18	1,60	0,08	0,01	27,81	3,95	1,43	0,26
JJN3	3,39	0,48	52,17	12,15	0,75	0,12	0,75	0,20	0,15	0,02	32,43	5,15	1,31	0,16
KJON	1,00	0,00	12,73	7,46	0,43	0,16	0,13	0,04	0,08	0,05	35,00	8,68	3,33	0,79
OH2	1,40	0,19	45,83	14,17	1,10	0,30	1,10	0,30	0,12	0,02	61,11	13,02	4,58	1,13
RPMI	3,88	1,05	183,33	30,56	2,12	0,37	1,62	0,48	0,16	0,02	78,57	11,22	3,79	0,78

Corresponding to these graphs:

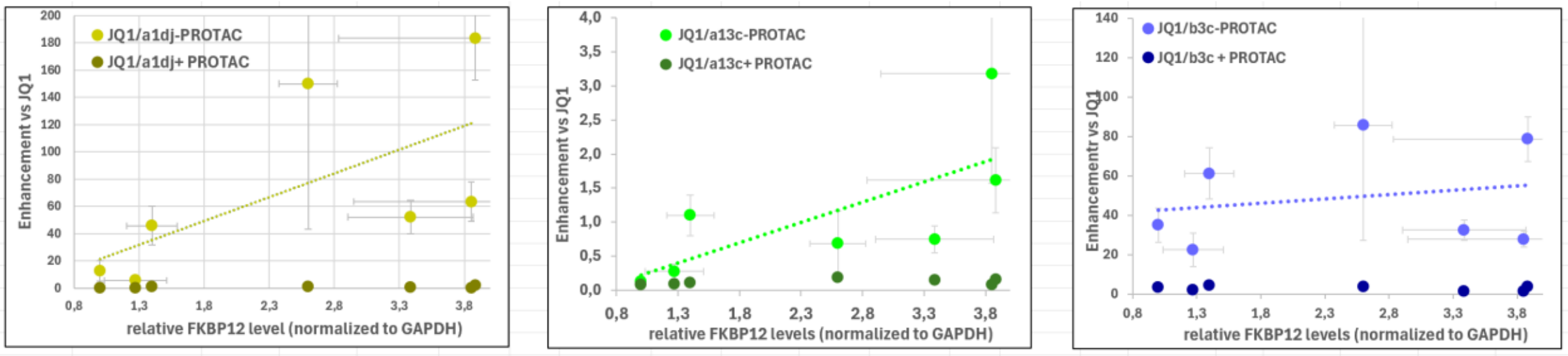

f) The Western blot data in Figure S11C should indicate the specific cell line used. Additionally, a proper control group should be included in Figure S11E.

We apologize that the labels of cell lines used got lost in the initial submission. This was rectified as shown below. In Figure S11E, the data is normalized to the house keeping gene GAPDH and the respective control group (ΔΔCT method). We adjusted the labeling of the graph to clarify this.

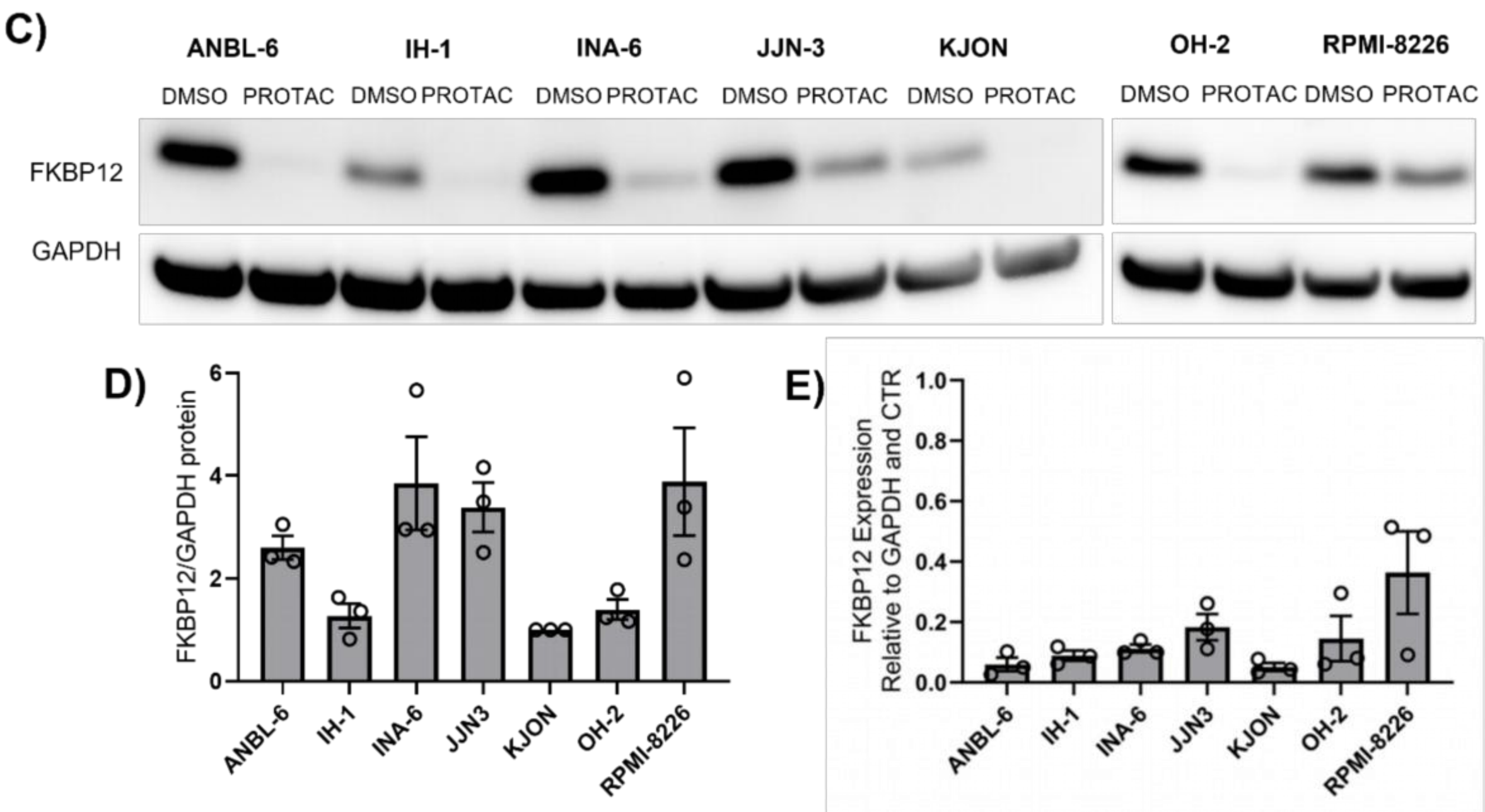

g) There are a few typos (e.g., compasrtment).

The manuscript was carefully checked for remained typos, which were corrected

h) Some experiments (e.g., Fig. 3) were done twice instead of typical 3 independent tests.

We repeated the experiment and the data now represent independent tests.

<u>Reviewer #2 (Remarks to the Author)</u>

Bulldan, Zheng and Meyners et al. describe the synthesis and characterization of non-catalytic heterobifunctional compounds that link the target protein BRD4 to the presenter protein FKBP12. They show that selected compounds induce the formation of ternary BRD4:compound:FKBP12

complexes, partially with substantial positive cooperativity based on stabilizing protein:protein-, protein:non-cognate-ligand 'crossover'-, and protein:linker- contacts.

Using FKBP12-depletion via a PROTAC, FKBP12 KO cells and a panel of myeloma cells expressing various levels of FKBP12, they show that the bioactivity of the non-catalytic heterobifunctional compounds is dependent on the presence of the presenter protein FKBP12. This opens a therapeutic window to selectively kill cells that express high levels of the presenter protein, as demonstrated in co-culture assays.

The pronounced increase in potency in the presence of the presenter protein cannot be explained by the positive cooperativity of the ternary complex alone. The authors conclude that the high affinity of the heterobifunctional compounds to the presenter protein results in an intracellular accumulation of the compounds, a concept they term 'CellTrap effect'. They suggest that this effect underlies the enhanced potency and selectivity of the heterobifunctional compounds compared to the monovalent parental BET inhibitor.

Originality and significance: Non-catalytic heterobifunctional compounds are conceptually not new. E.g. Raina et al. recently published a similar concept ("RIPTACs"), showing that heterobifunctional compounds that recruit ectopically expressed HaloTag-FKBP12 to various effector proteins, including BRD4, selectively kill HaloTag-FKBP12 expressing cells. They show 1) positive cooperativity of the ternary target:compound:effector protein complex and 2) accumulation of the compound in target protein expressing cells as two mechanisms contributing to enhanced selectivity and potency of heterobifunctional over parental monovalent compounds. Here, Bulldan, Zheng and Meyners et al. solidify the concept of non-catalytic heterobifunctional compounds and expand it to an endogenous presenter:target pair. They furthermore confirm positive cooperativity of the most potent compounds and present a mathematical model to explain how intracellular compound accumulation can drive enhanced potency.

However, alternative hypotheses for the mechanism of action of non-catalytic heterobifunctional compounds are plausible and data that convincingly shows that the CellTrap effect is causal for the enhanced efficacy of these compounds is lacking. To justify publication in Nature Communications, the authors **should attempt to definitively solve the mechanism of action underlying enhanced potency of non-catalytic heterobifunctional compounds by a) solidifying evidence for the CellTrap effect and b) test contributions of alternative mechanisms**.

<u>Major points</u>

2.1. Using LC-MS/MS or another feasible method, the authors should quantify intracellular compound concentrations to test if heterobifunctional compounds selectively accumulate in FKBP12 expressing over FKBP12 deficient cells. If yes, is this selective accumulation of compounds (in combination with positive cooperativity) sufficient to explain the drastically +increased potency observed e.g. in INA-6 WT vs FKBP12 KO cells?

We agree that this is a key point and addressed this as outlined above (Reviewer 1, point 1.1). In brief, we directly measured the concentrations of the key compound a1dj in MV4-11 cell lysates with and without FKBP12 depletion (SI Fig 11A-B) and confirmed the postulated substantial FKBP12-dependent accumulation in cell lysates. The modelling of the intracellular concentrations combined with the measured K_{D1} and K_{D2} values shows that this can explain the drastically increased potencies, see SI Fig. 11C.

2.2. It seems at least plausible to assume that BRD4 regions or domains outside of isolated bromodomains could contribute contacts that stabilize the ternary BRD4:compound:FKBP12 complex. The in vitro binding assays using isolated BD1 or BD2 presented in this study are blind to such effects and might underestimate the true cooperativity. Cellular nanoBRET assays using full length BRD4-NanoLuc are reported in the study, but the data is not shown. The authors should include this data (plots) and use this or a similar assay to compare binding in the presence vs absence of FKBP12 (i.e. WT vs KO or +/- PROTAC treatment) to determine cooperativity of ternary complexes involving full length BRD4.

We also determined the binding and cooperativity of our compounds to a BRD4 construct harboring both BD domains and the connecting sequence. This confirmed the medium cooperativity previously observed with the isolated BD domains. While we cannot exclude additional cooperative contacts with the outer regions of BRD4 this appear less likely in light of the unstructured nature of these protein sequences. We discussed this in manuscript as follows:

We also tested the advanced compounds a1dj, a13c, and b3c for binding to a protein construct harboring both bromodomains and the connecting endogenous protein sequence (SI Tab. 2). The selected compounds retained the positive cooperativity and $BRD4^{BD1}$ preference of a13c was retained, indicating that these properties are not influenced by crosstalk between both bromodomains or interactions with the linking sequence.

We also performed more sophisticated nanoBRET assay as requested, included as:

We also generated a stable cell line constitutively expressing BRD4–NanoLuc as a fusion protein and performed NanoBRET assays in the presence or absence of FKBP12 (SI Fig. 7). All bifunctional compounds tested displayed strong BRD4 engagement in the presence of FKBP12, whereas treatment with an FKBP12-targeting PROTAC markedly attenuated the apparent BRD4 occupancy of the bifunctional ligands. In contrast, the monovalent BRD4 ligand JQ1 showed only minimal target engagement and was unaffected by PROTAC treatment, consistent with its FKBP12-independent binding mode.

2.3. BRD4 is famously prone to induced degradation and several compounds have been shown to induce BRD4 degradation by unexpected mechanisms. Unexpected BRD4 degradation could contribute to the enhanced potency of the heterobifunctional compounds. Using immunoblotting or quantitative methods (e.g. fluorescent stability reporter or HiBiT/luciferase assays), the authors should test if the heterobifunctional compounds induce unexpected BRD4 degradation that could explain the enhanced potency.

As suggested by the reviewer, we tested for BRD4 degradation upon treatment of MV4-11 cells with our bifunctional compounds using western blot. We did not observe degradation of BRD4, but rather a stabilization of the protein for all compounds at high concentrations including JQ1. We also tested the effect of our key bifunctional a1dj in MV4-11 cells by global proteomics. a1dj did not affect BRD4 levels (or of any other protein), whereas the BRD4-PROTAC 43 potently did so.

	**bifunctional binder**	FKBP12 PROTAC		BRD4 PROTAC	
Gene	**10nM a1dj vs. DMSO**	100nM 5a1 vs. DMSO	100nM 5a1 + 10nM a1dj vs. DMSO	100nM 43 vs. DMSO	100nM 43 + 10nM a1dj vs. DMSO
FKBP1A	98%	6%	6%	95%	92%
FKBP3	85%	80%	79%	80%	65%
FKBP4	87%	75%	74%	81%	70%
FKBP2	90%	92%	86%	92%	80%
FKBP5	87%	80%	80%	84%	74%
FKBP8	88%	86%	84%	89%	76%
**BRD4**	**94%**	86%	86%	2%	1%
FKBP15	84%	79%	75%	72%	56%
BRD3	93%	84%	87%	0%	1%
BRD2	93%	80%	73%	1%	1%
BRD1	90%	88%	81%	80%	68%
BRD7	90%	75%	77%	76%	56%
BRD8	90%	91%	85%	73%	63%
FKBP7	78%	89%	92%	85%	79%
FKBP9	65%	111%	98%	94%	85%

We discuss this in manuscript as:

Importantly, none of the compounds tested induced degradation of BRD4 (SI Fig. 8E-H and SI Table 4).

2.4. Recruitment of the presenter protein by the heterobifunctional compounds could disrupt target protein function more effectively than inhibition by the JQ1 warhead of the heterobifunctional compounds alone, for example via steric hindrance of endogenous protein-protein interactions (Schultze, Seamon and Zhao et al.), changes in subcellular localization (Ng et al.) or changes in chromatin localization. Using methods like co-IP/MS or proximity labelling, fluorescence microscopy and ChIP-Seq or Cut&Run, the authors should test if these alternative mechanisms contribute to the increased potency of the compounds in presence of the presenter protein.

To investigate potential differences in the cellular effects of our bifunctional compounds compared to JQ1, we determined the resulting changes in gene expression by RNAseq. In brief, we observed very similar effects of JQ1, a1dj and b3c, which were clearly separated from cells treated with the non-FKBP12 binding variant b3c-neg (see Reviewer 1, point 1.5a) or solvent only. We discuss this in the manuscript as:

To investigate whether inhibition of BRD4 by FKBP12-BRD4 bifunctionals leads to different remodeling of the transcriptome compared to JQ1, we performed RNA sequencing in MV4-11 cells. Principal component analysis of the resulting data revealed similar effects on gene expression of JQ1, a1dj and b3c, which were clearly separated from cells treated with the non-FKBP12 binding variant b3c-neg or solvent only (Fig. 7A). As expected, BRD4 inhibition induced the differential expression of about 3000 genes, which were largely overlapping between the BRD4-targeting compounds (SI Fig. 10A and B). Clustering differentially expressed genes corroborated the closely related effects of JQ1, a1dj and b3c (Fig. 7B). When gene expression profiles of a1dj and b3c treated cells were compared to JQ1 – treated cells, only about 300 genes were identified as differentially expressed (Fig. 7C). Of note, these genes could not be clearly assigned to distinct biological processes (SI Fig. 10C-D).

We also determined whether our bifunctional compounds would induce changes in the subcellular localization of BRD4 using immunofluorescence, but did not observe substantial changes. We discuss this in the manuscript as follows:

As it was recently suggested that the formation of ternary complexes by heterobifunctional compounds could alter the subcellular localization of target proteins[8], we determined the subcellular location of BRD4 in PC3 cells by immunofluorescence (SI Fig. 13I-J). However, we did not observe any substantial alteration of the ratio between nuclear and cytoplasmic BRD4 levels.

Taken together, we did not observe any evidence for alternative mechanisms contributing to the increased potency of the bifunctional compounds and remain confident it that it can be mainly assigned to the proposed CellTrap effect.

2.5. Minor points:

a) The authors assume that the CellTrap effect requires excess presenter over target protein. It would be informative to titrate presenter protein levels, e.g. via PROTAC dilution series, to quantify the impact of the presenter:target protein ratio on the CellTrap effect.

We agree with the reviewer and addressed this as outlined above (Reviewer 1, point 1.5a). In brief, we titrated the FKBP12-targeting PROTAC in MV4-11 cells and observed a concentration depending modulation of the cytotoxic effect induced by a1dj. We added this data as SI Fig. 8C-D.

b) Fig.4 would benefit from labels clarifying the protein names.

The protein labels were included in Fig. 4 to facilitate interpretation of the Fig. → FH

c) Fig.7D, mid panel, y-axis is missing a unit (i.e. is this % or fold enhancement?), and a measure for correlation (i.e. R value) should be added.

The labelling of the y-axis protein in Fig. 7D (now Fig. 8D) was amended and the R value was included → FH

d) Line 417: "as the FKBP12-assisted biochemical affinities were only 2-5-fold higher (Tab.1)". What number does this refer to? The Kd values for compound binding to BRD4 BDs given in Table 1 are 7-285-fold higher with FKBP12 compared to without FKBP12.

The biochemical affinities (to $BRD4^{BD1}$ or $BRD4^{BD2}$) of our compounds are 2-5 higher than the affinities of JQ1, see Tab. 1, e.g. for binding to $BRD4^{BD1}$+ FKBP12: JQ1= 47nM, a1d=26nM, a13c= 20nM, a1dj= 32nM, b3c= 8nM. We clarified this in the text by amending:

Notably, the efficacies of our cooperative protein binders were orders of magnitude higher compared to JQ1. This observation cannot be explained by enhanced biochemical binding, as the FKBP12-assisted biochemical affinities were only 2 - 5-fold higher (Tab. 1).

Reviewer #4 (Remarks to the Author):

The manuscript builds upon the RIPTAC work pioneered by the Crews lab, with a focus on utilizing endogenously expressed targets. The authors have identified BRD4-FKBP12-based bifunctional molecules that demonstrate strong cooperative binding, resulting in the selective killing of cells

with high FKBP12 expression, a mechanism termed CellTrap. These findings underscore the potential of leveraging highly expressed proteins to engage tumor-dependent targets, thereby inhibiting their activity to enhance drug efficacy and specificity. The paper is well-written and presents compelling data regarding the BRD4-driven mechanism. However, a key limitation is the translatability of this mechanism to other targets. Additionally, the concept itself is not entirely novel. I would recommend the manuscript for publication if the authors can address the following comments, specifically by demonstrating the CellTrap concept with another target and/or through in vivo applications.

Major Points

4.1. The cell selectivity seems to hinge on weak binding affinity to BRD4. Although very interesting, the fact that the JQ1 portion has poor affinity to BRD4 seems serendipitous. The paper would be stronger if the authors could expand this application to other targets. At the very least, can the authors speculate if the learnings from their structural study on cooperativity can be more broadly applied? Can a retrospective analysis of these ternary structures be used to predict other targets with complementarity?

a) While a slightly reduced BRD4 binding was anticipated for the bifunctionals based on the switch from the tBu ether (as in JQ1) to the primary amide, the observed reduction was indeed much higher. We previously observed a similar reduction of binding affinity for bifunctionals for FKBP-targeting PROTACs and uncover a hydrophobic collapse as a basis for this (Geiger et al ACIE 2024).

b) The positive cooperativity for our compounds can be retrospectively rationalized based on our ternary complex structures. However, the prospective prediction of cooperativity is – to our best knowledge – a major challenge. Much more ternary structures will be necessary for this, as suggested by recent work on CRBN-based molecular glues (Petzold et al., Science 2025).

We discussed this in the manuscript by including:

The key bifunctionals had reduced BRD4 binding compared to JQ1 although all contained the same BRD4-binding moiety. We previously observed a similar reduction of binding affinity for bifunctionals for FKBP-targeting PROTACs, and uncover a hydrophobic collapse as a basis for this[24]. The positive cooperativity for our compounds can be retrospectively rationalized based on our ternary complex structures. However, for prospective prediction of cooperativity, many more ternary structures will be necessary, as suggested by recent work on CRBN-based molecular glues[29].

4.2. To strengthen the paper, the authors should consider conducting in vivo experiments to demonstrate the efficacy and specificity of their molecules in animal models. This would provide a more comprehensive understanding of the therapeutic potential and pharmacokinetic properties of CellTrap.

The current compounds were designed for mechanistic studies, not for in vivo experiments. To assess the possibility of animal studies we conducted an *in vitro* metabolic study for the two most advanced compounds (a1dj and b3c). Both compounds were found to be metabolically labile (see below). While we agree that it is exciting to explore the influence of the CellTrap effect on pharmacokinetic properties this must be explored in follow up studies, which require substantial medicinal chemistry optimization. With the current compounds we believe animal experiments are ethically not warranted.

Intrinsic clearance (Clint) data for a1dj (MSC3104193A-1) and b3c (MSC3104194A-1):

Sample	Species	Response at T=0	Stability	Intrinsic clearance (µL/min/mg)	k (1/min)	% Remaining at first time point	T_Half	Category
MSC3104193A-1	mouse	30400	1.02	422	0.211	32.1	3.29	High
MSC3104194A-1	mouse	132000	1.11	>500	0.752	2.33	<5	High

4.3. Are there specific residues essential for the observed cooperativity? If so, empirically mapping these mutants and testing for a reduction in binding would help validate the structures and provide clues to the translatability of the CellTrap mechanism. The ternary complex structures revealed two non-conserved loop/strand regions of FKBP12 to mediate the direct protein contacts to BRD4. To explore this, we measured binding of the homolog FKBP51, which differs in the loop/strand regions, to BRD4. We did not observe any cooperativity for any compound, strongly supporting the relevance of the crystallographically observed contact. We discussed this in the text as:

In both ternary complexes, contacts to $BRD4^{BD1}$ are formed by the tip of the so called 80s loop of FKBP12 ($H^{87}P^{88}G^{89}I^{90}$) and parts of the β2-strand 2 (D^{41}, R^{42}, K^{44}, $P^{45}F^{46}K^{47}$), (SI Fig. 5A, B). These residues are among the most divergent within the FKBP superfamily. For example, the larger homolog FKBP51 has $S^{118}L^{119}P^{120}K^{121}$ in the 80s-equivalent loop and D^{72}, R^{73}, $E^{75}P^{76}F^{77}V^{78}$ in the β2-strand. Strikingly, none of the key bifunctional compounds showed a cooperative binding to BD1 or BD2 in the context of FKBP51 (SI Tab. 3). This strongly supports the crucial role of direct

protein-protein contacts for the observed positive cooperativity of the compounds in the context of FKBP12.

4.4. The authors focus their analysis on BRD4, but JQ1 engages other BET family members, such as BRD2 and BRD3. Did the authors test whether their bifunctionals also engage these proteins (both in their binary form and in conjunction with FKBP12)? This would provide insight into whether the glue-like mechanism extends beyond BRD4.

We tested the advanced bifunctional molecules also for binding to the BD1 and BD2 domains of BRD2 and BRD3, in the absence and presence of FKBP12 (Fig. SI 5). The affinities for these domains were similar as for BRD4 (<2-fold difference). Interestingly, the selectivity of compound a13c for $BRD4^{BD1}$ over $BRD4^{BD2}$ was conserved for BRD2 and BRD3. We thus conclude that the glue-like mechanism extends beyond BRD4. We discussed this in the text as:

While we discuss the observed cellular effects in the context of BRD4 throughout this manuscript we cannot exclude that some of the cellular effects might be partially mediated via other target proteins such as BRD2 or BRD3.

4.5. The lack of BRD4 binding of the bifunctionals isn't intuitive. Can the structure help explain this lack of binding? If so, it would be beneficial to add an explanation to the text.

Since the structures were obtained in the presence of FKBP12, they do not inform on the binding of the compounds to BRD4 alone. The structure-activity relationships observed for the different analogs allow some hypotheses (e.g. hydrophobic collapse, influence of the exit vectors, etc), which we discuss in the manuscript as outlined above (see point 4.1).

4.6. Figure 5: The IC50 to BRD4 in the presence of FKBP12 is similar to that of JQ1 (e.g. a1dj), yet it is over 300 times more potent in the cell-killing assay. Can the authors explain this discrepancy? Does the ternary complex have a slower off-rate to BRD4 compared to JQ1 alone? Does the ternary complex disrupt other protein-protein interactions, leading to more potent cell killing? Explaining this difference would improve the manuscript.

We attribute the 300-fold higher cellular potency to the CellTrap effect, which is possible for bifunctionals such as a1dj but not for JQ1. This enables much higher intracellular concentrations for a1dj, as shown in new SI Fig. 11A. Due to the CellTrap effect, effective intracellular concentrations are reached at much lower doses for a1dj compared to JQ1, see modelling in SI Fig. 11C.

Our RNAseq analysis showed that our bifunctional compounds lead to similar changes in gene expression as JQ1 alone. While we cannot formally rule out disruption of other protein-protein interactions, we are confident that the cytotoxic effects of the bifunctional compounds are mainly based on BRD4 inhibition similar to JQ1. Please also see our comment to Reviewer 2, Point 2.4. We discussed this in the text as:

Although not observed in this work, we note that engaging the target with a presenter-compound pre-complex might be functionally different than blocking the target with a small molecule alone.

4.7.Minor points

a) Line 301: Table 2 cited, but there is no Table 2 (referring to Table 1?)

We apologize and corrected all citations of tables and figures.

b) Line 308: Consider removing reference 26 (inaccessible without login credentials) → We apologize. We deleted Ref. 26. The results of the Merck-ProxiDRUGS Structure Prediction Challenge will be presented at the TF Med Conference in April 2026 (https://www.tfmed.de/)

c) Line 360: Remove citation to SI Fig. 8 in this sentence (no reference to a1d)

The preceding half-sentence reads 'BRD4-FKBP12 bifunctionals showed higher efficacies…', which included a1d. For this reason, we also referred to SI Fig. 8.

d) Line 521: Define "ultra high-affinity" ligands

We amended this section and specified 'with high-affinity ligands (K_D<5nM)'. We expect that the CellTrap effect is even more pronounced for ultra high-affinity" ligands (K_D^{FKBP12}<0.5nM) but this remains to be shown in following work.

e) Line 626: Remove PMID reference

We removed the reference as suggested.

f) Line 630: Remove PMID reference

We removed the reference as suggested.

g) Figure 3A and C are not cited in the text

Fig. 3A and C are now cited in manuscript.

h) Figure 4B and C are not cited in the text

Fig. 4B and C are now cited in manuscript.

i) Figure 4: Switch A), B), and C) panels with D) and F) so that the flow of the figure moves alphabetically from left to right.

The panels were reordered as suggested

j) Figure 8: Caption does not match image. Switch caption for panels A) and B)

The captions were reordered as suggested

k) Figure 8D is not cited in the text

Fig. 8D is now cite as Fig. 9D as '…the population of PC3 cells could be efficiently depleted from the co-culture while PNT1A cells continued to proliferate (Fig. 9D).'

l) SI Figure 1: Fix typos in legend

The typos were fixed

m) SI Figure 3: Would like to see negative control compounds (JQ1 and FKBP12 ligand alone)

We performed a control experiment (see below) and as expected the HTRF signal is dependent on ternary complex formation. Blocking the binding on either side by addition of JQ1 or 18-(S^{Me}) prevents complex formation.

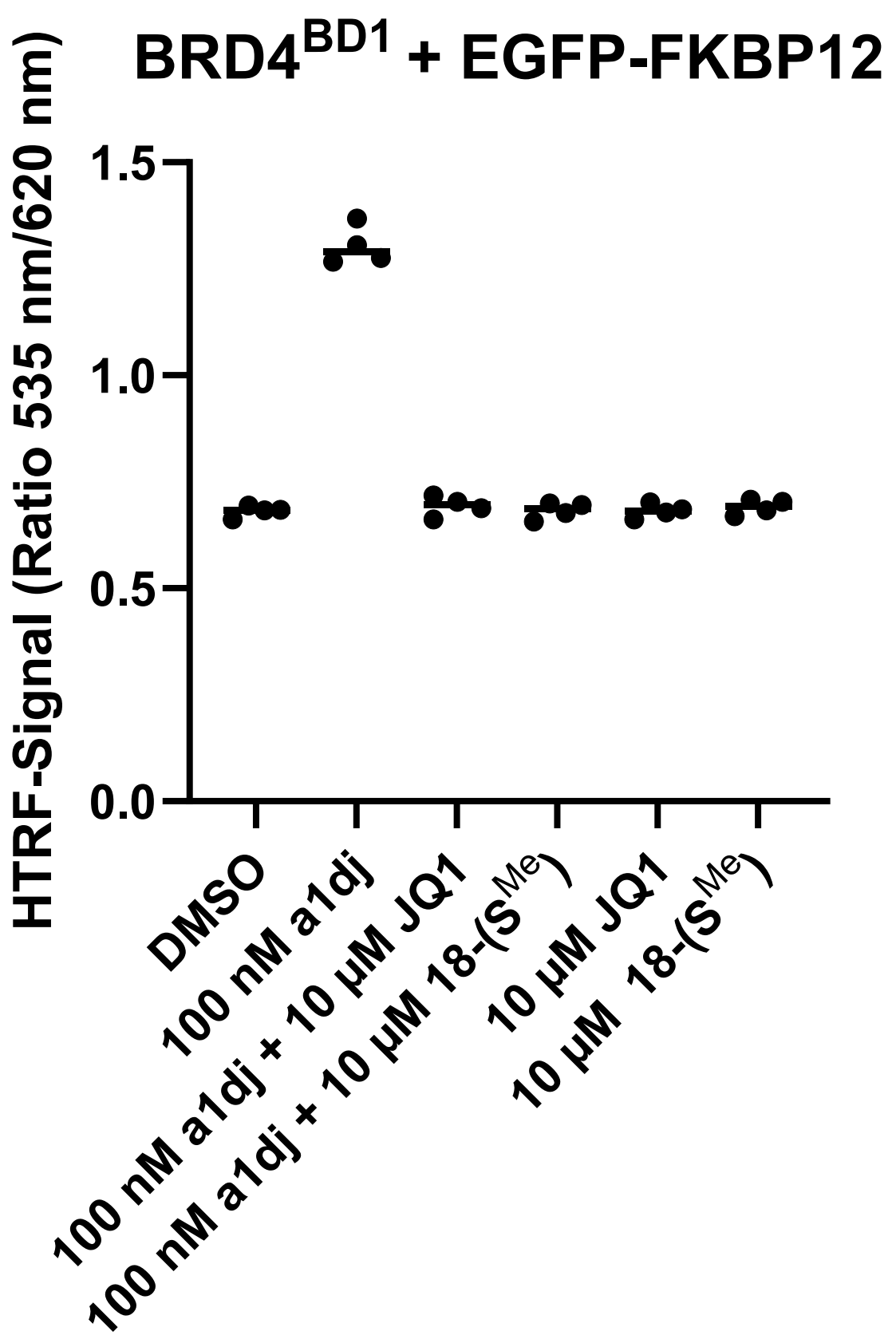

n) SI Figure 11C: Add cell lines above western blot image

The cell lines were indicated above the western blot image.

o) SI Figure 14C: Check compound labeling "1adj"

The label has been changed to a1dj.

p) SI Table 1: Clarify exit vector on JQ1 and FKBP12 ligands – remove alkyne handle if it is not present in the degrader conjugate

We preferred to show the BRD4 and FKBP12 ligands as well as the linkers in SI table 1 to be consistent with Fig. 2A of the main manuscript. However, to avoid any ambiguities we amended the legend of SI table 1 by including

Compounds were synthesized by amide coupling of the BRD4 ligand and the linker, followed by copper-catalyzed click reaction between resulting intermediate to the alkyne present in the FKBP12 ligand, ...

q) SI Table 1: Fix typos in legend

The typos were fixed.

Revision protocol

Bulldan, Zheng, Meyners et al.

Bifunctional Protein Binders Enable Cell Type-Specific Killing Through In-cell Enrichment

<u>Reviewer #1 (Remarks to the Author):</u>

The authors have addressed the major concerns raised. However, several relatively **minor issues remain** to be addressed.

1. In Fig 8D, the increase in a1dj levels in the culture medium upon treatment with the FKBP12 degrader 5a1 appears negligible, yet in SI Fig 11B, the identical treatment with 5a1 causes a remarkably prominent elevation in extracellular a1dj levels. The inconsistency between the results of Fig 8D and SI Fig 11B contradicts the authors' claim that FKBP-mediated intracellular enrichment exerts only a mild effect on the concentration of a1dj in the culture medium. What is the explanation? Also, it'd be important to provide more detailed statistical analysis (including the number of independent experimental replicates, applied statistical test methods, and exact P values). In addition, the figure legends for Fig 8D and SI Fig 11B should provide more detailed information on the drug treatment time and the exact administration concentration of all reagents used in the experiments.

We thank the reviewer for prompting a more rigorous cross-experiment statistical analysis. In response, we have expanded the presentation to include all four independent experimental rounds rather than individual runs, substantially increasing statistical power. All pairwise comparisons have been revised to use one-way ANOVA with Tukey HSD post-hoc correction. Tukey HSD-adjusted p-values are now displayed directly on both figures.

Intracellular a1dj was consistently and significantly reduced by 5a1 across all four independent experiments (41–58% reduction per round; pooled n=11, Tukey HSD $p<0.0001$ in all rounds and in the pooled analysis), confirming the robustness of the FKBP12-dependent cellular enrichment mechanism. Co-treatment with 5a1+43 produced an equally significant reduction (pooled Tukey HSD $p<0.0001$), while 43 alone had no significant effect versus DMSO ($p=0.859$).

Extracellular a1dj showed substantially higher inter-experiment variability (5a1/DMSO fold-change ranging from 0.98 to 3.75 across rounds), with significance reached in only 2 of the 4 experiments. Pooled analysis across all four rounds yields a modest but significant difference for 5a1 versus DMSO (Tukey HSD $p=0.031$, FC=1.58), while neither 43 nor 5a1+43 differed significantly from DMSO ($p=0.9999$ and $p=0.179$, respectively). We attribute the inter-experiment variability to technical factors, including non-specific adsorption of the lipophilic compound 5a1 to plastic surfaces and batch-to-batch differences in plates, rather than biological inconsistency. Notably, round 2 — where no extracellular effect was observed — still showed a significant 43% intracellular reduction ($p=0.006$), confirming that the cellular mechanism was fully operative.

This improved statistical analysis was implemented in the corresponding section of the manuscript, which now reads as:

"To experimentally test the CellTrap hypothesis, we directly determined cellular concentrations of a1dj in MV4-11 cells. This compound accumulated in the cellular fraction at a mean concentration of 1.68 ± 0.35 µM across four independent experiments (Fig. 8D, SI Fig. 12A, see SI Fig. 12C for approximation parameters[28]), consistent with the estimated intracellular FKBP12 levels in these cells (SI Fig. 12F-H). FKBP12 degradation by the PROTAC 5a1 resulted in a highly consistent reduction of intracellular a1dj accumulation across four independent experiments (mean 51% reduction, range 41–58%; pooled n=11 per group, one-way ANOVA with Tukey HSD $p<0.0001$), demonstrating that approximately half of the cellular enrichment is FKBP12-mediated. An additional 22% of cellular enrichment was mediated by other FKBPs, which was abolished by competition with a pan-FKBP ligand (18-(S)Me))[19]. The cellular enrichment was not mediated by BRD4 binding, since prior BRD4 depletion did not affect cellular compound levels (SI Fig. 12A). Extracellular a1dj levels showed a modest but significant increase upon treatment by the PROTAC 5a1 (pooled n=12 per group, Tukey HSD $p=0.031$, mean FC=1.58 vs DMSO across four experiments; SI Fig. 12B), as expected from reduced intracellular trapping upon FKBP12 degradation. The magnitude of this increase varied between experiments (FC range 0.98–3.75), probably reflecting technical factors including non-specific adsorption of the lipophilic compound to plastic culture surfaces and differences in DMSO concentrations across experiments (2% vs 3% final), rather than biological variability. This interpretation is supported by the high consistency of the intracellular data across all four rounds (Fig. 8D and SI Fig. 12B)."

Figures were updated as well, to include a better method description (compound concentrations and incubation times). SI Fig.12 now includes the results of four independent runs (n=9-12 per groups reported in the figure). Individual data points are also displayed as well as the p-values resulting from a one-way ANOVA Tukey HSD statistical analysis.

2. Please provide statistical analysis for Fig 5E–H, Fig S8B and Fig S9F–J.

We have included statistical analyses for Fig. 5E–H, Fig. S8B (now Fig. S9B), and Fig. S9F–J (now Fig. S10F-J).

3. Please provide the IC50 values for Fig S12A, Fig S12B and Fig 8E.

We have now included a table with the corresponding values as SI Fig. 13F:

(F) Quantification of IC_{50} values and FKBP12 expression levels from Fig. 8E and SI Fig. 13A and B.

F)

IC50 Values

Cell Line	JQ1-	JQ1 +PROTAC	a1dj -PROTAC	a1dj +PROTAC	a13c -PROTAC	a13c +PROTAC	b3c -PROTAC	b3c +PROTAC
ANBL6	240	230	1.6	200	350	1200	2.8	60
IH1	110	110	19	430	400	1200	4.9	50
INA6	89	77	1.4	210	28	960	3.2	54
JJN3	120	105	2.3	140	160	680	3.7	80
KJON	140	120	11	280	1100	1500	4.0	36
OH2	110	110	2.4	100	100	950	1.8	24
RPMI	110	110	0.6	52	68	670		

Mean FKBP12 Levels (Normalized to GAPDH)

Cell Line	Mean FKBP12
ANBL6	2.60
IH1	1.27
INA6	3.85
JJN3	3.39
KJON	1.00
OH2	1.40
RPMI	3.88

Enhancement vs JQ1

Cell Line	JQ1/a1dj - PROTAC	JQ1/a1dj + PROTAC	JQ1/a13c - PROTAC	JQ1/a13c + PROTAC	JQ1/b3c- PROTAC	JQ1/b3c+ PROTAC
ANBL6	150.00	1.15	0.69	0.19	85.71	3.83
IH1	5.79	0.26	0.28	0.09	22.45	2.20
INA6	63.57	0.37	3.18	0.08	27.81	1.43
JJN3	52.17	0.75	0.75	0.15	32.43	1.31
KJON	12.73	0.43	0.13	0.08	35.00	3.33
OH2	45.83	1.10	1.10	0.12	61.11	4.58
RPMI	183.33	2.12	1.62	0.16	78.57	3.79

Reviewer #2 (Remarks to the Author):

In this revised version of 'Heterobifunctional Protein Binders Enable Cell Type-Specific Killing Through In-cell Enrichment', the authors have by and **large addressed** the reviewer concerns by providing additional data.

In particular, they have solidified evidence for the claimed 'CellTrap' effect by quantifying intracellular accumulation of a bifunctional compound and found roughly 5 times higher intracellular compound concentration in the presence vs absence of presenter protein. They have also quantified compound binding and positive cooperativity of the ternary complex with a BRD4 construct harboring both bromodomains, and ruled out BRD4 degradation and changes in subcellular localization as potential alternative explanations for the enhanced compound potency.

Additional presenter protein-dependent mechanisms, most notably disruption of BRD4 protein-protein interactions or interference with chromatin engagement by recruitment of the presenter protein are not investigated in this work. This is problematic, because the extraordinary potency increase observed in the presence vs absence of presenter protein (e.g. 1144-fold for a1dj; Fig. 5C) does not align with the relatively mild presenter protein-dependent increase in intracellular compound concentration (~5-fold for a1dj; Fig. 8d), even when factoring in positive cooperativity of the ternary complex (~10-fold for a1dj; Table1 and SI Table 2). It thus seems likely that additional presenter protein-dependent effects, such as disruption of BRD4 protein-protein and protein-chromatin interactions play a role in the extraordinary cellular potency of the bifunctional compounds in the presence of presenter protein. This is currently only very briefly discussed in the manuscript (lines 521 – 523).
Before publication, I recommend that the text should be adjusted to clarify that the enhanced cellular potency in the presence of presenter protein is likely due to a combination of positive cooperativity, intracellular enrichment ('CellTrap') and other effects (e.g. disruption of PPIs, chromatin eviction,...), rather than attributing it entirely to the 'CellTrap effect' (lines 54-56, 474-475).

We amended the manuscript as follows:

'We attribute this effect mainly to the intracellular trapping of heterobifunctionals by the highly expressed FKBP12, which we termed 'CellTrap' effect (Fig. 8). The potency is further enhanced by the positive cooperativity upon ternary complex formation and possibly could contain other components such as disruption of PPIs by the tethered FKBP12. Assuming unrestricted equilibration of free ligand...'

Reviewer #3 (Remarks to the Author):

I co-reviewed this manuscript with one of the reviewers who provided the listed reports. This is part of the Nature Communications initiative to facilitate training in peer review and to provide appropriate recognition for Early Career Researchers who co-review manuscripts.

Reviewer #4 (Remarks to the Author):

As mentioned before, this is nice work and contributes to the generalizability of the RIPTAC approach. I maintain that the work appears to be an incremental advancement of RIPTAC, and that moving to in vivo study to test the CellTrap approach would have significantly increased the impact and scientific value of the manuscript. Other reviewers seem to share this sentiment. That said, I appreciate the authors' thorough responses and the robustness of the data, and I am generally **supportive of moving forward** with final publication.

Reviewer #5 (Remarks to the Author):

Bifunctional compounds that recruit FKBP12, including approaches such as dTAG, are likely to become increasingly important. While degron-based systems enable efficient protein degradation, they may not always achieve complete depletion. In this context, the strategy presented here—trapping target proteins via bifunctional compounds to modulate their function—provides a complementary and potentially valuable approach.
The ternary complex structures reported in this study offer important insights into FKBP12-mediated recruitment and, to the best of my knowledge, represent among the first such structural examples. These data provide a useful framework for understanding how protein–protein interactions and complex stability can be tuned in future compound design.
Notably, two distinct binding modes are observed, which are consistent with and help explain the biochemical and cellular data. The observed differences in orientation, including a tilted FKBP12 conformation in one structure, suggest that linker attachment at the exit vector of the JQ1 moiety may confer a degree of conformational flexibility. In contrast, alternative linker attachment (as in b3c) may impose more restricted orientations.
Overall, these findings provide **a coherent structural basis for the reported activities**. Further clarification and analysis of the points outlined below would help to strengthen the conclusions.

Major Comment 1
The structural interpretation of the linker region and its contribution to FKBP12 rotation would benefit from further clarification.

Two distinct structures of the ternary complex containing a1d are reported. In one structure, the N=N double bond within the **triazole ring forms three hydrogen bonds** (Fig. 4G; Supplementary Fig. 6F, H), which may stabilize protein–protein interactions. In contrast, these interactions are absent in the second structure, suggesting that the linker retains conformational flexibility.

The two structures differ by an approximately 30° rotation of FKBP12. Notably, the orientations of both the JQ1 moiety and the [4.3.1] bicyclic high-affinity ligand moiety remain largely unchanged, implying that linker flexibility may contribute to the observed FKBP12 rotation.

In addition, for BRD4BD2, the **triazole ring may introduce steric interference with residue H437**, potentially contributing to BD1/BD2 selectivity. Given that H437 lies along the exit vector of the acetyl-lysine moiety of JQ1, a similar effect may also apply to a13c (line 461).

To **improve clarity, it would be helpful to more explicitly discuss** the role of triazole-mediated hydrogen bonding in the main text. Furthermore, a structural alignment in which FKBP12 is superimposed and the relative rotation of BRD4BD1 is highlighted may provide a more intuitive visualization of the reported ~30° rotation. Such a representation would also strengthen the connection to the proposed linker flexibility and CellTrap mechanism.

The precise interactions of the linker with BRD4^{BD1} was indeed not easy to analyze in the provided 2D-figures. To facilitate this, we included new SI figures 6G and J. Please note that the geometries are not fully consistent with hydrogen bonds. For this reason we referred to them as 'triazole-contacting residues'.

We also included new SI figures 6K to specifically illustrate the rotation of the BRD4^{BD1} domains in the two structures of the two ternary complexes of a1d.

The residues in BRD4^{BD1} that are close to the triazole of the linker of a1d indeed deviate from the corresponding residues in BRD4^{BD2}. For this reason, we solved the crystal structure of the a1d-induced ternary complex of FKBP12 and BRD4^{BD2}, provided as new SI Fig. 7.

In the manuscript, we discuss the new figures as follows:

'In one complex, the triazole moiety of the linker is involved in interactions with BRD4^{BD1} (SI Fig. 6F and 6J). Notably, the triazole-contacting residues are different in BRD4^{BD1} (Asp^{101}, Asp^{102}, and Ile^{103}, SI Fig. 6G and J) vs BRD4^{BD2} (His^{437}, Glu^{438}, and Val^{439}). To investigate this, we solved the structure of the a1d-induced ternary complex between FKBP12 and BRD4^{BD2} (SI Fig. 7). We observed a rearrangement of the linker, which in the BRD^{BD2}-ternary complexes is stabilized by a hydrogen bond from the backbone of Val^{438} to His^{437}, which further forms a hydrogen bond to the oxygen of the linker (SI Fig. 7E and F). The linker conformation in the BRD^{BD2}-ternary complexes is similar to the linker conformation of the FKBP12-a1d-BRD4^{BD1a} complex (SI Fig. 7A and B) but FKBP12 can nonetheless adopt a similar orientation as in the FKBP12-a1d-BRD4^{BD1b} complex (SI Fig. 7C). Note that the different interaction patterns of BRD4^{BD1} and BRD4^{BD2} both resulted in energetically similar ternary complexes (Tab 1).'

SI Fig. 6G

SI Fig. 6J

SI Fig. 6K

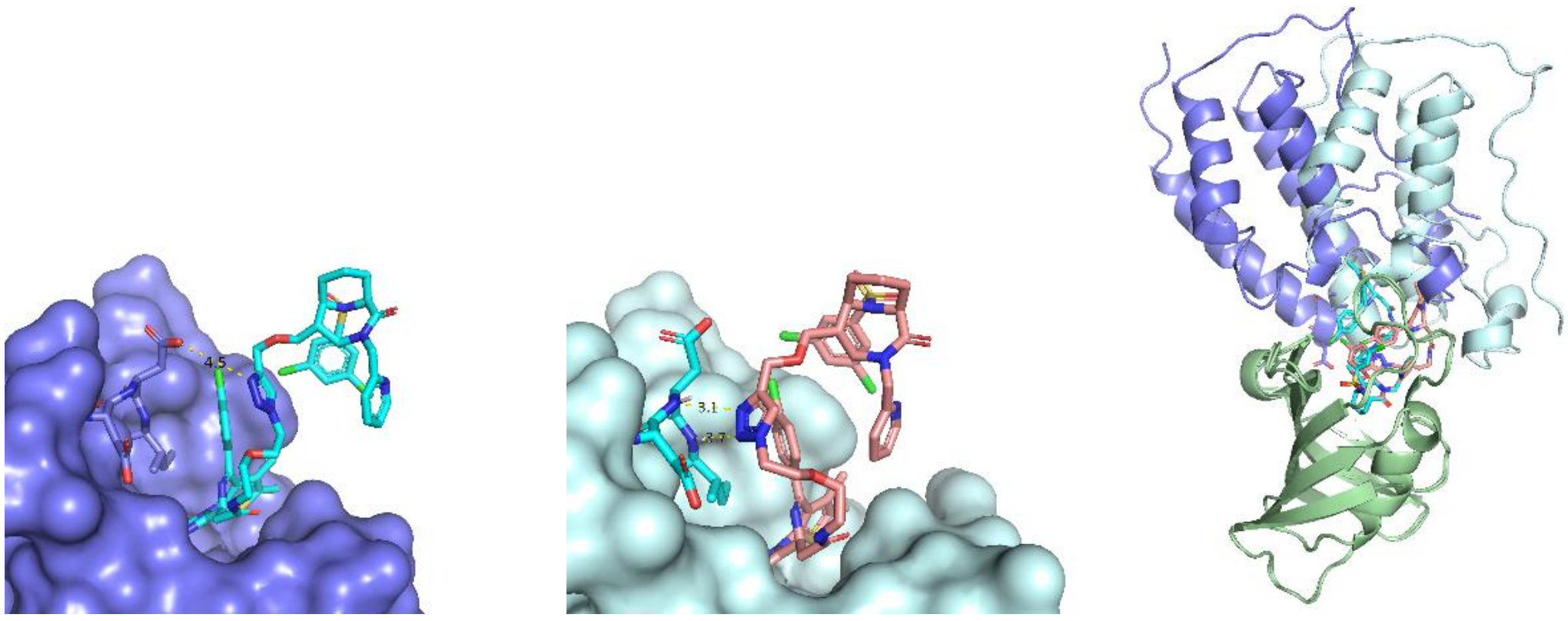

Amended legend of SI Fig. 6: '**(G,J)** Interaction of linker triazole moiety of **a1d** with BRD4^{BD1}. (**H,I**) **... (K)** Structures of the two FKBP12-a1d-BRD4^{BD1} complexes observed in the crystal, super-positioned by their FKBP12 unit. **a1d** is shown as cyan or pale pink sticks.'

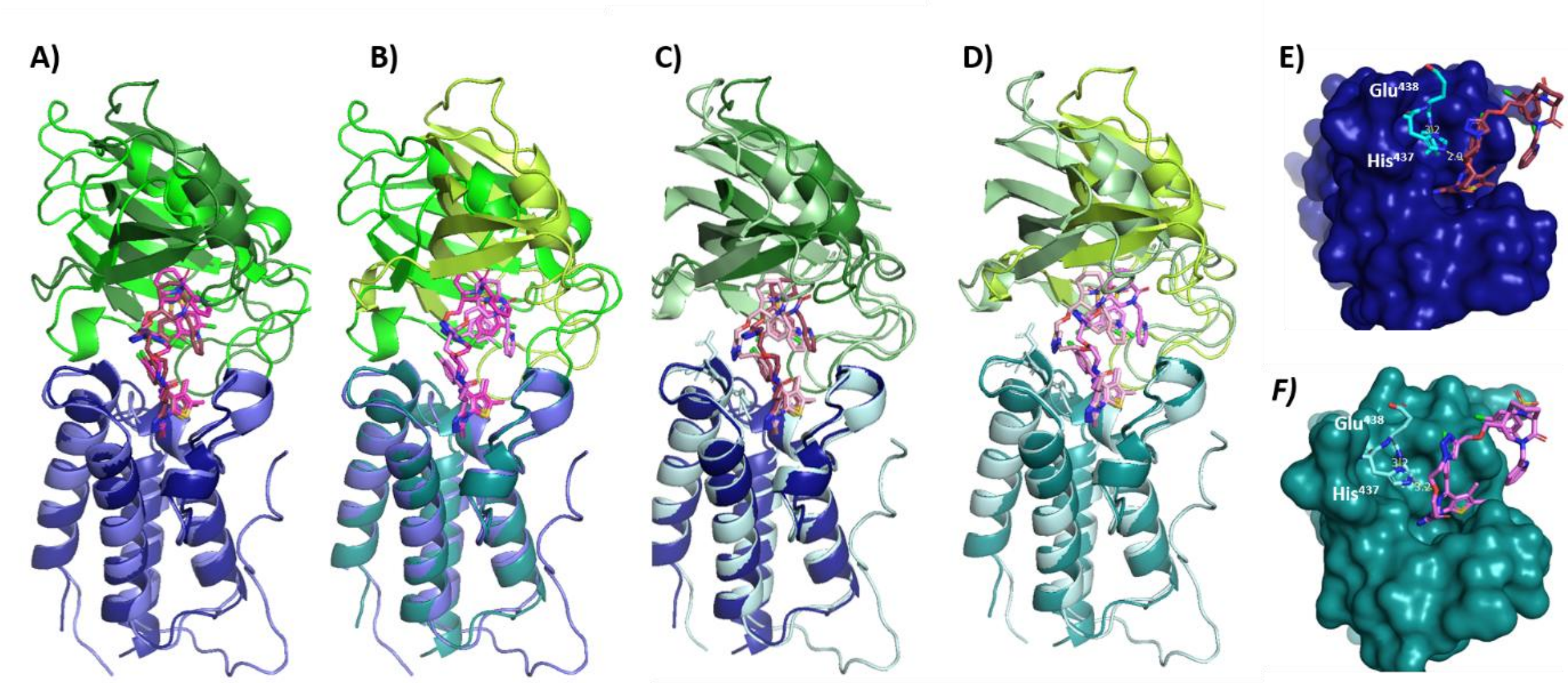

SI Fig. 7. Structure of the FKBP12:a1d:BRD4^{BD2} ternary complex (PDB-ID tbd). Two different ternary FKBP12:a1d:BRD4^{BD2} complexes are present in the crystallographic unit cell (sub-complexes BD2a and BD2b). In the FKBP12:a1d:BRD4^{BD2a} complex, FKBP12, a1d and BRD4^{BD2} are colored dark green, raspberry and dark blue, and in the FKBP12:a1d:BRD4^{BD2b} complex, FKBP12, a1d and BRD4^{BD2} are colored limon, violet and teal, respectively. **A-D)** Superposition of the two BRD4^{BD2}-containing ternary complexes on the each of the FKBP12:a1d:BRD4^{BD1} ternary complex (from PDB-ID 9QW8), aligned on the BRD4BD subunits. Proteins are shown as cartoons, a1d ist shown as sticks. **E,F)** Binding of a1d to BRD4^{BD2} in the ternary complexes (FKBP12 omitted for clarity). $H^{437}EV^{430}$ of BRD4^{BD2}-is highlighted as sticks. Hydrogen bonds from H^{437} to V^{430} to and the linker oxygen are shown as yellow dotted lines, with distances in Å.

Major Comment 2

The **interpretation** of the decreased HTRF signal for the FKBP12:b3c:BRD4BD1 ternary complex may **require** further **clarification**.

The decrease in HTRF signal is attributed to changes in distance (lines 233 and 235); however, the structural data suggest that the distance between the N-termini of FKBP12 and BRD4BD1 is shortened rather than increased compared to the FKBP12:adj:BRD4BD1 complex. This raises the possibility that the signal reduction may not be explained solely by a distance-dependent

effect.
Notably, b3c exhibits the strongest binding affinity to BRD4BD1 in the presence of FKBP12 among the compounds listed in Table 1 (Kd = 8 ± 2). This may indicate that the corresponding ternary complex adopts a more rigid conformation relative to those formed by other compounds.
In this context, it may be helpful to consider additional factors that could contribute to the reduced HTRF signal, such as potential steric interference between BRD4 and the antibody recognizing the His tag.

The different distances are difficult to assess in 2D-structural representations. We therefore provided quantitative measurements for the structurally resolved N-termini of FKBP12 and BRD4BD1 and presented these in the text as:

'(48.4Å and 53.3Å for the two b3c-induced complexes vs 25.2 Å and 29.4Å for the two b3c-induced complexes)'

HTRF signals are also affected by orientation, which we already had acknowledged as:

'…relative FKBP12-BRD4^{BD1} orientation was drastically different compared to the FKBP12:a1d:BRD4^{BD1} complexes'.

HTRF signals can indeed be affected by other effects, which we acknowledged as:

'b3c was the most potent ternary complex-inducing compound, which in turn could translate into a more rigid ternary complex. This as well as other effects such as altered antibody accessibility could have contributed to the reduced HTRF efficacies of the b3c-induced complexes.'

Major Comment 3
The relationship between the **mathematical model** and its interpretation appears to **require clarification**.
In Fig. 8C, line 380 states that "an increased intracellular concentration of ligand species available for BRD4 inhibition (free ligand + FKBP12-bound ligand)" correlates with FKBP12 levels. However, in the corresponding equations, a ligand term is included in addition to the FKBP12 term, and this ligand term does not appear to correlate with FKBP12 levels. Similarly, the "intracellular concentration enhancement scales" described in line 478 do not appear to exhibit a linear relationship with presenter protein levels.
Taken together, these observations suggest that the current interpretation may not be fully consistent with the mathematical formulation of the model. Clarifying the assumptions underlying the model, or revising the description of these relationships, would improve consistency and interpretability.

a) We agree with Reviewer and revised the sentence to 'an increased intracellular concentration of ligand species available for BRD4 inhibition (free ligand + FKBP12-bound ligand), which was positively driven by high FKBP12 levels.'

b) At $[L] << K_{D1}$ the equation on 'concentration enhancement' in Fig. 8C simplifies to $[FKBP]^{total}/K_{D1}$. To make this more clear we revised the corresponding sentence to 'The intracellular concentration enhancement scales linearly with presenter protein levels and for $[L] << K_{D1}$ approximately anti-proportionally with the affinity of the presenter protein'

Major Comment 4

The possibility of simultaneous engagement of both bromodomains by the bifunctional compounds could be more explicitly discussed.

The bifunctional compounds bind BRD2/3/4 BD1 and BD2 in the presence of FKBP12 with affinities comparable to or slightly weaker than JQ1, and SI Table 2 further evaluates binding to both bromodomains. Binding is retained even when mutations that disrupt interaction with the JQ1 moiety on one bromodomain are introduced. In addition, the Kd values observed without mutations fall between those measured for each individual bromodomain.

These observations suggest that a fraction of the compounds may simultaneously engage both bromodomains. Furthermore, structural data indicate that FKBP12 binding does not fully occlude the space surrounding BRD4, raising the possibility that, in cells, FKBP12 could engage both domains via the bifunctional compound.

Such a binding mode could enhance the "CellTrap Effect" described in the manuscript and may be particularly relevant for proteins containing multiple homologous domains, such as BRDs. This interpretation is also consistent with the observation that higher FKBP12 levels potentiate the activity of the bifunctional compounds.

We agree that a dual engagement of both BD domains is a possible scenario which we now acknowledge in the discussion as follows:

'It is also important to keep in mind that in cells two FKBP12-heterobifunctional complexes may be able to bind simultaneously to the BD1 and BD2 domains of full length BRD4.'

If the two FKBP12 proteins would productively interact with each other upon dual BD1/BD2 engangement this would indeed be especially important for the CellTrap effect. However, despite extensive studies on FKBP12, we have no evidence hinting at a dimerization of FKBP12. Therefore, we did not discuss this scenario as we believe it is currently too speculative.

Minor Comments

1. Figure 4 layout and labeling

The arrangement of panels (A–J) in Figure 4 appears uneven in both vertical and horizontal alignment, and the overall panel sizes seem relatively small. In addition, the font size used for protein names in panels B and C appears small. Including explicit labels for compounds (e.g., "a1d") within the structural representations would improve interpretability. If other compounds (e.g., b3c or JQ1) are shown, consistent labeling across panels would further enhance clarity.

We apologize and have adjusted the layout of the figure accordingly.

2. Figure 5G color scheme

The color scheme in the bar graph of Figure 5G appears to be reversed and may need correction.

We apologize for this oversight. The color scheme in the bar graph of Figure 5G was inadvertently switched and has now been corrected.

3. Use of italic formatting in figure labels

In Figures 5 and 9, as well as several Supplementary figures, panel labels (A, B, C, etc.) are

presented in italics. If this formatting does not carry specific meaning, using a non-italic style may improve consistency across figures.

We have revised Figures 5 and 9, as well as the relevant Supplementary Figures, to remove italic formatting from panel labels (A, B, C, etc.) and replaced it with a consistent non-italic style.

4. Equation notation in Figure 8C

In Figure 8C, the term "[L ・ FKBL]" should be revised to "[FKBL ・ L]" in the concentration enhancement equation for consistency.

The term was revised

5. Identification of compounds in structural figures

The two binding modes mediated by a1d and b3c exhibit different orientations. Clearly indicating which compound is shown in each structural representation would improve clarity. In particular, labeling compounds (e.g., "a1d") in Supplementary Figures 4–6 is recommended.

All compounds were clearly indicated in the legend. Compounds names were now also added to SI Fig. 4. SI Figures 5 & 6 only displayed one compound – we therefore did not show the compounds name in the figure.

6. Annotation of IC_{50} values

In Figures 5 and 8, as well as Supplementary Figures 9 and 11, explicitly indicating that the reported values correspond to IC_{50} would improve clarity (as shown in Supplementary Figure 9, panels M and N). In addition, the numerical values in Figure 6B appear to represent IC_{50} but are not clearly defined.

We thank the reviewer for this suggestion. We have revised Figures 5 and 8, as well as Supplementary Figure 9 (now 10), to explicitly indicate that the reported values correspond to IC_{50}. Supplementary Figure 11 does not include IC_{50} data, and therefore no changes were made to this figure. In addition, we have clarified in the legend of Figure 6B that the numerical values shown represent IC_{50} values.

7. Representation style in Supplementary Figure 4

Sphere representations are used in Supplementary Figure 4. If there is no specific reason for this choice, a cartoon representation may improve clarity and consistency with other figures.

We highlighted the N-terminal residues to illustrate the change in distance. We explained this in the legend of Fig. 4 as:

'The N-terminal residues of each protein are highlighted as spheres.'

8. Supplementary Figure 6 presentation

In Supplementary Figure 6, labeling protein and compound names within the structural diagrams would improve clarity. Additionally, the orientations of BRD4 in panels E and F appear similar to those in panels A and B, and the 90° side view may therefore be redundant.

Compound a1d is indicated in the legend (there is only one compound). The protein names are shown in SI Fig. 4A&B.

9. Reference correction (Line 217)
"SI Fig. 5A,B" should be corrected to "Fig. 4D,E and SI Fig. 6A,B".

The label has been corrected

10. Description of interacting residues (Lines 217–220)
The FKBP–BRD4BD1 interacting residues are described such that residues in the 80s loop are presented consecutively, whereas residues in the β2 strand are listed separately. It would be helpful to clarify whether this distinction has specific significance. In addition, repositioning "FKBP12" later in the sentence may improve readability.

We repeated the mentioning of FKBP12 to improve readability.

In the 80s loop, all consecutive residues interact with BRD4BD1 (H87P88G89I90) and are in turn all mentioned. In the beta2-strand only the mentioned residues interact with BRD4BD1 (D41, R42, K44, P45F46K47).

11. Consistency in residue presentation (Line 219)
For consistency, the β2 strand residues could be presented in the same format as those of FKBP12.
All amino acids are now separated by a komma

12. Residue labeling in structural figures
In Fig. 4 and Supplementary Fig. 6, adding labels to indicate the positions of residues involved in protein–protein interactions, as described in the main text, would improve clarity.
The interaction positions were indicated in SI Fig. 6. We opted not to show them in the main figure 4 as it added detail but substantially reduced clarity.

13. Overall figure formatting consistency
Across several figures, italic formatting appears overused, and label positioning and sizing are inconsistent. Standardizing font style, label placement, and size would improve overall clarity and readability.

We have carefully revised all figures to improve overall formatting consistency. Specifically, we reduced the use of italic formatting and standardized font style, label size, and label placement across all figures.
